# Higher-Order Structure of Adeno-Associated Virus Serotype 8 by Hydrogen/Deuterium Exchange Mass Spectrometry

**DOI:** 10.3390/v16040585

**Published:** 2024-04-10

**Authors:** Tomohiko Ikeda, Yuki Yamaguchi, Hiroaki Oyama, Aoba Matsushita, Yasuo Tsunaka, Mitsuko Fukuhara, Tetsuo Torisu, Susumu Uchiyama

**Affiliations:** 1 Department of Biotechnology, Graduate School of Engineering, Osaka University, 2-1 Yamadaoka, Suita 565-0871, Osaka, Japan; tomohiko.ikeda@bio.eng.osaka-u.ac.jp (T.I.); yuki.yamaguchi@bio.eng.osaka-u.ac.jp (Y.Y.); hiroaki.oyama.osaka.u@gmail.com (H.O.); aoba.matsushita@bio.eng.osaka-u.ac.jp (A.M.); yasuo.tsunaka@bio.eng.osaka-u.ac.jp (Y.T.); mitsuko.fukuhara@bio.eng.osaka-u.ac.jp (M.F.); tetsuo.torisu@bio.eng.osaka-u.ac.jp (T.T.); 2 Exploratory Research Center on Life and Living Systems (ExCELLS), National Institutes of Natural Sciences, 5-1 Higashiyama, Myodaiji, Okazaki 444-8787, Aichi, Japan

**Keywords:** gene therapy, adeno-associated virus, hydrogen/deuterium exchange mass spectrometry, higher-order structure

## Abstract

The higher-order structure (HOS) is a critical quality attribute of recombinant adeno-associated viruses (rAAVs). Evaluating the HOS of the entire rAAV capsid is challenging because of the flexibility and/or less folded nature of the VP1 unique (VP1u) and VP1/VP2 common regions, which are structural features essential for these regions to exert their functions following viral infection. In this study, hydrogen/deuterium exchange mass spectrometry (HDX-MS) was used for the structural analysis of full and empty rAAV8 capsids. We obtained 486 peptides representing 85% sequence coverage. Surprisingly, the VP1u region showed rapid deuterium uptake even though this region contains the phospholipase A2 domain composed primarily of α-helices. The comparison of deuterium uptake between full and empty capsids showed significant protection from hydrogen/deuterium exchange in the full capsid at the channel structure of the 5-fold symmetry axis. This corresponds to cryo-electron microscopy studies in which the extended densities were observed only in the full capsid. In addition, deuterium uptake was reduced in the VP1u region of the full capsid, suggesting the folding and/or interaction of this region with the encapsidated genome. This study demonstrated HDX-MS as a powerful method for probing the structure of the entire rAAV capsid.

## 1. Introduction

Adeno-associated virus (AAV) is a non-enveloped virus belonging to the genus Dependoparvovirus in the family Parvoviridae [1]. The AAV is composed of single-stranded DNA encoding rep/cap genes flanked by two inverted terminal repeats (ITRs) in the T = 1 icosahedral capsid [2]. The assembled AAV capsid consists of 60 subunits involving three different viral proteins (VPs), which are a mixture of VP1, VP2, and VP3 at an estimated molar ratio of 5:5:50 [3,4,5]. VP1 contains the entire VP2 sequence at its C-terminus and a unique (VP1u) region. Meanwhile, VP2 contains the entire sequence of VP3 and the VP1/VP2 common region. Recombinant AAV (rAAV), in which the rep/cap genes are replaced with a therapeutic gene, is used as a viral vector for *in vivo* human gene therapy because of its wide tissue tropism, low pathogenicity, and ability to sustain the long-term gene expression of a transgene [2,6,7,8]. Although several products have already been approved in the US and a large number of clinical trials are ongoing [6,9], the detailed structural mechanism underlying the biological function of rAAV capsids remains elusive. To develop rAAV capsid engineering and quality control, there is a need to clarify the mechanisms by which VP1 and VP2 N-terminal regions, which are supposed to be located inside the capsid prior to rAAV infection, are externalized in order to exert their functions, such as phospholipase A2 activity for endosomal escape and nuclear localization signaling to enter the nucleus [10,11]. There is also a need to determine how structural rearrangement of the capsid protein occurs during genome release [12].

rAAV capsid structural analysis has been conducted by cryo-electron microscopy (cryo-EM) and X-ray crystallography [13,14]. rAAV capsid structures for several serotypes have been determined in atomic detail, except for VP1u, VP1/VP2 common, and the N-terminal residues of VP3. The prominent surface characteristics of the capsid can be described as a depression at the 2-fold symmetric axis, a protrusion surrounding the 3-fold symmetric axis, and a channel at the 5-fold symmetric axis [15]. The structural topology of the resolved VP3 region was found to be highly conserved among all serotypes. The VP3 region contains one α-helix and nine β-strands (βA-βI). Two four-stranded antiparallel β-sheets composed of βB to βI adopt the jelly roll fold. This fold is common among viral capsid proteins and constitutes a fundamental structure during rAAV capsid assembly [16]. Contrary to the conserved regions, rAAV also contains nine variable regions (VRs), which are structurally and sequentially diverse across serotypes [17]. These VRs contain loop structures, and their biological functions are diverse, such as vector tropism, binding sites with extracellular receptors, and immune evasion [18,19,20].

The infectious pathway and transition (or rearrangement) of rAAV capsid structures have been extensively studied [10,12,21]. rAAV infection begins with the binding of rAAV to cellular receptors, such as heparan sulfate and sialic acids [22,23]. Once rAAV attaches to the cell surface, it engages with membrane-bound coreceptors. Subsequently, rAAV is internalized through clathrin-coated pits and transported into the cell via the endocytic pathway [24,25]. rAAV, transported to the perinuclear region by the endosome, is believed to escape from the endosome and be transported into the nucleus by importins recognizing the nuclear localization signal (NLS) [26,27]. Several reports have suggested that its endosomal escape involves the functions of the phospholipase A2 (PLA2) domain of VP1u [12,28]. The NLS in the VP1/VP2 common region of rAAV contributes to its transport into the nucleus. In our previous studies, a significant correlation was observed between the total incorporation of rAAV capsid VP1 and VP2 into the capsid and the transduction efficiency of rAAV. This observation indicated that both the PLA2 domain in the VP1u and NLS in the VP1u and VP1/VP2 common regions also make significant contributions to the gene delivery of rAAV [29]. From these perspectives related to the rAAV infectious pathway, the structurally unsolved VP1u and VP1/VP2 common regions play crucial roles in rAAV infection.

Despite extensive research on rAAV, structural information on the regions corresponding to VP1u, the VP1/VP2 common region, and the N-terminal residues of VP3 has not been determined. The number and location of VP1 and VP2 in the 60 subunits constituting a capsid vary, resulting in a population of stoichiometrically and structurally heterologous rAAV particles, even after purification [5,29]. Moreover, detecting the VP1u and VP1/VP2 common regions requires analytical methods with high sensitivity and specificity because of the low incorporation of VP1 and VP2 into the capsid. Thus, higher-order structural analysis of the entire rAAV capsid is challenging. Hydrogen/deuterium exchange mass spectrometry (HDX-MS) is a powerful method for observing protein structural dynamics and interactions in the solution state [30,31]. HDX-MS has been applied to assess the higher-order structure (HOS) similarity between antibody drugs and their biosimilars, and it has already been used to characterize several viral capsids, namely dengue virus and minute virus of mice (MVM) [32,33,34,35].

The rate of deuterium uptake in HDX-MS is highly dependent on the solution pH and temperature [36,37]. In standard automated HDX-MS systems, labeling with D_2_O buffers is performed at 20 °C, and labeling is detected at several time points, including 30 s as the earliest one. The advantage of an automated HDX-MS system is that all steps of labeling, quenching, digestion, desalting, peptide separation, and MS detection can be carried out automatically, realizing high reproducibility of HDX-MS data to reveal minor conformational differences in each protein state. However, deuterium exchange is rapid for proteins with high structural flexibility, such as disordered proteins, and deuterium uptake reaches a plateau in approximately 30 s at 20 °C. Labeling at lower temperatures, such as 4 °C, and with labeling detection at earlier times of less than 30 s can only be performed manually to overcome this issue [38].

In this study, we combined manual labeling and semi-automated HDX-MS to evaluate the HOS and dynamics of the entire rAAV capsid at labeling temperatures of 4 and 20 °C. Our HDX-MS results were associated with more than 85% sequence coverage, including the structurally unsolved VP1u and VP1/VP2 common regions. The loop structures exhibited high deuterium uptake, and regions with secondary structures, such as the conserved α-helix and nine β-strands, demonstrated relatively low deuterium uptake, as expected from the cryo-EM structures. Surprisingly, the VP1u region showed rapid deuterium uptake, although the region contains a phospholipase A2 domain mainly composed of α-helices [21,39]. The comparison of deuterium exchange uptake between full and empty capsids revealed the impact of the encapsidated genome on capsid structure. This study is the first application of HDX-MS to rAAV for the HOS assessment of rAAV-based therapeutics.

## 2. Materials and Methods

### 2.1. rAAV Samples

rAAV8 particles were produced by the triple transfection method. HEK293 cells were harvested 96 h post-transfection of transgene plasmids (CMV-EGFP) (Vector Builder, Chicago, IL, USA), pAAV-Rep/Cap (serotype8) and pAd helper. Following cell lysis, the samples were purified by affinity chromatography and separated by CsCl density gradient ultracentrifugation (DG-UC). The lower-density band was collected as empty particles, and the higher-density band was collected as full particles. Both full and empty particles were formulated in 1× PBS (Gibco [Thermo Fisher Scientific], Grand Island, NY, USA), 200 mM sodium citrate and 0.001% poloxamer-188 (BASF, Ludwigshafen, Germany) at pH 7.4 and were concentrated to 1.0 mg/mL using Amicon Ultra Centrifugal Filters, 30 kDa MWCO (Sigma-Aldrich, St. Louis, MO, USA).

### 2.2. Capillary Gel Electrophoresis (CGE) of ssDNA

rAAV8 full and empty samples at 46 μg/mL (7.4 × 10^12^ capsid/mL), as quantified by the Pierce BCA assay (Thermo Fisher Scientific, Waltham, MA, USA), were digested by benzonase at 37 °C for 30 min to remove the contaminant-free DNA in the original samples. After the benzonase was quenched by adding 500 mM EDTA to a final concentration of 50 mM, the rAAV8 capsids were digested by proteinase K at 55 °C for 60 min to release the encapsidated DNA. Proteinase K was denatured by heating at 95 °C for 20 min. The QIAquick PCR Purification Kit (QIAGEN, Hilden, Germany) was used to purify the extracted DNA from rAAV8 capsids, and DNA was eluted using nuclease-free water. Samples were analyzed on a PA800Plus (SCIEX, Framingham, MA, USA) using a laser-induced fluorescence (LIF) detector with a 520 nm (excitation 488 nm) emission filter.

### 2.3. Capillary Gel Electrophoresis (CGE) for the Capsid Protein

CGE measurements of the capsid protein were carried out primarily in accordance with previously reported protocols [40]. rAAV samples (1.5 μg) were denatured at 60 °C for 3 min and buffer-exchanged using an Amicon Ultra Centrifugal Filter, 30 kDa MWCO (Sigma-Aldrich). The collected samples were diluted up to 80 μL with deionized water. CGE measurements were performed using PA800Plus (SCIEX) with UV detection at 214 nm. The VP ratio was calculated by the peak area, using the molar absorption coefficient at 214 nm estimated from the amino acids of each VP [3,41]. VP3_total_ was defined as the sum of the molar ratios of VP3 and VP3_clip_.

### 2.4. Characterization of Full and Empty Particles

Fifteen microliters of rAAV8 full sample (0.4 mg/mL) and a mixture of five microliters of 200 mM ammonium acetate solution (Sigma-Aldrich) adjusted at pH 7.4 and 10 μL of rAAV8 empty sample (0.1 mg/mL) were buffer-exchanged into 200 mM ammonium acetate, pH 7.4, using Micro Bio-Spin P-6 gel columns (Bio-Rad, Hercules, CA, USA). Five-microliter samples were loaded into in-house-prepared gold glass capillaries for nano-electrospray ionization. Mass analysis was conducted in Direct Mass Technology (DMT) mode on a Thermo Scientific Q Exactive UHMR (Thermo Fisher Scientific) with an ion transfer target and detector optimization of high *m*/*z*, a spray voltage of 1.2 kV and desolvation voltage of −15 V. Trapping gas pressure readouts were in the range of 2.19–2.37 × 10^−11^ mbar. Data were acquired in triplicate at 50 K @ *m*/*z* 400 resolution with an *m*/*z* range of 15,000–40,000 for 20 min. Data were processed by STORIBoard software ver. 1.0 (Proteinaceous, Chicago, IL, USA) and analyzed by an in-house Python 3 script to obtain mass values and the full-to-empty ratio.

### 2.5. HDX-MS Measurements

For HDX-MS analysis, the rAAV8 sample concentration was adjusted to 1.0 mg/mL in the rAAV8 and control samples. The adjusted rAAV8 sample was diluted 10-fold with H_2_O or D_2_O buffer. The H_2_O buffer was prepared by 10-fold dilution of 10× PBS, pH 7.4, with pure water, and the pH was adjusted to 7.4 using HCl (FUJIFILM Wako Pure Chemicals, Osaka, Japan). The D_2_O buffer was prepared by dissolving dried 10× PBS and 200 mM NaCl with 10 volumes of D_2_O (Iwatani, Osaka, Japan), and the pD value was adjusted to 7.4 using DCl (FUJIFILM Wako Pure Chemicals). Four microliters of 1.0 mg/mL rAAV samples were labeled with 36 μL of D_2_O buffer for different time points (15, 30, 180, 600, and 3600 s) at 4 and 20 °C. HDX reactions were quenched soon after labeling by mixing 65 μL quench buffer with 39 μL of the labeled sample at pH 2.5 and 0 °C. The composition of the quench buffer was 8 M guanidine hydrochloride (Gdn-HCl) (FUJIFILM Wako Pure Chemicals), 8 mM tris(2-carboxyethyl)phosphine (TCEP) (Nacalai Tesque, Inc., Kyoto, Japan), and 200 mM NaH_2_PO_4_ (FUJIFILM Wako Pure Chemicals). The samples were adjusted to pH 2.5 after mixing the two solutions. Quenched samples were diluted with 135 μL of quench diluent to reduce the guanidine hydrochloride (Gdn-HCl) concentration to 2 M, at which level pepsin is not denatured [42]. The quench diluent composition was formic acid [Tokyo Chemical Industry (TCI) Co. Ltd., Tokyo, Japan] in pure water adjusted to pH 2.5 after mixing 39 μL of H_2_O buffer, 65 μL of quench buffer, and 135 μL of quench diluent. A total of 250 μL of the quench-diluted sample was automatically injected into the HDX system. Labeling and quenching were performed manually, and the quench dilution and injection were performed by the HDX PAL system (Trajan, Morrisville, NC, USA) and Chronos software ver. 5.1.8 (Trajan).

Online digestion was performed using a pepsin column (Enzymate Protein Pepsin Column, 300 Å, 5 µm, 2.1 × 30 mm; Waters, Milford, MA, USA) for 4 min. Peptides digested by pepsin were separated by trap and analytical columns at 2 °C. The mobile phase for the digestion was MS-grade water at pH 2.5, adjusted using formic acid. Digested peptides were desalted by the trap column (ZORBAX Eclipse XDB-C8, 2.1 × 20 mm, 1.8-micron, 600 bar; Agilent, Santa Clara, CA, USA) and separated by the analytical column (Hypersil GOLD^TM^, 1 × 50 mm, 1.9 μm particle size; Thermo Fisher Scientific) at 2 °C. The mobile phase for separation was mobile phase A (0.1% formic acid in MS-grade water; Kanto Chemical) and mobile phase B (0.1% formic acid in acetonitrile; Kanto Chemical). Separation was performed with a step linear gradient. In the first step, %B increased from 8% to 15% for 1 min, and in the second step, %B increased from 15% to 40% for 9 min. Ultimate 3000 (Thermo Fisher Scientific) LC pumps were used for digestion, desalting, and separation. A Q Exactive HF-X mass spectrometer (Thermo Fisher Scientific) was used for MS measurements with the following settings: capillary temperature, 275 °C; resolution, 120,000; and *m*/*z* window range, 260–2000. For data-dependent MS/MS analysis for peptide mapping analysis using unlabeled samples, the maximum injection time was optimized to 250 ms to improve the number of identified peptides and peptide spectrum matches (PSMs).

### 2.6. HDX-MS Data Analysis

Peptide mapping analysis with non-deuterated samples was performed using Proteome Discoverer^TM^ ver. 2.4 (Thermo Fisher Scientific) with a precursor mass tolerance of 10 ppm and a fragment mass tolerance of 0.02 Da. N-terminal Met excision and acetylation, oxidation, and phosphorylation were selected as dynamic post-translational modifications [3,43]. HDX analysis was performed by HDExaminer ver. 3.2.1 (Sierra Analytics, Modesto, CA, USA). Statistical comparisons were performed by Woods plot with the accumulated significance test [44,45]. Vertical significance limits of ΔHX were 2.15 and 2.73 Da at labeling temperatures of 4 and 20 °C, respectively, for the comparison between rAAV8 full capsids and rAAV8 empty capsids. Structural mapping of HDX-MS results was performed by PyMOL ver. 2.5.0 (Schrödinger, New York, NY, USA). The VP3 structures of rAAV8, PDB ID: 6PWA for full and 6U20 for empty (aa: 220–738), were used as the template [46]. A structural model for the structurally unsolved VP1u and VP1/VP2 common regions (aa: 1–219) was generated by Alphafold2 [47]. Data analysis and data presentation followed the recommended guidelines [48].

## 3. Results and Discussion

### 3.1. rAAV Sample Characterization

The protein sample state was carefully monitored before HDX-MS analysis. We first characterized the rAAV8 samples using CGE with a UV detector for the rAAV8 capsid proteins and the LIF detector for the rAAV8 encapsidated genome. Three major peaks corresponding to VP3, VP2, and VP1 were observed for full and empty samples, with a minor peak representing the VP3 clip [3] (Figure S1A). The average ratios of VP1:VP2:VP3_total_ were 4.7:5.2:50.2 for full samples and 4.4:6.1:49.6 for empty samples. No peaks derived from protein contaminants other than rAAV8 particles were observed, indicating that the purity of the samples was sufficiently high for HDX-MS analysis. For ssDNA, one peak of the encapsidated genome was observed only for the full samples. No peak was observed for the empty samples, confirming that the rAAV8 empty samples did not contain particles encapsidating any nucleic acids (Figure S1B).

**Figure 1:**
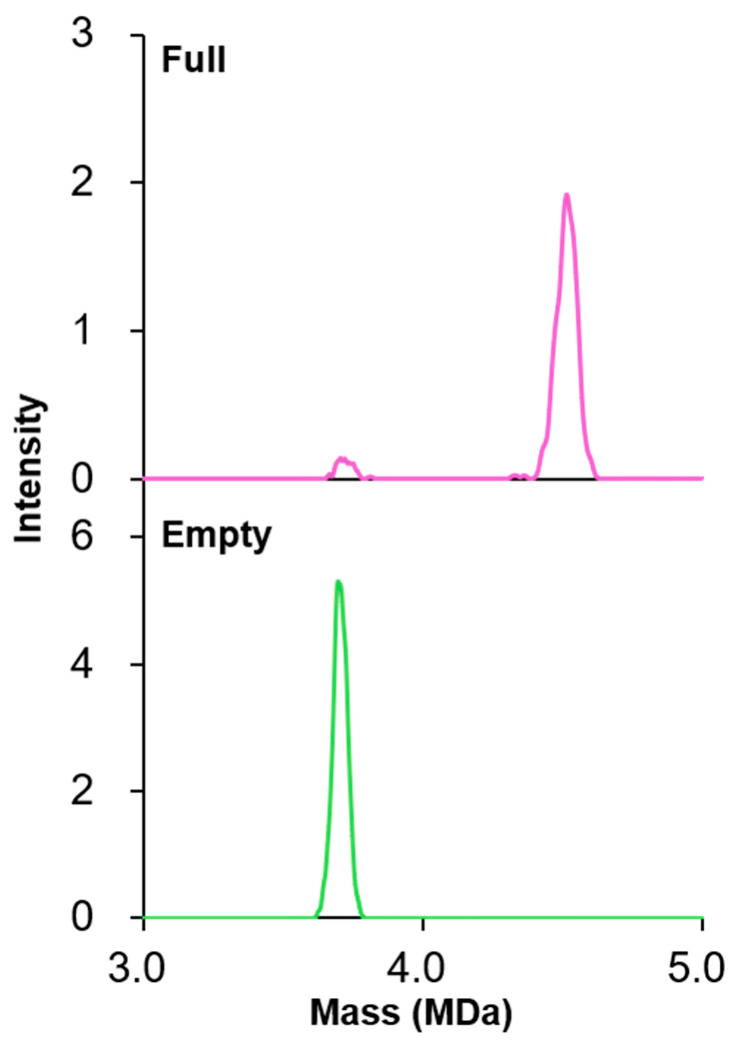
Characterization of rAAV8 full (purple; **upper**) and empty (green; **lower** panels) samples by DMT mode analysis.

DMT analysis was conducted to confirm that the rAAV8 full sample contained predominantly rAAV8 full particles, and the rAAV8 empty sample contained primarily rAAV8 empty particles (Figure 1). Two peaks at 4.51 ± 0.00 MDa and 3.72 ± 0.01 MDa were observed for the rAAV8 full sample, and one peak at 3.71 ± 0.00 MDa was observed for the rAAV8 empty sample. The calculated average masses of rAAV8 full and empty particles are 4.51 MDa and 3.73 MDa, respectively, when VP1:VP2:VP3 = 5:5:50 with or without 2521 bases of ssDNA. Thus, the observed peak at 4.51 ± 0.00 MDa corresponded to full particles, and the peaks at 3.72 ± 0.01 MDa and 3.71 ± 0.00 MDa corresponded to empty particles. The results from calculating the full-to-empty ratio using the peak areas showed that 94.3 ± 1.5% full particles contained the rAAV8 full sample, whereas the empty sample did not contain any full particles. Thus, we concluded that our prepared rAAV8 full and empty samples reflected the characteristics of full and empty particles, so we used them for subsequent HDX-MS analysis.

### 3.2. Dynamics of the rAAV8 Capsid

HDX-MS was used to evaluate the dynamics of rAAV8 full and empty capsids. This evaluation detected 486 peptides with 85% sequence coverage for the entire rAAV8 at a labeling temperature of 4 °C. At a labeling temperature of 20 °C, 393 peptides were detected with 79% coverage for the entire rAAV8. We attached the uptake plot for each peptide in the supplemental files (Figures S5–S7). The VP1/VP2 common region could not be covered by HDX-MS since this region is rich in lysine, arginine, and proline, after which pepsin cannot cleave [49]. Dual-protease digestion, such as with protease type XIII/pepsin, would be helpful to improve sequence coverage [50,51].

**Figure 2:**
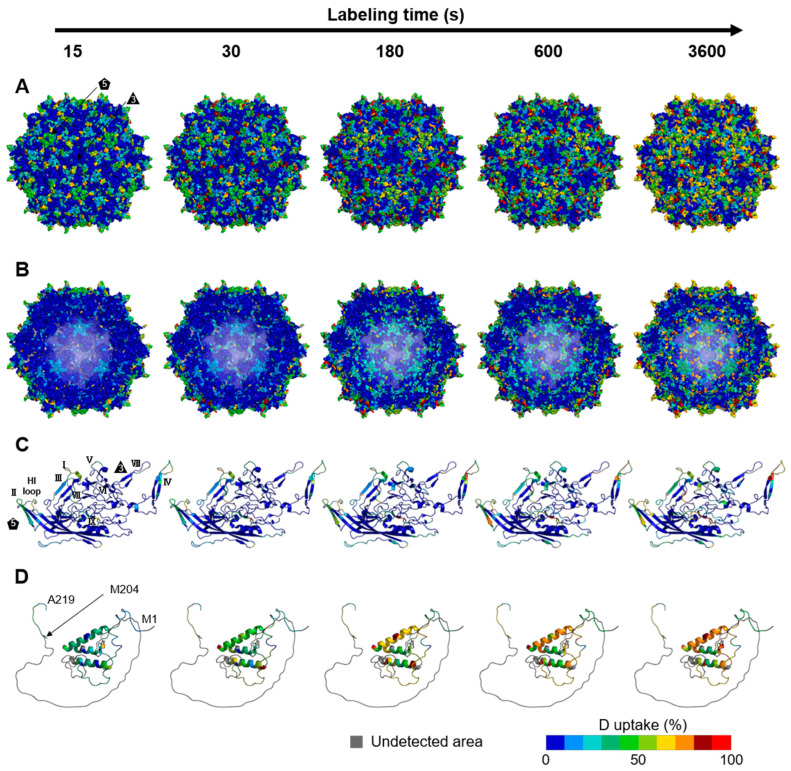
Structural dynamics of rAAV8 full capsids at a labeling temperature of 4 °C over the labeling time course. The structural dynamics of the rAAV8 full capsid mapped onto the assembled full capsid (PBD ID: 6PWA) external surface (**A**) and internal surface, which is a cross-sectional view of a capsid (**B**), VP3 monomer (**C**), and model of the structurally unsolved region (**D**). The VP3 monomers are enlarged views of a VP3 taken from a capsid composed of 60 VPs. The predicted model of the structurally unsolved region is magnified. The characteristic structures of rAAV capsids, such as 3-fold and 5-fold symmetry axes and VRs, are shown. In the predicted model of the structurally unsolved region, M204 (N-terminus of VP3) and, the N- and C-terminus of the region are shown. The regions where the peptides were not detected are colored gray.

**Figure 3:**
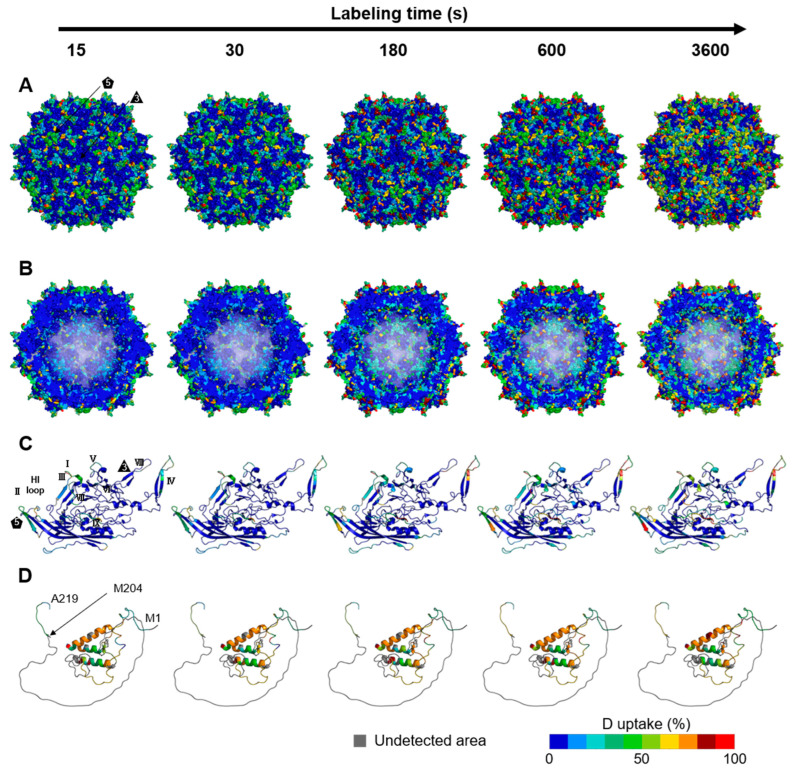
Structural dynamics of rAAV8 empty capsids at a labeling temperature of 4 °C over the labeling time course. The structural dynamics of the rAAV8 empty capsid mapped onto the assembled empty capsid (PDB ID: 6U20) external surface (**A**) and internal surface, which is a cross-sectional view of a capsid (**B**), VP3 monomer (**C**), and model of the structurally unsolved region (**D**). The VP3 monomers are enlarged views of a VP3 taken from a capsid composed of 60 VPs. The predicted model of the structurally unsolved region is magnified. The characteristic structures of rAAV capsids, such as 3-fold and 5-fold symmetry axes and VRs, are shown. In the predicted model of the structurally unsolved region, M204 (N-terminus of VP3) and, the N- and C-terminus of the region are shown. The regions where the peptides were not detected are colored gray.

The deuterium uptake ratios at 4 °C and five time points (15, 30, 180, 600, and 3600 s) were mapped onto the three-dimensional structures to investigate the rAAV8 full and empty capsid structural dynamics (Figure 2 and Figure 3). Additionally, we mapped the results of HDX-MS onto the three-dimensional structures at a 20 °C labeling temperature (Figures S2 and S3). The cryo-EM structure of the rAAV8 full capsid (PDB ID: 6PWA) and empty capsid (PDB ID: 6U20) were used for structural mapping of the entire capsid and the VP3 monomer (aa: D220–L738) (Figure 2A,B and Figure 3A,B) [46]. A structural model of the structurally unsolved region corresponding to M1–A219, including the VP1u and VP1/VP2 common regions, was produced by AlphaFold2 (Figure 2D and Figure 3D).

A time-dependent increase in deuterium uptake was observed on the outer surface of the capsid, such as at S705–D714 (Figure S4A). An observed increase in deuterium uptake in regions such as L406–T417 on the inner surface of the capsid over the time course indicates penetration of heavy water into the capsid (Figure S4B). This observation can be attributed to the size of heavy water molecules with a diameter of 1.4 Å, which is smaller than the pores with a diameter of approximately 12 Å that are located along the 5-fold symmetry axis of the capsid [21]. Consequently, the water molecules within the capsid are replaced with heavy water, which enables the evaluation of the dynamics of the entire rAAV capsid by HDX-MS.

As shown in Figure 2A and Figure 3A, increases in deuterium uptake during the time course were observed around the protrusions surrounding the 3-fold symmetry axis, the channel structure of the 5-fold symmetry axis, and all VRs that form loop structures on the outer surface of the capsid, indicating that these regions have flexible structures. These characteristic structural topologies at the 3-fold and 5-fold symmetry axes and VRs contribute to rAAV infection, such as via receptor binding, tissue transduction, and the pathway of externalization of the VP1u and VP1/VP2 common regions [10,52,53]. The dynamic structures of these regions may play a crucial role in rAAV infection. Moreover, the highly dynamic behavior observed in the protrusions and the channels is consistent with the results of HDX-MS for MVM, another parvovirus, indicating that the high flexibility of the 3-fold and 5-fold symmetry axis regions is conserved among members of the Parvoviridae family [35].

Conversely, deuterium uptake in regions with secondary structures, such as the α-helices and jelly roll folds, was less than 10% and remained essentially unchanged over the labeling time. The structure and sequence of the α-helix and jelly roll fold in the common VP3 region of rAAV capsids are highly conserved among most serotypes [54].

Interestingly, the deuterium uptake of the VP1u region in the structurally unsolved region reached a plateau even at the shortest labeling time of 15 s in empty capsids, suggesting that this region of empty capsids may not be folded as it exhibits an exchange level equivalent to or higher than that of the loop structures located on the capsid surface (Figure 3D). In contrast, the VP1u region of the full capsid showed a time-dependent increase in deuterium uptake of 20–60%, indicating that VP1u in the full capsid has a different structure from that in the empty capsid; namely, the VP1u in the full capsid may adopt a folding state in a manner influenced by the surrounding DNA. Although the AlphaFold2-predicted structure of this region for residues 67–129 contained α-helical structures, as indicated by the lower deuterium uptake in the α-helix of VP3 even at the longest labeling time of 3600 s, the VP1u region of the full capsid is considered to have α-helical structures with a fluctuating nature. This interpretation could be explained by the following HDX reaction mechanisms [55,56].

A protein in a solution state continuously interconverts between open and closed conformations both locally and globally [57]. The momentary disruption of internal hydrogen bonds in the folded proteins allows the formation of hydrogen bonds with the solvent and the replacement of the amide hydrogen with deuterium. Thus, the HDX of amides is significantly influenced by the level of protein folding. The HDX reaction mechanisms considering the localized protein fluctuation behavior can be described by Equation (1) [58]:

(1) $$cl(H)\begin{array}{c} \begin{array}{c} k_{op}\\ ⇌ \end{array}\\ k_{cl} \end{array}\;op(H)\begin{array}{c} \begin{array}{c} k_{ch}\\ \to \end{array}\\ D2O \end{array}\;op(D)\begin{array}{c} \begin{array}{c} k_{op}\\ ⇌ \end{array}\\ k_{cl} \end{array}cl(D)$$

where cl refers to the folded and exchange-incompetent states of the protein, op refers to the solvent-exposed and exchange-competent states of the protein, *k*_op_ and *k*_cl_ are the rate constants of the opening and closing reactions, and *k*_ch_ is the intrinsic exchange rate, which is a site-specific amide HDX rate in an unstructured peptide. The HDX reaction rate constant (*k*_HDX_) of proteins is highly affected by structural unfolding and refolding. The α-helix structures in the VP1u region may have relatively large *k*_op_ values, reflecting structural fluctuations of this region. From Equation (1), the HDX reaction rate constant (*k*_HDX_) of proteins can be described by Equation (2) [58,59]:

(2) $$k_{HDX}=\frac{k_{op}\times k_{ch}}{k_{op}+k_{cl}+k_{ch}}$$

As shown in Equation (2), *k*_HDX_ is affected by the structural unfolding and refolding (*k*_op_ and *k*_cl_) and the intrinsic exchange rate (*k*_ch_). Considering that the native state of most proteins under physiological conditions is quite stable and the refolding rate is much higher than the intrinsic exchange rate (*k*_cl_ ≫ *k*_ch_), *k*_HDX_ is highly affected by *k*_op_ and *k*_cl_. The α-helix structures in the VP1u region exhibited rapid deuterium exchange, in other words, higher *k*_HDX_ values, indicating that this region may have relatively large *k*_op_ values and exhibit structural fluctuations.

In previous reports, it is proposed that VP1u and VP1/VP2 common regions are externalized through the 5-fold symmetry axis channel with a diameter of only 12 Å [21]. Notably, another study showed that the externalization is not necessarily dependent on endosomal acidification [60]. The fluctuating nature of the VP1u region as demonstrated by the HDX-MS at pH 7.4 supports these previous findings.

### 3.3. Structural Differences between Full and Empty Capsids

**Figure 4:**
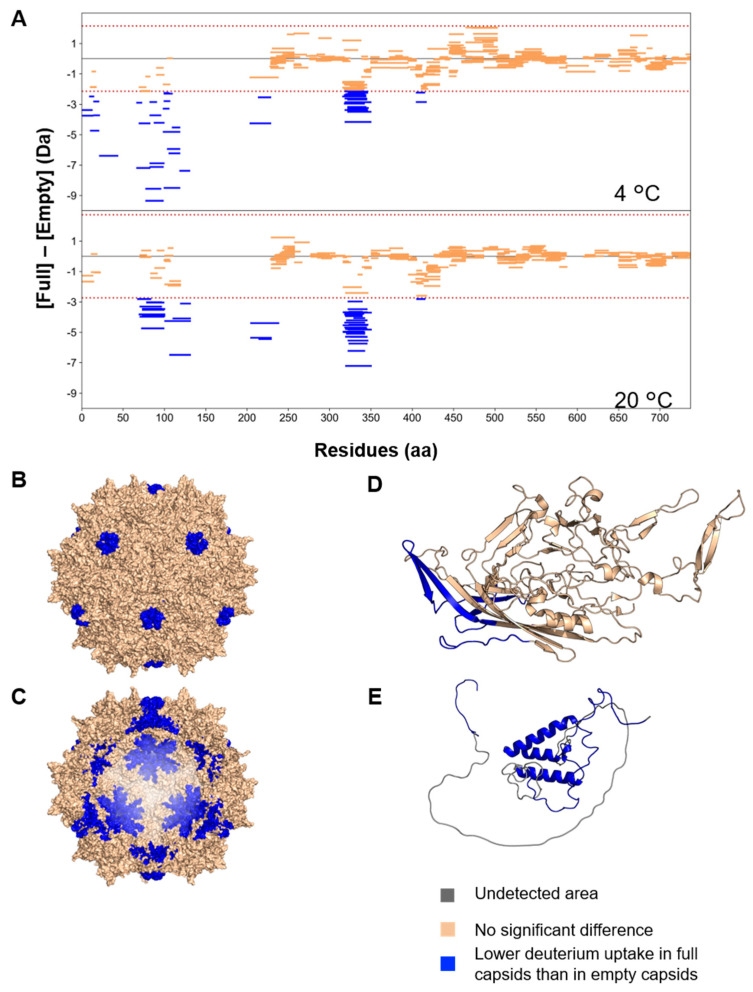
Structural comparison between rAAV8 full and empty capsids by differential HDX-MS analysis. (**A**) Woods plots illustrating the variance in deuterium uptake for each peptide under 4 °C (upper panel) and 20 °C (lower panel) labeling conditions. Each bar represents a peptide corresponding to a particular fragment. The brown dotted line represents the vertical significance limits of ΔHX of 2.15 (4 °C) and 2.73 Da (20 °C). The blue peptides show significant differences between full and empty capsids. (**B**–**E**) Structural differences were mapped onto the three-dimensional structure. The regions colored in blue indicate a difference in deuterium uptake of less than 2.5 Da, which was observed in rAAV8 full particles compared with that of empty particles. Regions where peptides were not detected are colored gray.

The deuterium uptake of rAAV8 full and empty capsids was compared to investigate the impact of the encapsidated genome on the capsid. The difference in the deuterium uptake between full and empty capsids ([Full]–[Empty]) for each peptide is illustrated as Woods plots at labeling temperatures of 4 and 20 °C (Figure 4A). Notably, all peptides with significant differences between full and empty capsids showed lower deuterium uptake in the full capsid, indicating lower structural flexibility and/or interactions with the genome in the full capsid. According to several previous comparative studies of rAAV full and empty capsids, the RMSD of C_α_–C_α_ distance between full and empty capsids is only approximately 0.3 Å, with no significant differences observed [54,61,62]. Moreover, our HDX-MS analysis detected differences in the structural dynamics in a solution state between full and empty capsids, highlighting the advantage of HDX-MS.

At labeling temperatures of both 4 and 20 °C, significant differences in deuterium uptake were observed for A68–F100 and E102–L131 in the VP1u region, and A206–W229, L318–E350, and R407–T417 in the VP3 common region. Significant differences were only identified by labeling at 4 °C for A2–G44, Q101 located in the VP1u region and Y416–T417. In contrast, significant differences in deuterium uptake were additionally captured by labeling at 20 °C in H230–D238, which are located in the VP3 common region. In the VP1u region, which displays higher dynamics, more peptides showed a significant difference in deuterium uptake between full and empty capsids at 4 °C than at 20 °C. Conversely, differences in the VP3 common region were slightly more noticeable at 20 °C than under the 4 °C labeling conditions. This is because the deuterium exchange rate is suppressed by 83.6% at 4 °C compared with that at 20 °C [38], which enables unambiguous detection of differences in deuterium uptake in highly flexible regions. HDX-MS data at 4 and 20 °C were then integrated and used for further discussion.

We next mapped regions where differences occurred in the HDX-MS results onto the three-dimensional structure of the rAAV8 capsid and predicted the structure of the structurally unsolved region (Figure 4B–E). In terms of the VP3 common region, the region demonstrating significant differences in deuterium uptake between full and empty particles was located around the 5-fold symmetry axis (Figure 4B–D). Extending density beneath the 5-fold channel was previously observed in full capsids but not in empty capsids for rAAV8 and AAVrh39 [61]. Our HDX-MS results also showed the impact of the encapsidated DNA on the 5-fold symmetry axis region, which is consistent with previous reports.

Remarkably, a significant decrease in HDX was observed in the VP1u region of rAAV8 full capsids compared with that of the empty capsids (Figure 3D). Although, to the best of our knowledge, no information has previously been published regarding the impact of encapsidated DNA on this region, the observed reduction in deuterium uptake for the full capsid in this study may have arisen from the folded structure of VP1u in the full capsid adopted under the influence of surrounding DNA, as described above. Since no significant differences in deuterium uptake between full and empty capsids were observed after 180 s of labeling, VP1u may not have a specific binding site and interact with encapsidated DNA with weak affinity. In proteins with weakly binding states, significant differences in deuterium uptake between binding and lack of binding can be observed with short labeling times. However, as the labeling time increases, the duration of being in the unbound state becomes sufficiently long, so that differences in deuterium exchange between two states are no longer apparent [63,64]. Thus, the interaction between the VP1u region and encapsidated DNA is inferred to be relatively weak, and an unbound state capable of undergoing the HDX reaction is readily adopted. This finding highlighted that the VP1u region is located inside of the capsid and VP1u may be kept in a folded state by the interaction with surrounding DNA.

### 3.4. Structural Differences near the VP3 Transcriptional Start Site between VP1/VP2 and VP3

**Figure 5:**
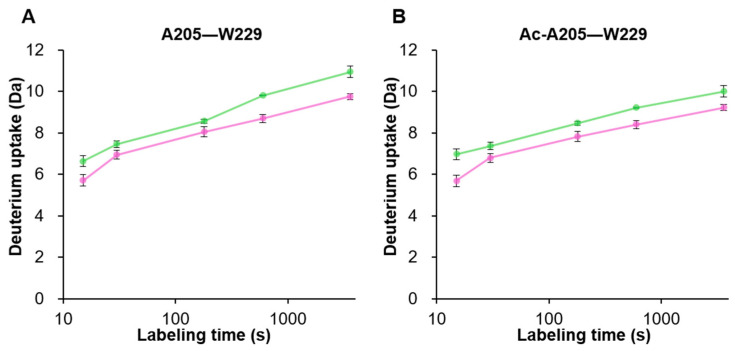
The transition of deuterium uptake at a 4 °C labeling temperature by the A205–W229 peptides derived from VP1/VP2 and VP3. The uptake plots illustrate the deuterium uptake at each labeling time point of rAAV8 full (in purple) and empty (in green). (**A**) Uptake plot of the A205 acetylated peptides derived from VP3. (**B**) Uptake plot of the A205 non-acetylated peptides derived from VP1/VP2.

In the MS analysis, most peptides derived from the VP3 common region (aa: M204–L738) cannot be distinguished by their respective origins from VP1, VP2, or VP3. However, VP3 N-terminal peptides were identified based on the acetylation state of A205 [65]. This identification is because pepsin exhibits a high digestion efficiency of 38% between methionine and alanine residues, and peptides starting from non-acetylated A205 that originate from VP1/VP2 can be generated [49]. We compared deuterium uptake transitions at a 4 °C labeling temperature to explore differences in the deuterium uptake between VP1/VP2-derived and VP3-derived peptides starting from A205 (Figure 5). The summational ΔHX values between acetylated and non-acetylated peptides were 1.23 and 1.38 Da for full and empty, respectively. The fact that the values of these peptides were lower than the ΔHX criterion of 2.15 Da indicated that the deuterium uptake transition in the A205–W229 region of VP1/VP2-derived and VP3-derived peptides remained unchanged despite capturing structural differences between full and empty particles on both peptides. This result reveals that the structurally unsolved transcription start regions of VP3 in VP1/VP2 and VP3 have similar structural dynamics.

## 4. Conclusions

To the best of our knowledge, this is the first study to access structural information of the entire rAAV8 capsid, including the VP1u and VP1/VP2 common regions. The structural dynamics observed in this study for rAAV8 represent its behavior under physiological conditions in solution. Moreover, comparative analysis between rAAV8 full and empty capsids identified local structural differences between these two capsid states. Such findings have not been made by cryo-EM or X-ray crystallography, a method for structural analysis of rAAVs, highlighting the advantages of using HDX-MS to monitor the structural dynamics of the rAAV8 capsid. Thus, the use of HDX-MS in combination with cryo-EM and X-ray crystallography makes it possible to monitor rAAV structural integrity [66]. Future applications should be investigated to facilitate the development of rAAV-based gene therapies.

## Data Availability

The data of this study are available from the corresponding author upon reasonable request.

## Supplementary Materials

The following supporting information can be downloaded at: https://www.mdpi.com/article/10.3390/v16040585/s1, Figure S1. rAAV8 sample characterization by capillary gel electrophoresis (CGE) with UV and LIF detector; Figure S2. Structural dynamics of rAAV8 full capsids at a labeling temperature of 20 °C over the labeling time course; Figure S3. Structural dynamics of rAAV8 empty capsids at a labeling temperature of 20 °C over the labeling time course; Figure S4. Uptake plots of peptides derived from outer and inner surface of capsids; Figure S5. Deuterium uptake plots of all peptides without any post translational modification except VP1 N-terminal peptides at a labeling temperature of 4 °C; Figure S6. Deuterium uptake plots of all peptides with N-terminal Met excision and acetylation of VP3 N-terminal peptides at a labeling temperature of 4 °C; Figure S7. Deuterium uptake plots of all peptides without any post translational modification except VP1 N-terminal peptides at a labeling temperature of 20 °C.
