# Supplementary material for: Higher-Order Structure of Adeno-Associated Virus Serotype 8 by Hydrogen/Deuterium Exchange Mass Spectrometry
Source: Viruses. 2024 Apr 10;16(4):585. doi: 10.3390/v16040585 (PMC11053801; doi:10.3390/v16040585)
Supplement: Supplementary file 1 [file viruses-16-00585-s001.zip › Supplemental/AAV8-HDXMS-Uptake-plots(S5-S7).pdf]

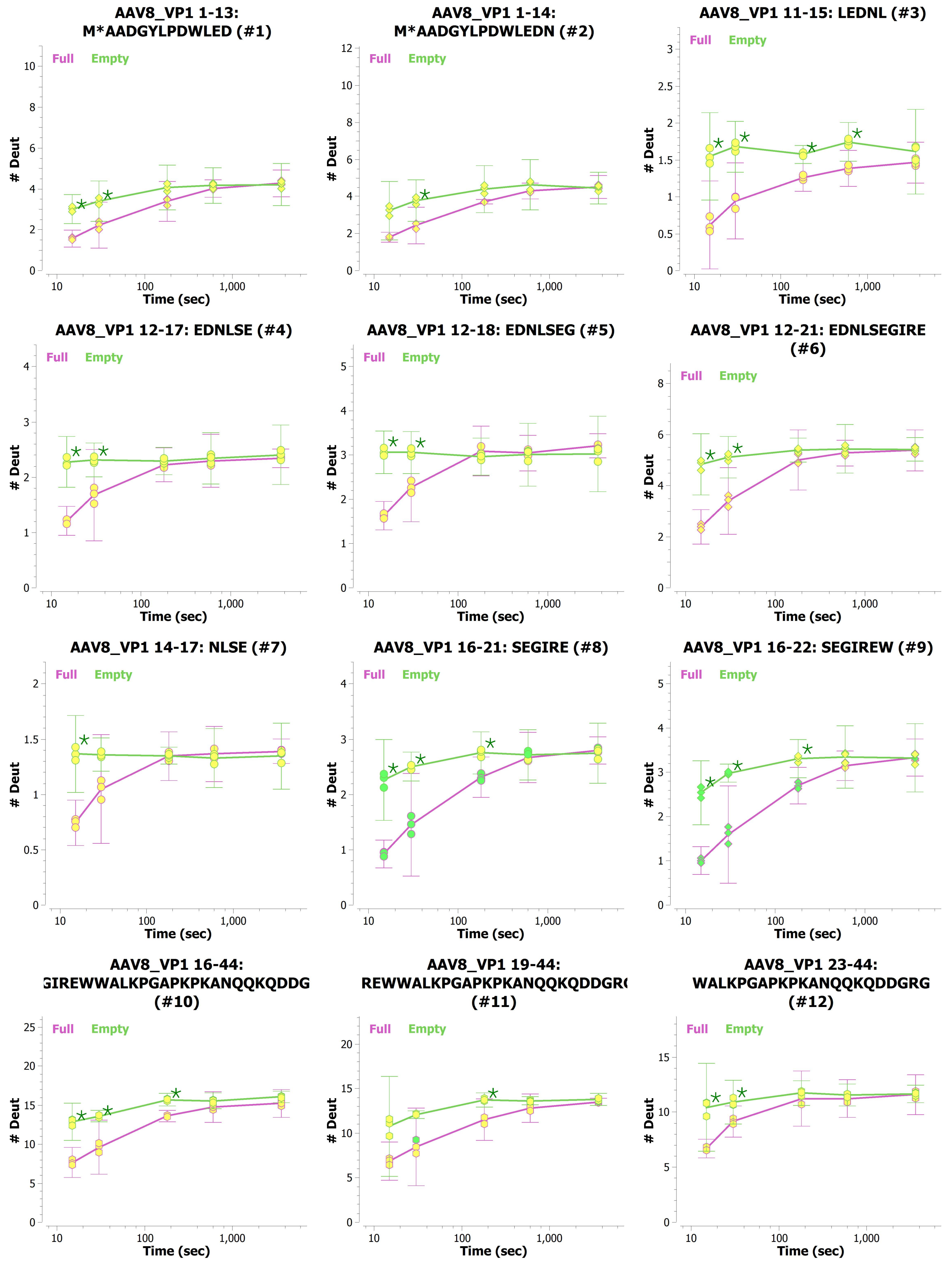
AAV8_VP1 1-13: M*AADGYLPDWLED (#1)
AAV8_VP1 1-14: M*AADGYLPDWLEDN (#2)
AAV8_VP1 11-15: LEDNL (#3)
AAV8_VP1 12-17: EDNLSE (#4)
AAV8_VP1 12-18: EDNLSEG (#5)
AAV8_VP1 12-21: EDNLSEGIRE (#6)
AAV8_VP1 14-17: NLSE (#7)
AAV8_VP1 16-21: SEGIRE (#8)
AAV8_VP1 16-22: SEGIREW (#9)
AAV8_VP1 16-44: GIREWWALKPGAPKPKANQQKQDDG (#10)
AAV8_VP1 19-44: REWWALKPGAPKPKANQQKQDDGR (#11)
AAV8_VP1 23-44: WALKPGAPKPKANQQKQDDGRG (#12)
Full
Empty
Deut
Time (sec)
10
100
1,000

AAV8_VP1 68-73: ADAAAL (#13)

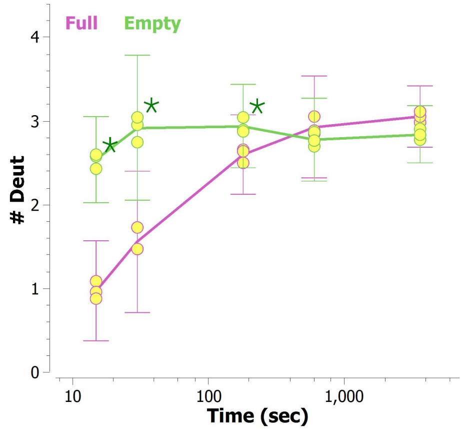

AAV8_VP1 68-83: ADAAALEHDKAYDQQL (#14)

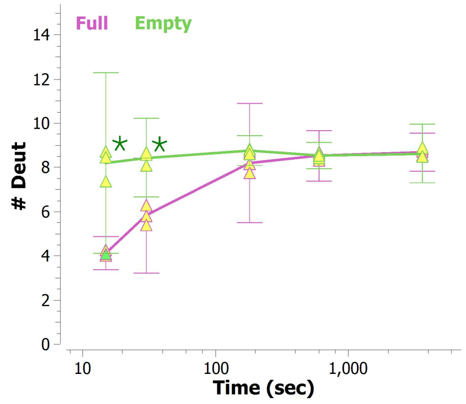

AAV8_VP1 70-100: ALEHDKAYDQQLQAGDNPYLRYNHAD (#15)

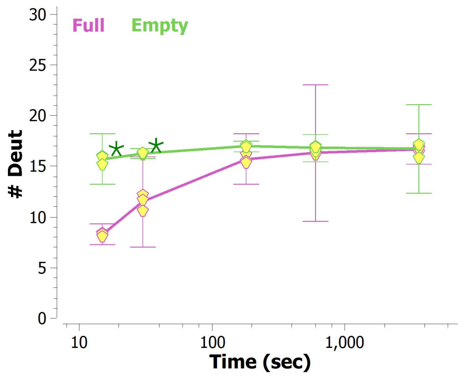

AAV8_VP1 71-78: AALEHDKA (#16)

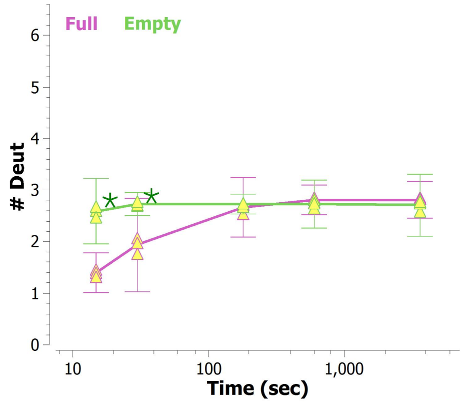

AAV8_VP1 71-83: AALEHDKAYDQQL (#17)

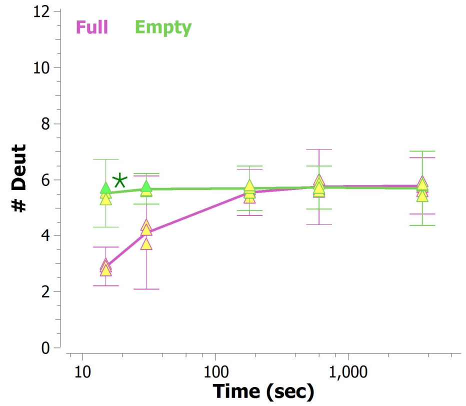

AAV8_VP1 71-96: AALEHDKAYDQQLQAGDNPYLRYNHA (#18)

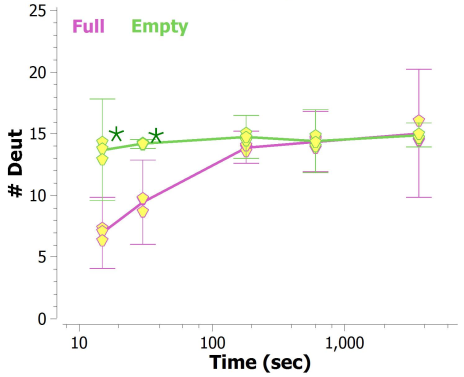

AAV8_VP1 71-99: ALEHDKAYDQQLQAGDNPYLRYNHAD (#19)

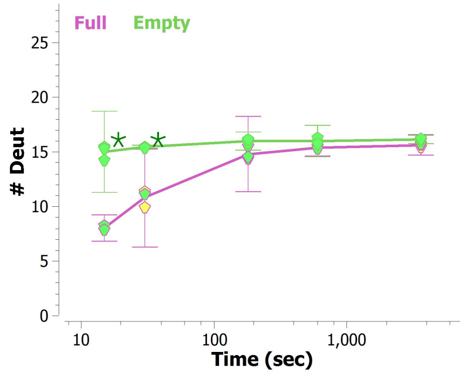

AAV8_VP1 71-100: LEHDKAYDQQLQAGDNPYLRYNHAD (#20)

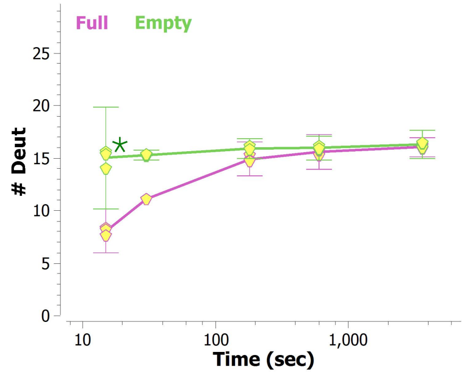

AAV8_VP1 73-83: LEHDKAYDQQL (#21)

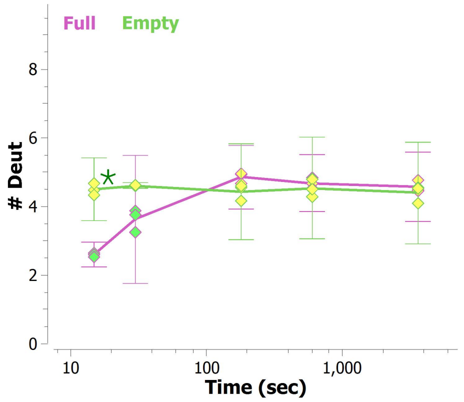

AAV8_VP1 73-96: LEHDKAYDQQLQAGDNPYLRYNHA (#22)

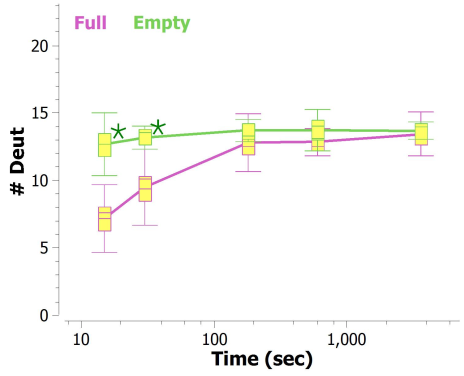

AAV8_VP1 73-99: EHDKAYDQQLQAGDNPYLRYNHADA (#23)

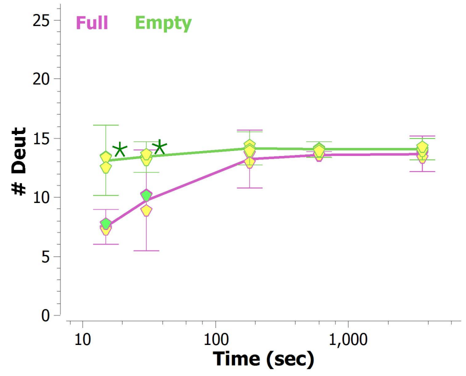

AAV8_VP1 73-100: EHDKAYDQQLQAGDNPYLRYNHADAE (#24)

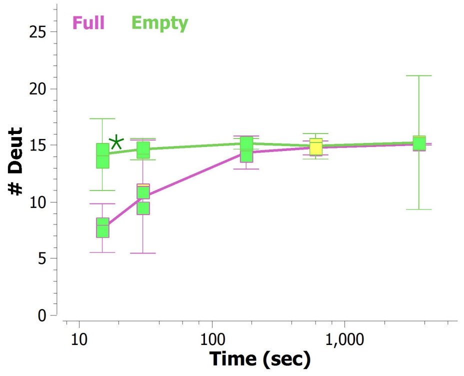

AAV8_VP1 74-83: EHDKAYDQQL (#25)

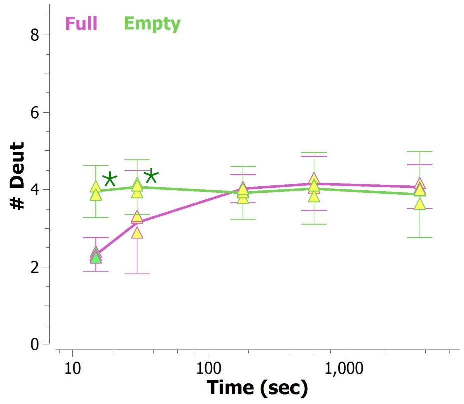

AAV8_VP1 74-91: EHDKAYDQQLQAGDNPYL (#26)

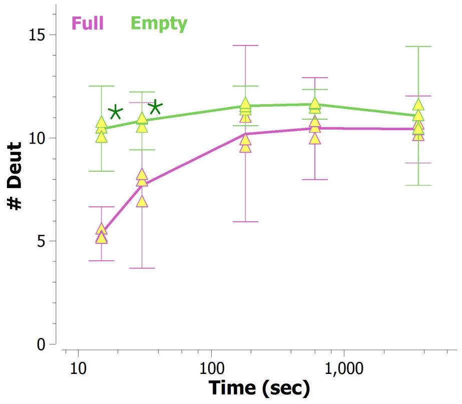

AAV8_VP1 74-96: EHDKAYDQQLQAGDNPYLRYNHA (#27)

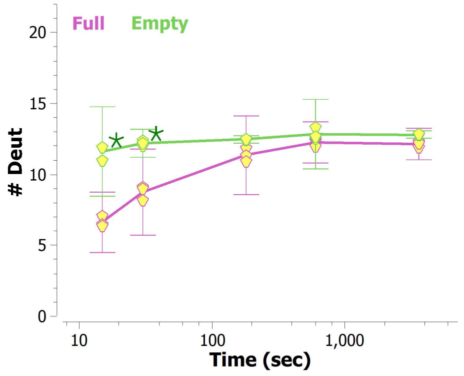

AAV8_VP1 74-99: EHDKAYDQQLQAGDNPYLRYNHADAE (#28)

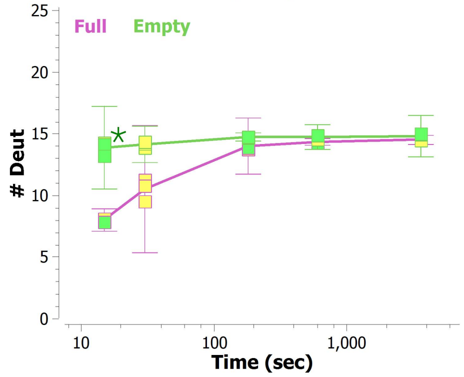

AAV8_VP1 74-100: HDKAYDQQLQAGDNPYLRYNHADAE (#29)

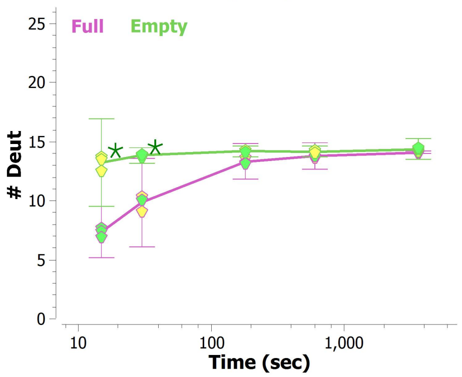

AAV8_VP1 79-83: YDQQL (#30)

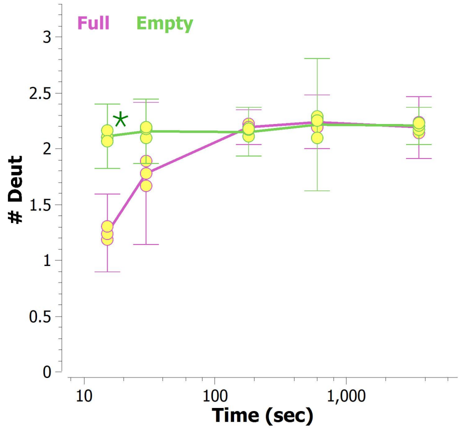

AAV8_VP1 79-96: YDQQLQAGDNPYLRYNHA (#31)

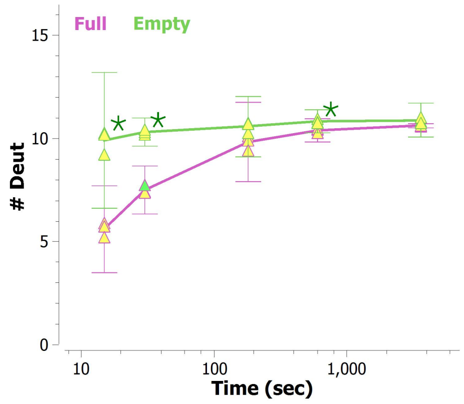

AAV8_VP1 79-99: YDQQLQAGDNPYLRYNHADAE (#32)

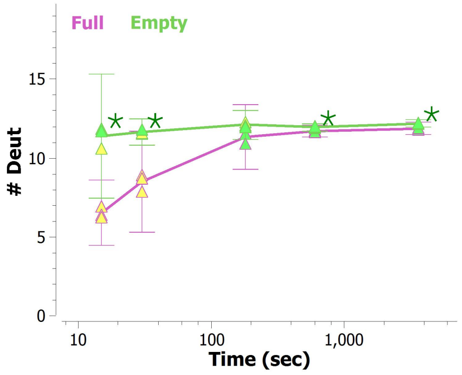

AAV8_VP1 79-100: YDQQLQAGDNPYLRYNHADAEF (#33)

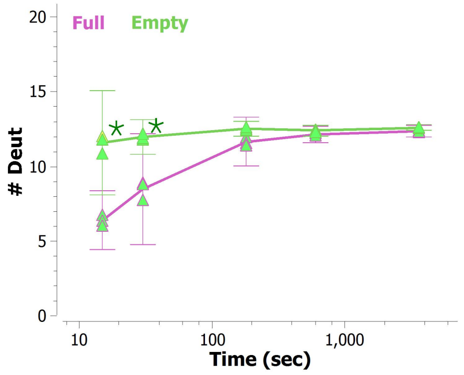

AAV8_VP1 84-91: QAGDNPYL (#34)

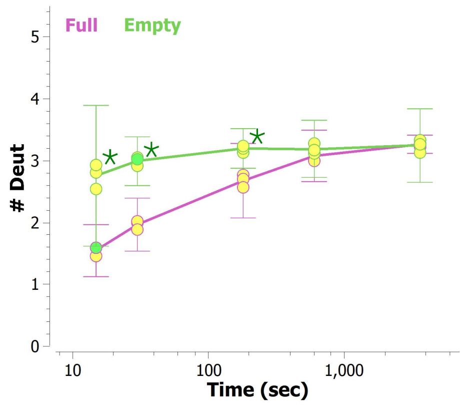

AAV8_VP1 84-96: QAGDNPYLRYNHA (#35)

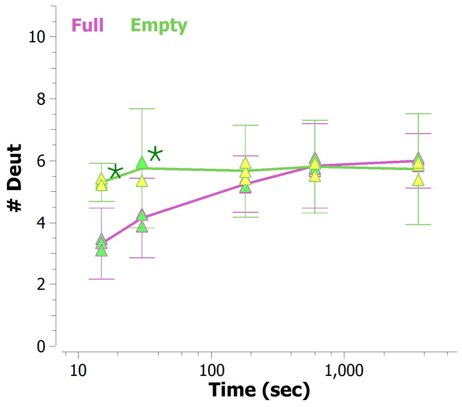

AAV8_VP1 84-99: QAGDNPYLRYNHADAE (#36)

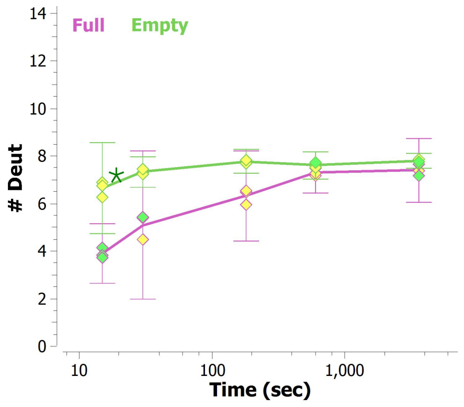

AAV8_VP1 84-100: QAGDNPYLRYNHADAEF (#37)

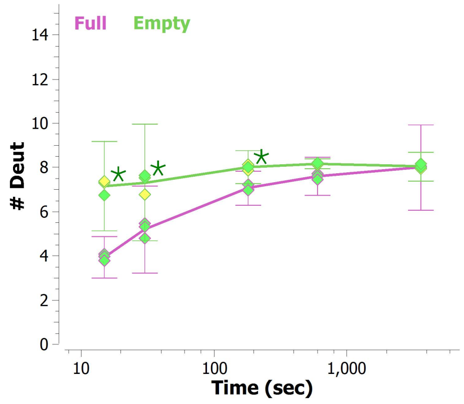

AAV8_VP1 89-100: PYLRYNHADAEF (#38)

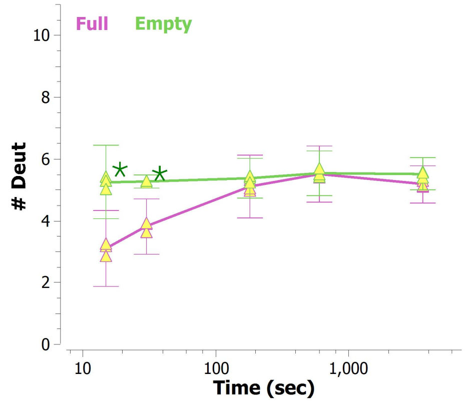

AAV8_VP1 92-99: RYNHADAE (#39)

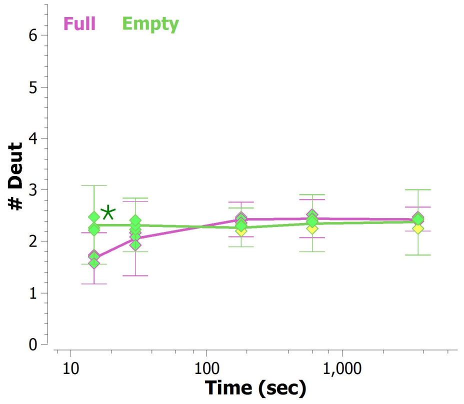

AAV8_VP1 92-100: RYNHADAEF (#40)

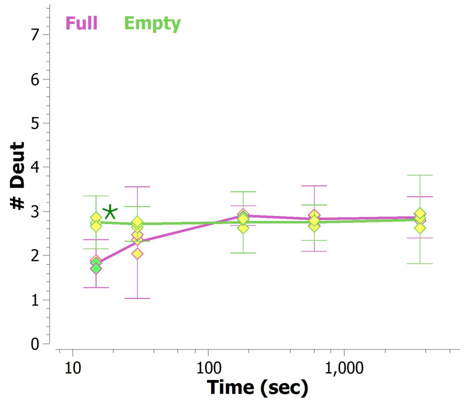

AAV8_VP1 100-106: FQERLQE (#41)

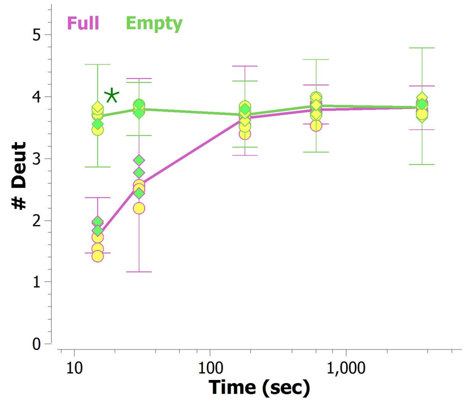

AAV8_VP1 100-107: FQERLQED (#42)

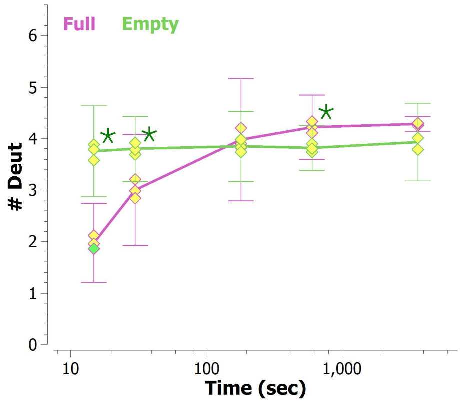

AAV8_VP1 100-119: FQERLQEDTSFGGNLGRAVF (#43)

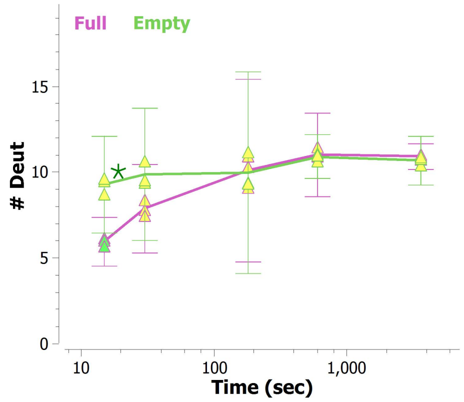

AAV8_VP1 101-106: QERLQE (#44)

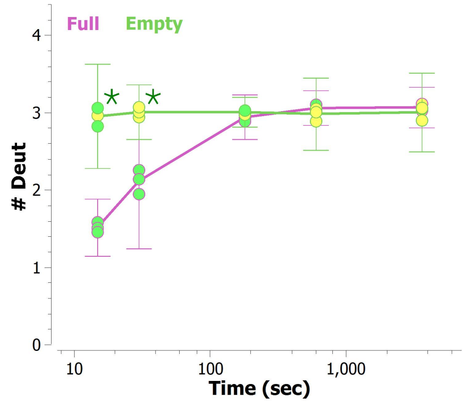

AAV8_VP1 101-107: QERLQED (#45)

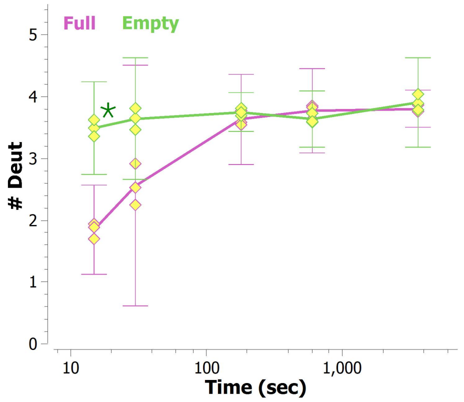

AAV8_VP1 101-110: QERLQEDTSF (#46)

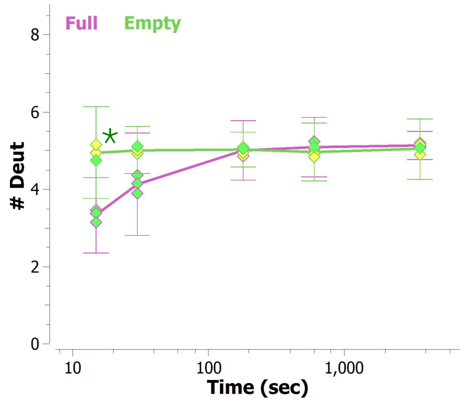

AAV8_VP1 101-119: QERLQEDTSFGGNLGRAVF (#47)

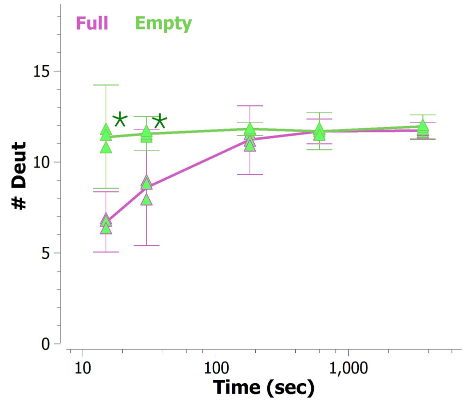

AAV8_VP1 101-131: RLQEDTSFGGNLGRAVFQAKKRVLEPL (#48)

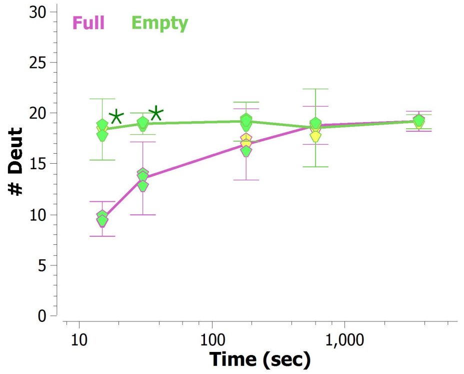

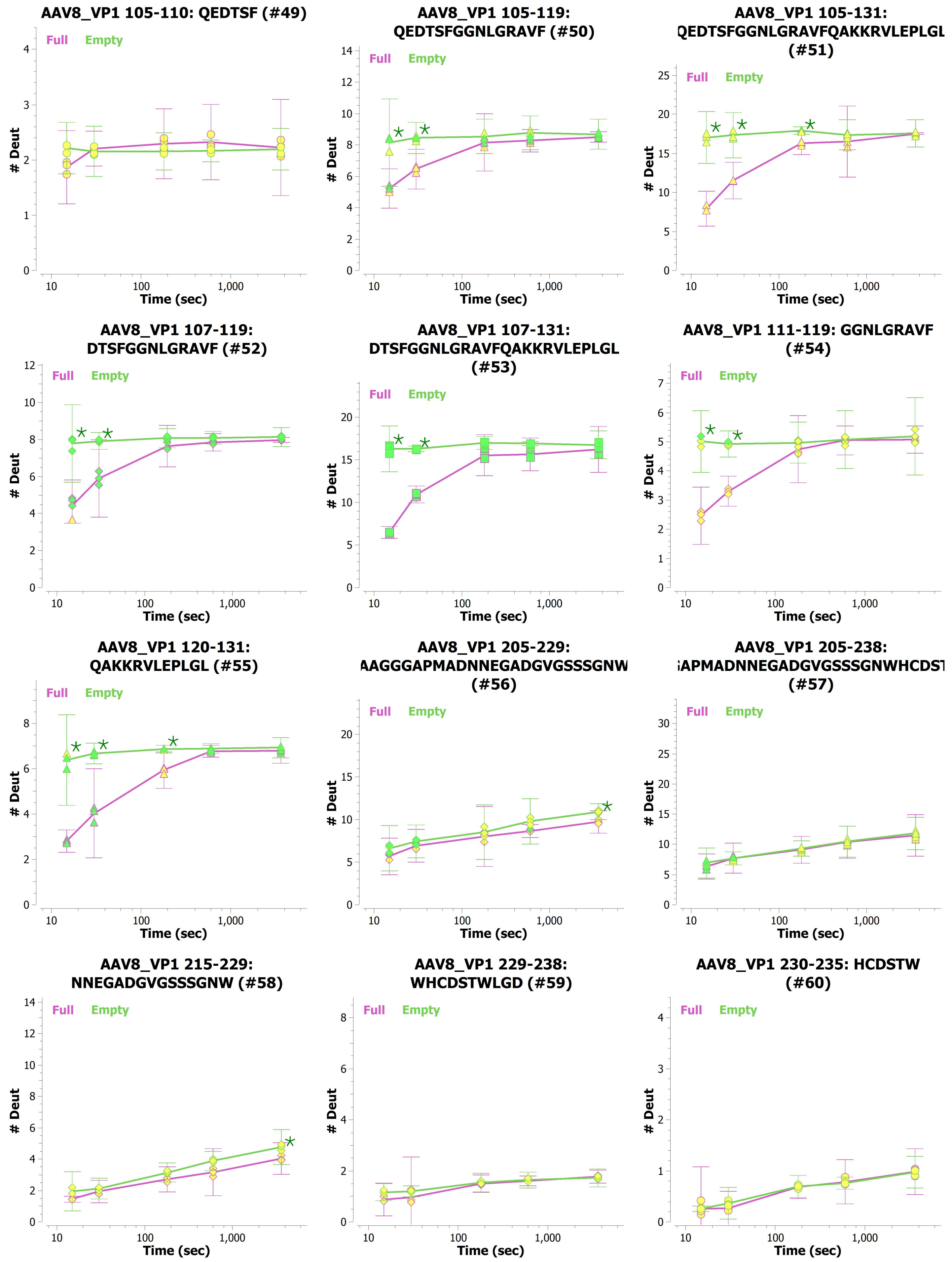
AAV8_VP1 105-110: QEDTSF (#49)
AAV8_VP1 105-119: QEDTSFGGNLGRAVF (#50)
AAV8_VP1 105-131: QEDTSFGGNLGRAVFQAKKRVLEPLGL (#51)
AAV8_VP1 107-119: DTSFGGNLGRAVF (#52)
AAV8_VP1 107-131: DTSFGGNLGRAVFQAKKRVLEPLGL (#53)
AAV8_VP1 111-119: GGNLGRAVF (#54)
AAV8_VP1 120-131: QAKKRVLEPLGL (#55)
AAV8_VP1 205-229: AAGGGAPMADNNEGADGVGSSSGNW (#56)
AAV8_VP1 205-238: APMADNNEGADGVGSSSGNWHCDST (#57)
AAV8_VP1 215-229: NNEGADGVGSSSGNW (#58)
AAV8_VP1 229-238: WHCDSTWLGD (#59)
AAV8_VP1 230-235: HCDSTW (#60)
Full
Empty
Deut
Time (sec)

AAV8_VP1 230-236: HCDSTWL (#61)

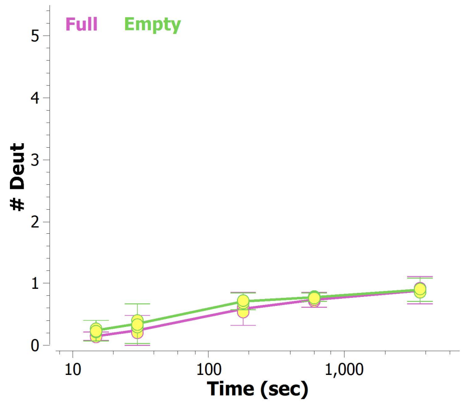

AAV8_VP1 230-238: HCDSTWLGD (#62)

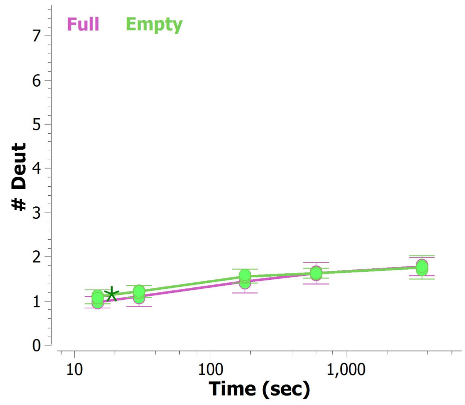

AAV8_VP1 230-240: HCDSTWLGDRV (#63)

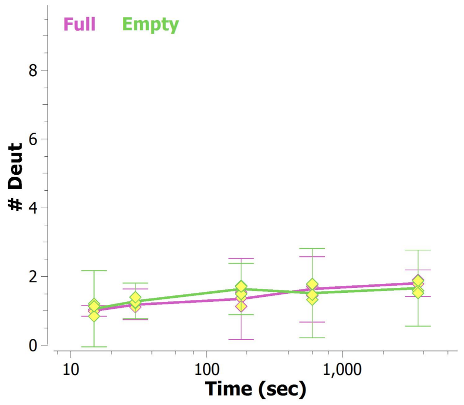

AAV8_VP1 230-242: HCDSTWLGDRVIT (#64)

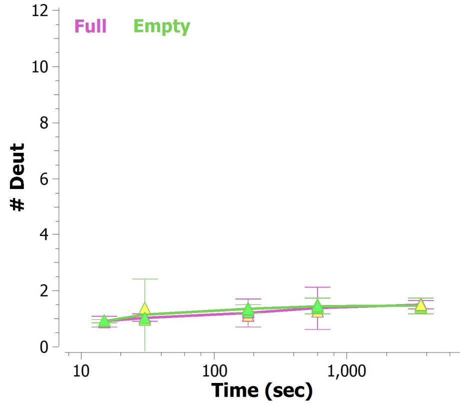

AAV8_VP1 230-243: HCDSTWLGDRVITT (#65)

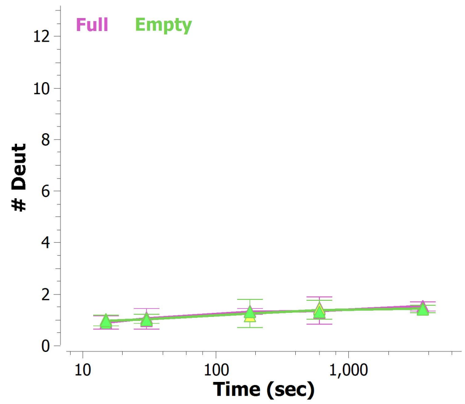

AAV8_VP1 230-245: HCDSTWLGDRVITTST (#66)

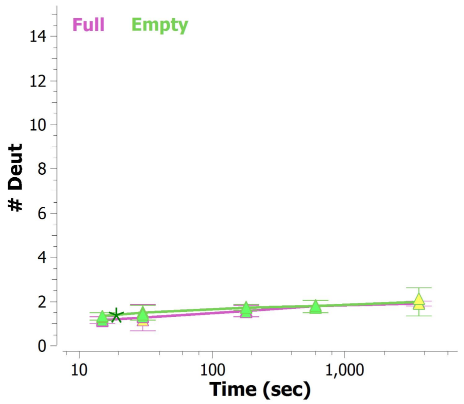

AAV8_VP1 230-247: HCDSTWLGDRVITTSTRT (#67)

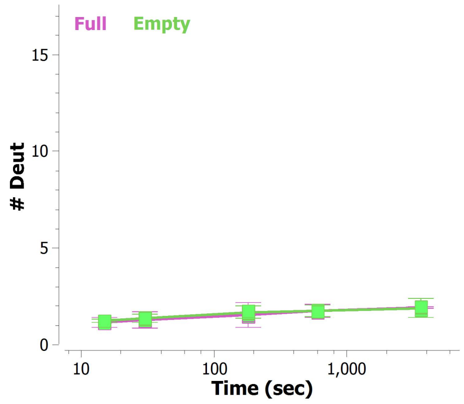

AAV8_VP1 230-257: CDSTWLGDRVITTSTRTWALPTYNNH (#68)

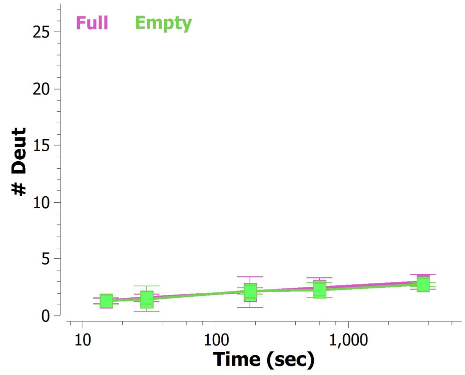

AAV8_VP1 232-243: DSTWLGDRVITT (#69)

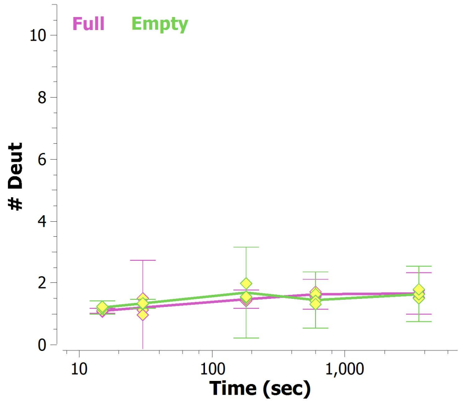

AAV8_VP1 232-247: DSTWLGDRVITTSTRT (#70)

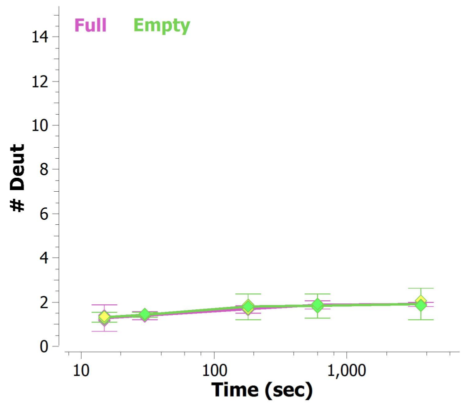

AAV8_VP1 232-257: DSTWLGDRVITTSTRTWALPTYNNHL (#71)

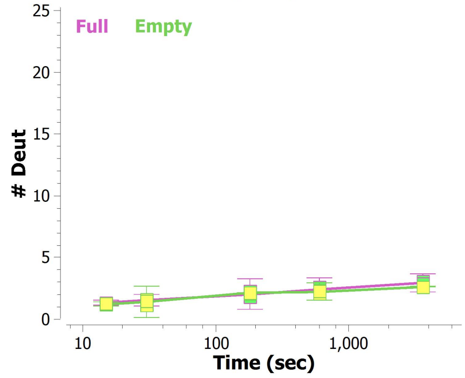

AAV8_VP1 234-238: TWLGD (#72)

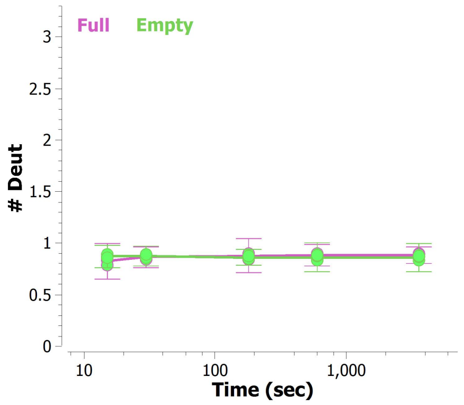

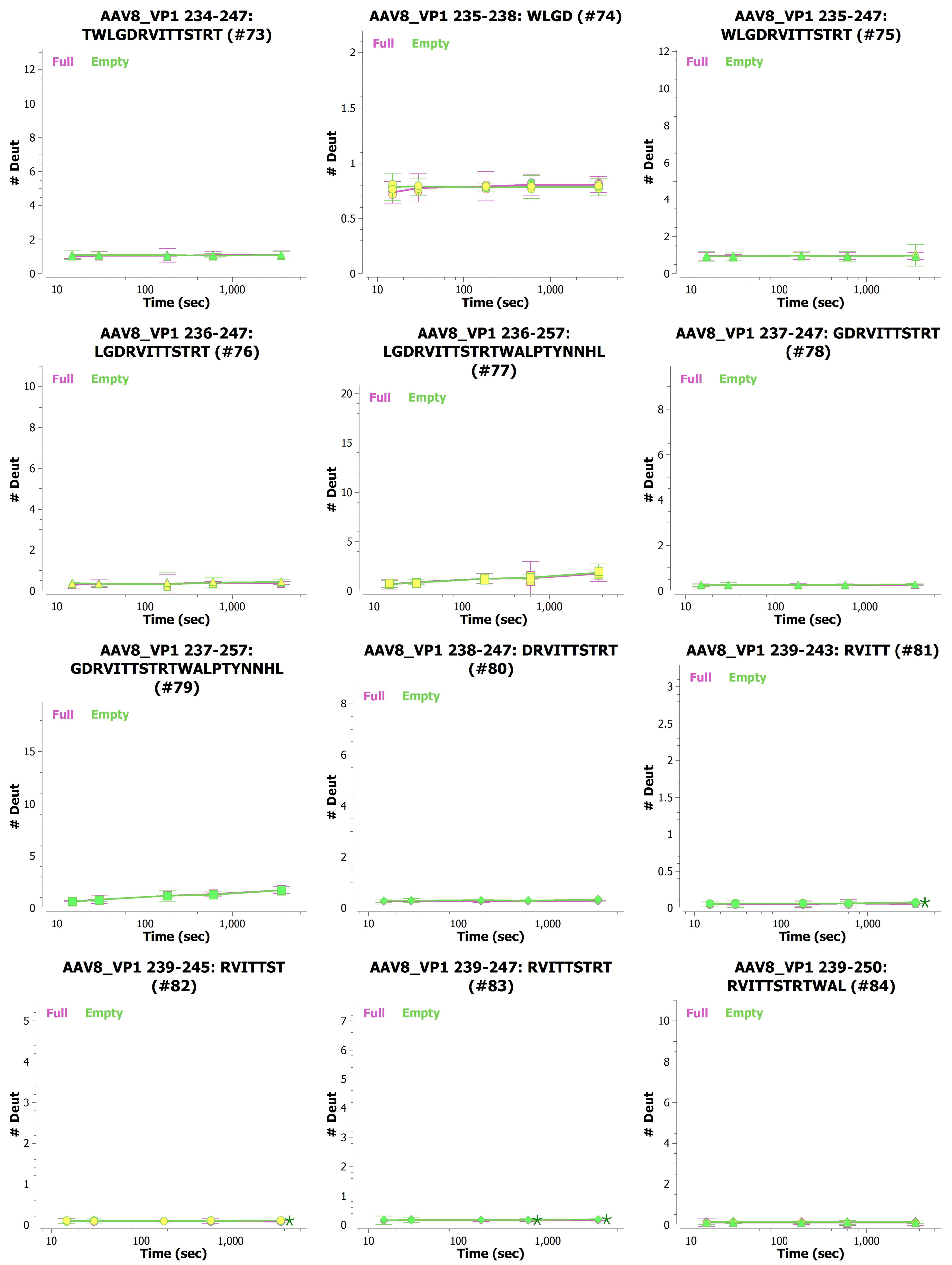
AAV8_VP1 234-247: TWLGDRVITTSTRT (#73)
AAV8_VP1 235-238: WLGD (#74)
AAV8_VP1 235-247: WLGDRVITTSTRT (#75)
AAV8_VP1 236-247: LGDRVITTSTRT (#76)
AAV8_VP1 236-257: LGDRVITTSTRTWALPTYNNHL (#77)
AAV8_VP1 237-247: GDRVITTSTRT (#78)
AAV8_VP1 237-257: GDRVITTSTRTWALPTYNNHL (#79)
AAV8_VP1 238-247: DRVITTSTRT (#80)
AAV8_VP1 239-243: RVITT (#81)
AAV8_VP1 239-245: RVITTST (#82)
AAV8_VP1 239-247: RVITTSTRT (#83)
AAV8_VP1 239-250: RVITTSTRTWAL (#84)
Full
Empty
Deut
Time (sec)
10
100
1,000

AAV8_VP1 239-257: RVITTSTRTWALPTYNNHL (#85)

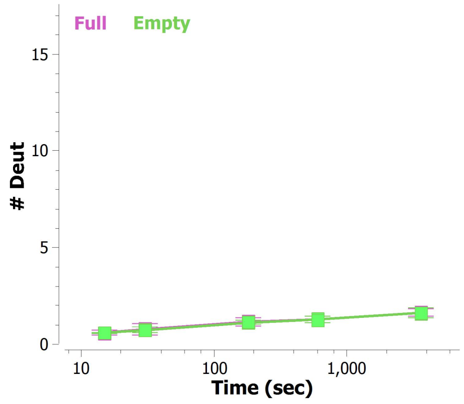

AAV8_VP1 241-257: ITTSTRTWALPTYNNHL (#86)

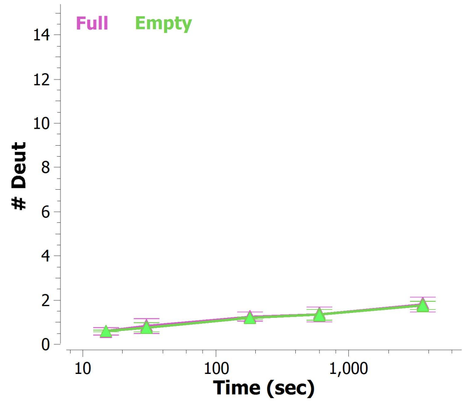

AAV8_VP1 243-247: TSTRT (#87)

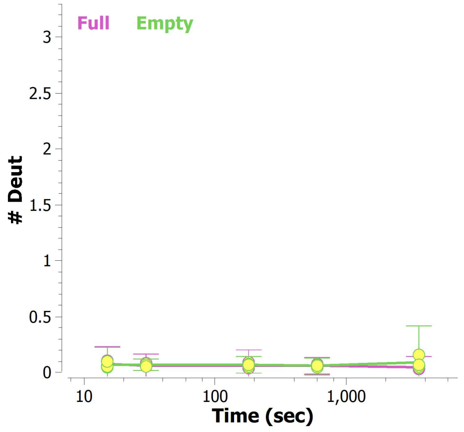

AAV8_VP1 243-257: TSTRTWALPTYNNHL (#88)

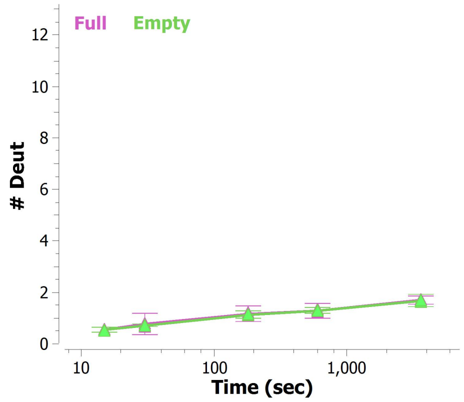

AAV8_VP1 244-257: STRTWALPTYNNHL (#89)

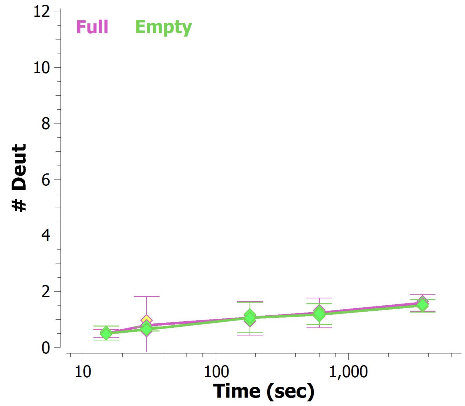

AAV8_VP1 245-257: TRTWALPTYNNHL (#90)

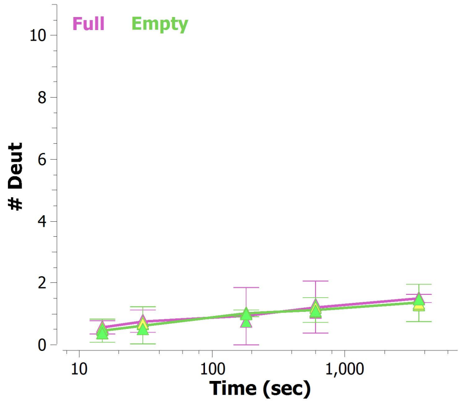

AAV8_VP1 246-257: RTWALPTYNNHL (#91)

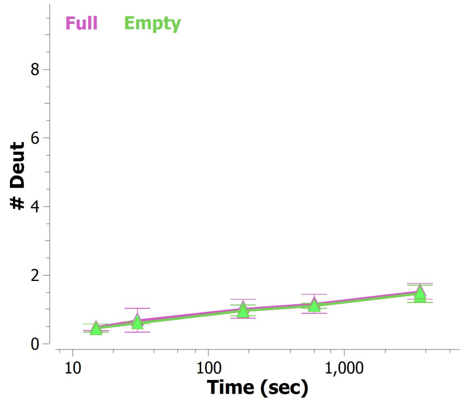

AAV8_VP1 248-257: WALPTYNNHL (#92)

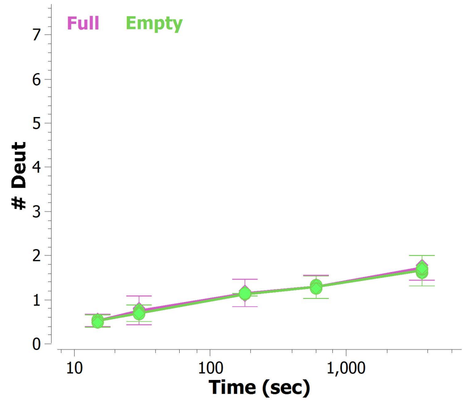

AAV8_VP1 250-257: LPTYNNHL (#93)

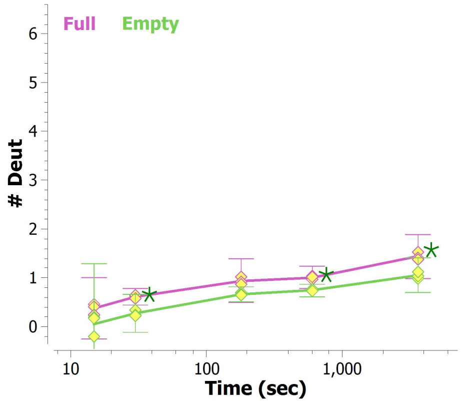

AAV8_VP1 251-257: PTYNNHL (#94)

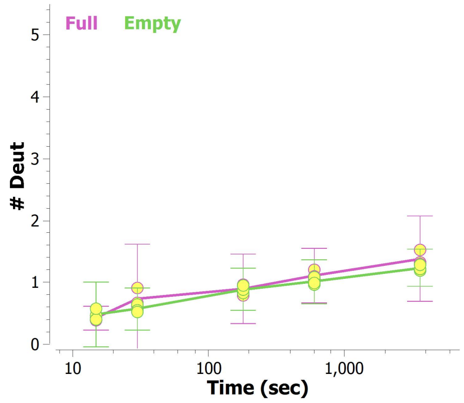

AAV8_VP1 258-264: YKQISNG (#95)

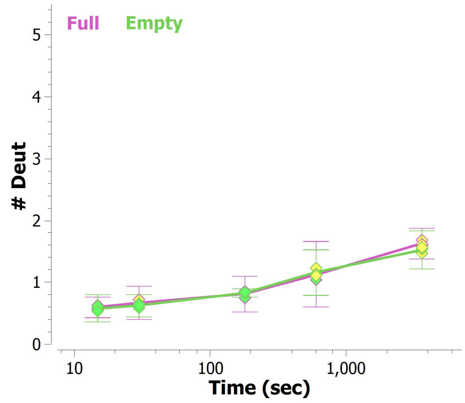

AAV8_VP1 258-265: YKQISNGT (#96)

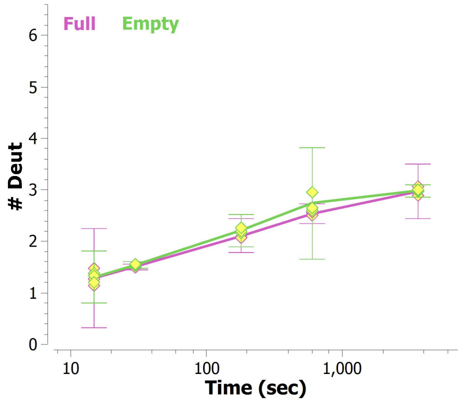

AAV8_VP1 258-266: YKQISNGTS (#97)

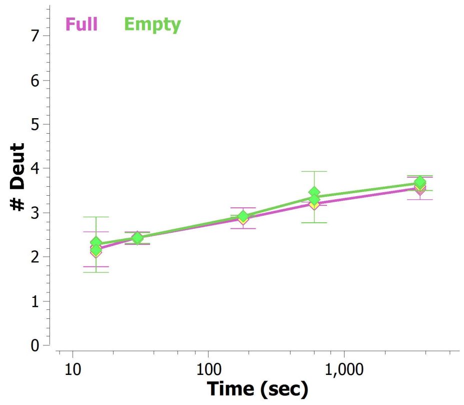

AAV8_VP1 258-272: YKQISNGTSGGATND (#98)

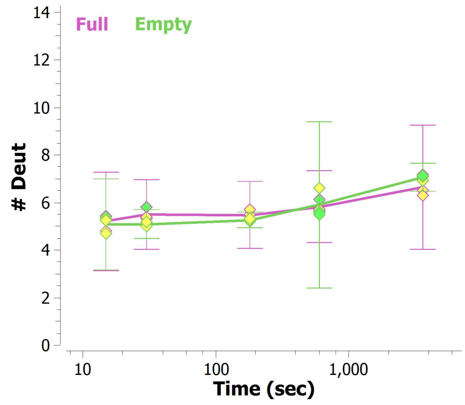

AAV8_VP1 258-274: YKQISNGTSGGATNDNT (#99)

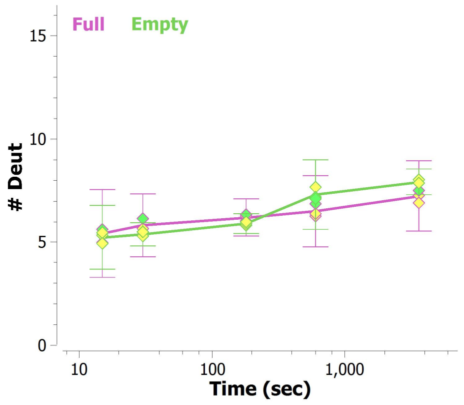

AAV8_VP1 258-275: YKQISNGTSGGATNDNTY (#100)

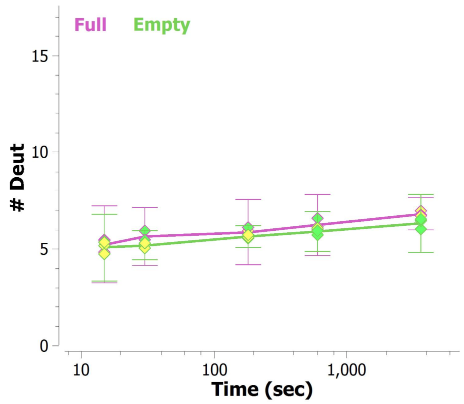

AAV8_VP1 273-284: NTYFGYSTPWGY (#101)

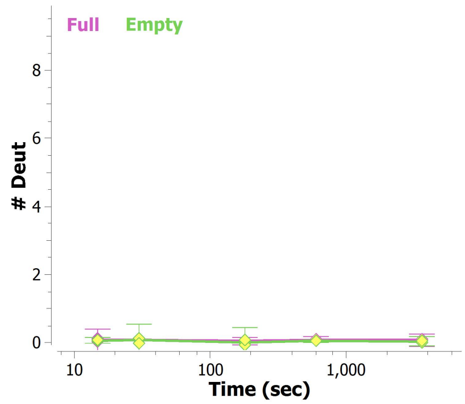

AAV8_VP1 277-284: GYSTPWGY (#102)

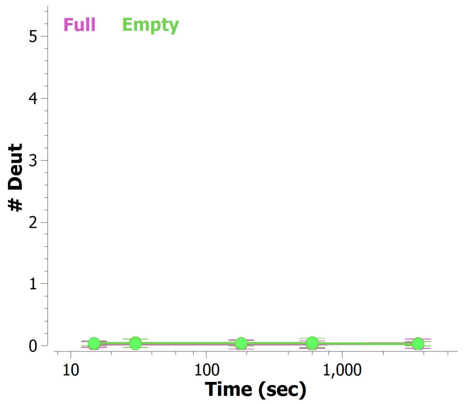

AAV8_VP1 277-285: GYSTPWGYF (#103)

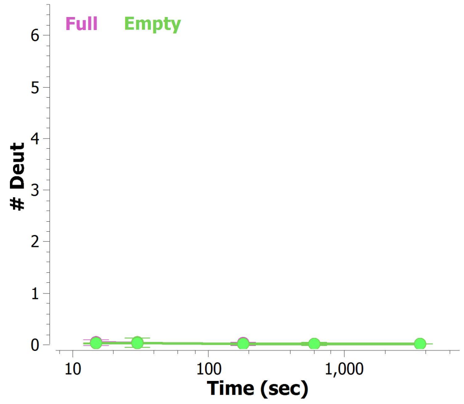

AAV8_VP1 278-284: YSTPWGY (#104)

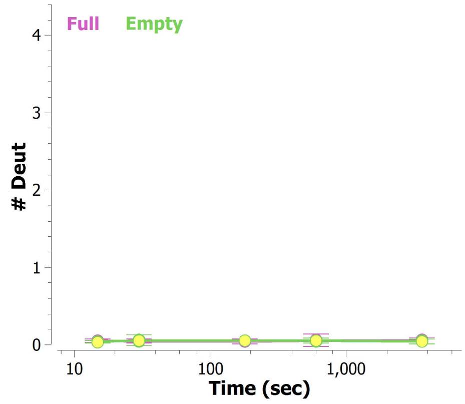

AAV8_VP1 278-285: YSTPWGYF (#105)

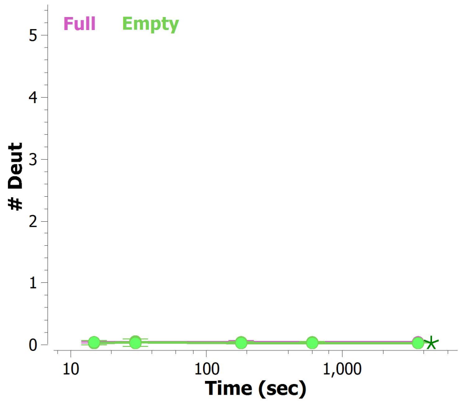

AAV8_VP1 278-286: YSTPWGYFD (#106)

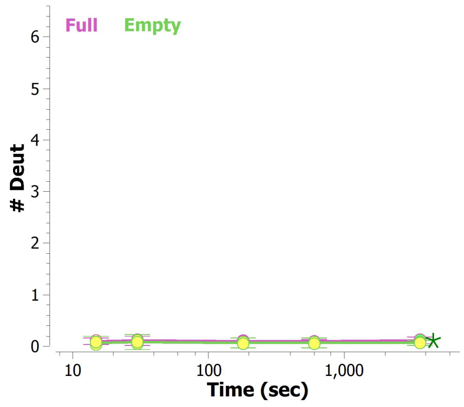

AAV8_VP1 285-298: FDFNRFHCHFSPRD (#107)

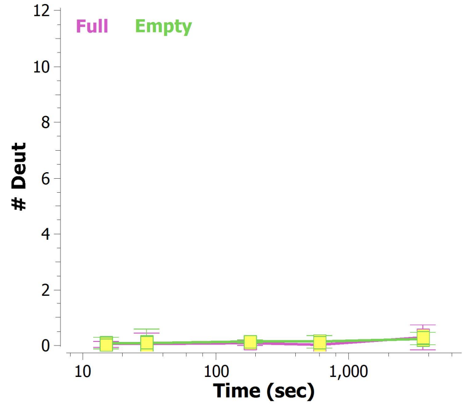

AAV8_VP1 286-294: DFNRFHCHF (#108)

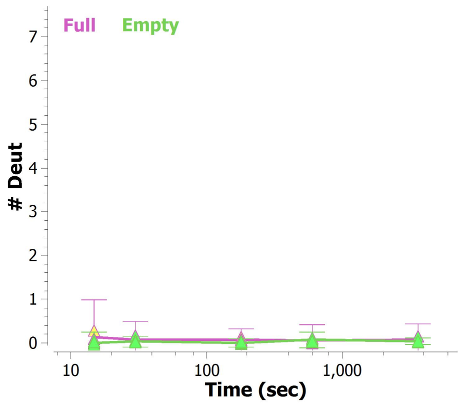

AAV8_VP1 286-302: DFNRFHCHFSPRDWQRL (#109)

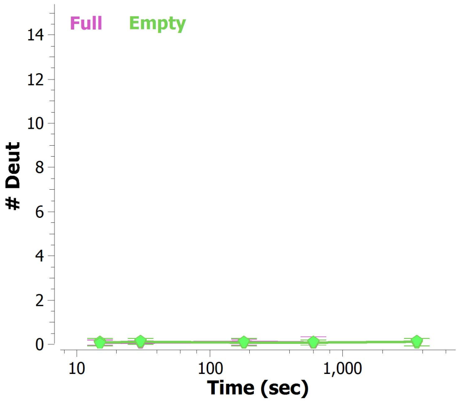

AAV8_VP1 288-302: NRFHCHFSPRDWQRL (#110)

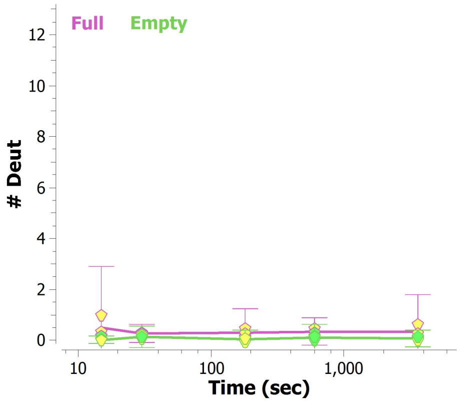

AAV8_VP1 288-306: NRFHCHFSPRDWQRLINNN (#111)

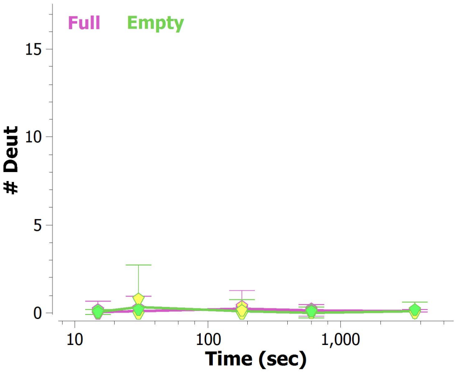

AAV8_VP1 291-298: HCHFSPRD (#112)

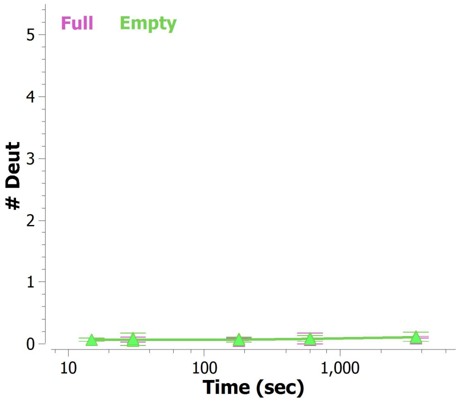

AAV8_VP1 291-302: HCHFSPRDWQRL (#113)

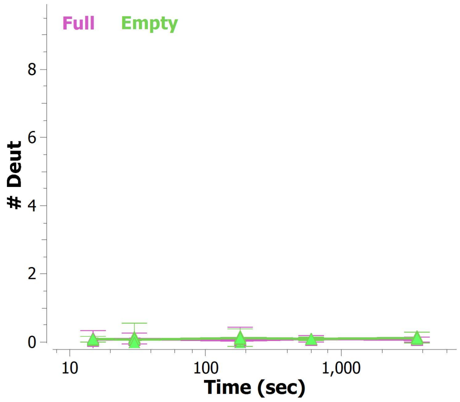

AAV8_VP1 291-306: HCHFSPRDWQRLINNN (#114)

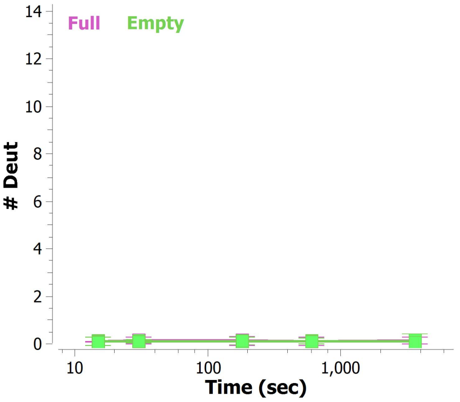

AAV8_VP1 291-307: HCHFSPRDWQRLINNNW (#115)

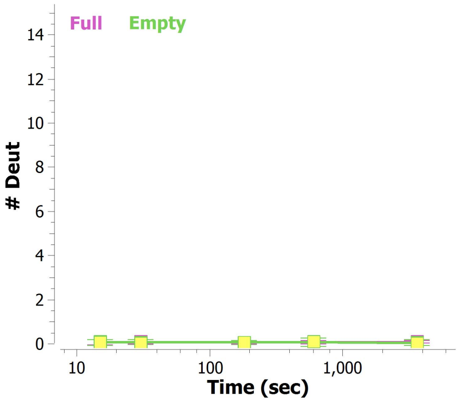

AAV8_VP1 299-306: WQRLINNN (#116)

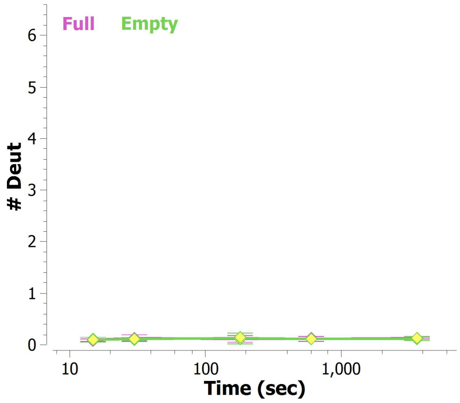

AAV8_VP1 299-307: WQRLINNNW (#117)

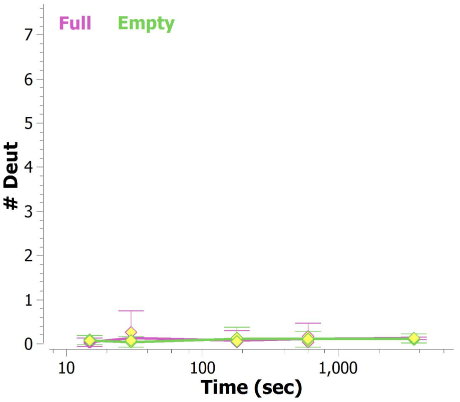

AAV8_VP1 299-316: WQRLINNNWGFRPKRLSF (#118)

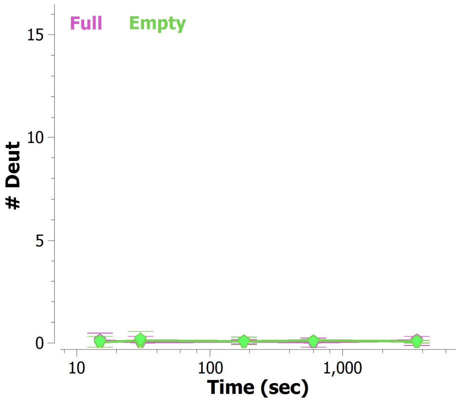

AAV8_VP1 299-318: WQRLINNNWGFRPKRLSFKL (#119)

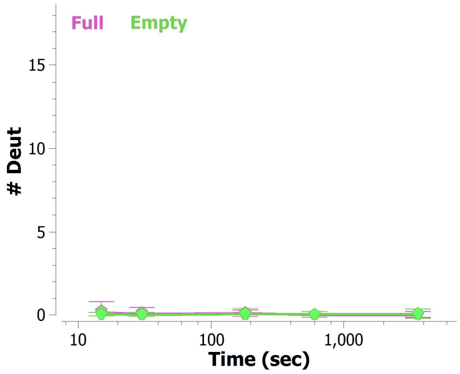

AAV8_VP1 300-316: QRLINNNWGFRPKRLSF (#120)

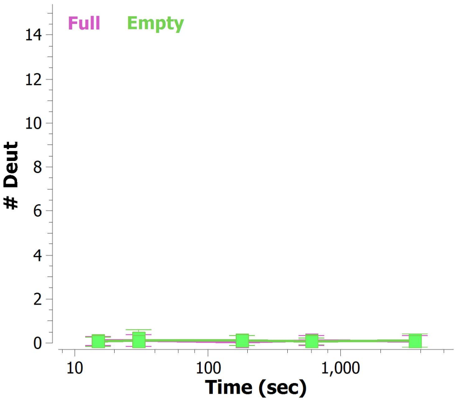

AAV8_VP1 303-316: INNNWGFRPKRLSF (#121)

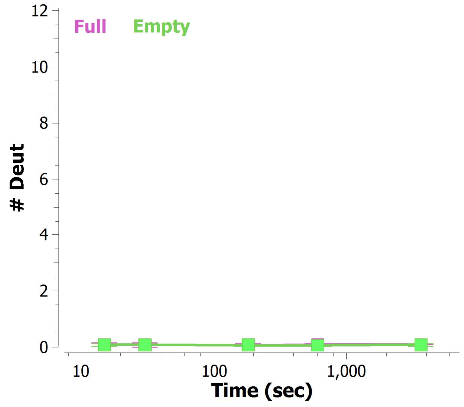

AAV8_VP1 303-318: INNNWGFRPKRLSFKL (#122)

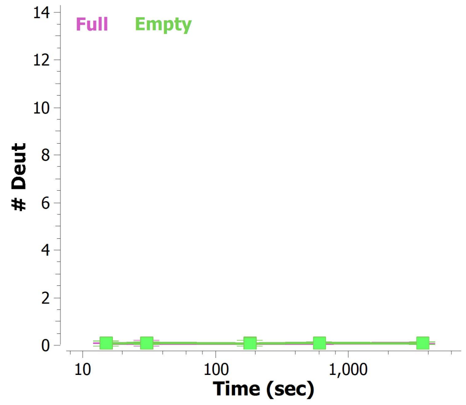

AAV8_VP1 307-318: WGFRPKRLSFKL (#123)

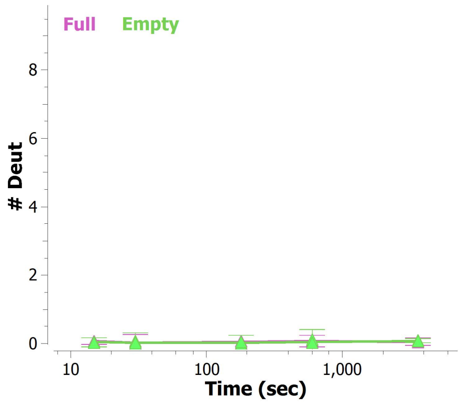

AAV8_VP1 317-320: KLFN (#124)

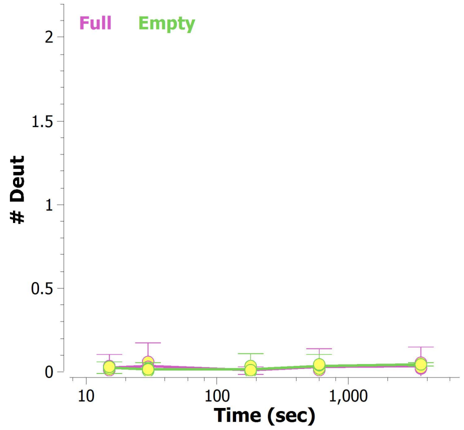

AAV8_VP1 317-322: KLFNIQ (#125)

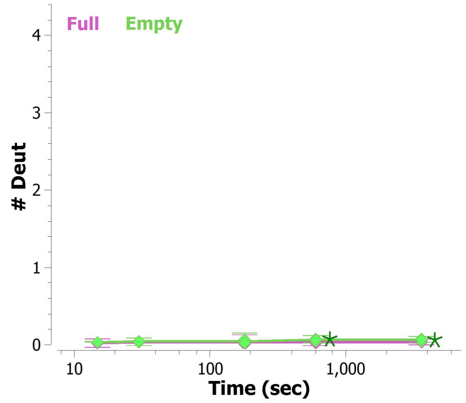

AAV8_VP1 317-323: KLFNIQV (#126)

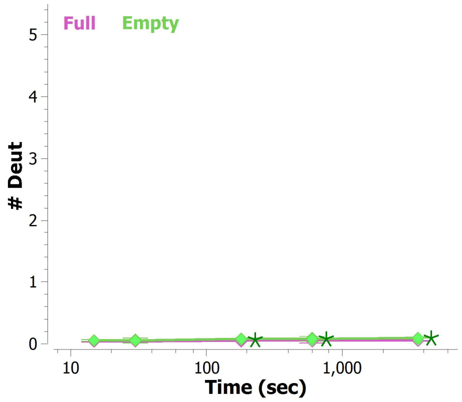

AAV8_VP1 317-325: KLFNIQVKE (#127)

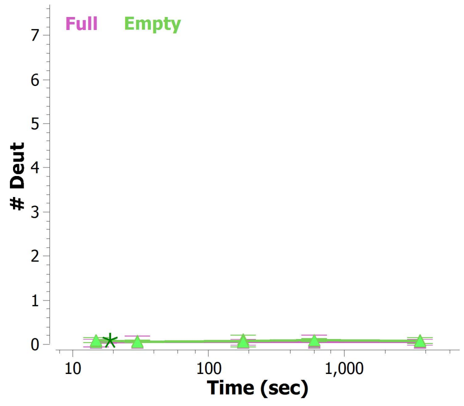

AAV8_VP1 317-338: KLFNIQVKEVTQNEGTKTIANN (#128)

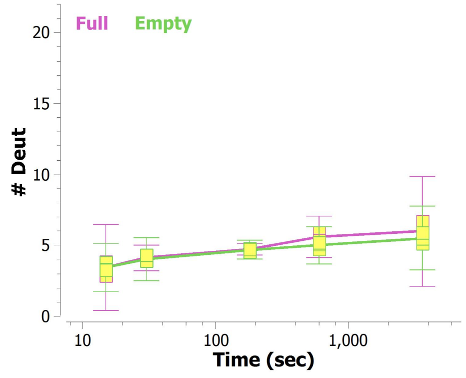

AAV8_VP1 317-339: KLFNIQVKEVTQNEGTKTIANNL (#129)

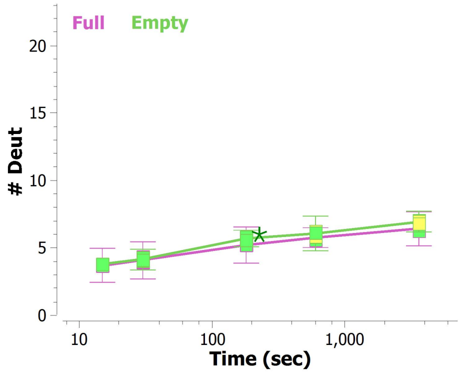

AAV8_VP1 317-341: KLFNIQVKEVTQNEGTKTIANNLTS (#130)

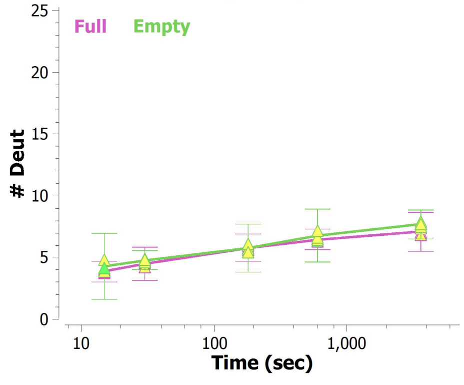

AAV8_VP1 317-342: KLFNIQVKEVTQNEGTKTIANNLTST (#131)

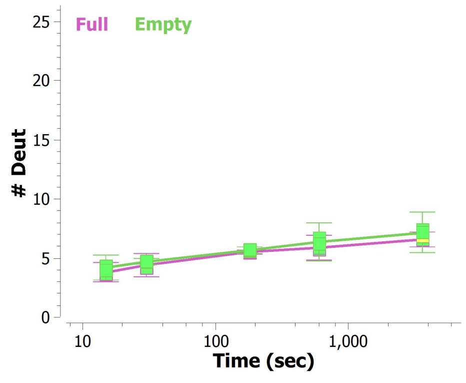

AAV8_VP1 317-344: (LFNIQVKEVTQNEGTKTIANNLTSTIC (#132)

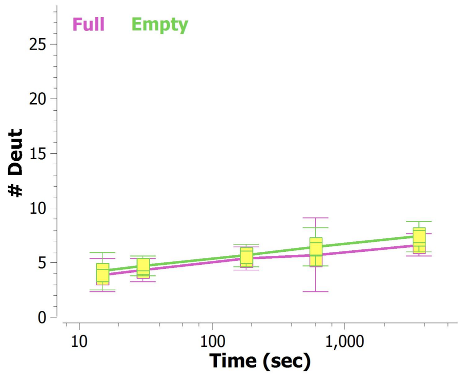

AAV8_VP1 317-345: [illegible]LFNIQVKEVTQNEGTKTIANNLTSTIQ[illegible] (#133)

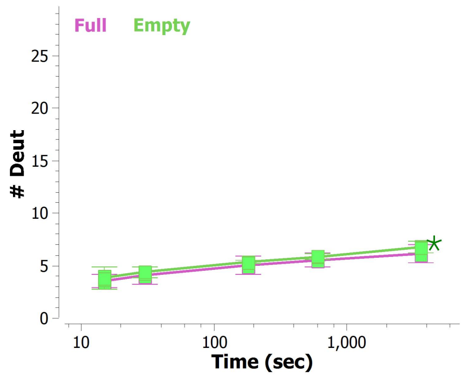

AAV8_VP1 319-322: FNIQ (#134)

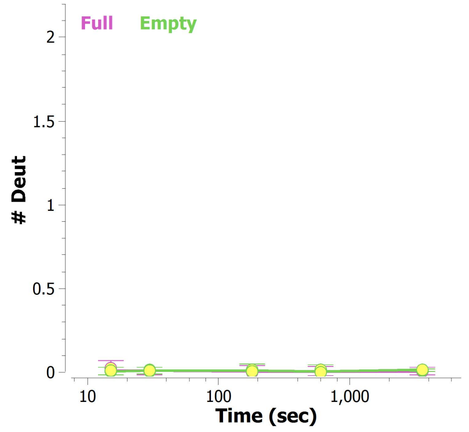

AAV8_VP1 319-325: FNIQVKE (#135)

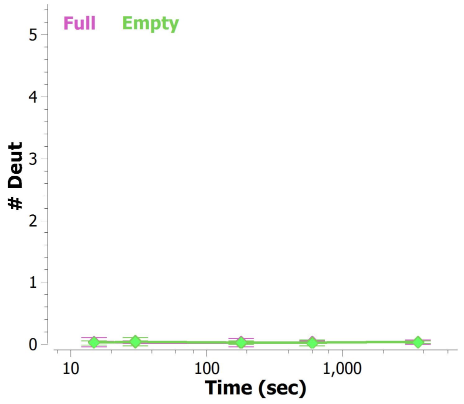

AAV8_VP1 319-334: FNIQVKEVTQNEGTKT (#136)

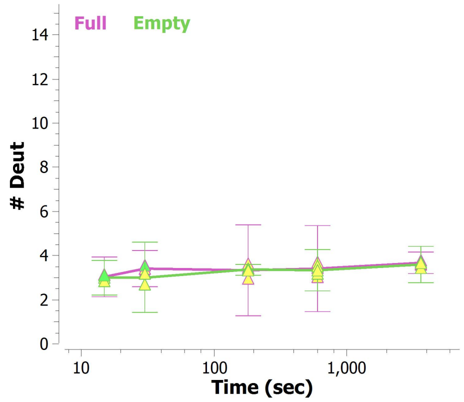

AAV8_VP1 319-339: FNIQVKEVTQNEGTKTIANNL (#137)

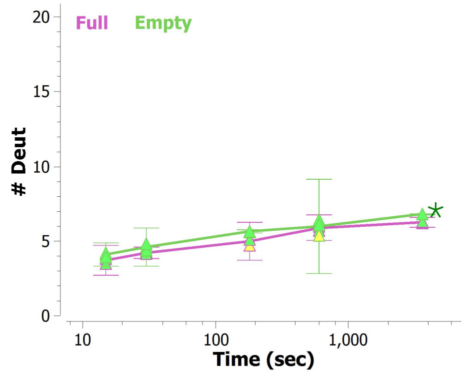

AAV8_VP1 319-341: FNIQVKEVTQNEGTKTIANNLTS (#138)

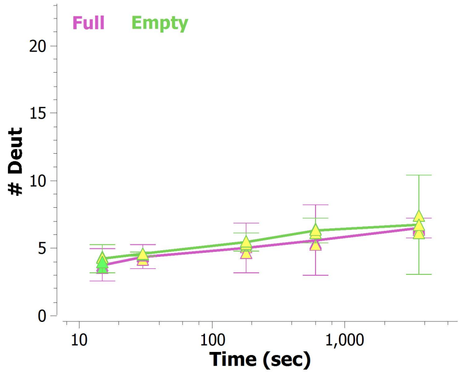

AAV8_VP1 319-342: FNIQVKEVTQNEGTKTIANNLTST (#139)

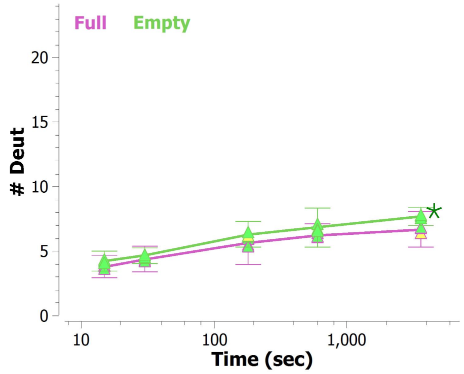

AAV8_VP1 319-344: FNIQVKEVTQNEGTKTIANNLTSTIQ (#140)

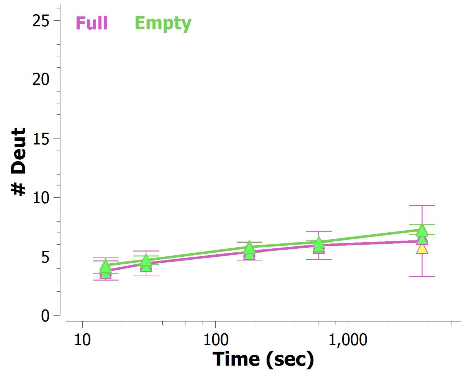

AAV8_VP1 319-345: FNIQVKEVTQNEGTKTIANNLTSTIQV (#141)

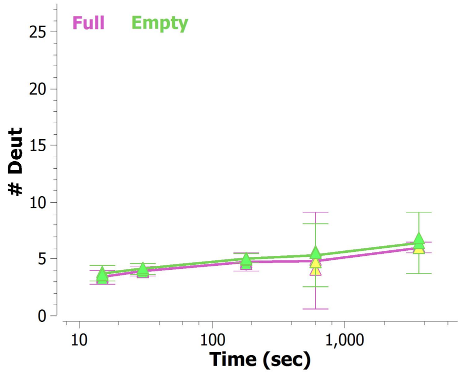

AAV8_VP1 320-339: NIQVKEVTQNEGTKTIANNL (#142)

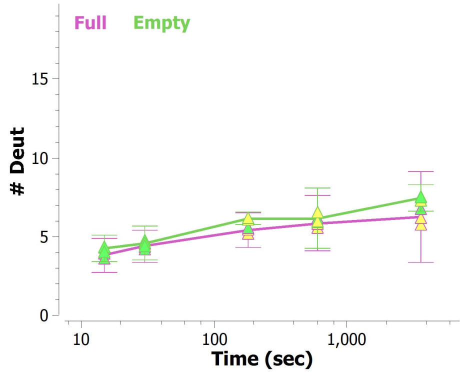

AAV8_VP1 320-341: NIQVKEVTQNEGTKTIANNLTS (#143)

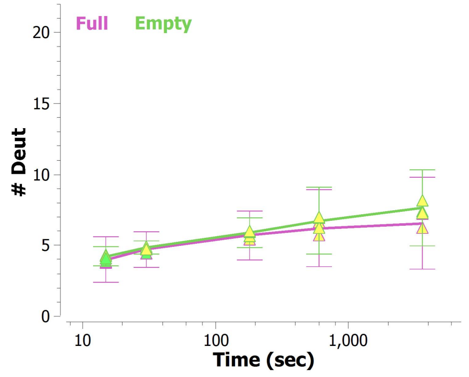

AAV8_VP1 320-342: NIQVKEVTQNEGTKTIANNLTST (#144)

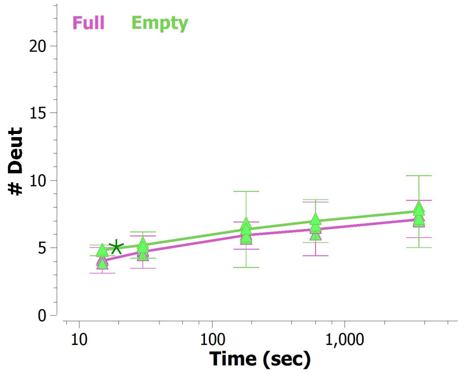

AAV8_VP1 320-345: NIQVKEVTQNEGTKTIANNLTSTIQV (#145)

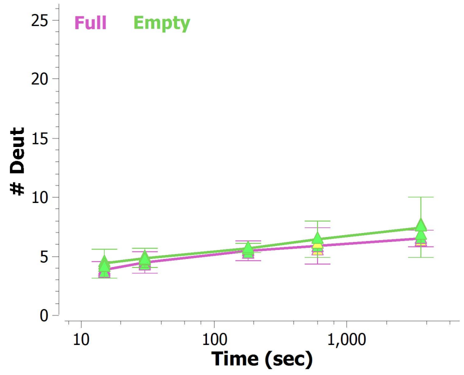

AAV8_VP1 320-346: NIQVKEVTQNEGTKTIANNLTSTIQVF (#146)

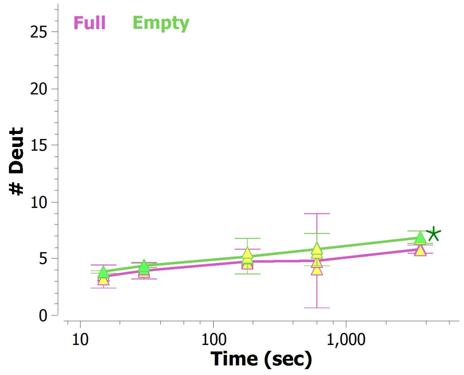

AAV8_VP1 320-350: QVKEVTQNEGTKTIANNLTSTIQVFTD (#147)

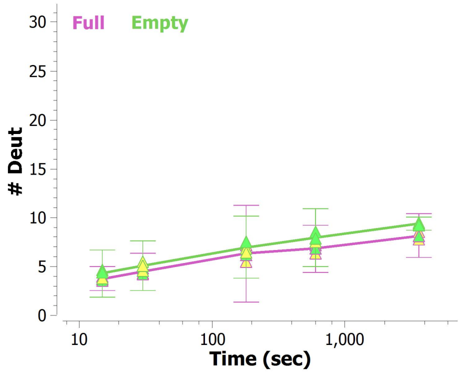

AAV8_VP1 321-342: IQVKEVTQNEGTKTIANNLTST (#148)

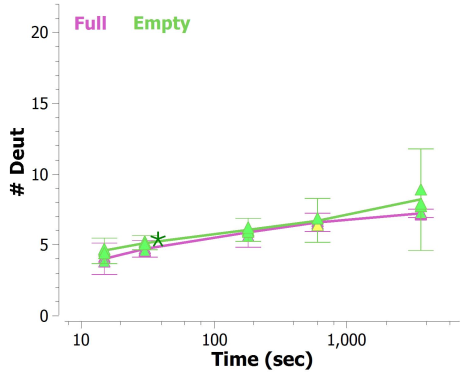

AAV8_VP1 321-345: IQVKEVTQNEGTKTIANNLTSTIQV (#149)

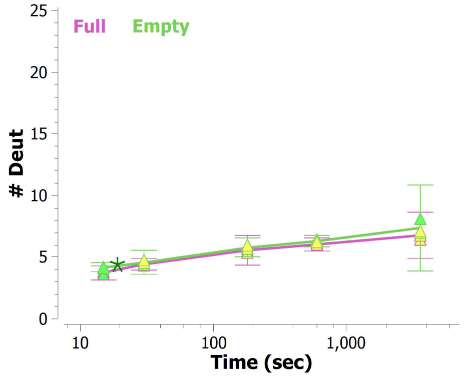

AAV8_VP1 323-339: VKEVTQNEGTKTIANNL (#150)

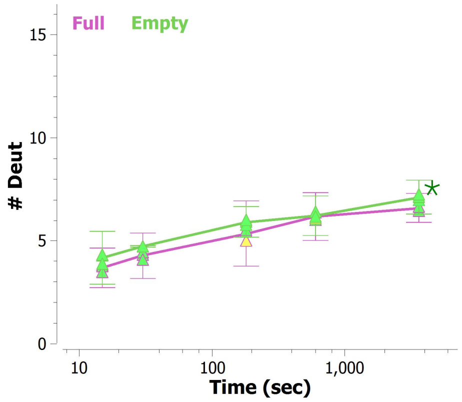

AAV8_VP1 323-342: VKEVTQNEGTKTIANNLTST (#151)

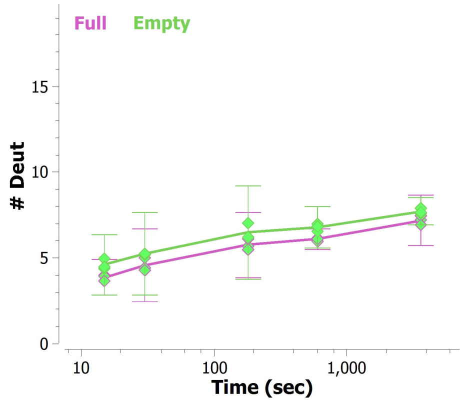

AAV8_VP1 323-344: VKEVTQNEGTKTIANNLTSTIQ (#152)

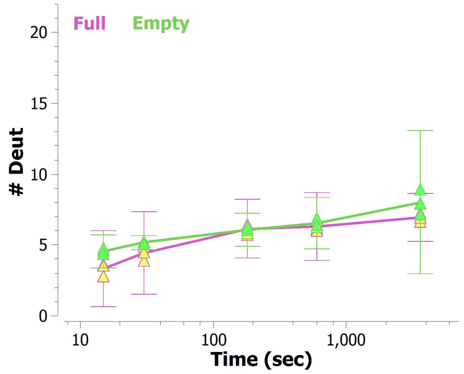

AAV8_VP1 323-345: VKEVTQNEGTKTIANNLTSTIQV (#153)

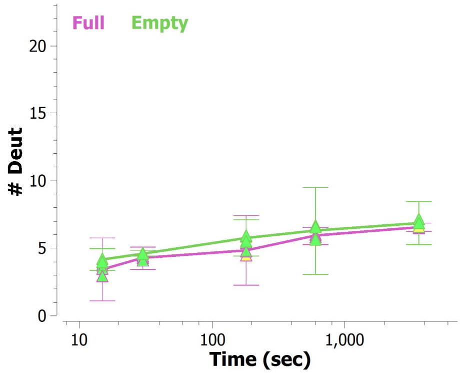

AAV8_VP1 323-346: VKEVTQNEGTKTIANNLTSTIQVF (#154)

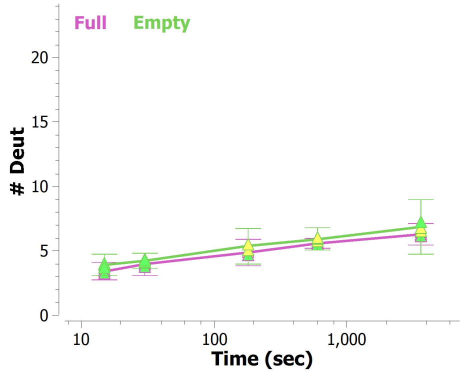

AAV8_VP1 323-347: VKEVTQNEGTKTIANNLTSTIQVFT (#155)

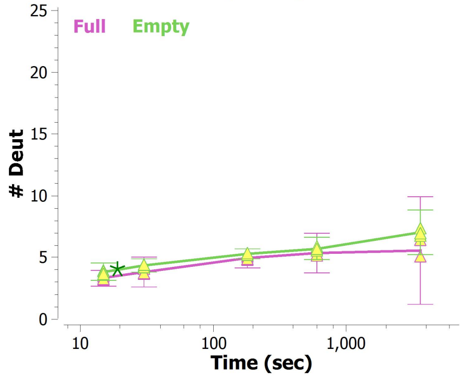

AAV8_VP1 323-350: VKEVTQNEGTKTIANNLTSTIQVFTDSE (#156)

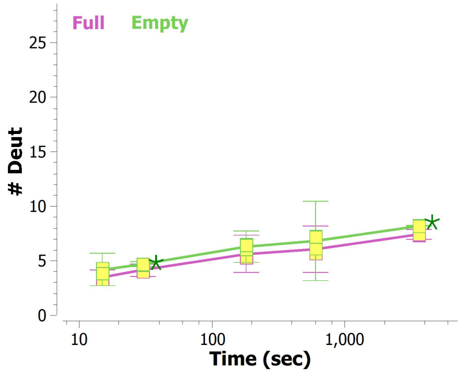

AAV8_VP1 324-339: KEVTQNEGTKTIANNL (#157)

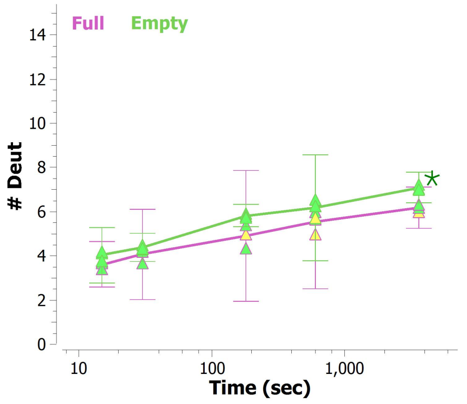

AAV8_VP1 324-342: KEVTQNEGTKTIANNLTST (#158)

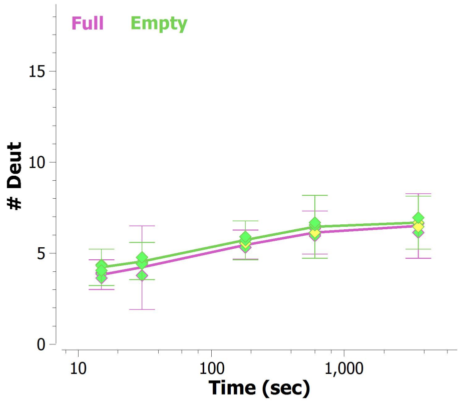

AAV8_VP1 324-345: KEVTQNEGTKTIANNLTSTIQV (#159)

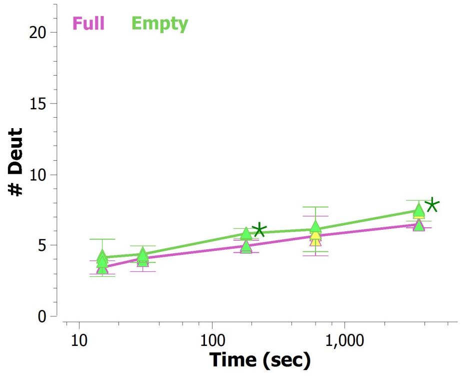

AAV8_VP1 324-346: KEVTQNEGTKTIANNLTSTIQVF (#160)

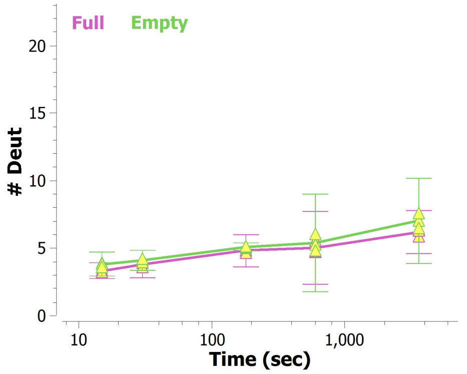

AAV8_VP1 326-342: VTQNEGTKTIANNLTST (#161)

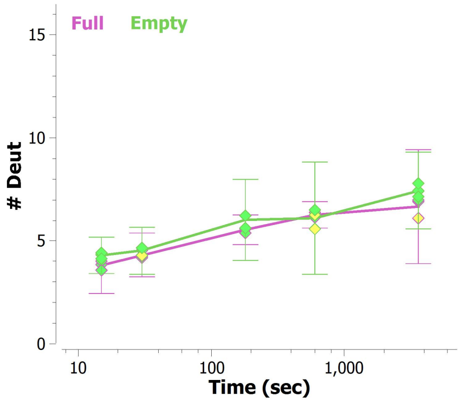

AAV8_VP1 326-350: VTQNEGTKTIANNLTSTIQVFTDSE (#162)

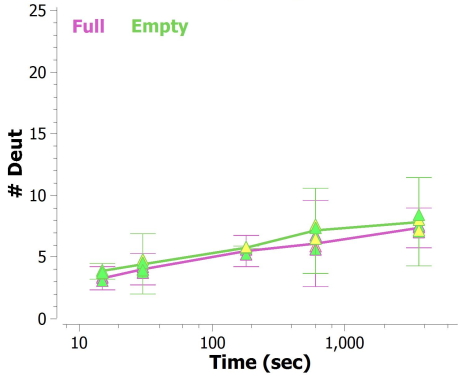

AAV8_VP1 330-342: EGTKTIANNLTST (#163)

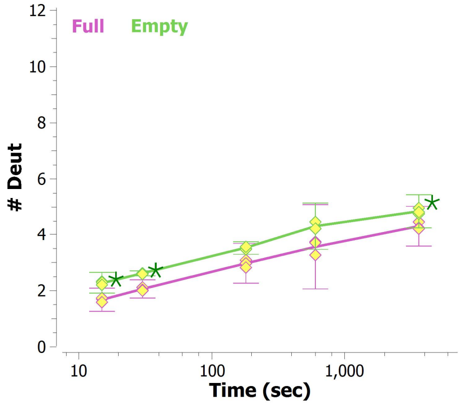

AAV8_VP1 335-339: IANNL (#164)

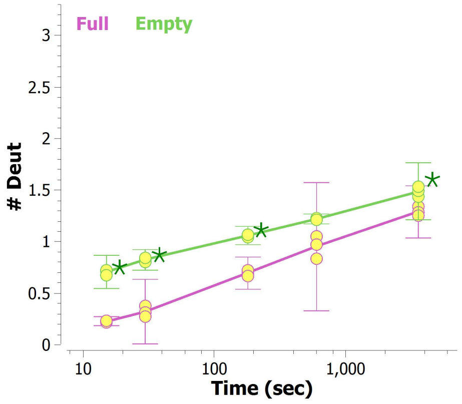

AAV8_VP1 335-342: IANNLTST (#165)

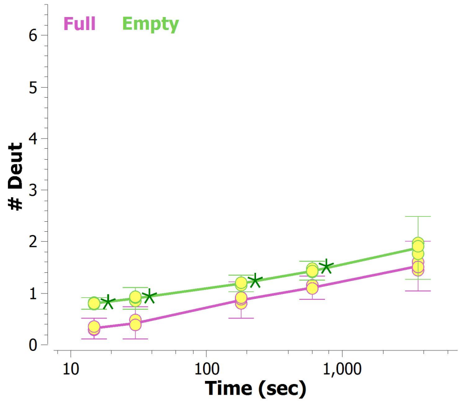

AAV8_VP1 340-345: TSTIQV (#166)

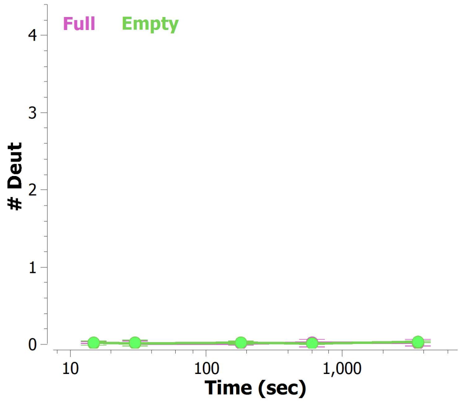

AAV8_VP1 340-346: TSTIQVF (#167)

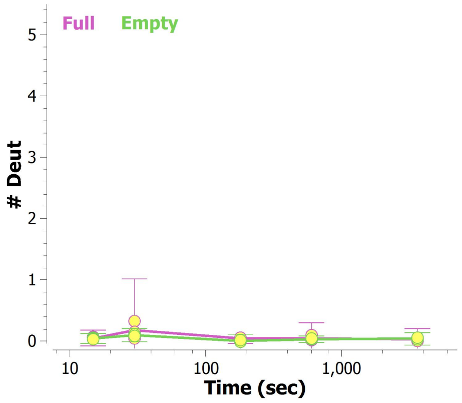

AAV8_VP1 340-350: TSTIQVFTDSE (#168)

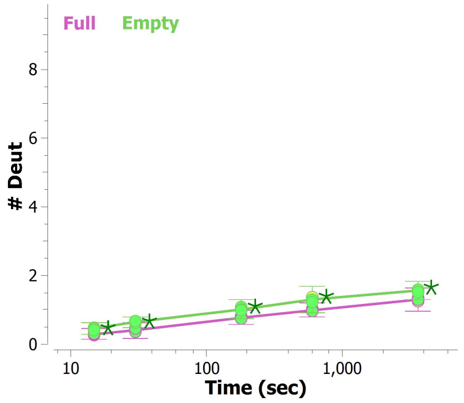

AAV8_VP1 343-346: IQVF (#169)

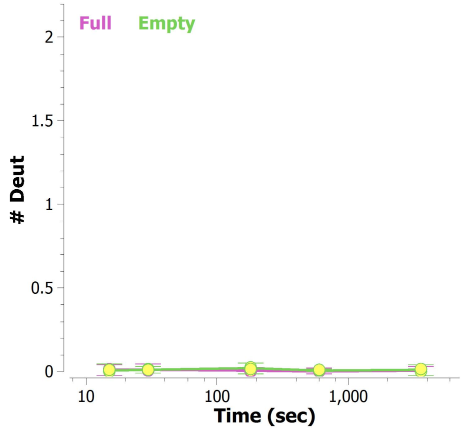

AAV8_VP1 343-347: IQVFT (#170)

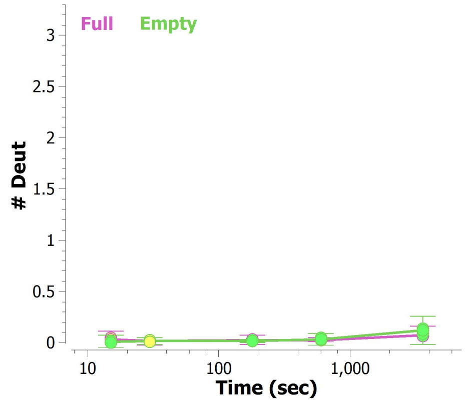

AAV8_VP1 343-349: IQVFTDS (#171)

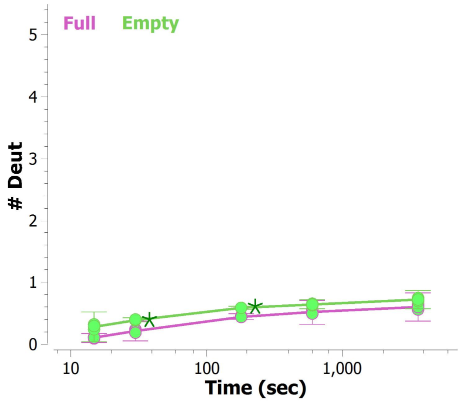

AAV8_VP1 343-350: IQVFTDSE (#172)

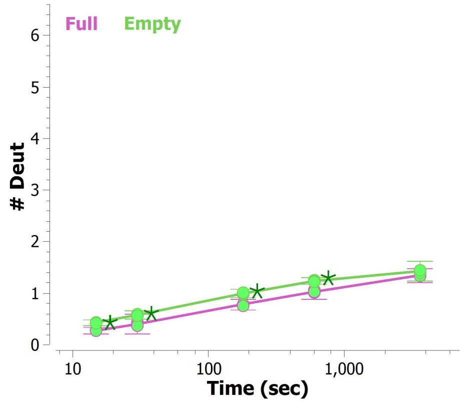

AAV8_VP1 345-350: VFTDSE (#173)

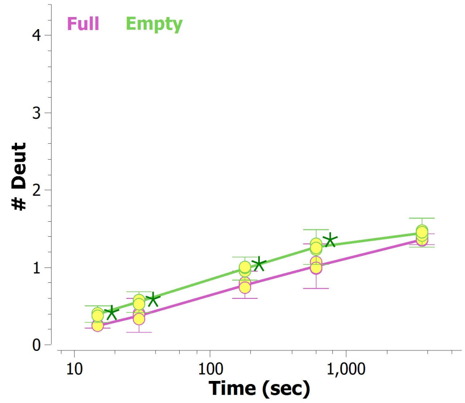

AAV8_VP1 346-350: FTDSE (#174)

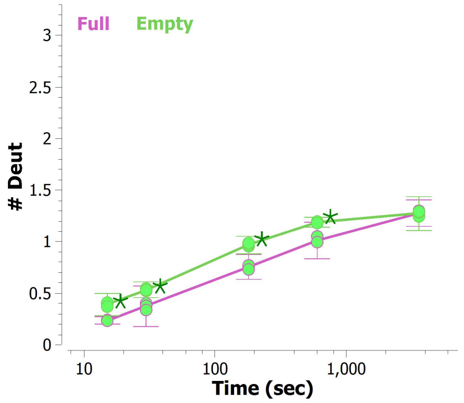

AAV8_VP1 351-358: YQLPYVLG (#175)

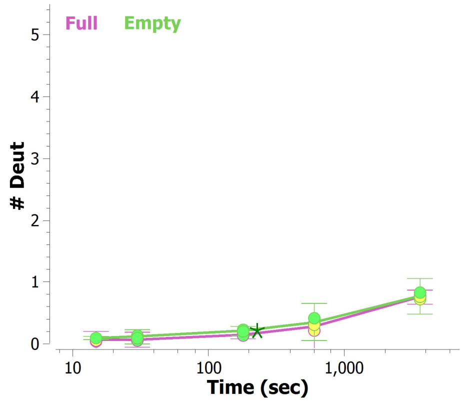

AAV8_VP1 351-360: YQLPYVLGSA (#176)

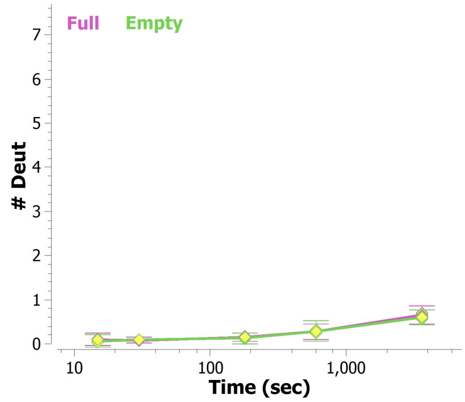

AAV8_VP1 351-371: YQLPYVLGSAHQGCLPPFPAD (#177)

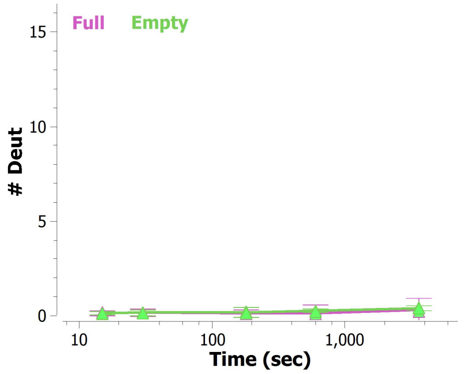

AAV8_VP1 351-372: YQLPYVLGSAHQGCLPPFPADV (#178)

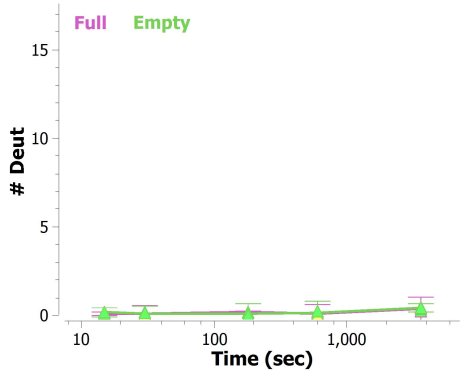

AAV8_VP1 351-373: YQLPYVLGSAHQGCLPPFPADVF (#179)

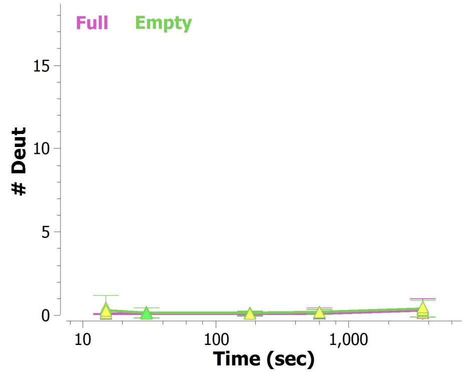

AAV8_VP1 372-379: VFMIPQYG (#180)

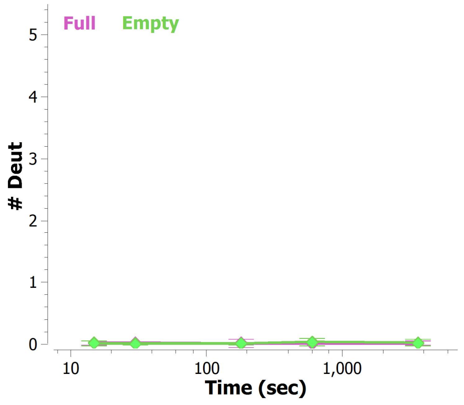

AAV8_VP1 372-380: VFMIPQYGY (#181)

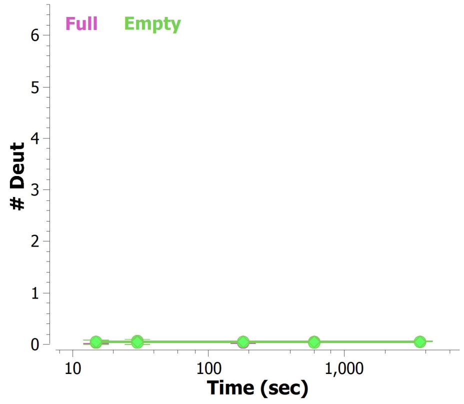

AAV8_VP1 372-381: VFMIPQYGYL (#182)

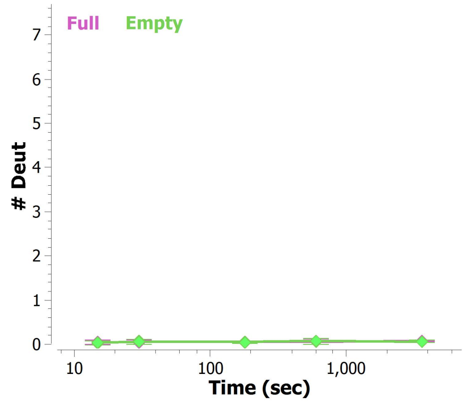

AAV8_VP1 373-379: FMIPQYG (#183)

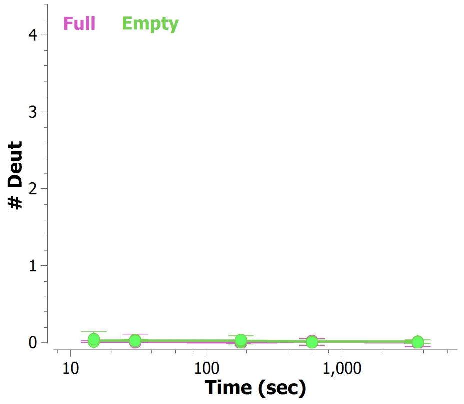

AAV8_VP1 373-380: FMIPQYGY (#184)

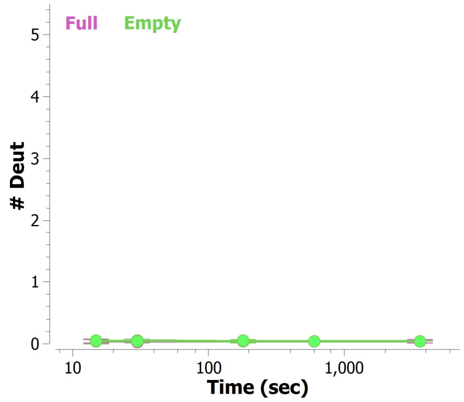

AAV8_VP1 373-381: FMIPQYGYL (#185)

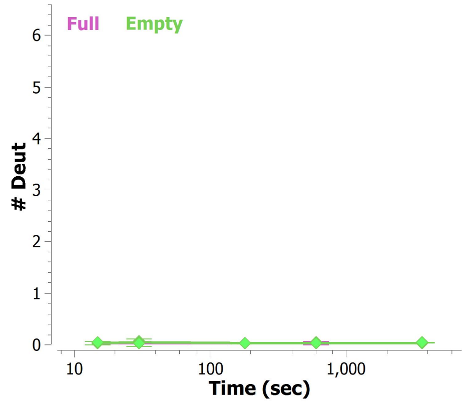

AAV8_VP1 373-388: FMIPQYGYLTLNNGSQ (#186)

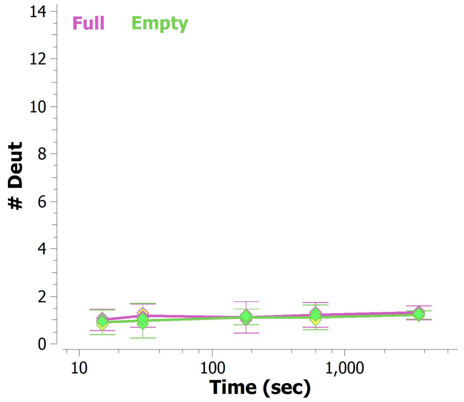

AAV8_VP1 373-395: FMIPQYGYLTLNNGSQAVGRSSF (#187)

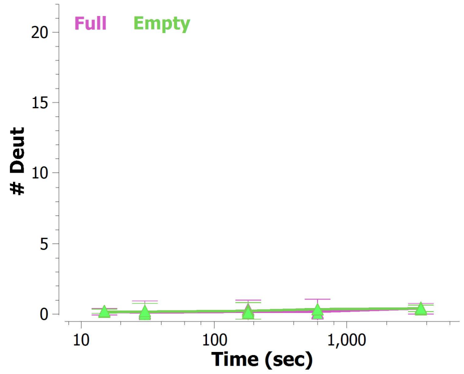

AAV8_VP1 374-380: MIPQYGY (#188)

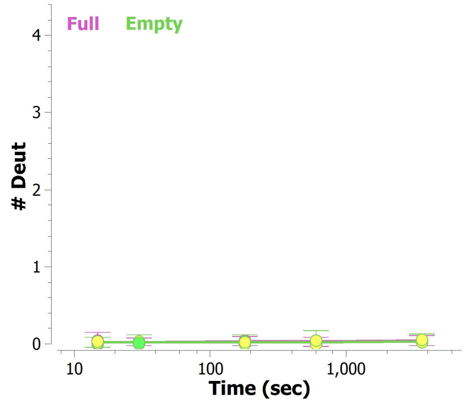

AAV8_VP1 374-381: MIPQYGYL (#189)

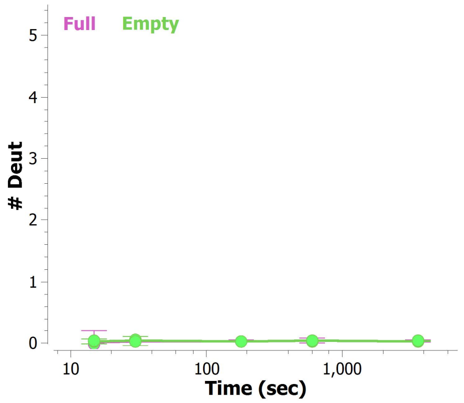

AAV8_VP1 374-383: MIPQYGYLTL (#190)

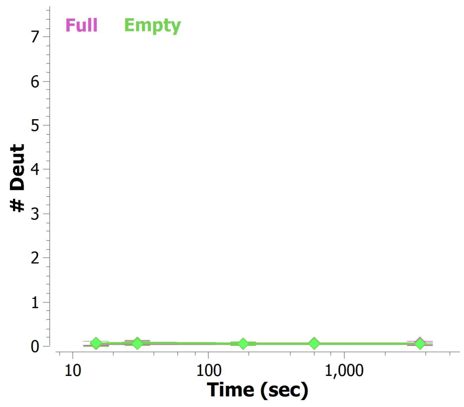

AAV8_VP1 374-388: MIPQYGYLTLNNGSQ (#191)

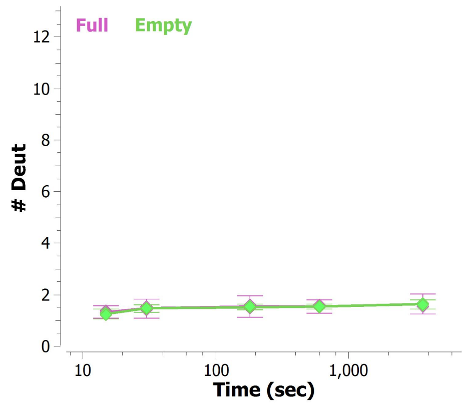

AAV8_VP1 374-395: MIPQYGYLTLNNGSQAVGRSSF (#192)

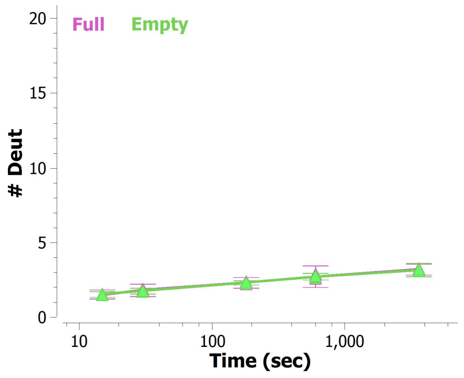

AAV8_VP1 375-381: IPQYGYL (#193)

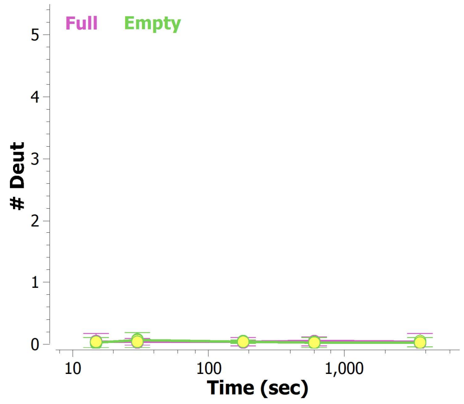

AAV8_VP1 375-383: IPQYGYLTL (#194)

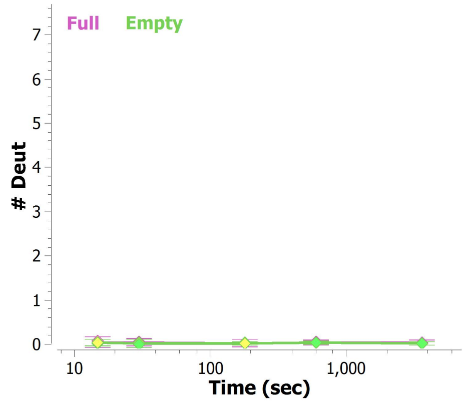

AAV8_VP1 375-395: IPQYGYLTLNNGSQAVGRSSF (#195)

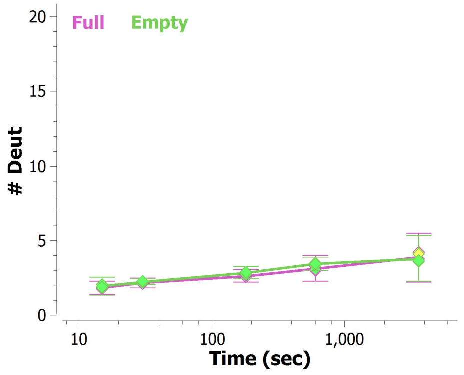

AAV8_VP1 376-380: PQYGY (#196)

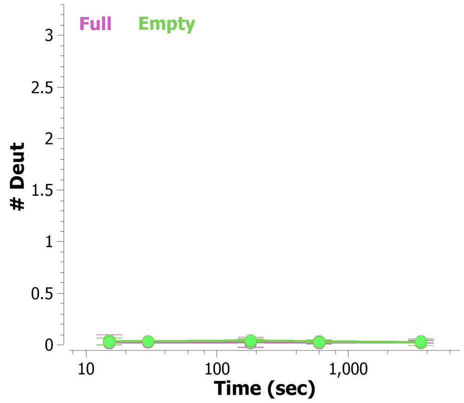

AAV8_VP1 380-388: YLTLNNGSQ (#197)

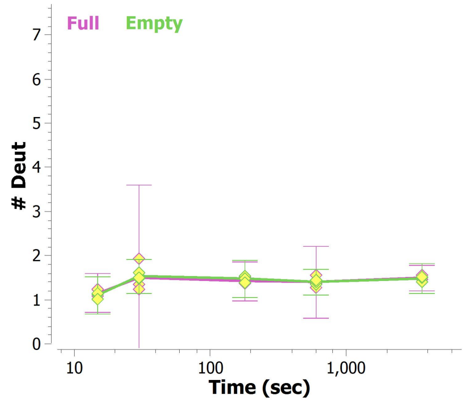

AAV8_VP1 380-395: YLTLNNGSQAVGRSSF (#198)

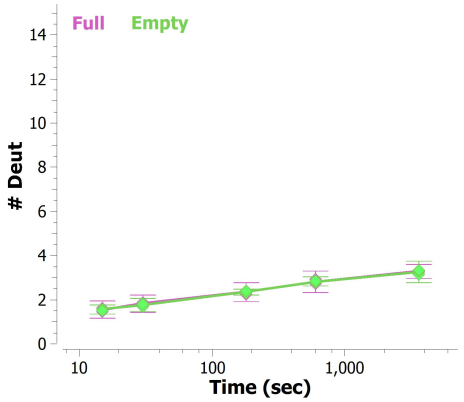

AAV8_VP1 381-388: LTLNNGSQ (#199)

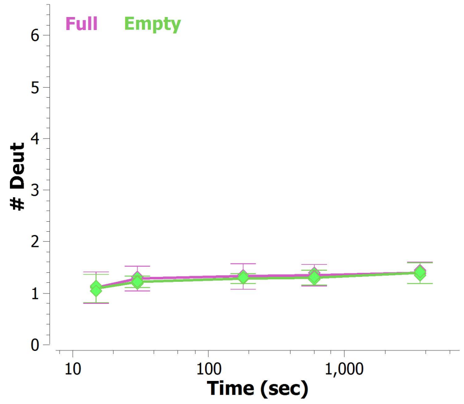

AAV8_VP1 381-395: LTLNNGSQAVGRSSF (#200)

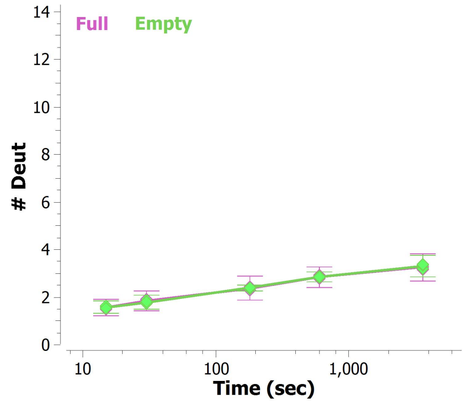

AAV8_VP1 382-388: TLNNGSQ (#201)

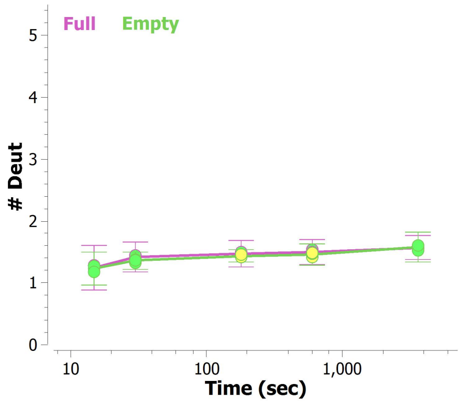

AAV8_VP1 382-389: TLNNGSQA (#202)

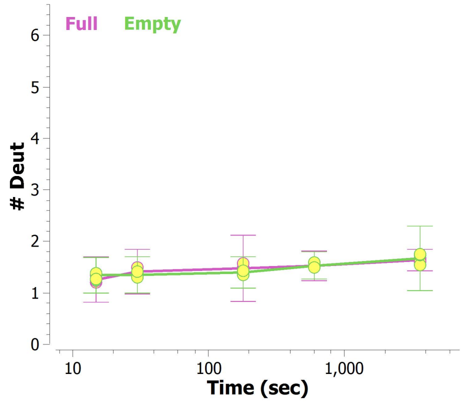

AAV8_VP1 382-395: TLNNGSQAVGRSSF (#203)

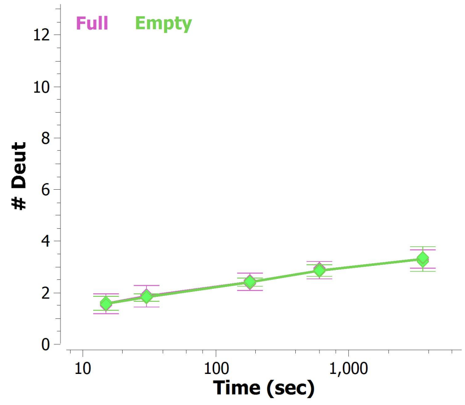

AAV8_VP1 384-395: NNGSQAVGRSSF (#204)

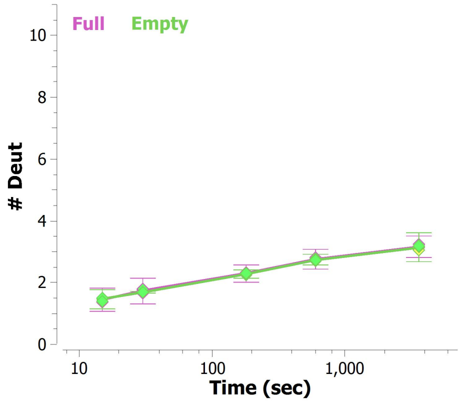

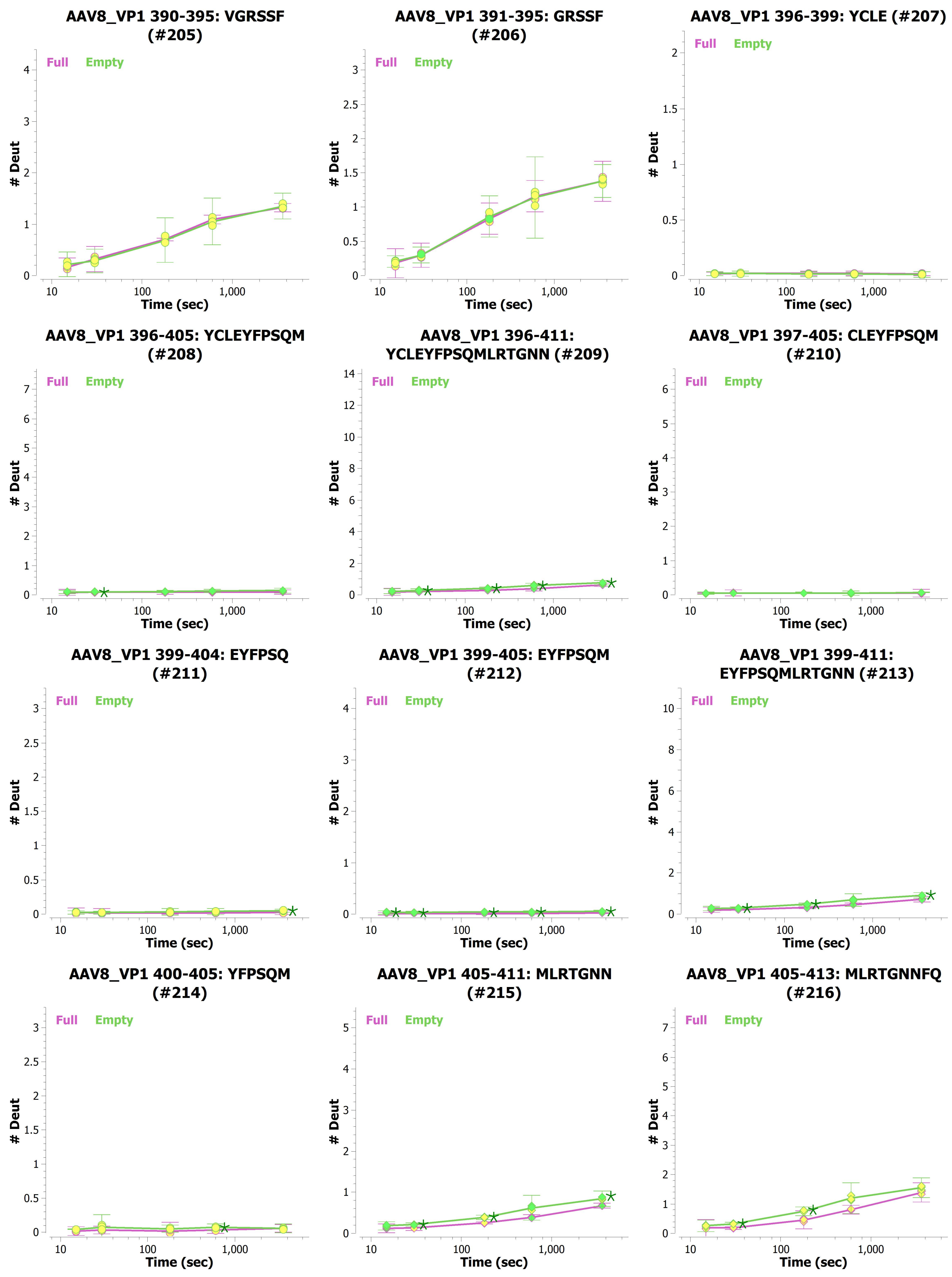
AAV8_VP1 390-395: VGRSSF (#205)
AAV8_VP1 391-395: GRSSF (#206)
AAV8_VP1 396-399: YCLE (#207)
AAV8_VP1 396-405: YCLEYFPSQM (#208)
AAV8_VP1 396-411: YCLEYFPSQMLRTGNN (#209)
AAV8_VP1 397-405: CLEYFPSQM (#210)
AAV8_VP1 399-404: EYFPSQ (#211)
AAV8_VP1 399-405: EYFPSQM (#212)
AAV8_VP1 399-411: EYFPSQMLRTGNN (#213)
AAV8_VP1 400-405: YFPSQM (#214)
AAV8_VP1 405-411: MLRTGNN (#215)
AAV8_VP1 405-413: MLRTGNNFQ (#216)
Full
Empty
Deut
Time (sec)
10
100
1,000

AAV8_VP1 406-411: LRTGNN (#217)

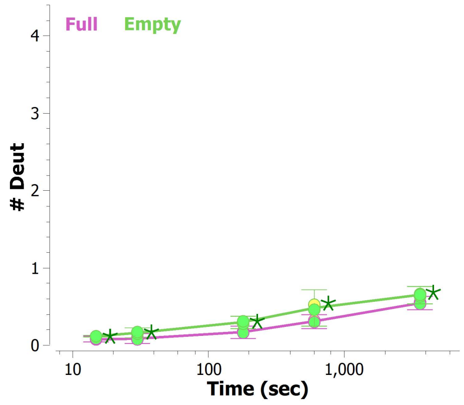

AAV8_VP1 406-412: LRTGNNF (#218)

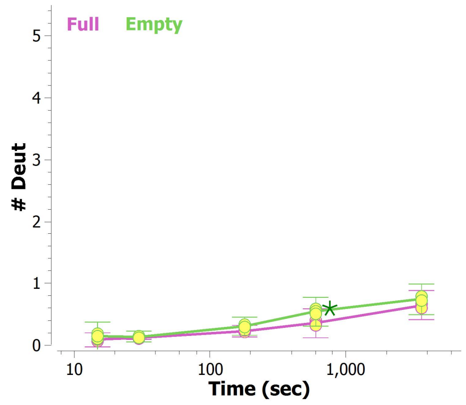

AAV8_VP1 406-413: LRTGNNFQ (#219)

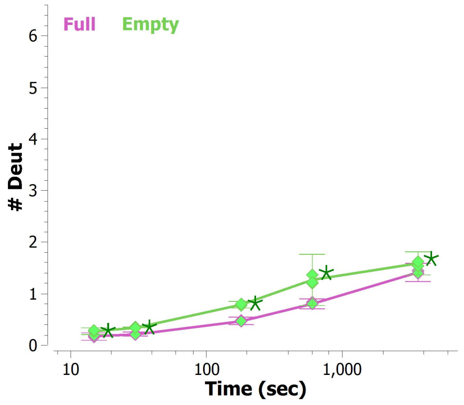

AAV8_VP1 406-414: LRTGNNFQF (#220)

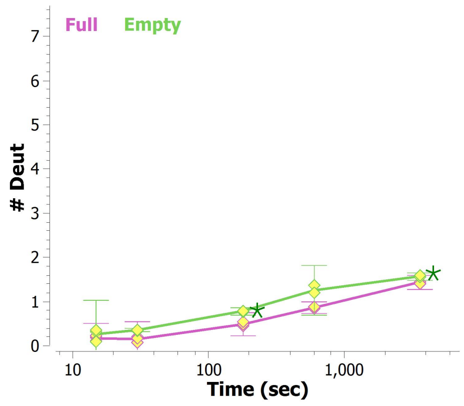

AAV8_VP1 406-415: LRTGNNFQFT (#221)

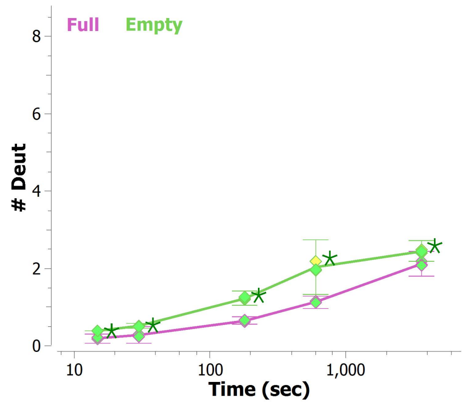

AAV8_VP1 406-417: LRTGNNFQFTYT (#222)

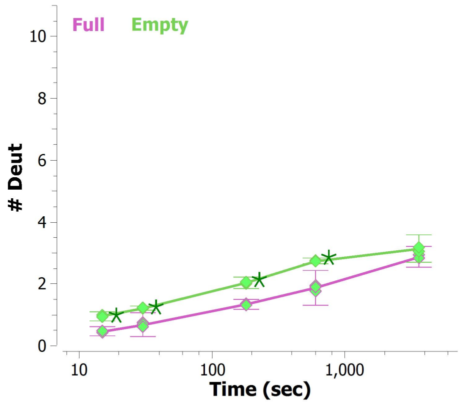

AAV8_VP1 412-417: FQFTYT (#223)

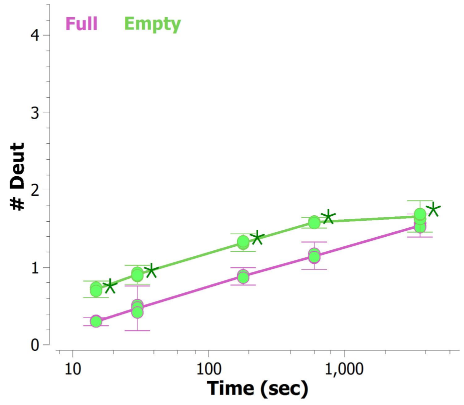

AAV8_VP1 412-418: FQFTYTF (#224)

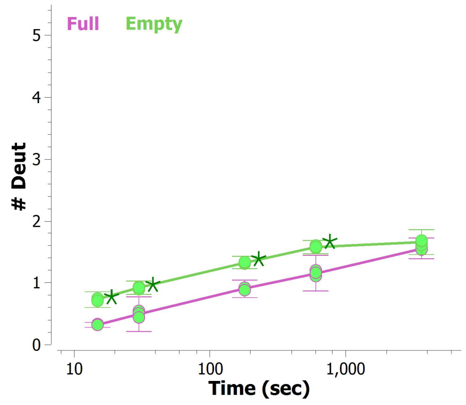

AAV8_VP1 412-419: FQFTYTFE (#225)

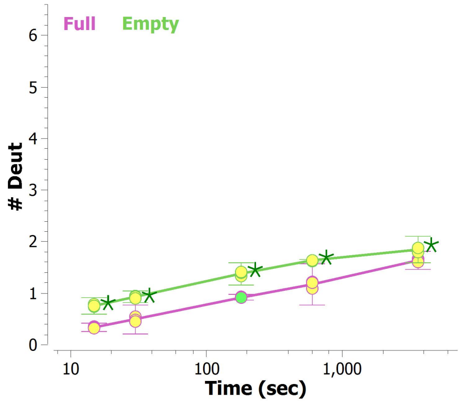

AAV8_VP1 413-417: QFTYT (#226)

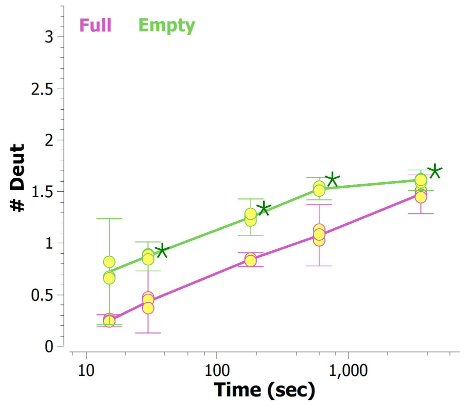

AAV8_VP1 414-418: FTYTF (#227)

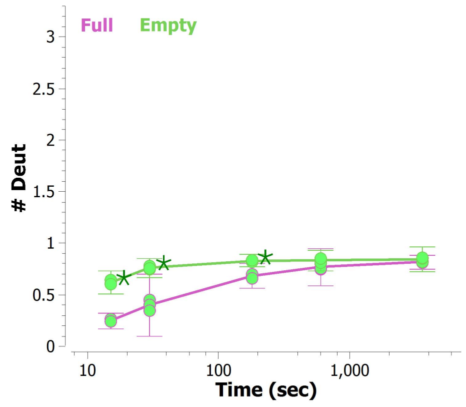

AAV8_VP1 414-433: FTYTFEDVPFHSSYAHSQSL (#228)

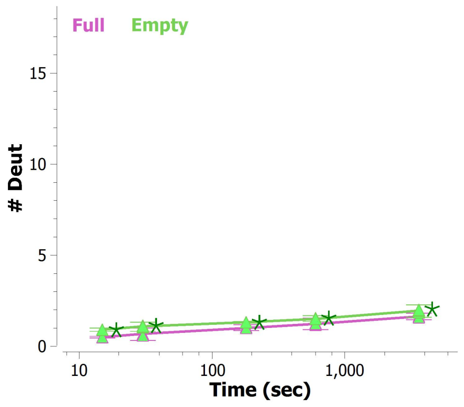

AAV8_VP1 415-433: TYTFEDVPFHSSYAHSQSL (#229)

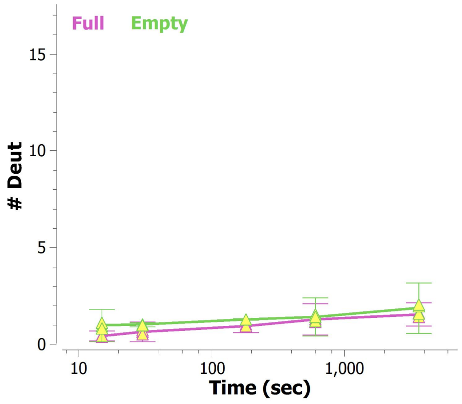

AAV8_VP1 416-433: YTFEDVPFHSSYAHSQSL (#230)

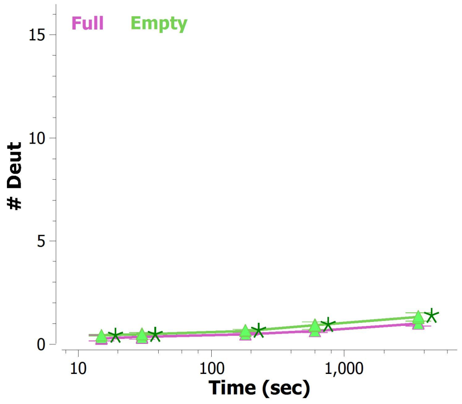

AAV8_VP1 418-426: FEDVPFHSS (#231)

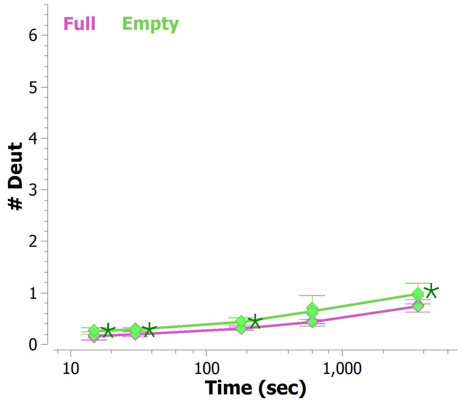

AAV8_VP1 418-427: FEDVPFHSSY (#232)

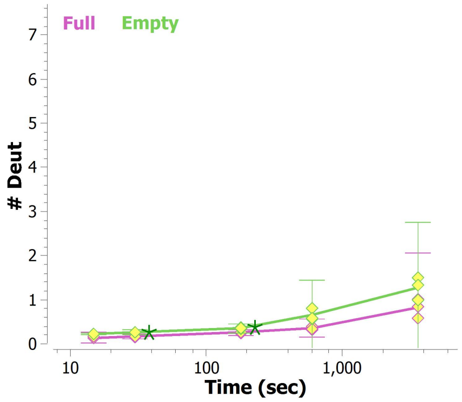

AAV8_VP1 418-428: FEDVPFHSSYA (#233)

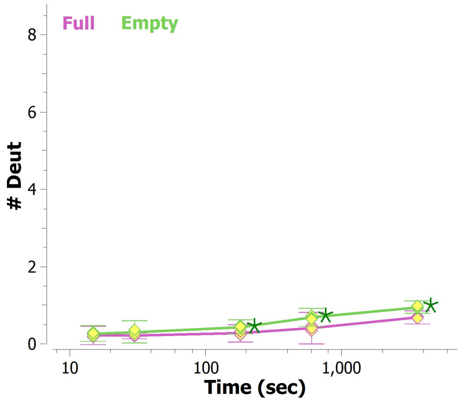

AAV8_VP1 418-430: FEDVPFHSSYAHS (#234)

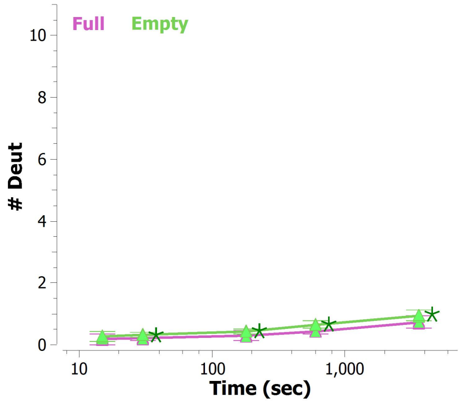

AAV8_VP1 418-433: FEDVPFHSSYAHSQSL (#235)

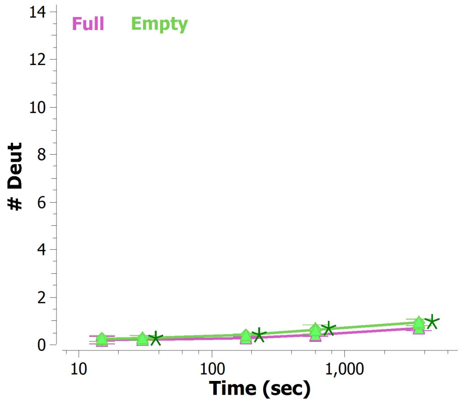

AAV8_VP1 418-434: FEDVPFHSSYAHSQSLD (#236)

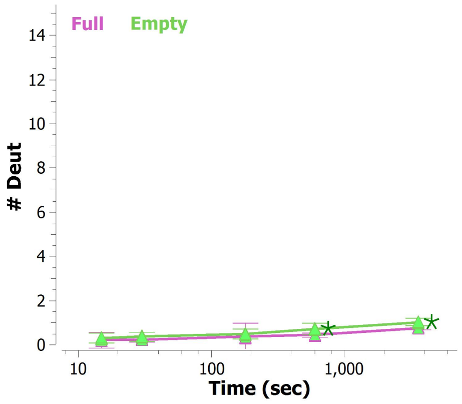

AAV8_VP1 419-430: EDVPFHSSYAHS (#237)

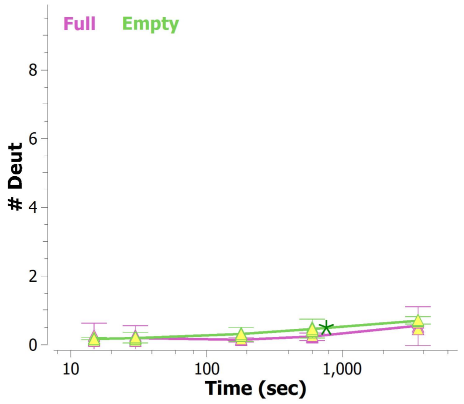

AAV8_VP1 419-433: EDVPFHSSYAHSQSL (#238)

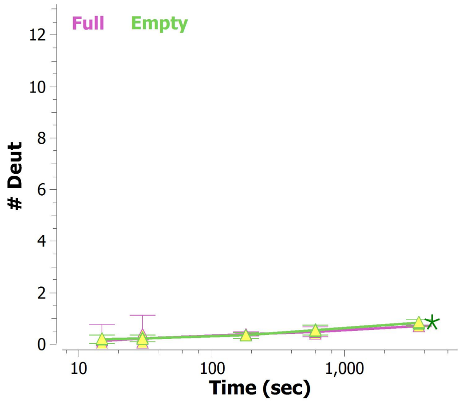

AAV8_VP1 419-443: EDVPFHSSYAHSQSLDRLMNPLIDQ (#239)

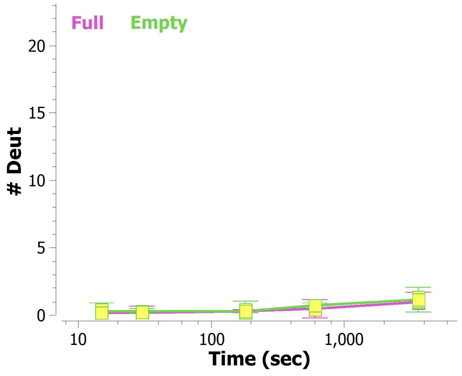

AAV8_VP1 420-433: DVPFHSSYAHSQSL (#240)

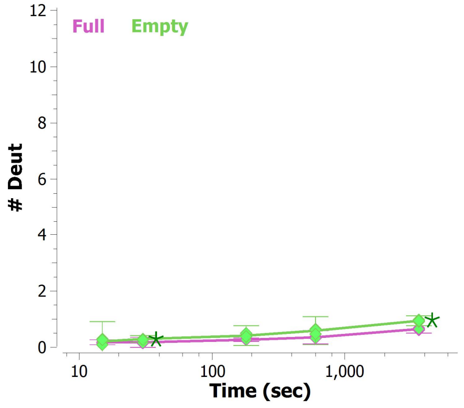

AAV8_VP1 420-443: DVPFHSSYAHSQSLDRLMNPLIDQ (#241)

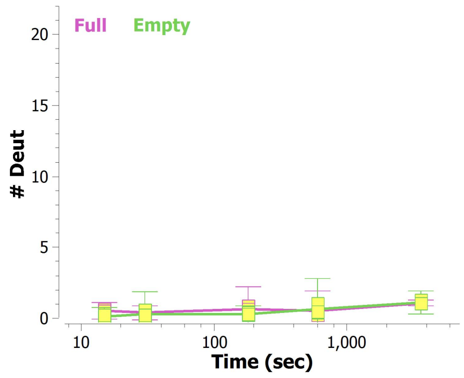

AAV8_VP1 422-433: PFHSSYAHSQSL (#242)

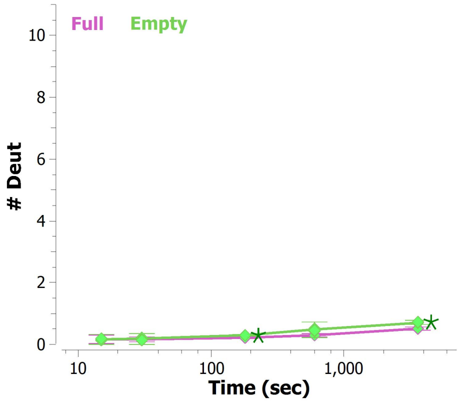

AAV8_VP1 427-433: YAHSQSL (#243)

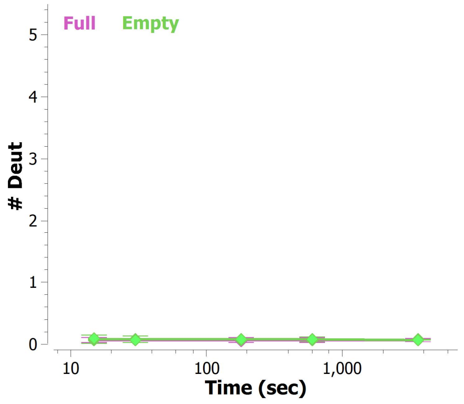

AAV8_VP1 429-433: HSQSL (#244)

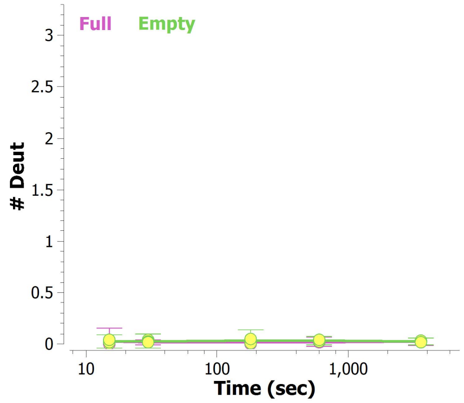

AAV8_VP1 434-442: DRLMNPLID (#245)

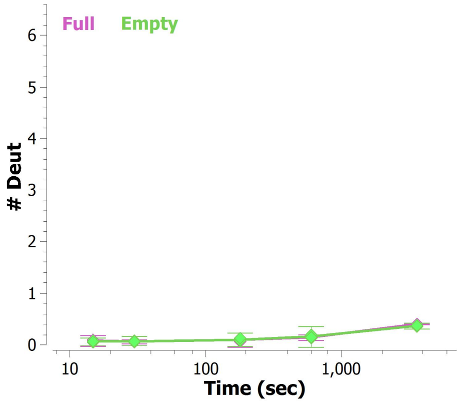

AAV8_VP1 434-443: DRLMNPLIDQ (#246)

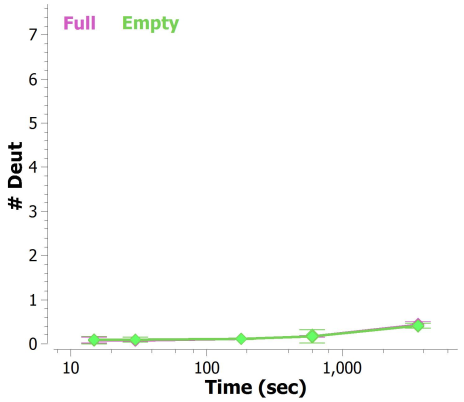

AAV8_VP1 434-444: DRLMNPLIDQY (#247)

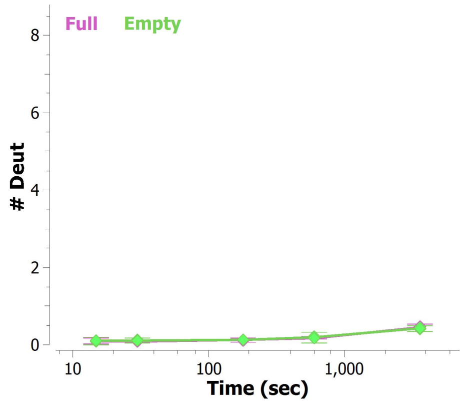

AAV8_VP1 434-445: DRLMNPLIDQYL (#248)

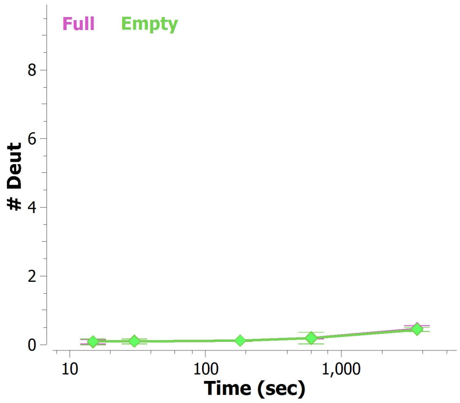

AAV8_VP1 434-447: DRLMNPLIDQYLYY (#249)

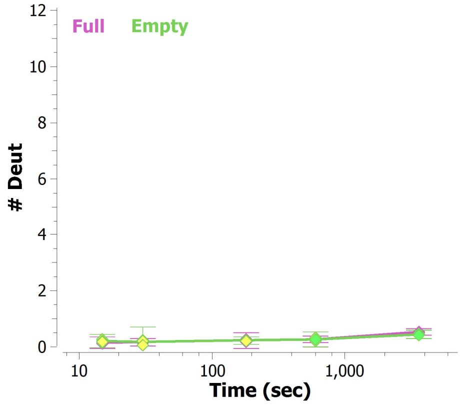

AAV8_VP1 435-443: RLMNPLIDQ (#250)

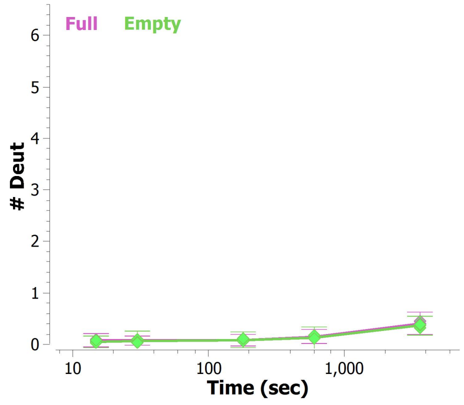

AAV8_VP1 435-445: RLMNPLIDQYL (#251)

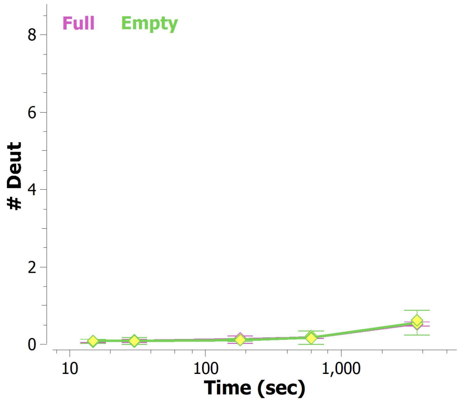

AAV8_VP1 441-445: IDQYL (#252)

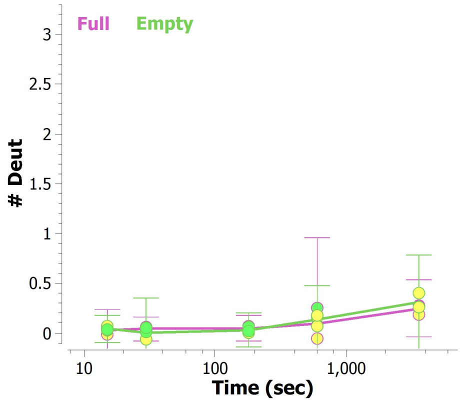

AAV8_VP1 444-447: YLYY (#253)

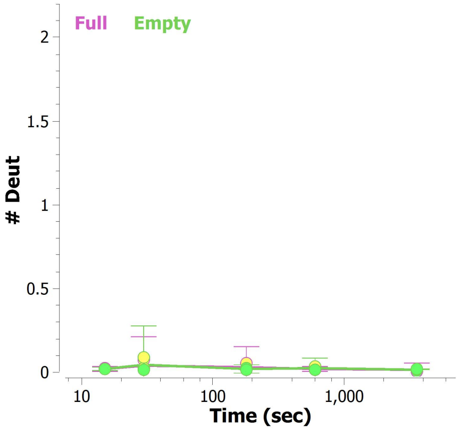

AAV8_VP1 444-460: YLYYLSRTQTTGGTANT (#254)

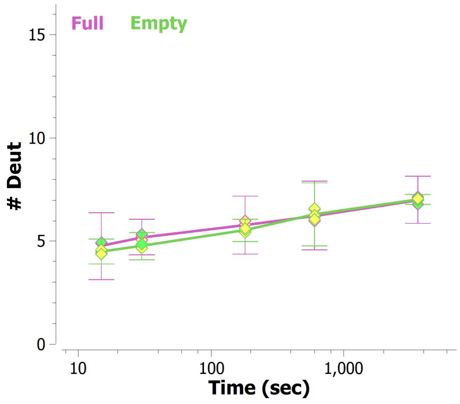

AAV8_VP1 446-451: YYLSRT (#255)

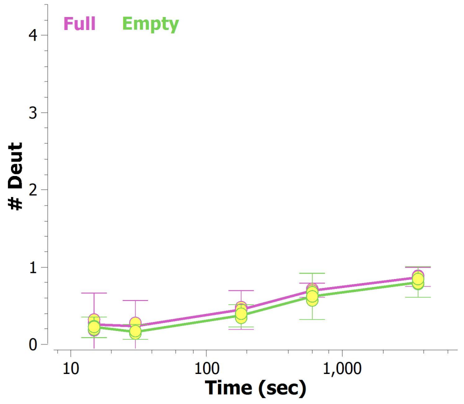

AAV8_VP1 446-453: YYLSRTQT (#256)

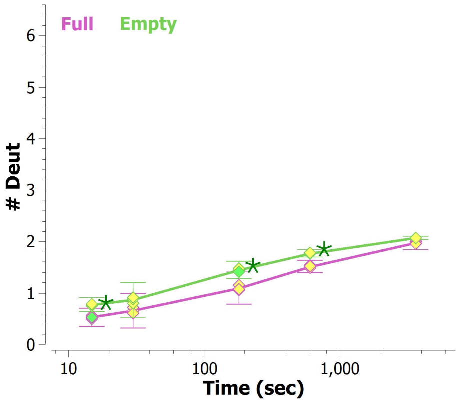

AAV8_VP1 446-454: YYLSRTQTT (#257)

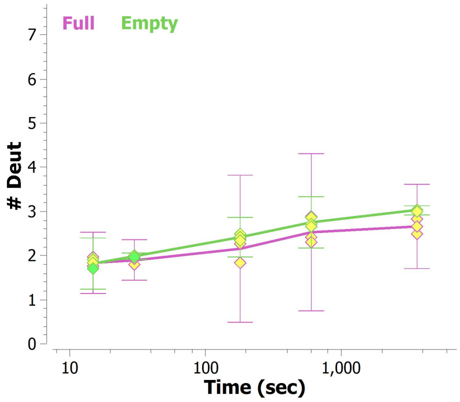

AAV8_VP1 446-455: YYLSRTQTTG (#258)

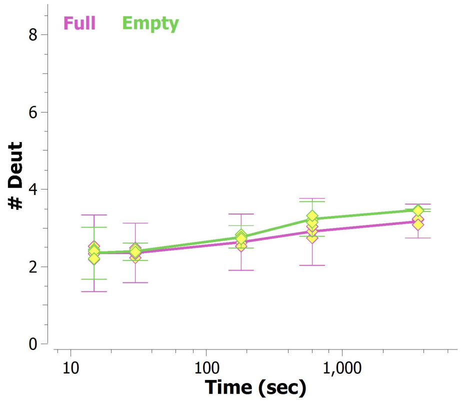

AAV8_VP1 446-458: YYLSRTQTTGGTA (#259)

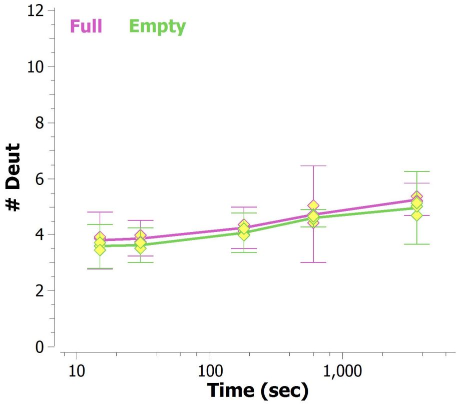

AAV8_VP1 446-460: YYLSRTQTTGGTANT (#260)

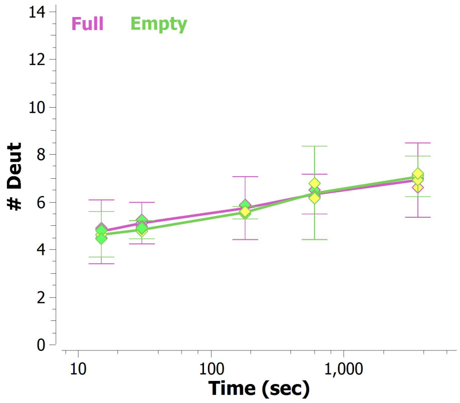

AAV8_VP1 446-462: YYLSRTQTTGGTANTQT (#261)

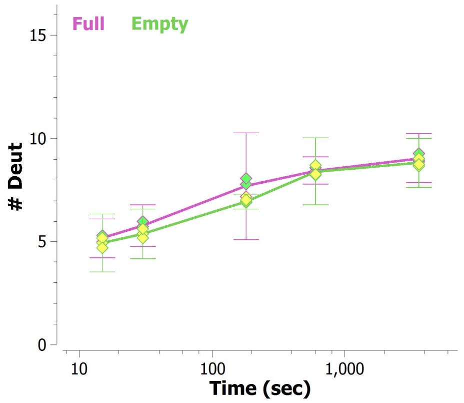

AAV8_VP1 446-463: YYLSRTQTTGGTANTQTL (#262)

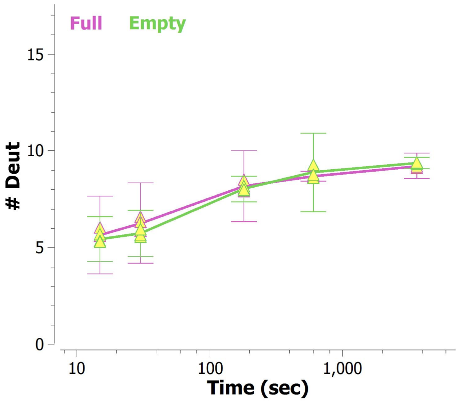

AAV8_VP1 446-464: YYLSRTQTTGGTANTQTLG (#263)

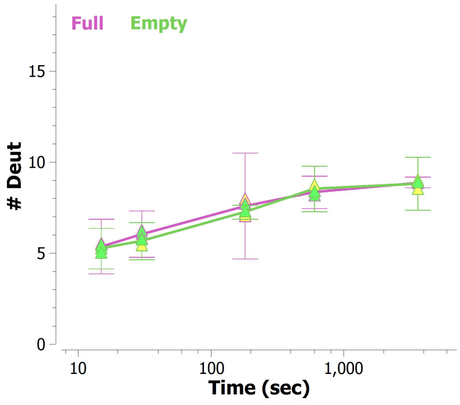

AAV8_VP1 446-465: YYLSRTQTTGGTANTQTLGF (#264)

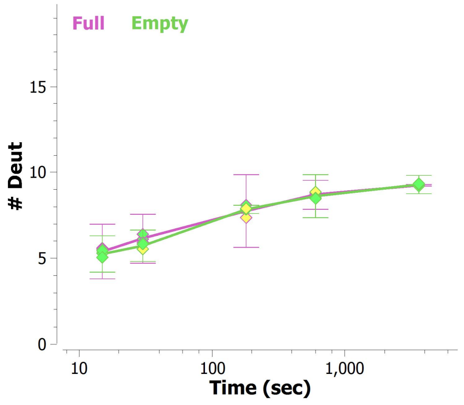

AAV8_VP1 447-460: YLSRTQTTGGTANT (#265)

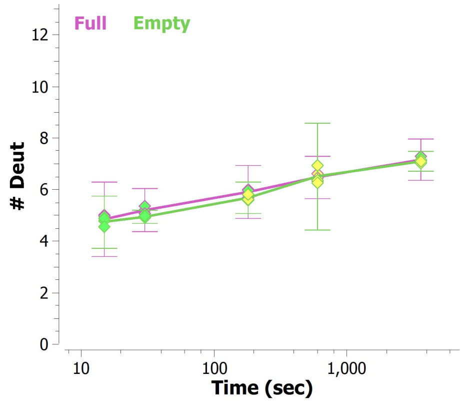

AAV8_VP1 447-464: YLSRTQTTGGTANTQTLG (#266)

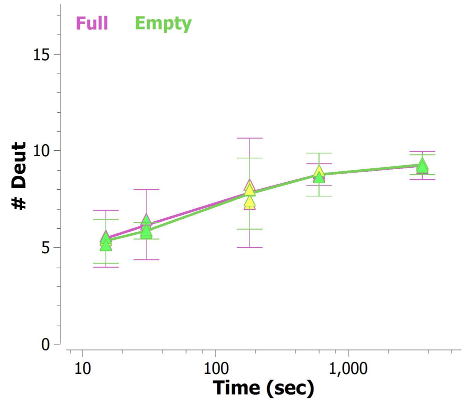

AAV8_VP1 447-465: YLSRTQTTGGTANTQTLGF (#267)

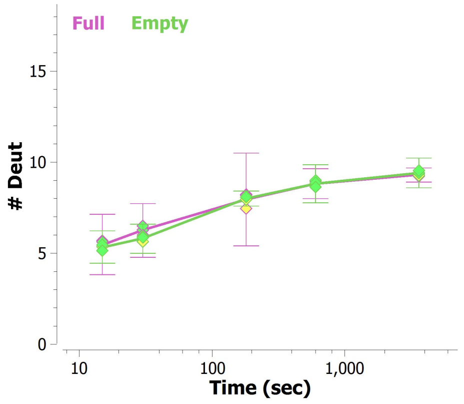

AAV8_VP1 448-458: LSRTQTTGGTA (#268)

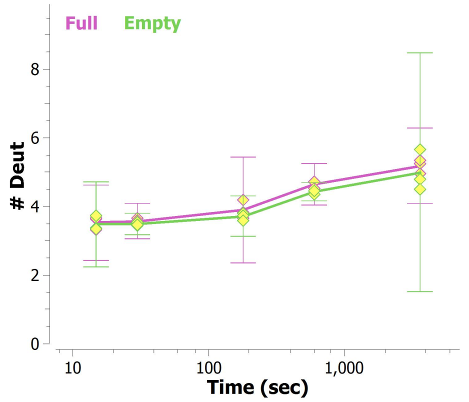

AAV8_VP1 448-460: LSRTQTTGGTANT (#269)

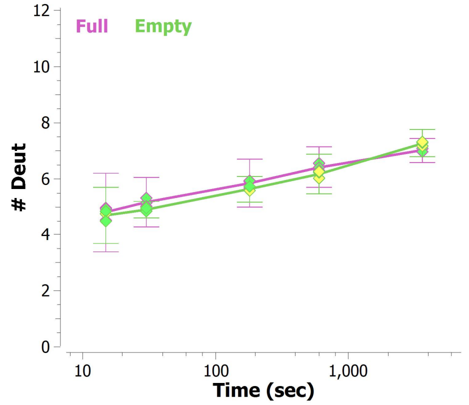

AAV8_VP1 448-462: LSRTQTTGGTANTQT (#270)

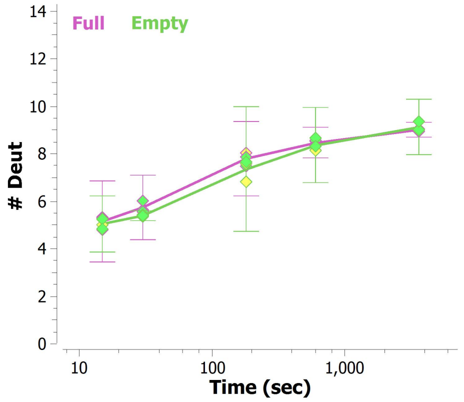

AAV8_VP1 448-464: LSRTQTTGGTANTQTLG (#271)

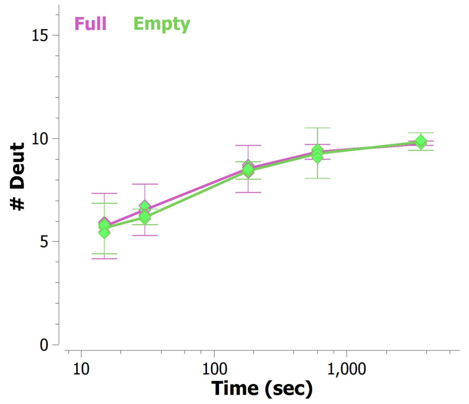

AAV8_VP1 448-465: LSRTQTTGGTANTQTLGF (#272)

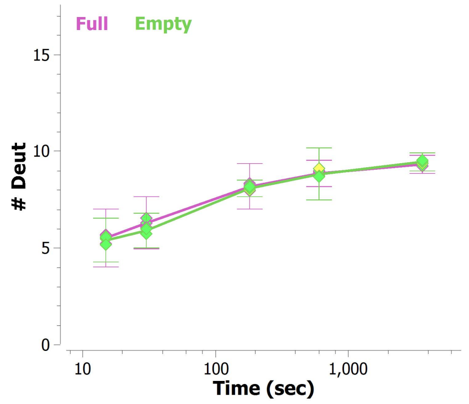

AAV8_VP1 449-460: SRTQTTGGTANT (#273)

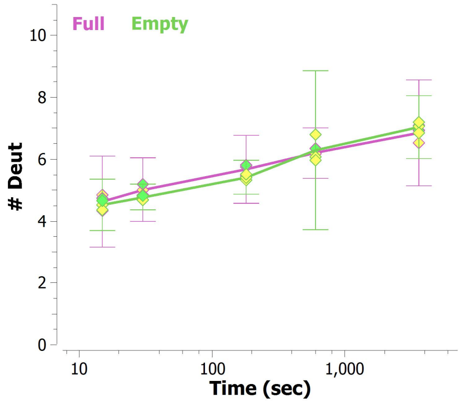

AAV8_VP1 449-464: SRTQTTGGTANTQTLG (#274)

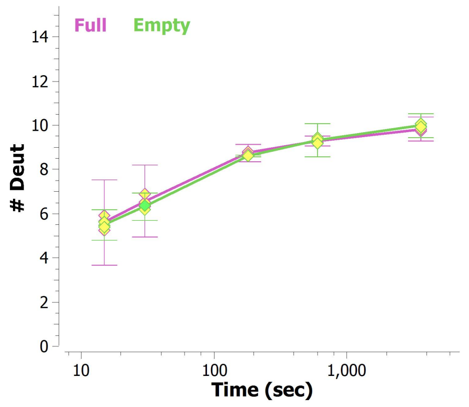

AAV8_VP1 449-465: SRTQTTGGTANTQTLGF (#275)

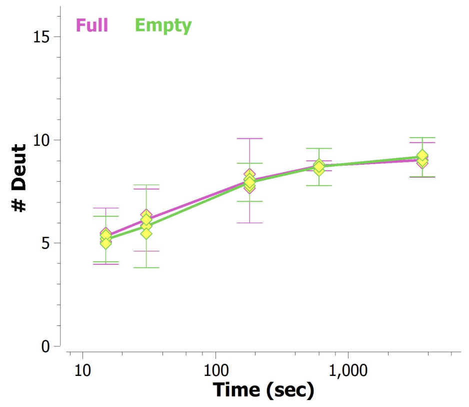

AAV8_VP1 461-465: QTLGF (#276)

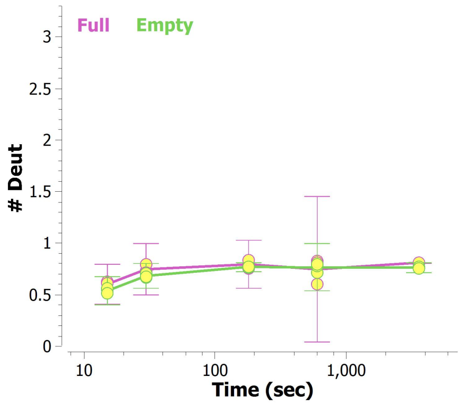

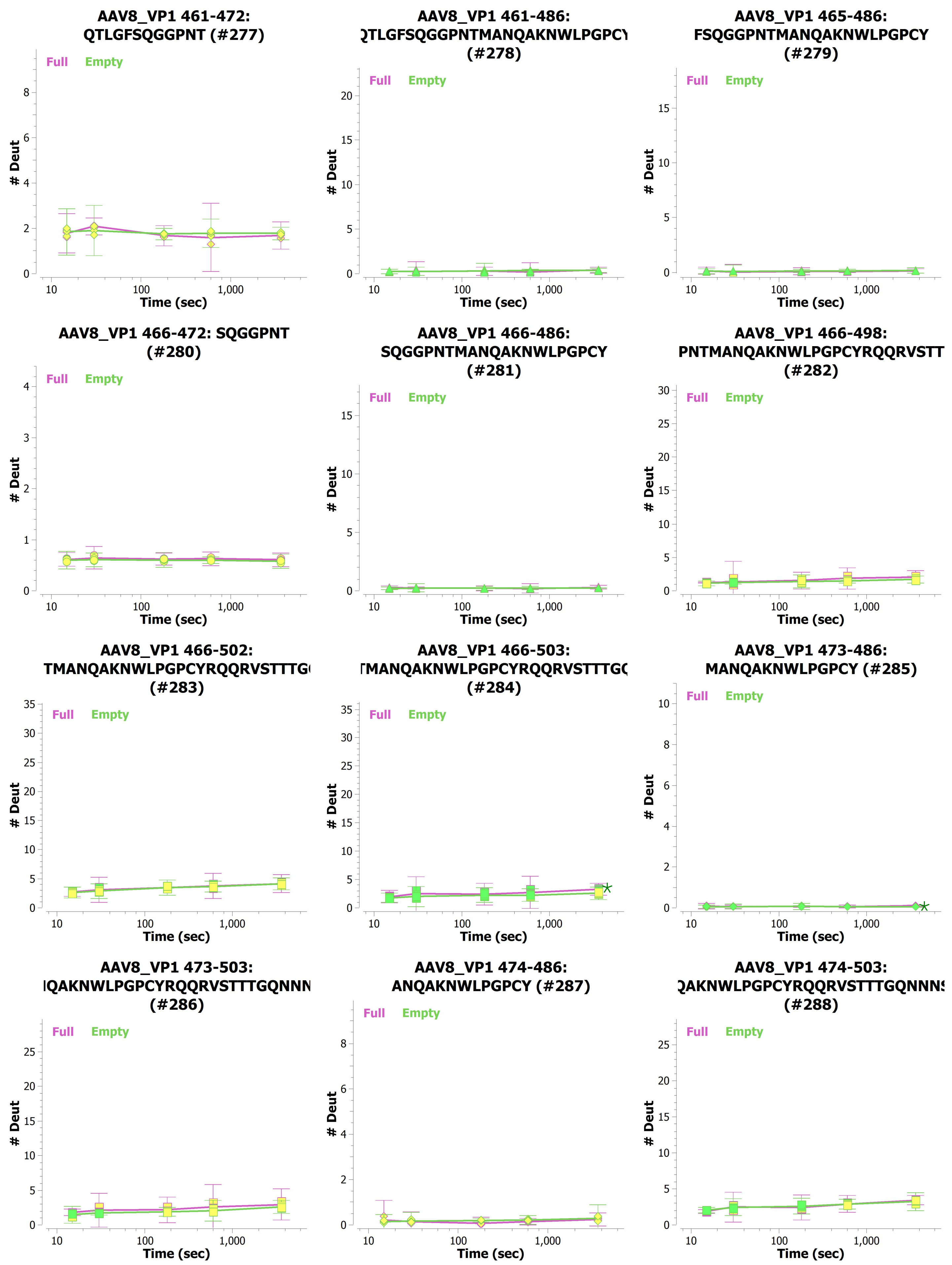
AAV8_VP1 461-472: QTLGFSQGGPNT (#277)
AAV8_VP1 461-486: QTLGFSQGGPNTMANQAKNWLPGPCY (#278)
AAV8_VP1 465-486: FSQGGPNTMANQAKNWLPGPCY (#279)
AAV8_VP1 466-472: SQGGPNT (#280)
AAV8_VP1 466-486: SQGGPNTMANQAKNWLPGPCY (#281)
AAV8_VP1 466-498: PNTMANQAKNWLPGPCYRQQRVSTT (#282)
AAV8_VP1 466-502: TMANQAKNWLPGPCYRQQRVSTTTG (#283)
AAV8_VP1 466-503: TMANQAKNWLPGPCYRQQRVSTTTGQ (#284)
AAV8_VP1 473-486: MANQAKNWLPGPCY (#285)
AAV8_VP1 473-503: IQAKNWLPGPCYRQQRVSTTTGQNNN (#286)
AAV8_VP1 474-486: ANQAKNWLPGPCY (#287)
AAV8_VP1 474-503: QAKNWLPGPCYRQQRVSTTTGQNNNS (#288)
Full
Empty
Deut
Time (sec)
10
100
1,000

AAV8_VP1 475-503: AKNWLPGPCYRQQRVSTTTGQNNNS (#289)

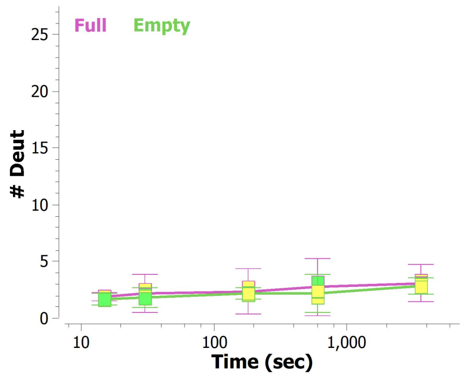

AAV8_VP1 476-503: AKNWLPGPCYRQQRVSTTTGQNNNS (#290)

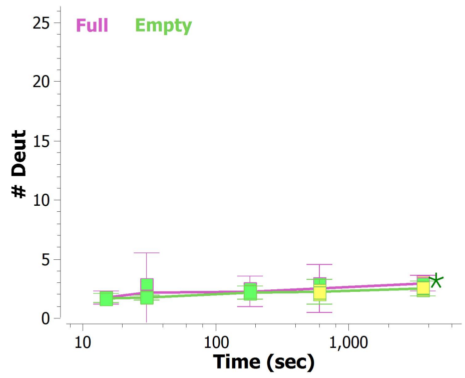

AAV8_VP1 477-486: AKNWLPGPCY (#291)

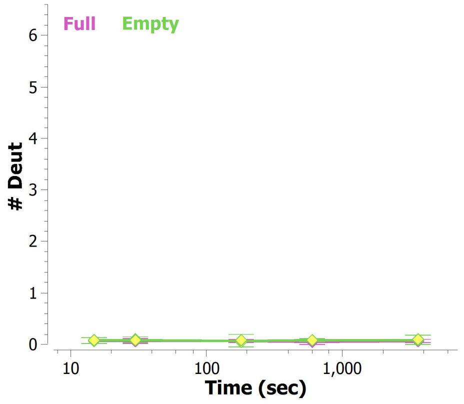

AAV8_VP1 477-503: KNWLPGPCYRQQRVSTTTGQNNNSN (#292)

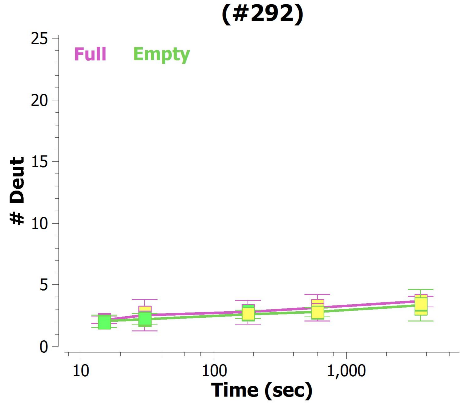

AAV8_VP1 486-503: YRQQRVSTTTGQNNNSNF (#293)

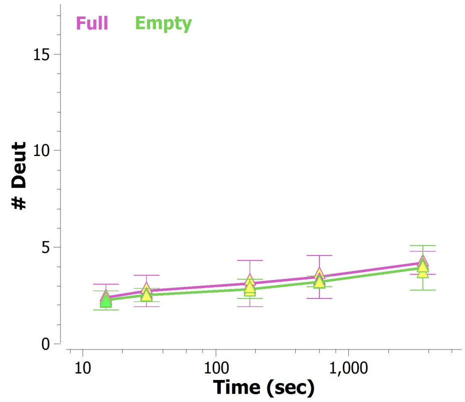

AAV8_VP1 487-498: RQQRVSTTTGQN (#294)

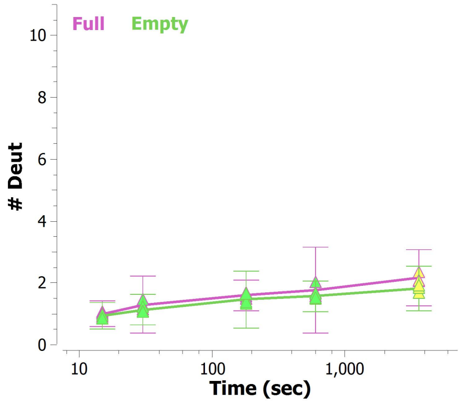

AAV8_VP1 487-503: RQQRVSTTTGQNNNSNF (#295)

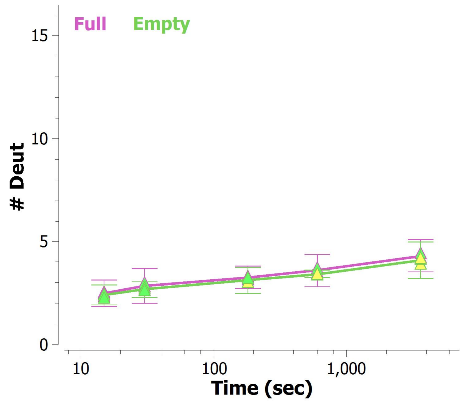

AAV8_VP1 487-504: RQQRVSTTTGQNNNSNFA (#296)

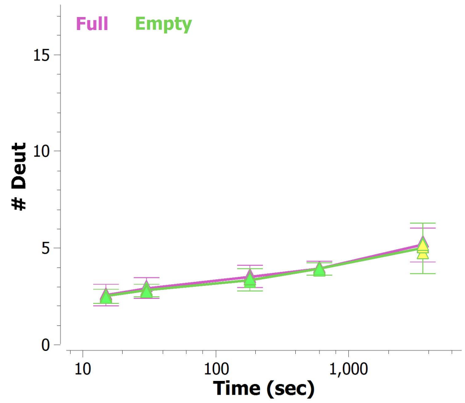

AAV8_VP1 488-517: RVSTTTGQNNNSNFAWTAGTKYHLNG (#297)

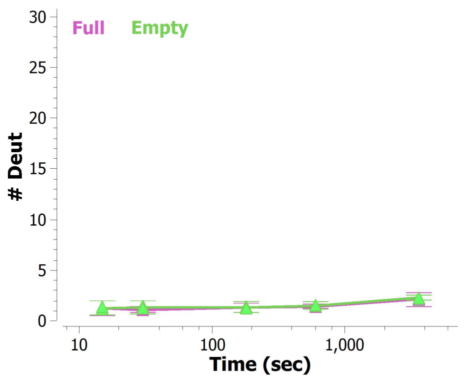

AAV8_VP1 494-503: TTGQNNNSNF (#298)

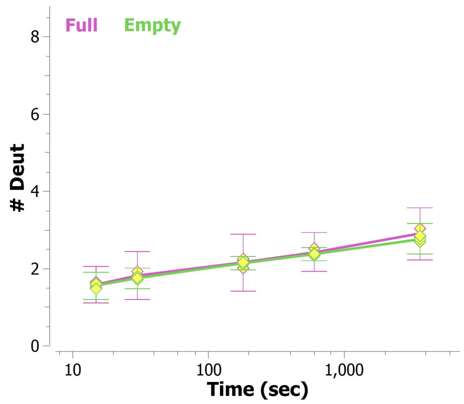

AAV8_VP1 499-503: NNSNF (#299)

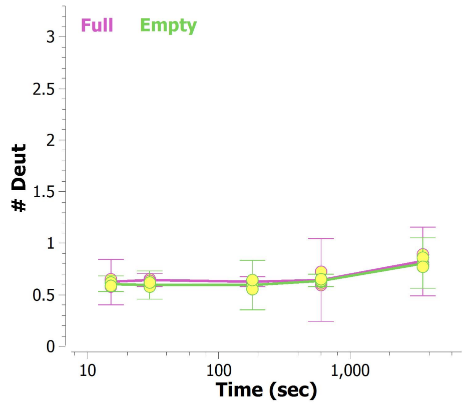

AAV8_VP1 503-525: FAWTAGTKYHLNGRNSLANPGIA (#300)

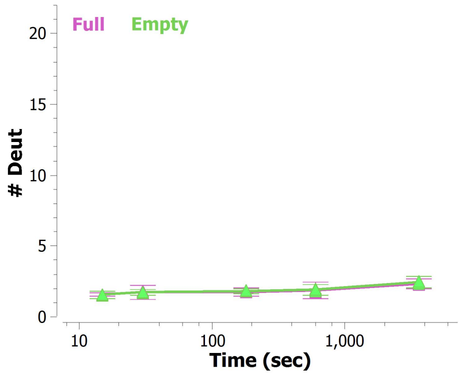

AAV8_VP1 504-511: AWTAGTKY (#301)

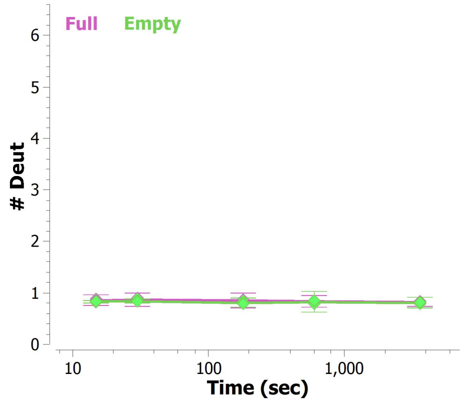

AAV8_VP1 504-513: AWTAGTKYHL (#302)

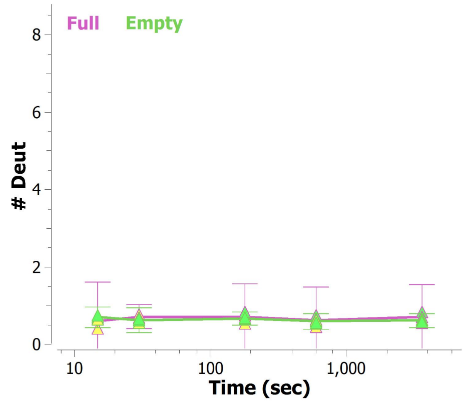

AAV8_VP1 504-517: AWTAGTKYHLNGRN (#303)

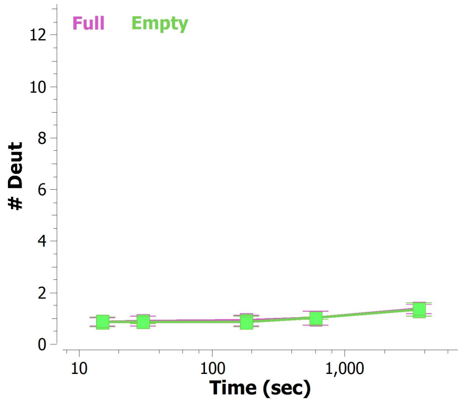

AAV8_VP1 504-525: AWTAGTKYHLNGRNSLANPGIA (#304)

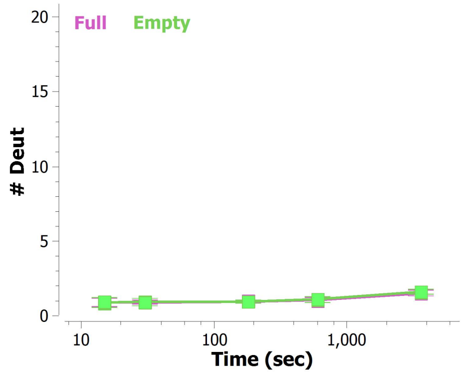

AAV8_VP1 504-526: AWTAGTKYHLNGRNSLANPGIAM (#305)

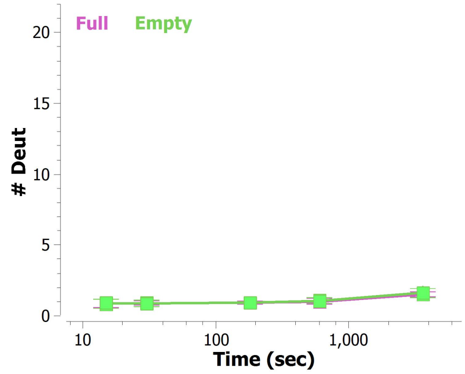

AAV8_VP1 504-533: TAGTKYHLNGRNSLANPGIAMATHK (#306)

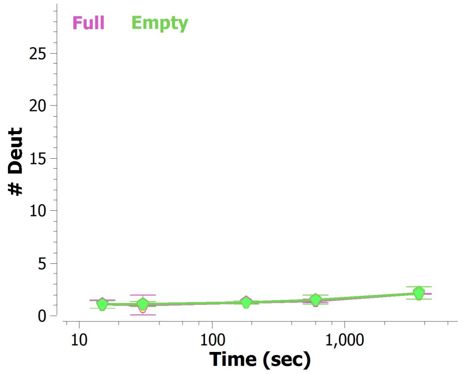

AAV8_VP1 504-534: AGTKYHLNGRNSLANPGIAMATHKD (#307)

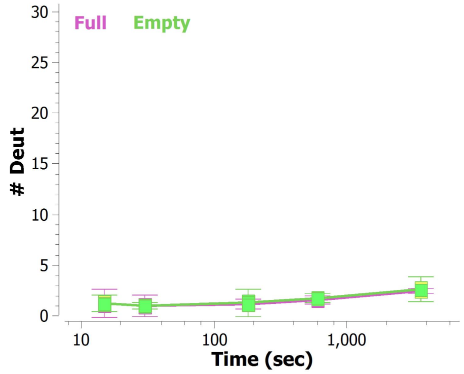

AAV8_VP1 504-543: YHLNGRNSLANPGIAMATHKDDEER (#308)

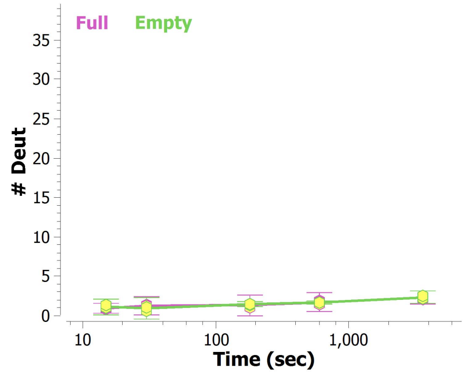

AAV8_VP1 505-517: WTAGTKYHLNGRN (#309)

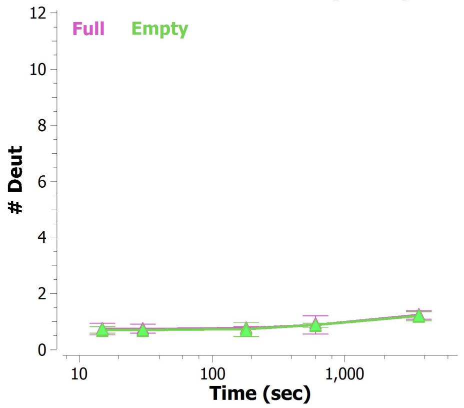

AAV8_VP1 505-525: WTAGTKYHLNGRNSLANPGIA (#310)

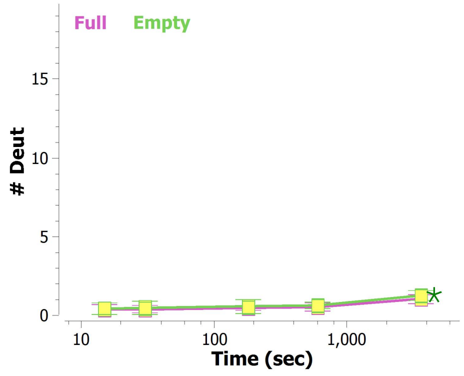

AAV8_VP1 505-526: WTAGTKYHLNGRNSLANPGIAM (#311)

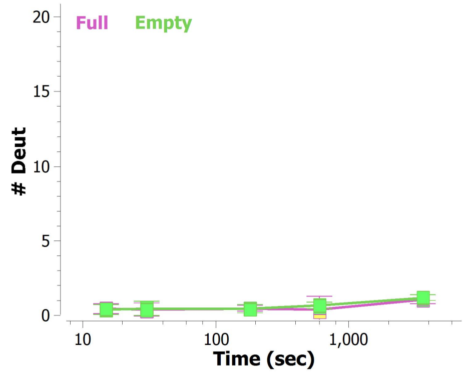

AAV8_VP1 507-517: AGTKYHLNGRN (#312)

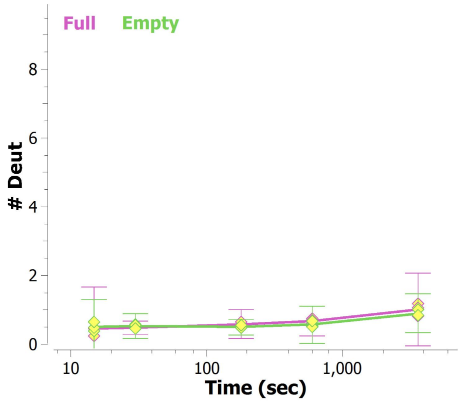

AAV8_VP1 512-525: HLNGRNSLANPGIA (#313)

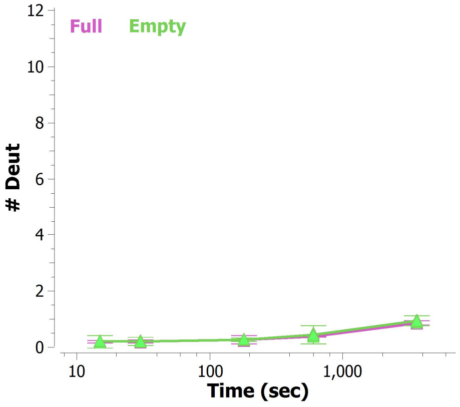

AAV8_VP1 512-526: HLNGRNSLANPGIAM (#314)

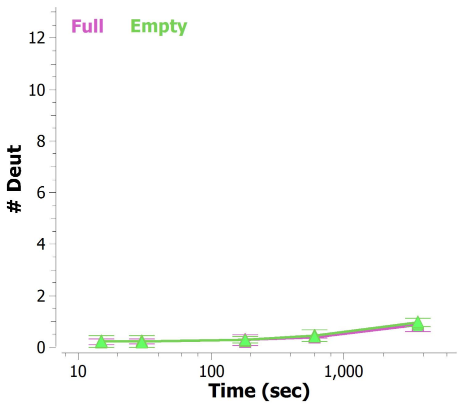

AAV8_VP1 518-525: SLANPGIA (#315)

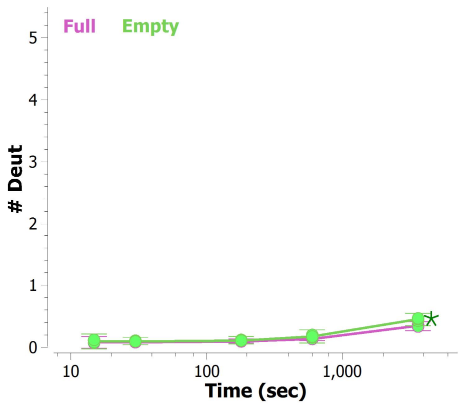

AAV8_VP1 518-526: SLANPGIAM (#316)

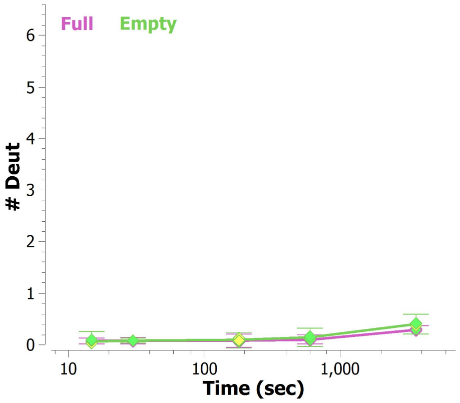

AAV8_VP1 518-533: SLANPGIAMATHKDDE (#317)

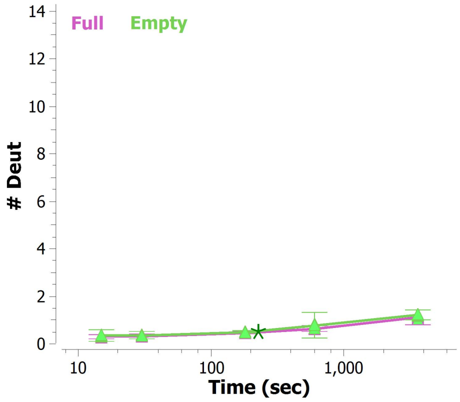

AAV8_VP1 518-534: SLANPGIAMATHKDDEE (#318)

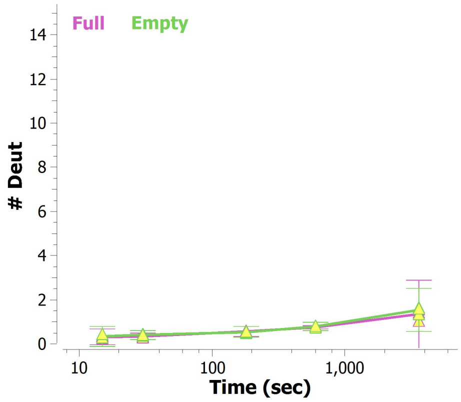

AAV8_VP1 526-533: MATHKDDE (#319)

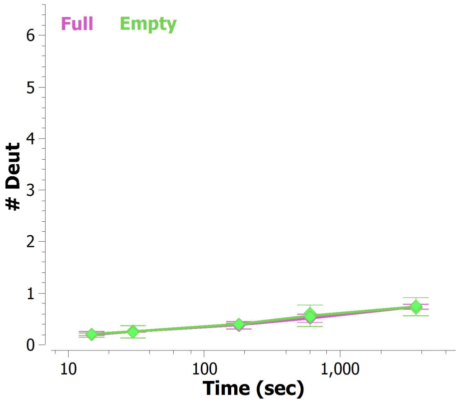

AAV8_VP1 526-534: MATHKDDEE (#320)

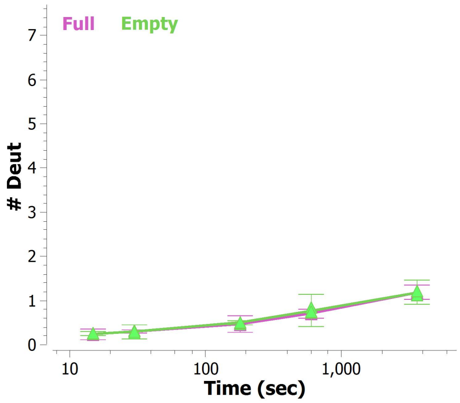

AAV8_VP1 526-536: MATHKDDEERF (#321)

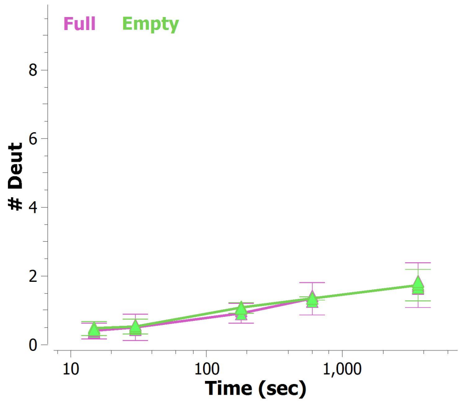

AAV8_VP1 526-540: MATHKDDEERFFPSN (#322)

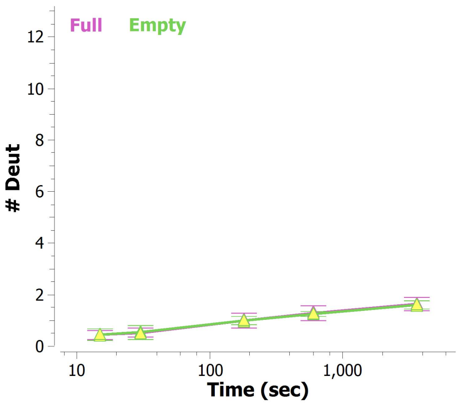

AAV8_VP1 526-541: MATHKDDEERFFPSNG (#323)

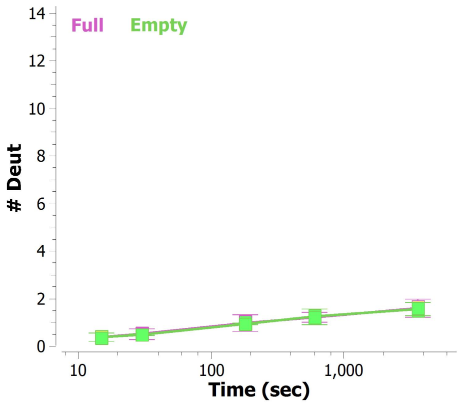

AAV8_VP1 526-543: MATHKDDEERFFPSNGIL (#324)

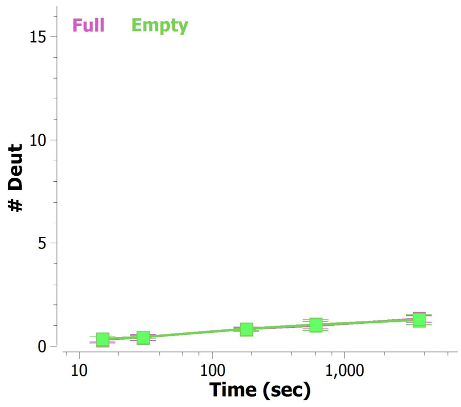

AAV8_VP1 526-544: MATHKDDEERFFPSNGILI (#325)

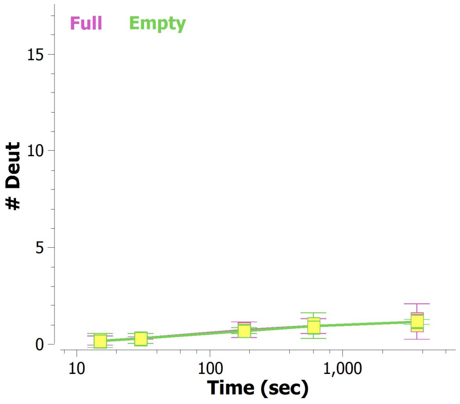

AAV8_VP1 526-545: MATHKDDEERFFPSNGILIF (#326)

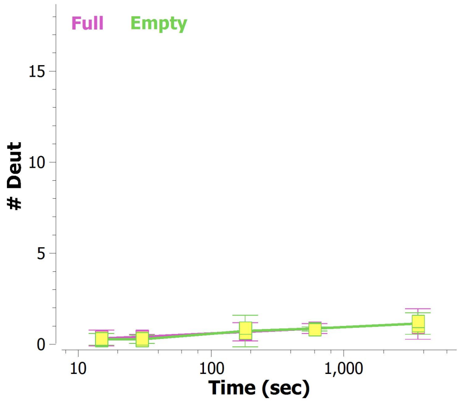

AAV8_VP1 526-556: THKDDEERFFPSNGILIFGKQNAARDN (#327)

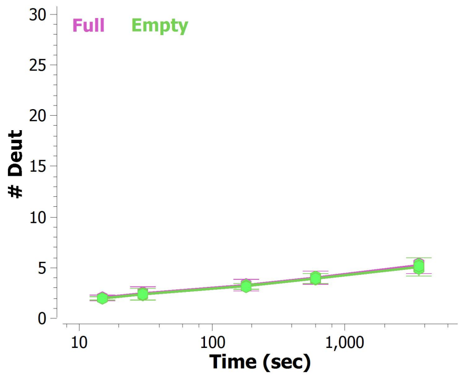

AAV8_VP1 527-540: ATHKDDEERFFPSN (#328)

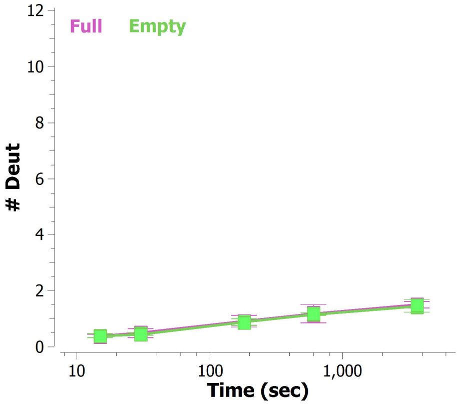

AAV8_VP1 527-541: ATHKDDEERFFPSNG (#329)

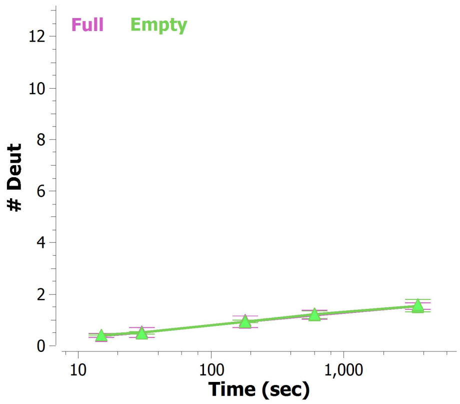

AAV8_VP1 527-543: ATHKDDEERFFPSNGIL (#330)

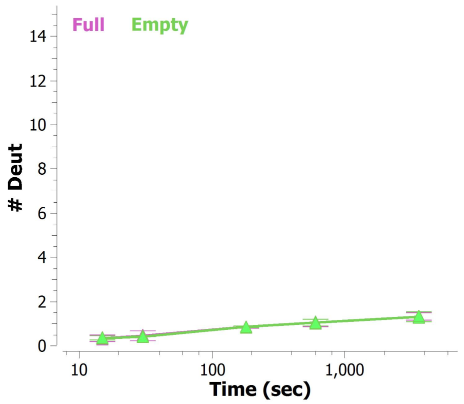

AAV8_VP1 527-556: HKDDEERFFPSNGILIFGKQNAARDN (#331)

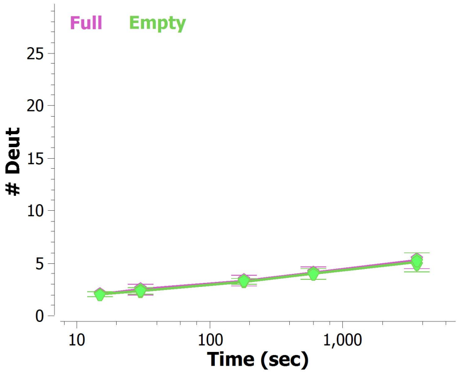

AAV8_VP1 529-543: HKDDEERFFPSNGIL (#332)

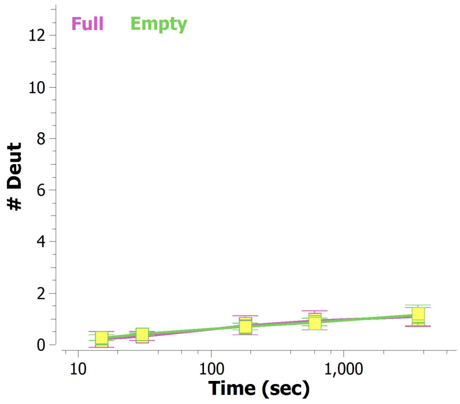

AAV8_VP1 534-540: ERFFPSN (#333)

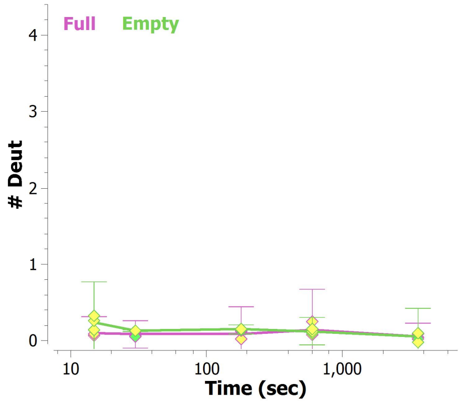

AAV8_VP1 534-541: ERFFPSNG (#334)

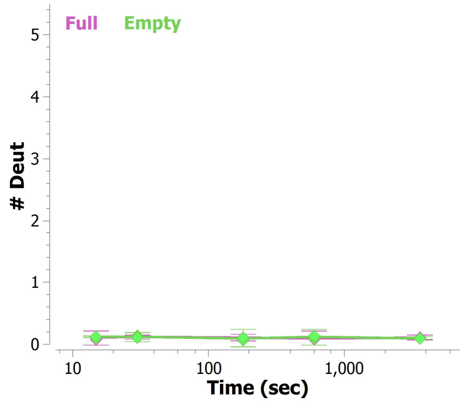

AAV8_VP1 534-543: ERFFPSNGIL (#335)

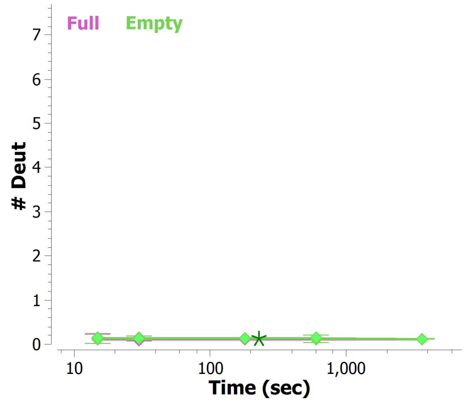

AAV8_VP1 534-544: ERFFPSNGILI (#336)

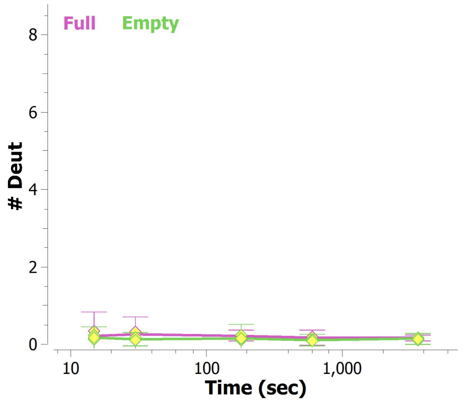

AAV8_VP1 534-556: ERFFPSNGILIFGKQNAARDNAD (#337)

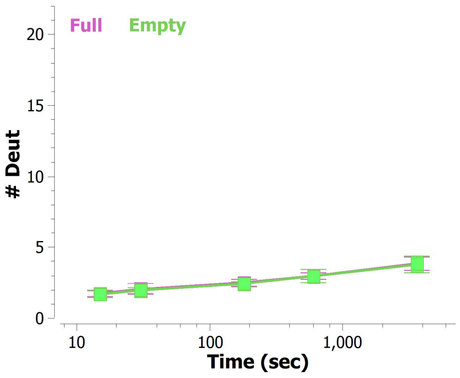

AAV8_VP1 535-541: RFFPSNG (#338)

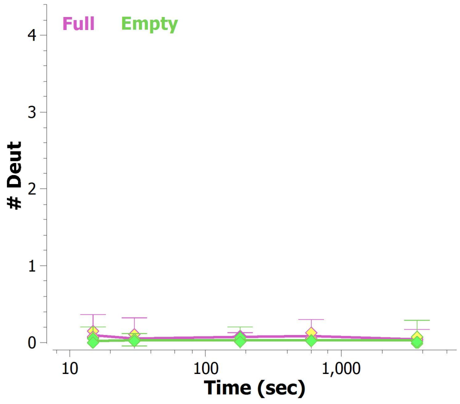

AAV8_VP1 535-543: RFFPSNGIL (#339)

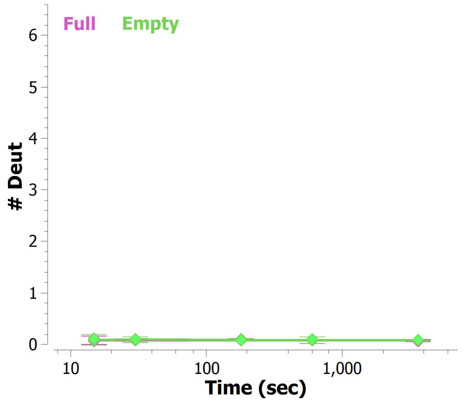

AAV8_VP1 535-556: RFFPSNGILIFGKQNAARDNAD (#340)

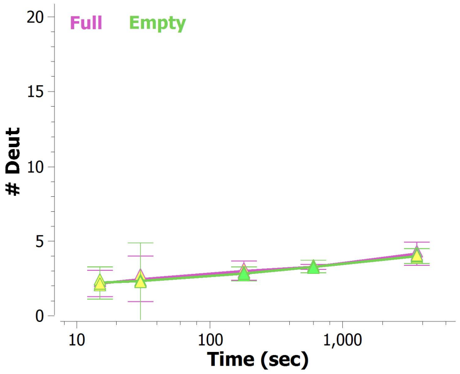

AAV8_VP1 537-543: FPSNGIL (#341)

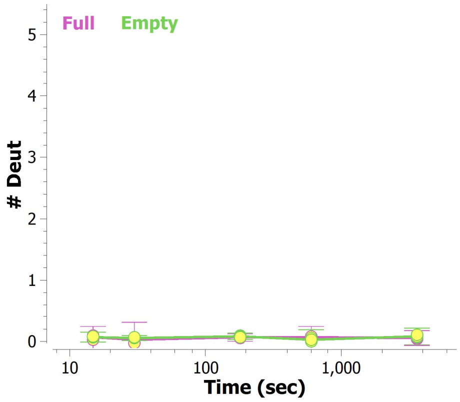

AAV8_VP1 537-556: FPSNGILIFGKQNAARDNAD (#342)

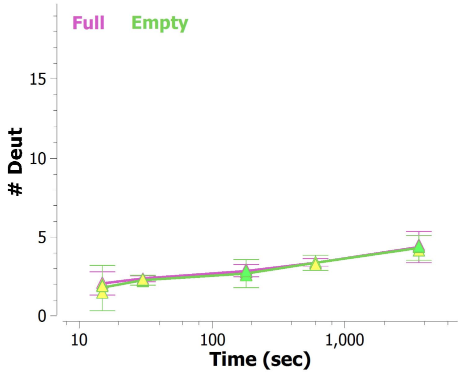

AAV8_VP1 538-543: PSNGIL (#343)

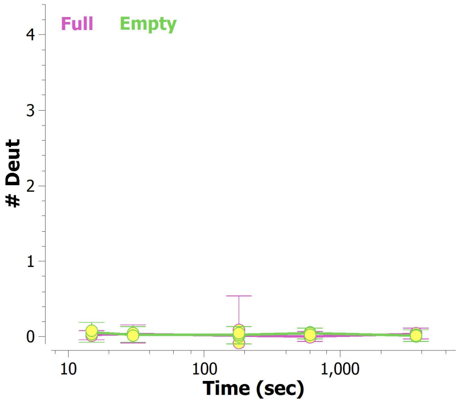

AAV8_VP1 541-556: GILIFGKQNAARDNAD (#344)

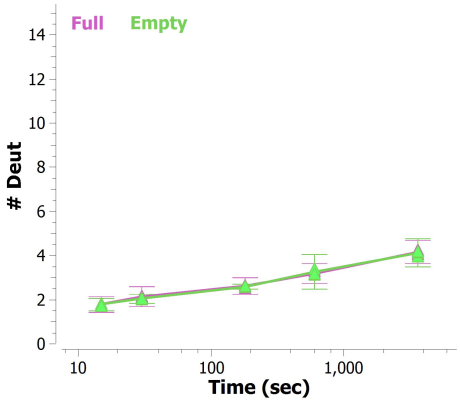

AAV8_VP1 541-559: GILIFGKQNAARDNADYSD (#345)

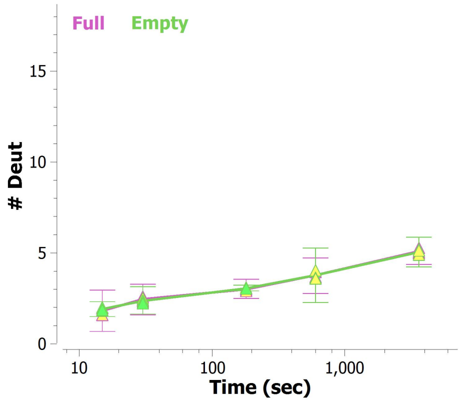

AAV8_VP1 542-556: ILIFGKQNAARDNAD (#346)

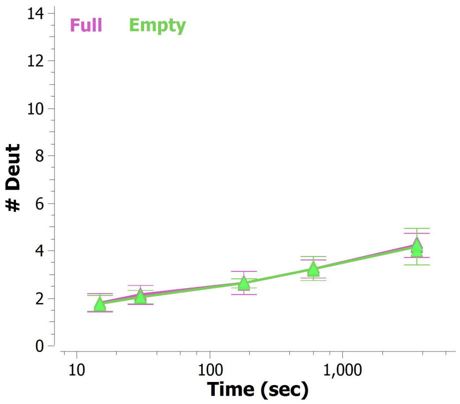

AAV8_VP1 542-559: ILIFGKQNAARDNADYSD (#347)

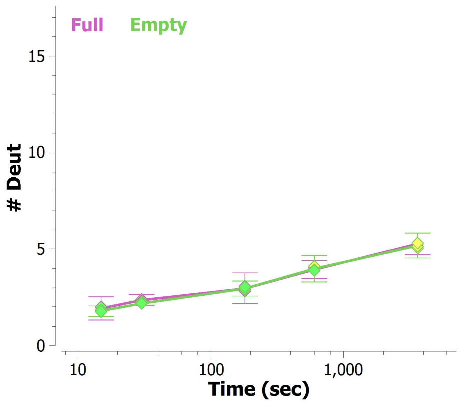

AAV8_VP1 544-548: IFGKQ (#348)

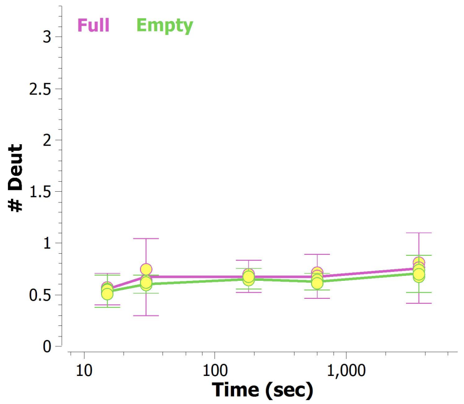

AAV8_VP1 544-550: IFGKQNA (#349)

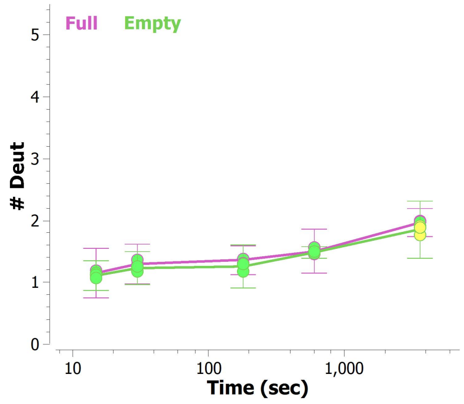

AAV8_VP1 544-552: IFGKQNAAR (#350)

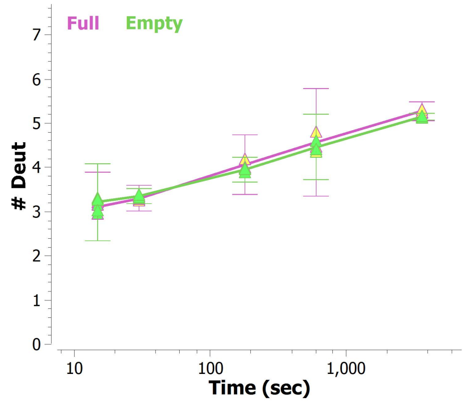

AAV8_VP1 544-553: IFGKQNAARD (#351)

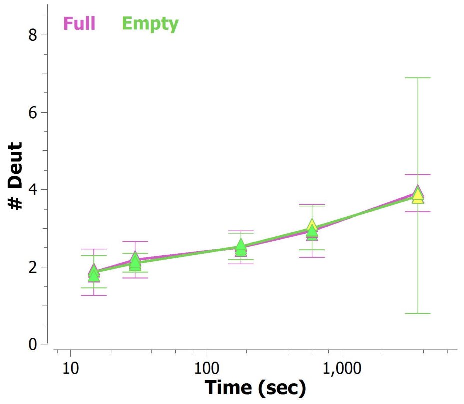

AAV8_VP1 544-556: IFGKQNAARDNAD (#352)

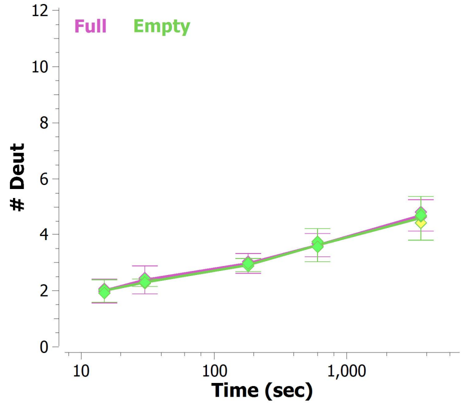

AAV8_VP1 544-558: IFGKQNAARDNADYS (#353)

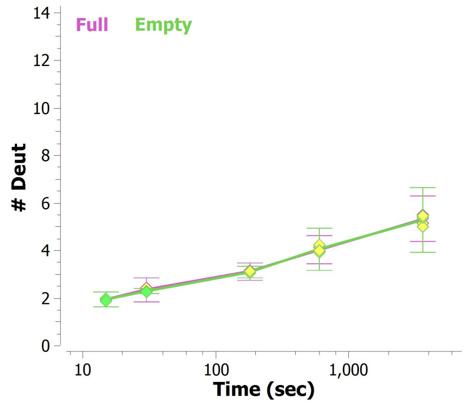

AAV8_VP1 544-559: IFGKQNAARDNADYSD (#354)

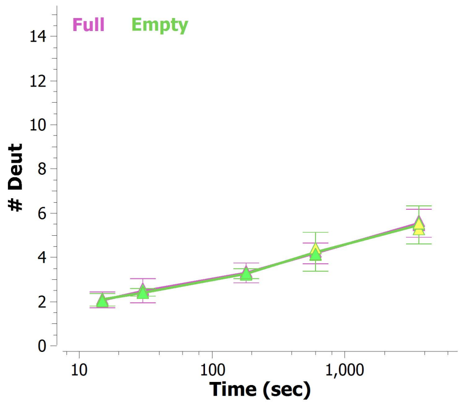

AAV8_VP1 544-561: IFGKQNAARDNADYSDVM (#355)

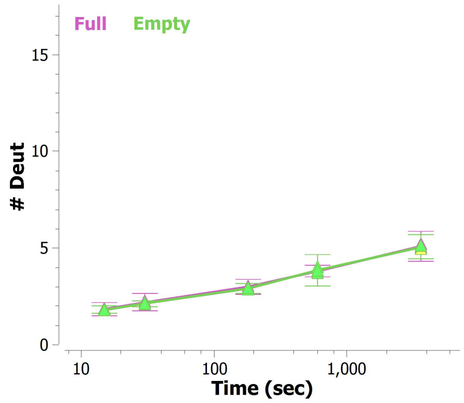

AAV8_VP1 544-562: IFGKQNAARDNADYSDVML (#356)

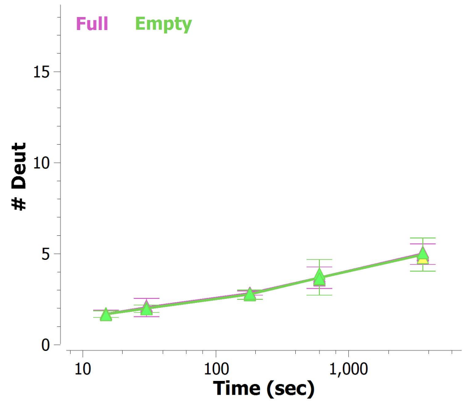

AAV8_VP1 545-556: FGKQNAARDNAD (#357)

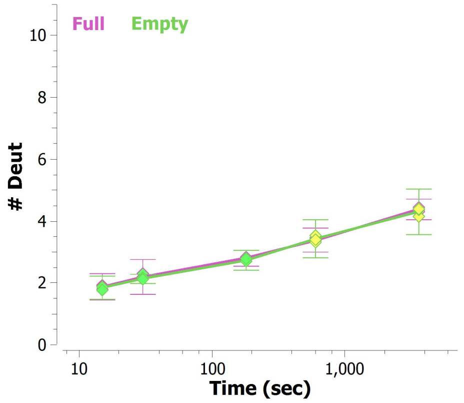

AAV8_VP1 546-556: GKQNAARDNAD (#358)

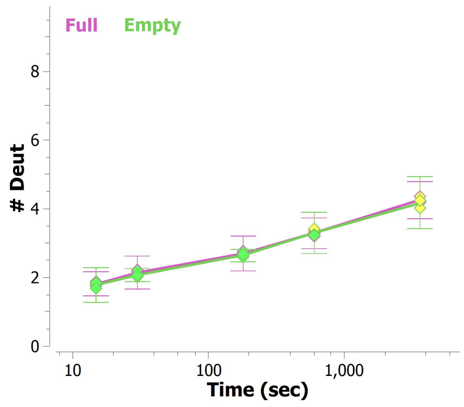

AAV8_VP1 546-559: GKQNAARDNADYSD (#359)

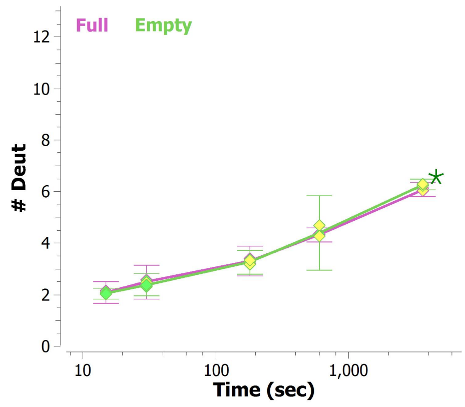

AAV8_VP1 554-559: NADYSD (#360)

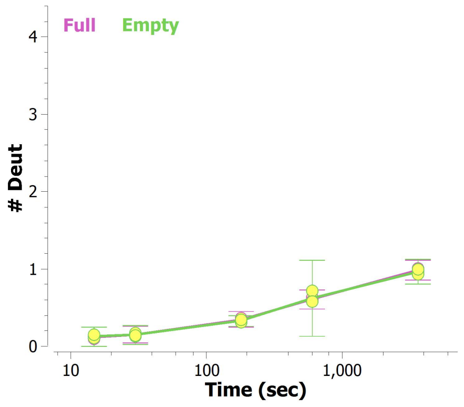

AAV8_VP1 557-560: YSDV (#361)

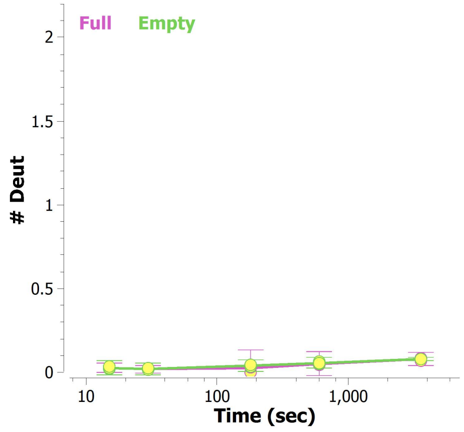

AAV8_VP1 557-561: YSDVM (#362)

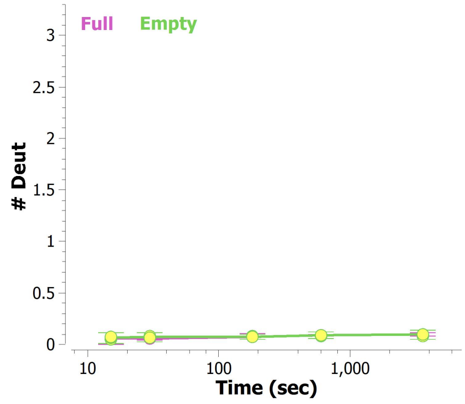

AAV8_VP1 557-565: YSDVMLTSE (#363)

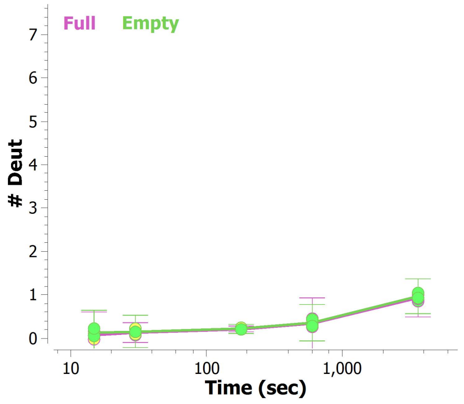

AAV8_VP1 559-565: DVMLTSE (#364)

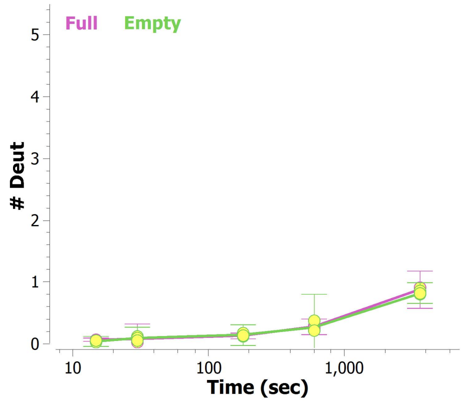

AAV8_VP1 560-565: VMLTSE (#365)

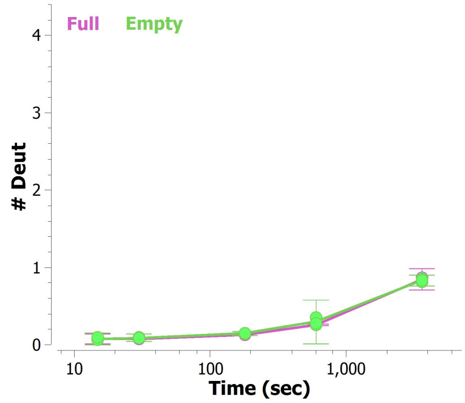

AAV8_VP1 560-566: VMLTSEE (#366)

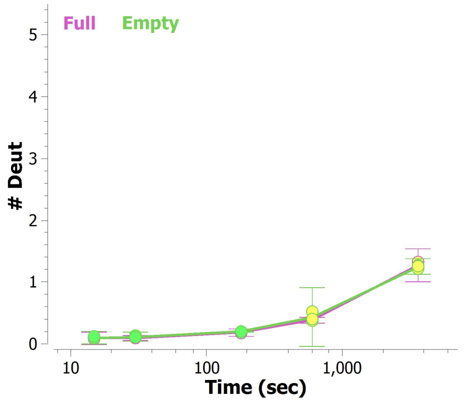

AAV8_VP1 560-567: VMLTSEEE (#367)

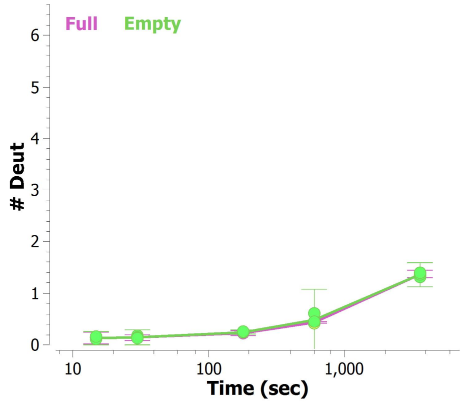

AAV8_VP1 560-576: VMLTSEEEIKTTNPVAT (#368)

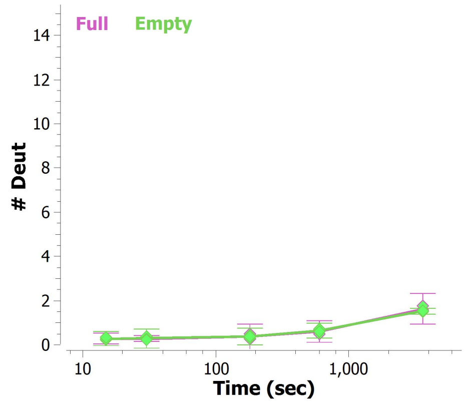

AAV8_VP1 560-578: VMLTSEEEIKTTNPVATEE (#369)

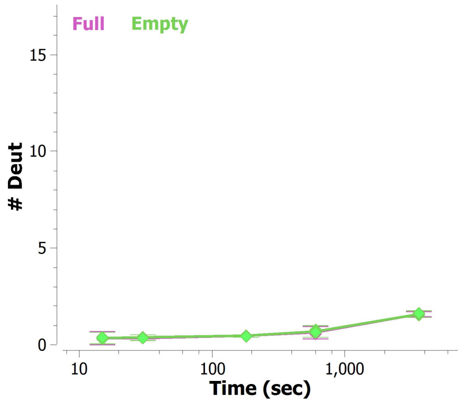

AAV8_VP1 561-565: MLTSE (#370)

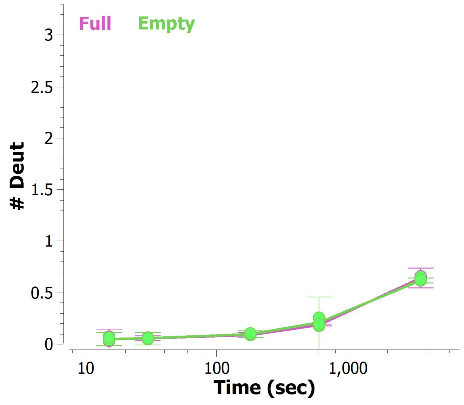

AAV8_VP1 561-567: MLTSEEE (#371)

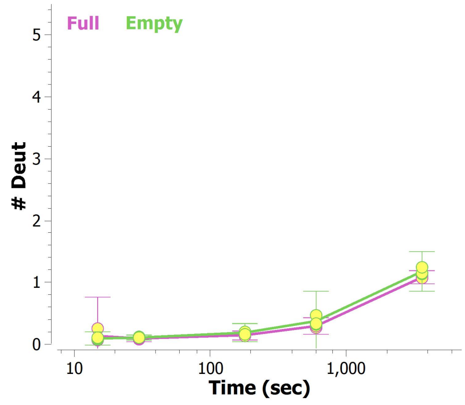

AAV8_VP1 561-578: MLTSEEEIKTTNPVATEE (#372)

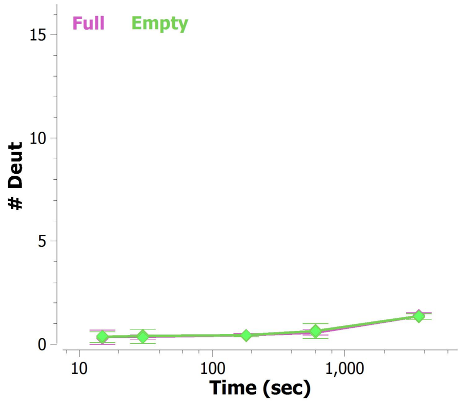

AAV8_VP1 562-566: LTSEE (#373)

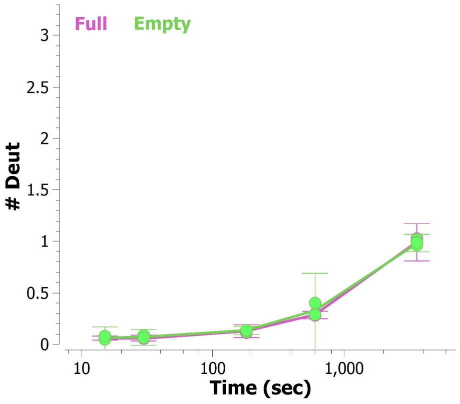

AAV8_VP1 562-567: LTSEEE (#374)

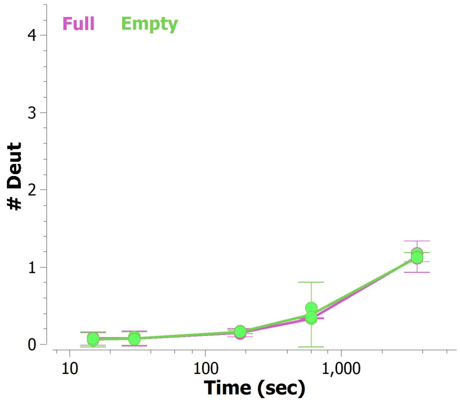

AAV8_VP1 562-575: LTSEEEIKTTNPVA (#375)

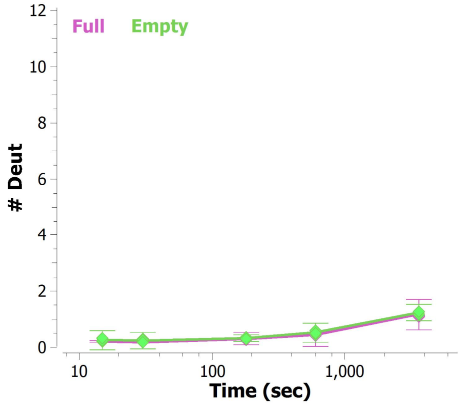

AAV8_VP1 562-576: LTSEEEIKTTNPVAT (#376)

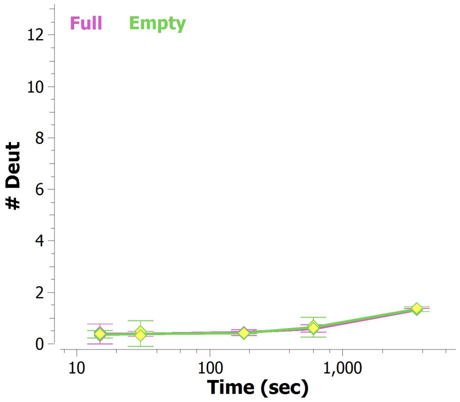

AAV8_VP1 562-578: LTSEEEIKTTNPVATEE (#377)

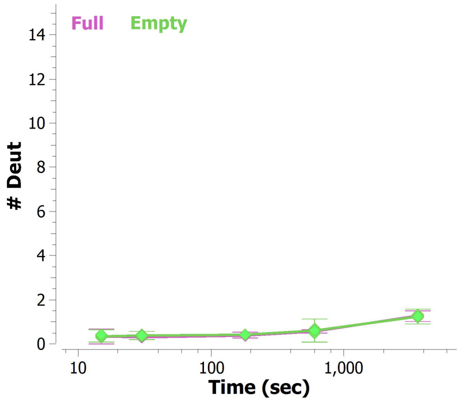

AAV8_VP1 563-575: TSEEEIKTTNPVA (#378)

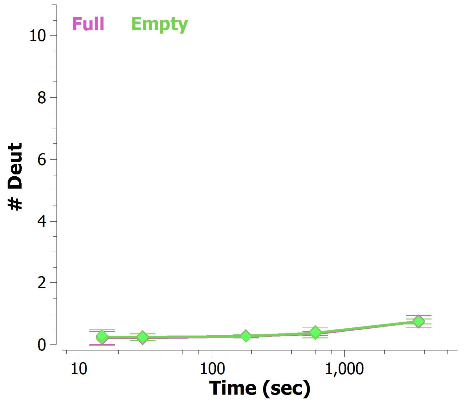

AAV8_VP1 563-576: TSEEEIKTTNPVAT (#379)

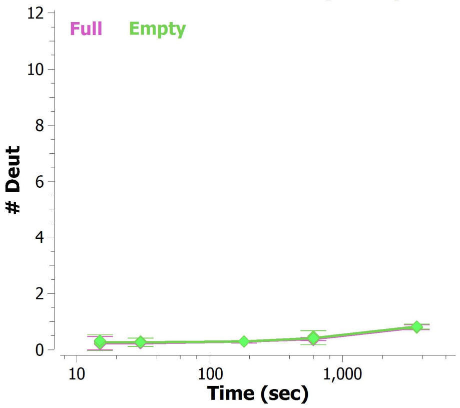

AAV8_VP1 563-578: TSEEEIKTTNPVATEE (#380)

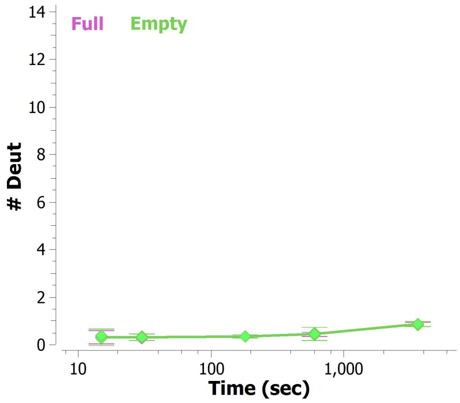

AAV8_VP1 563-586: TSEEEIKTTNPVATEEYGIVADNL (#381)

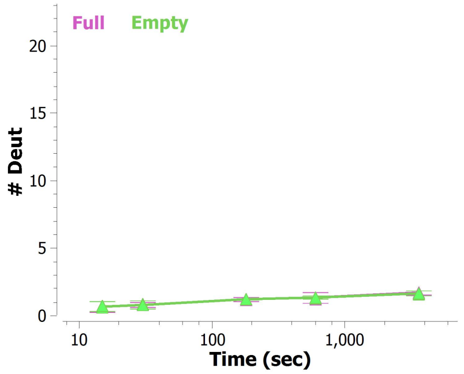

AAV8_VP1 563-587: TSEEEIKTTNPVATEEYGIVADNLQ (#382)

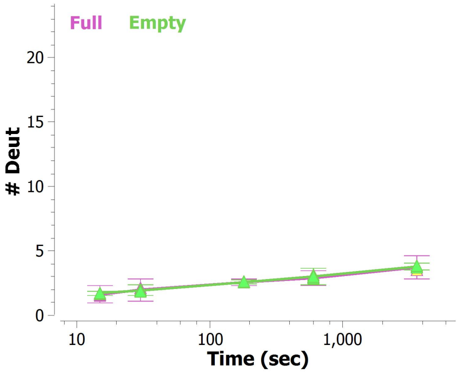

AAV8_VP1 566-576: EEIKTTNPVAT (#383)

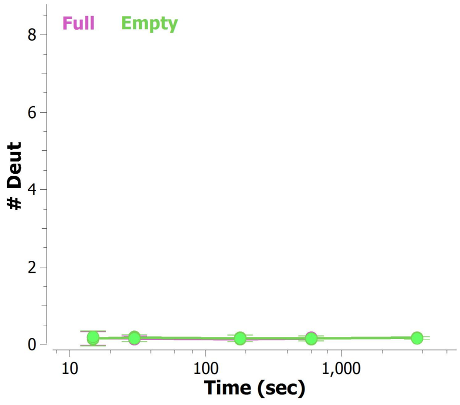

AAV8_VP1 566-578: EEIKTTNPVATEE (#384)

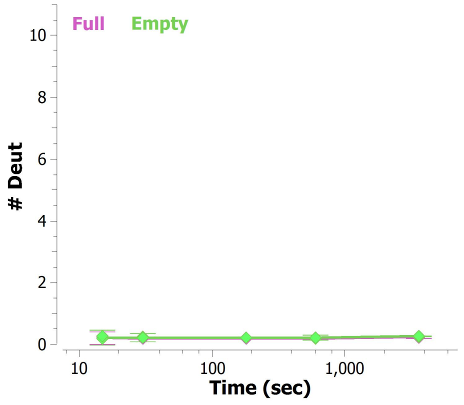

AAV8_VP1 566-579: EEIKTTNPVATEEY (#385)

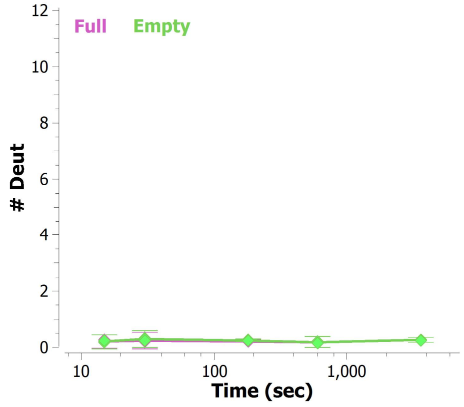

AAV8_VP1 566-586: EEIKTTNPVATEEYGIVADNL (#386)

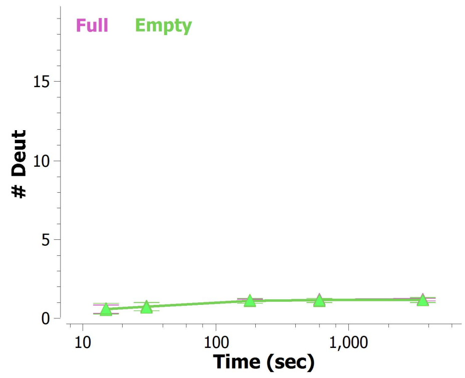

AAV8_VP1 567-576: EIKTTNPVAT (#387)

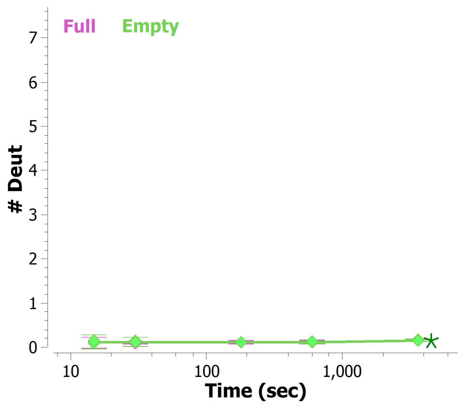

AAV8_VP1 567-578: EIKTTNPVATEE (#388)

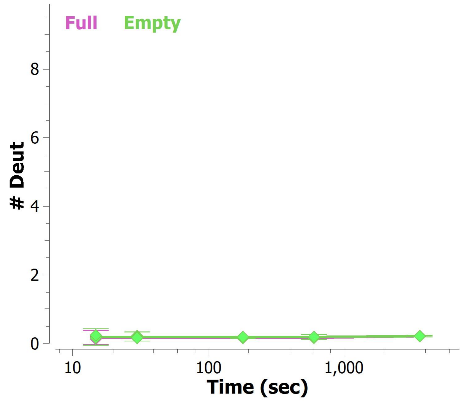

AAV8_VP1 567-586: EIKTTNPVATEEYGIVADNL (#389)

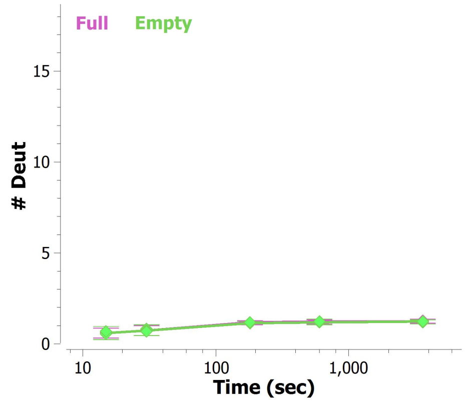

AAV8_VP1 568-576: IKTTNPVAT (#390)

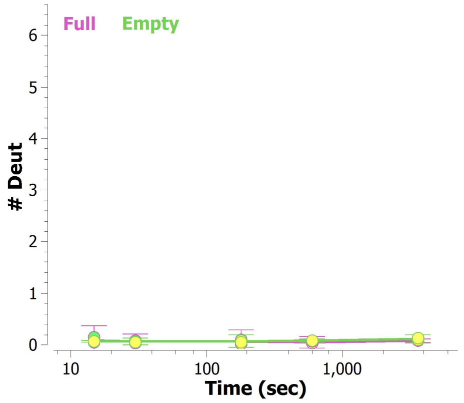

AAV8_VP1 568-578: IKTTNPVATEE (#391)

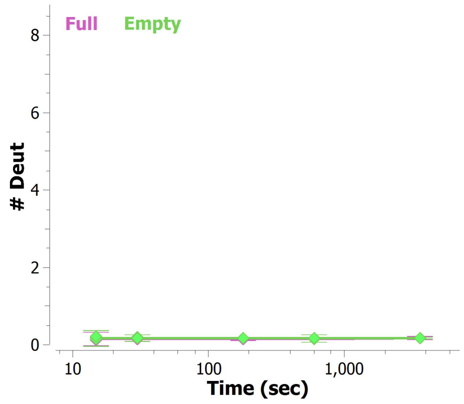

AAV8_VP1 568-586: IKTTNPVATEEYGIVADNL (#392)

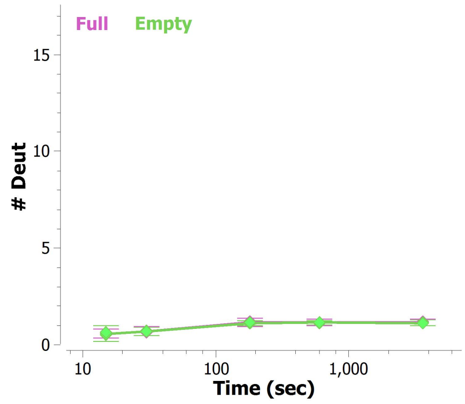

AAV8_VP1 577-584: EEYGIVAD (#393)

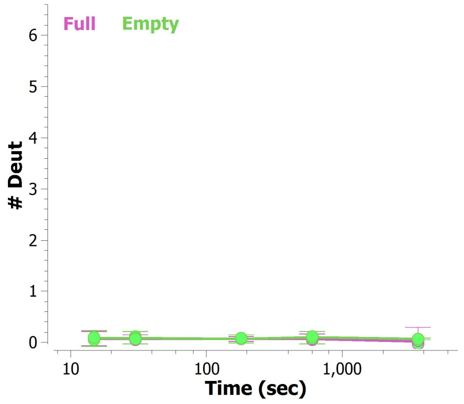

AAV8_VP1 577-586: EEYGIVADNL (#394)

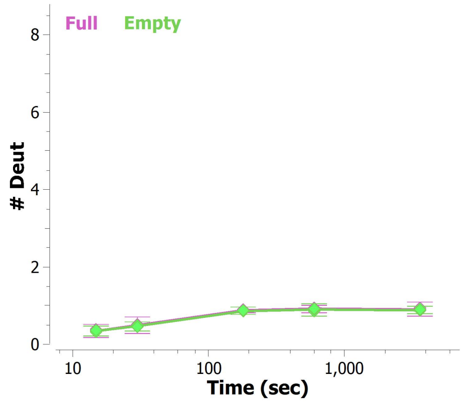

AAV8_VP1 579-583: YGIVA (#395)

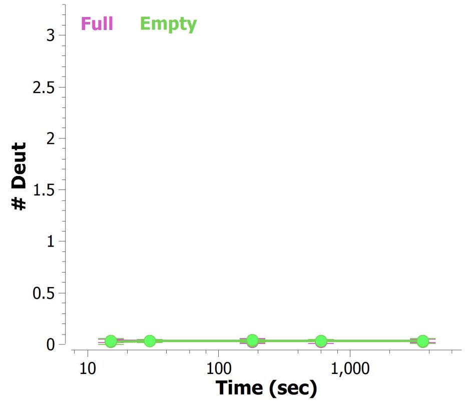

AAV8_VP1 579-584: YGIVAD (#396)

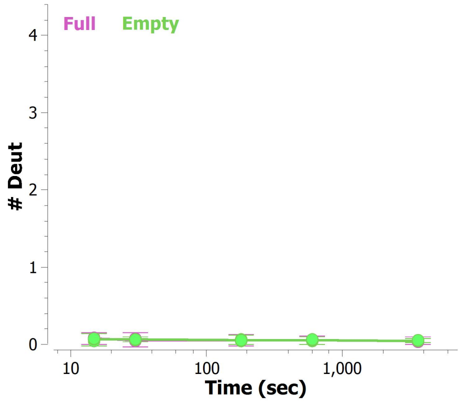

AAV8_VP1 579-585: YGIVADN (#397)

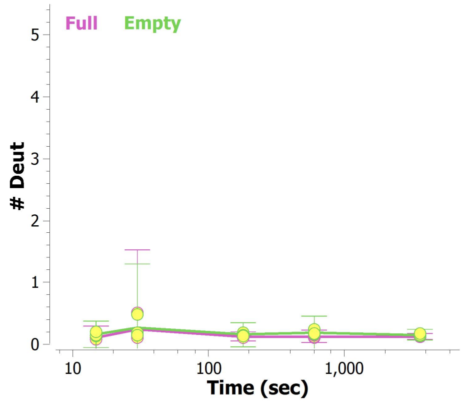

AAV8_VP1 579-586: YGIVADNL (#398)

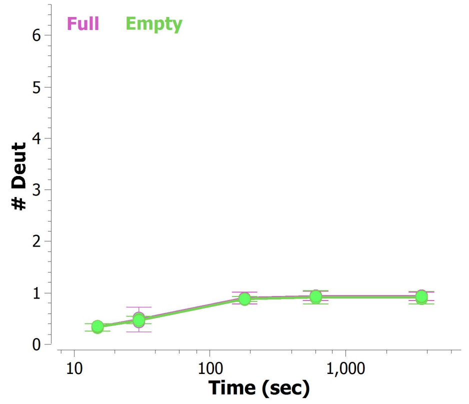

AAV8_VP1 579-587: YGIVADNLQ (#399)

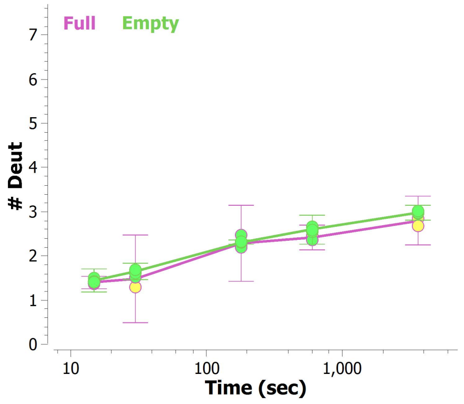

AAV8_VP1 580-586: GIVADNL (#400)

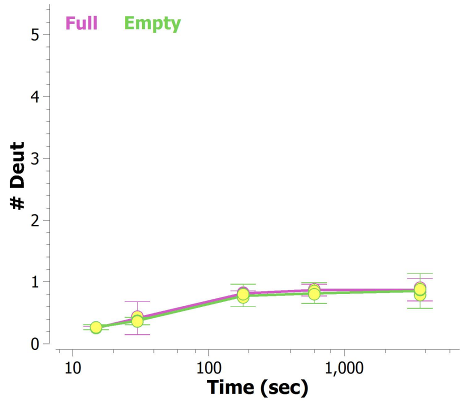

AAV8_VP1 581-586: IVADNL (#401)

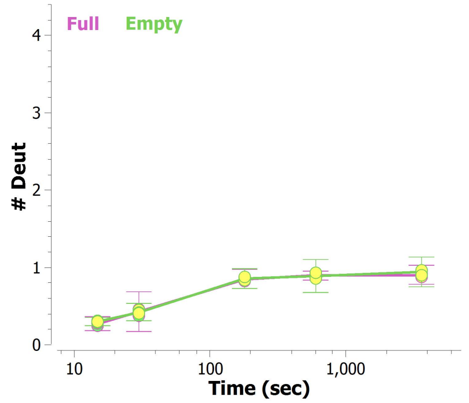

AAV8_VP1 587-613: QQNTAPQIGTVNSQGALPGMVWQNR (#402)

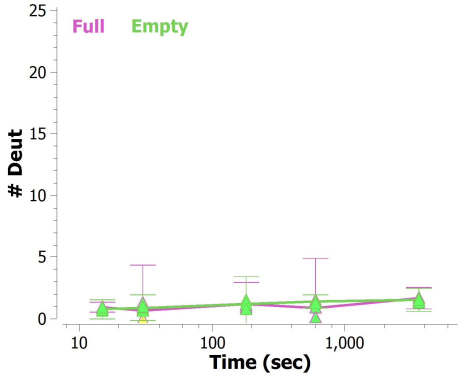

AAV8_VP1 587-621: APQIGTVNSQGALPGMVWQNRDVYL (#403)

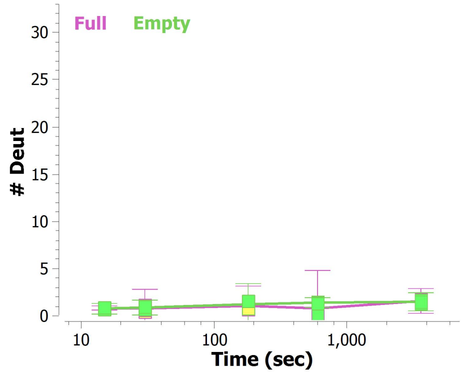

AAV8_VP1 608-613: VWQNRD (#404)

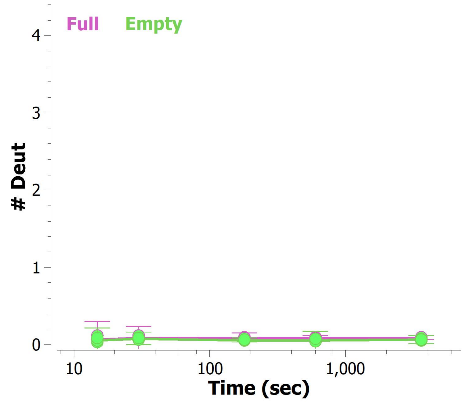

AAV8_VP1 608-616: VWQNRDVYL (#405)

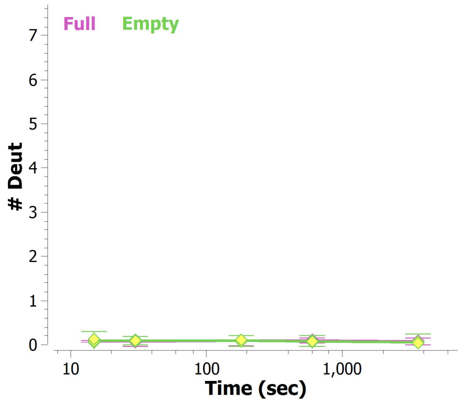

AAV8_VP1 610-621: QNRDVYLQGPIW (#406)

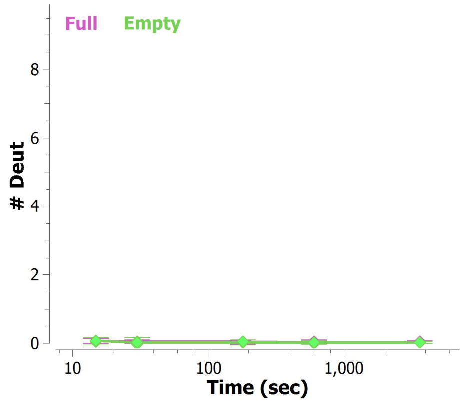

AAV8_VP1 611-621: NRDVYLQGPIW (#407)

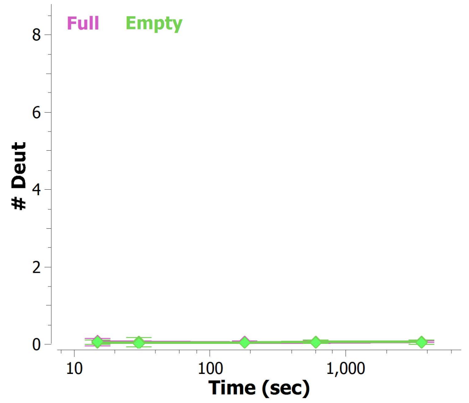

AAV8_VP1 614-621: VYLQGPIW (#408)

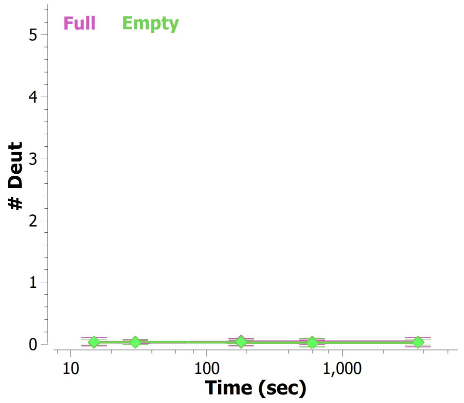

AAV8_VP1 616-621: LQGPIW (#409)

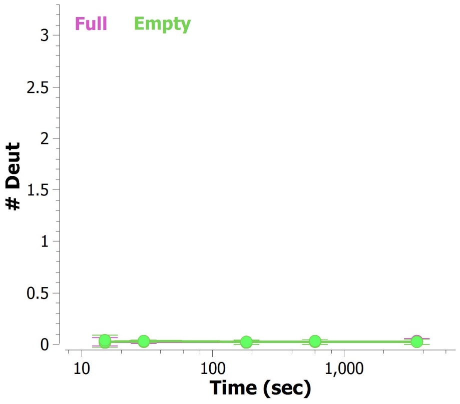

AAV8_VP1 622-631: AKIPHTDGNF (#410)

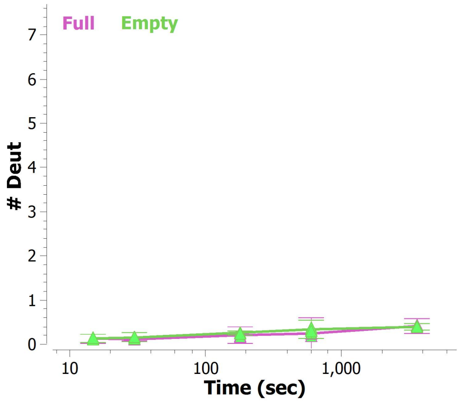

AAV8_VP1 622-637: AKIPHTDGNFHPSPLM (#411)

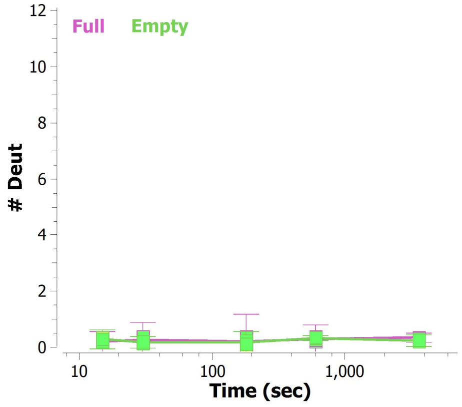

AAV8_VP1 622-639: AKIPHTDGNFHPSPLMGG (#412)

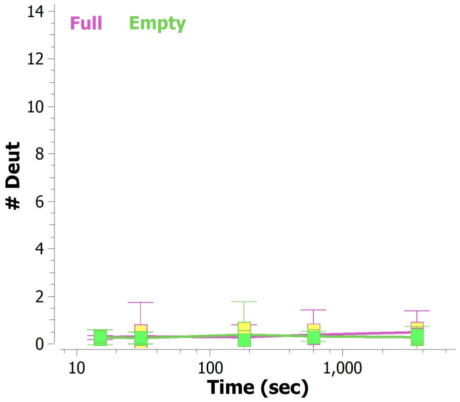

AAV8_VP1 622-640: AKIPHTDGNFHPSPLMGGF (#413)

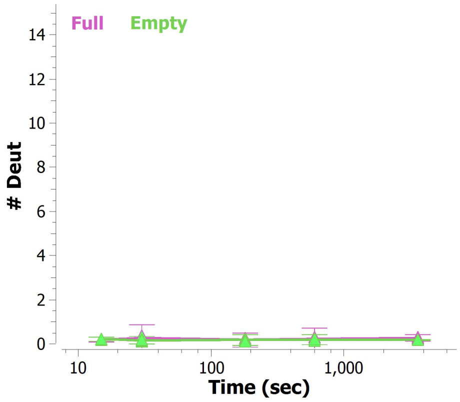

AAV8_VP1 638-650: GGFGLKHPPPQIL (#414)

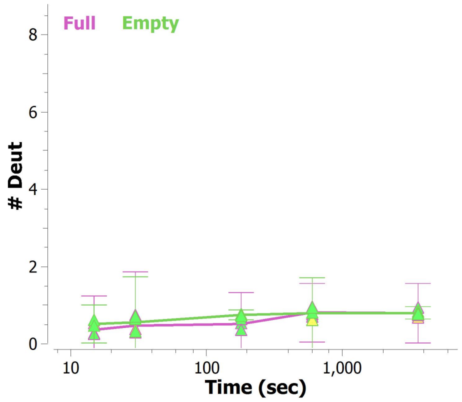

AAV8_VP1 640-650: FGLKHPPPQIL (#415)

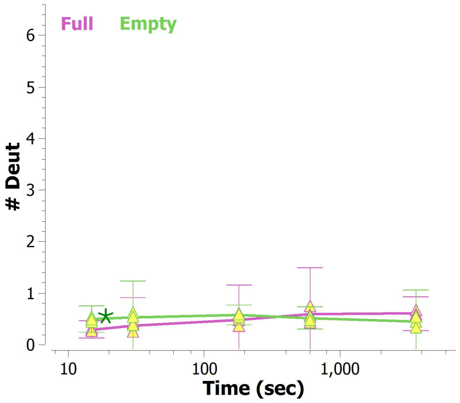

AAV8_VP1 641-650: GLKHPPPQIL (#416)

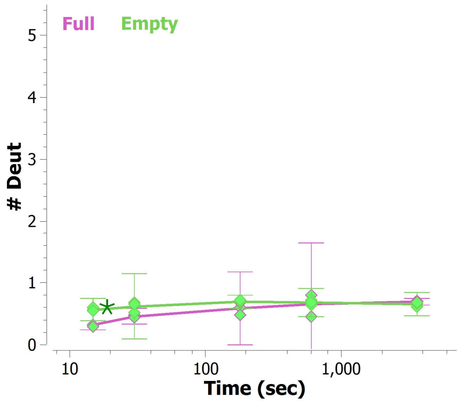

AAV8_VP1 642-650: LKHPPPQIL (#417)

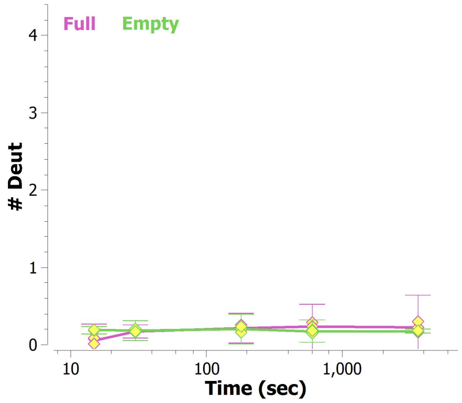

AAV8_VP1 650-669: LIKNTPVPADPPTTFNQSKL (#418)

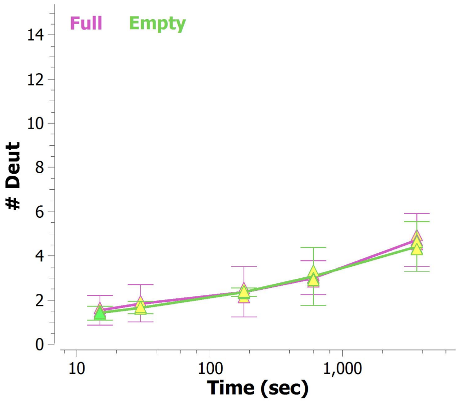

AAV8_VP1 650-671: LIKNTPVPADPPTTFNQSKLNS (#419)

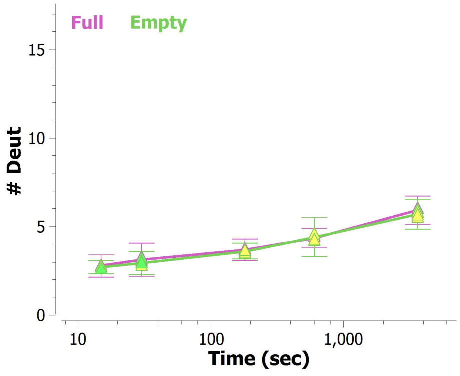

AAV8_VP1 651-663: IKNTPVPADPPTT (#420)

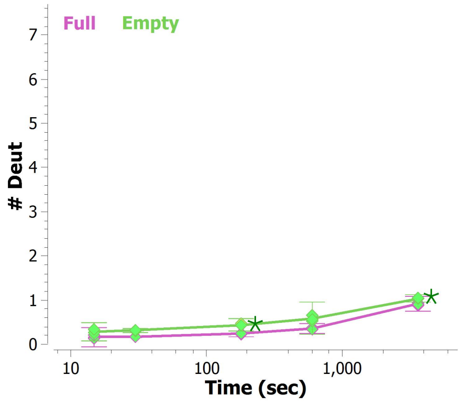

AAV8_VP1 651-664: IKNTPVPADPPTTF (#421)

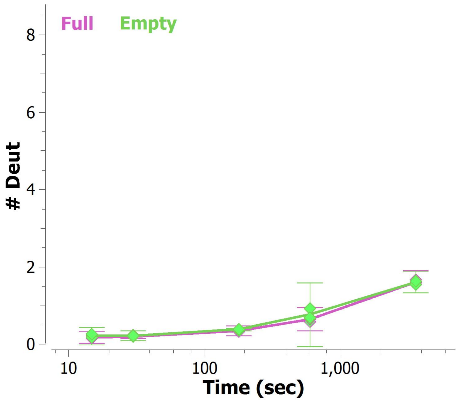

AAV8_VP1 651-665: IKNTPVPADPPTTFN (#422)

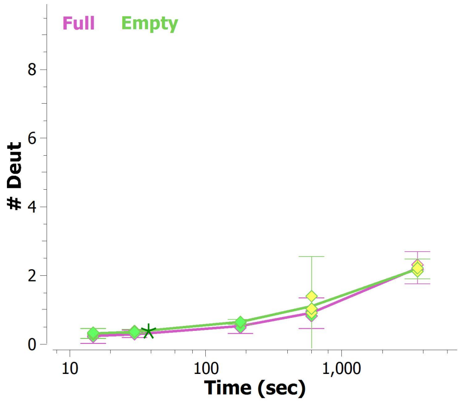

AAV8_VP1 651-666: IKNTPVPADPPTTFNQ (#423)

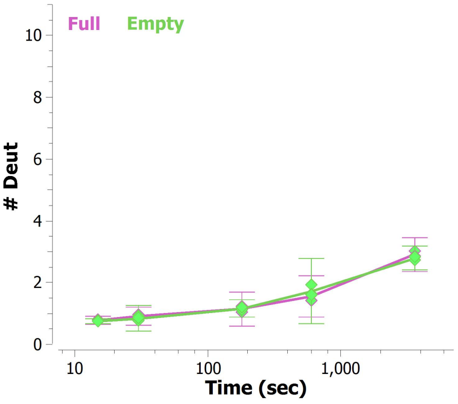

AAV8_VP1 651-669: IKNTPVPADPPTTFNQSKL (#424)

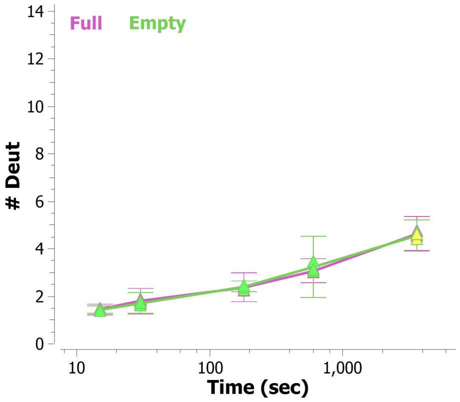

AAV8_VP1 651-671: IKNTPVPADPPTTFNQSKLNS (#425)

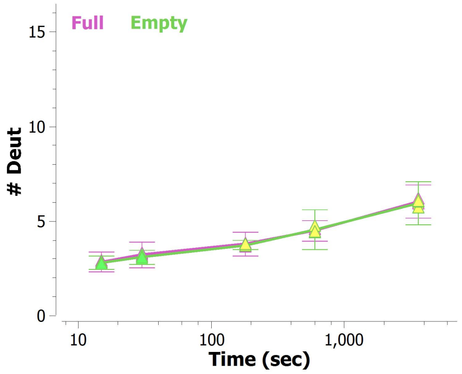

AAV8_VP1 651-672: IKNTPVPADPPTTFNQSKLNSF (#426)

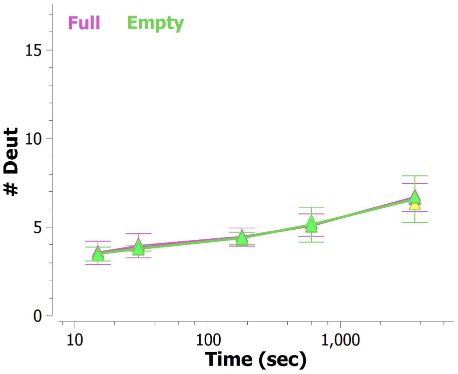

AAV8_VP1 665-669: NQSKL (#427)

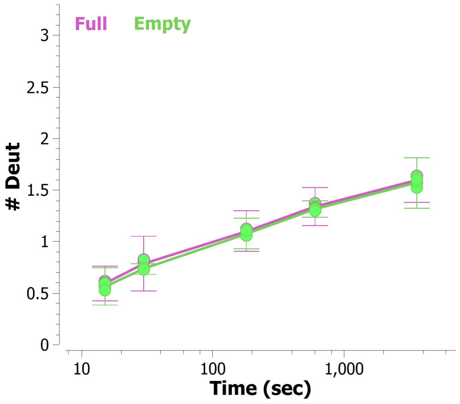

AAV8_VP1 665-671: NQSKLNS (#428)

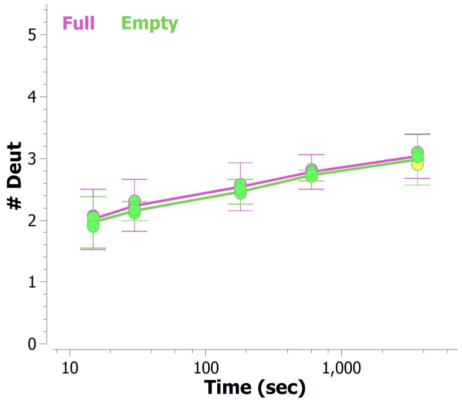

AAV8_VP1 665-672: NQSKLNSF (#429)

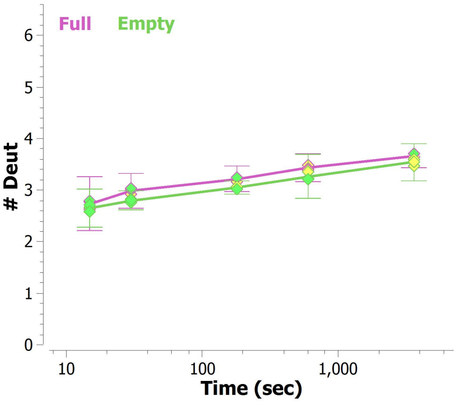

AAV8_VP1 665-684: NQSKLNSFITQYSTGQVSVE (#430)

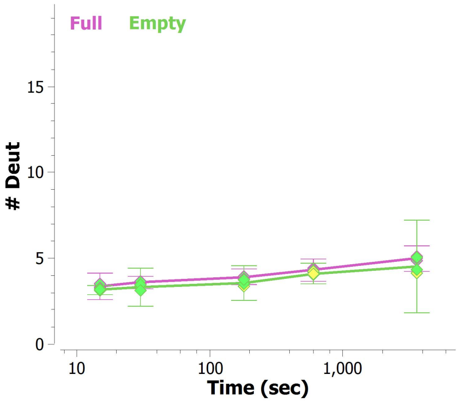

AAV8_VP1 670-675: NSFITQ (#431)

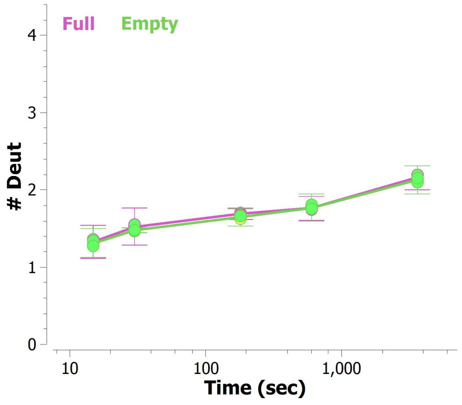

AAV8_VP1 670-683: NSFITQYSTGQVSV (#432)

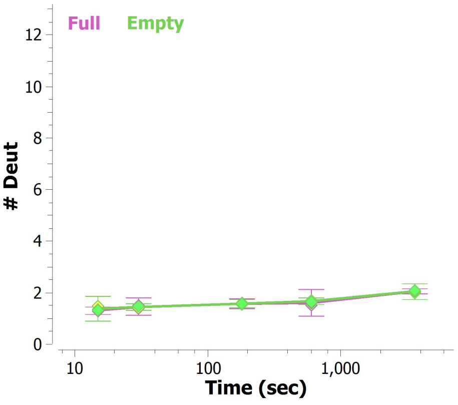

AAV8_VP1 672-675: FITQ (#433)

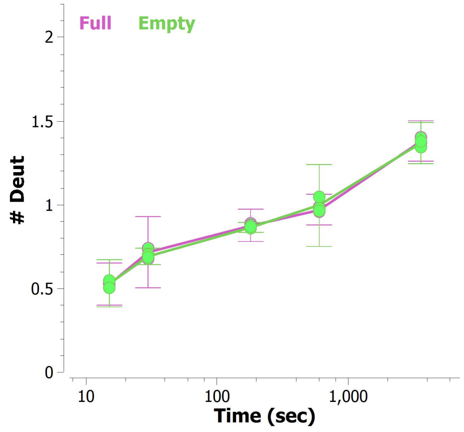

AAV8_VP1 672-676: FITQY (#434)

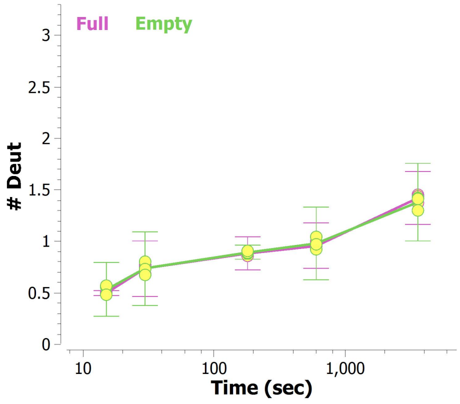

AAV8_VP1 672-682: FITQYSTGQVS (#435)

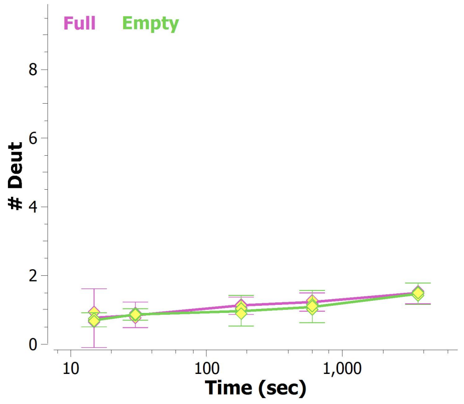

AAV8_VP1 672-683: FITQYSTGQVSV (#436)

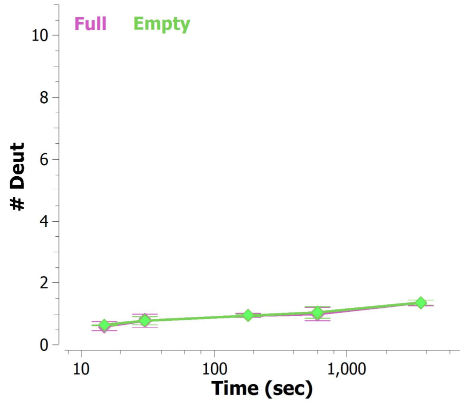

AAV8_VP1 672-684: FITQYSTGQVSVE (#437)

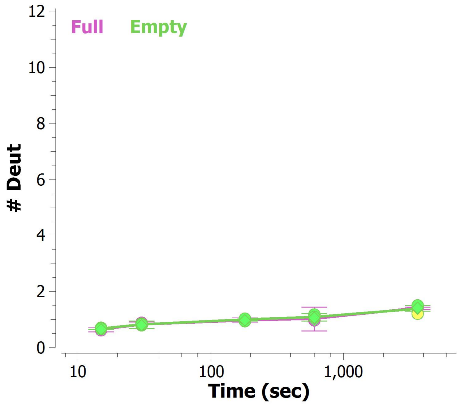

AAV8_VP1 673-676: ITQY (#438)

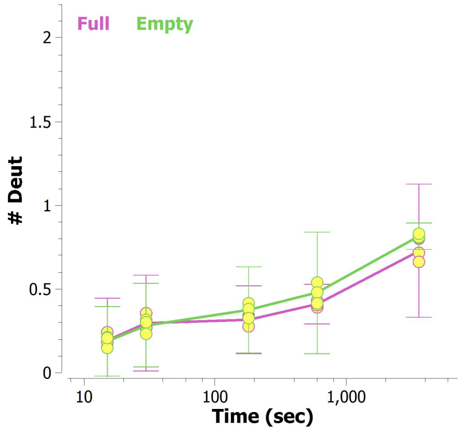

AAV8_VP1 673-683: ITQYSTGQVSV (#439)

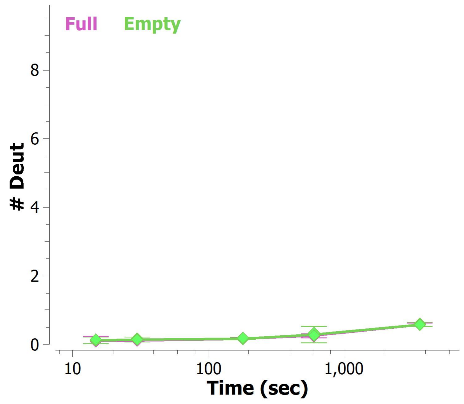

AAV8_VP1 673-684: ITQYSTGQVSVE (#440)

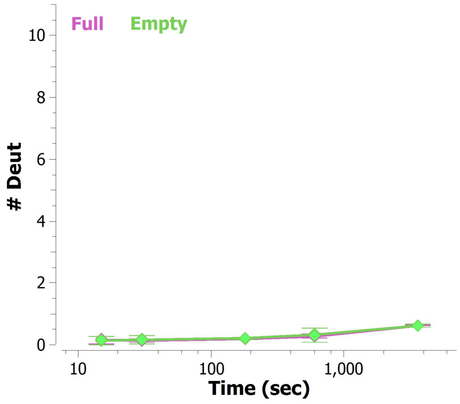

AAV8_VP1 676-683: YSTGQVSV (#441)

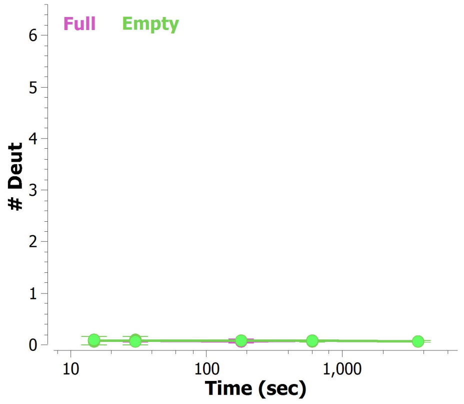

AAV8_VP1 676-684: YSTGQVSVE (#442)

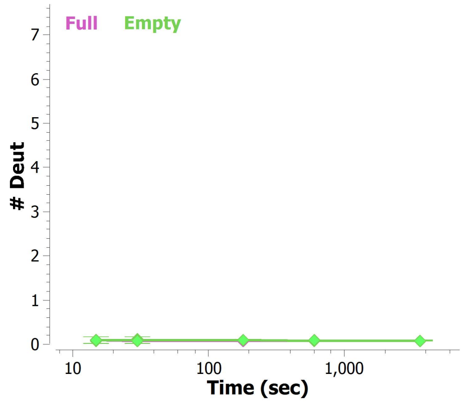

AAV8_VP1 677-684: STGQVSVE (#443)

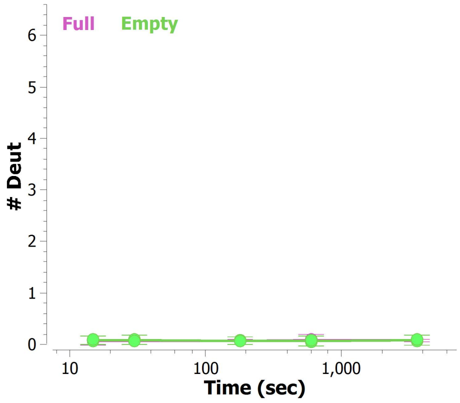

AAV8_VP1 683-689: VEIEWEL (#444)

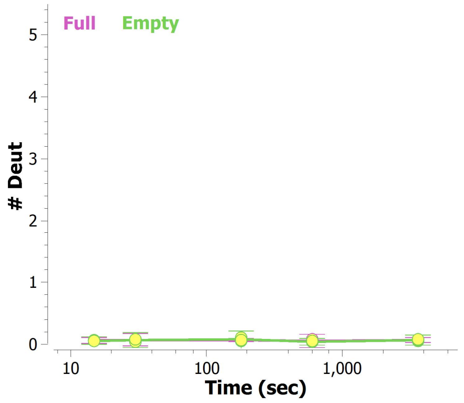

AAV8_VP1 683-703: VEIEWELQKENSKRWNPEIQY (#445)

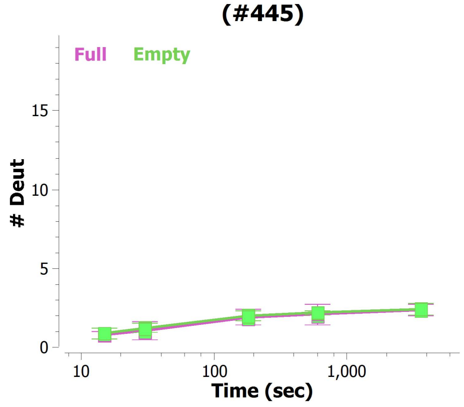

AAV8_VP1 684-689: EIEWEL (#446)

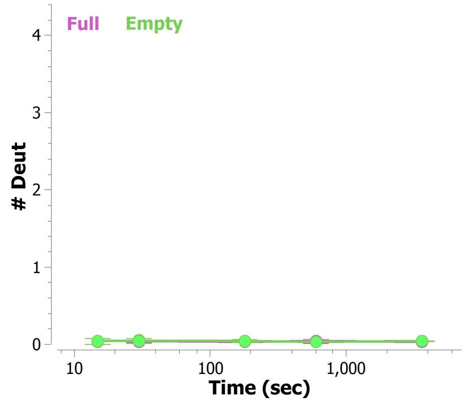

AAV8_VP1 684-703: EIEWELQKENSKRWNPEIQY (#447)

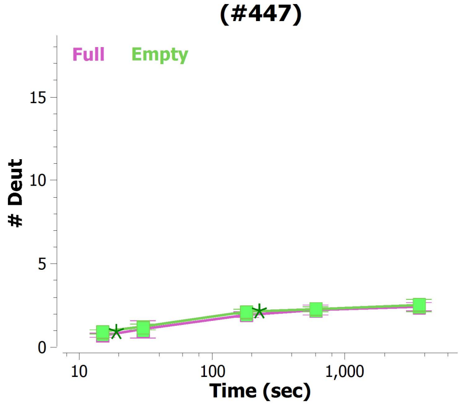

AAV8_VP1 684-705: EIEWELQKENSKRWNPEIQYTS (#448)

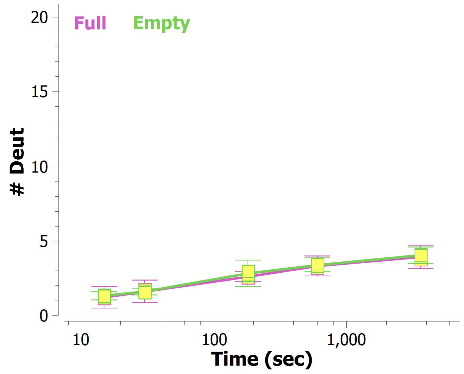

AAV8_VP1 684-706: EIEWELQKENSKRWNPEIQYTSN (#449)

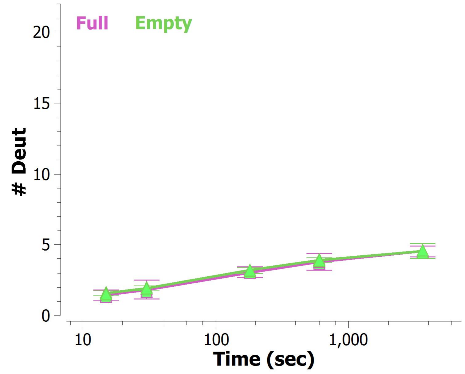

AAV8_VP1 684-707: EIEWELQKENSKRWNPEIQYTSNY (#450)

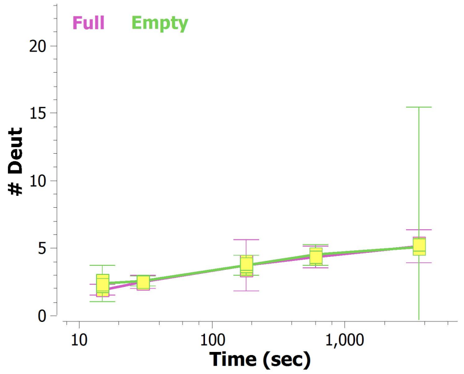

AAV8_VP1 685-689: IEWEL (#451)

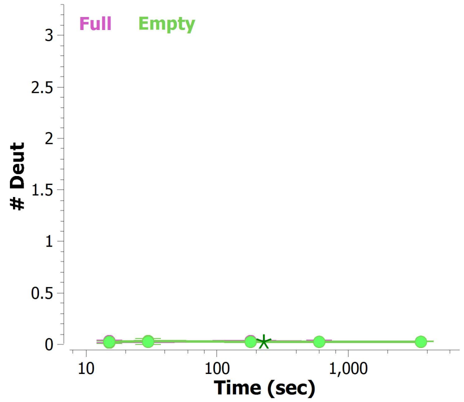

AAV8_VP1 685-690: IEWELQ (#452)

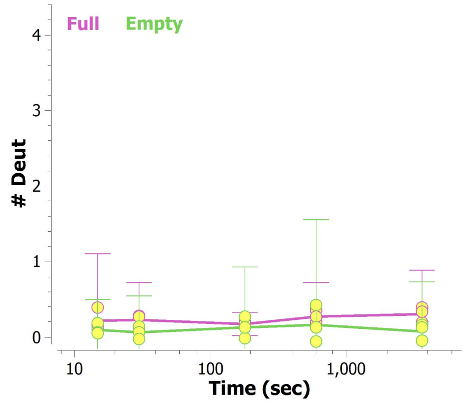

AAV8_VP1 685-702: IEWELQKENSKRWNPEIQ (#453)

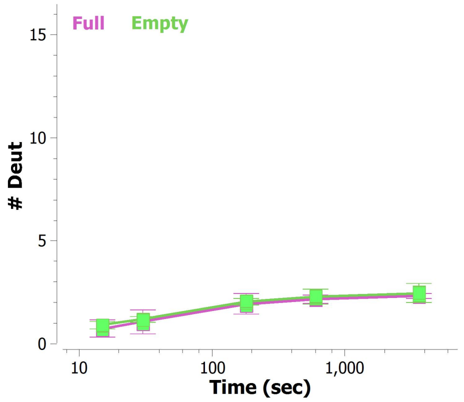

AAV8_VP1 685-703: IEWELQKENSKRWNPEIQY (#454)

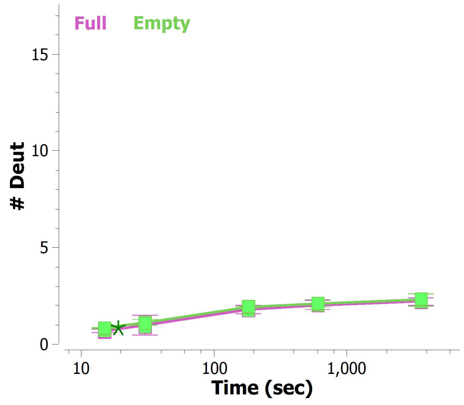

AAV8_VP1 685-706: IEWELQKENSKRWNPEIQYTSN (#455)

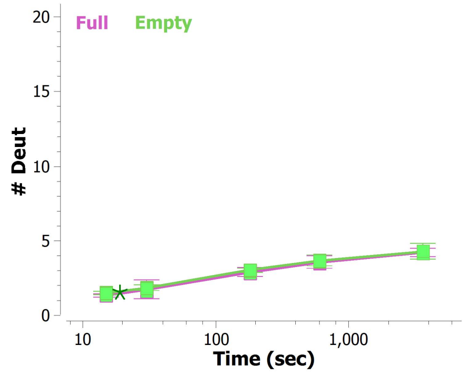

AAV8_VP1 685-707: IEWELQKENSKRWNPEIQYTSNY (#456)

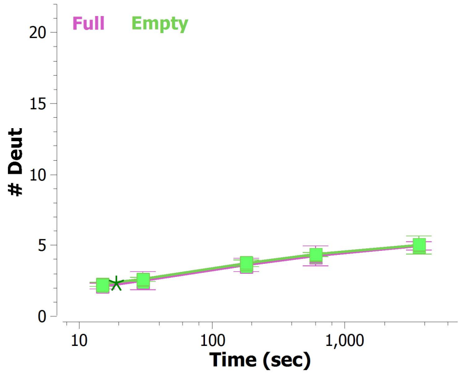

AAV8_VP1 687-703: WELQKENSKRWNPEIQY (#457)

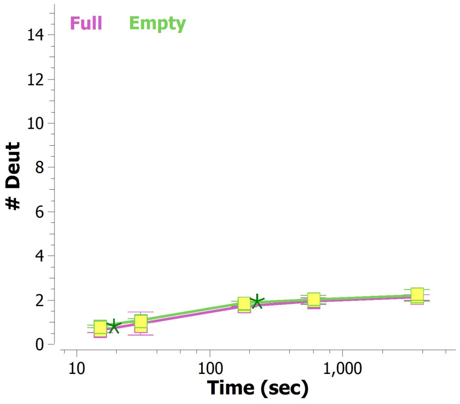

AAV8_VP1 687-706: WELQKENSKRWNPEIQYTSN (#458)

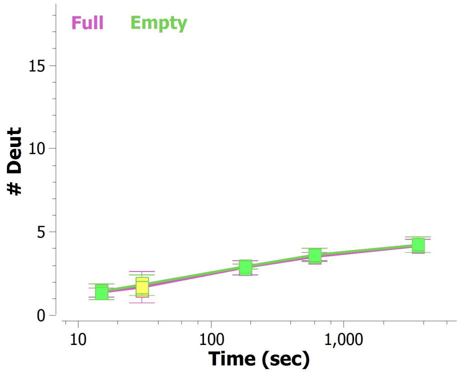

AAV8_VP1 688-703: ELQKENSKRWNPEIQY (#459)

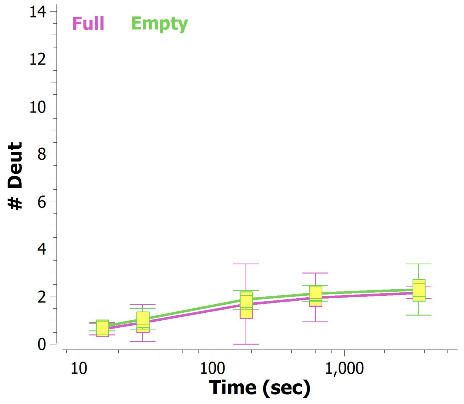

AAV8_VP1 688-706: ELQKENSKRWNPEIQYTSN (#460)

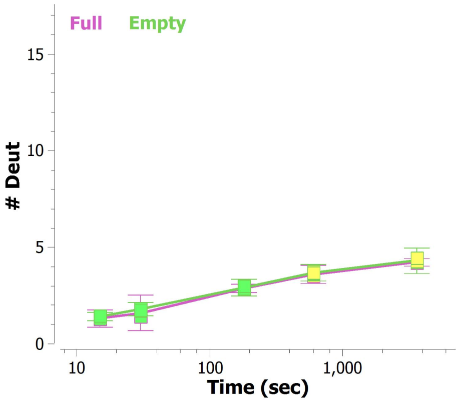

AAV8_VP1 689-706: LQKENSKRWNPEIQYTSN (#461)

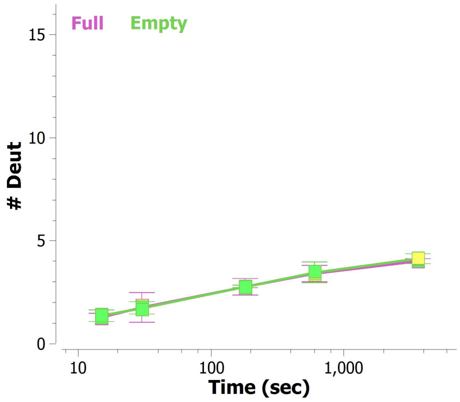

AAV8_VP1 690-702: QKENSKRWNPEIQ (#462)

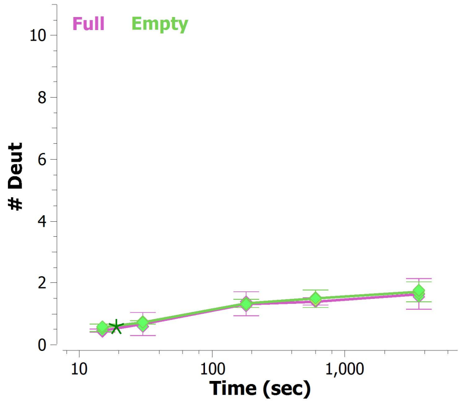

AAV8_VP1 690-703: QKENSKRWNPEIQY (#463)

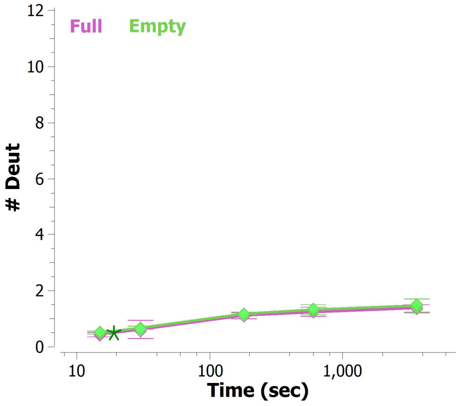

AAV8_VP1 690-706: QKENSKRWNPEIQYTSN (#464)

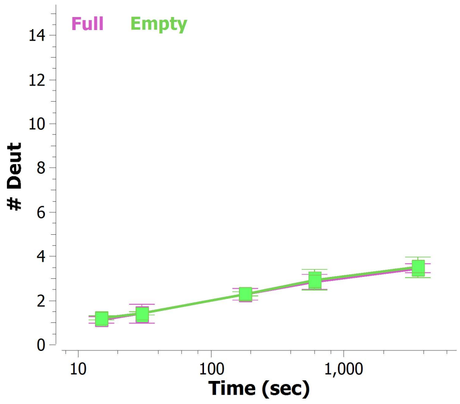

AAV8_VP1 690-707: QKENSKRWNPEIQYTSNY (#465)

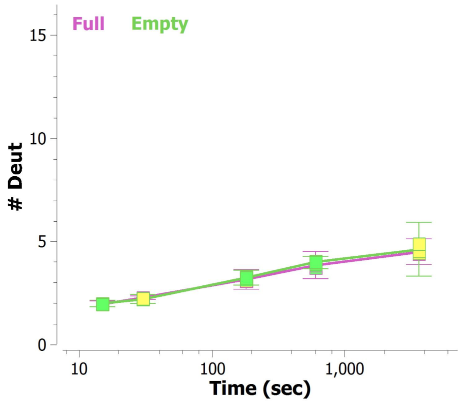

AAV8_VP1 691-703: KENSKRWNPEIQY (#466)

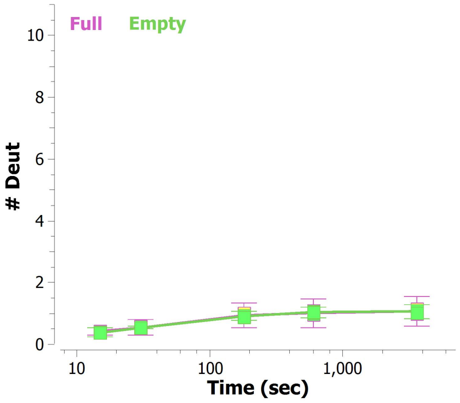

AAV8_VP1 695-703: KRWNPEIQY (#467)

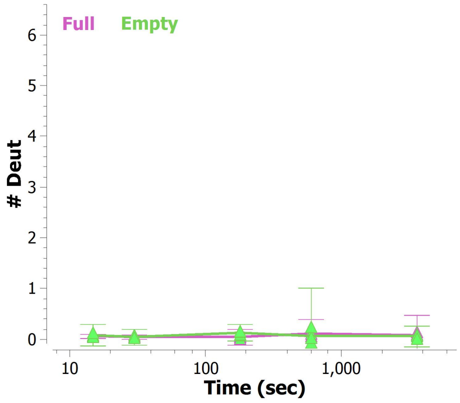

AAV8_VP1 695-706: KRWNPEIQYTSN (#468)

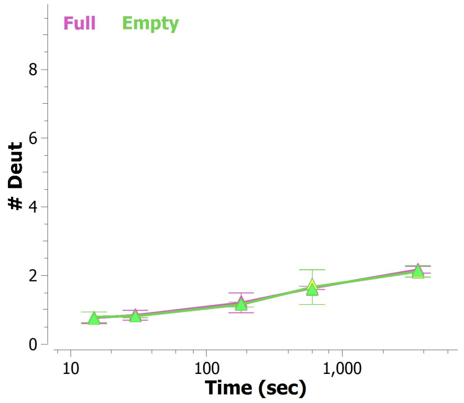

AAV8_VP1 703-707: YTSNY (#469)

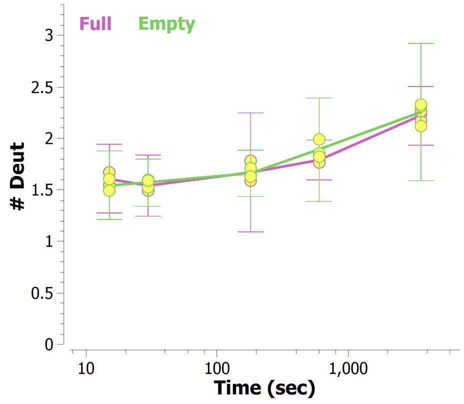

AAV8_VP1 704-707: TSNY (#470)

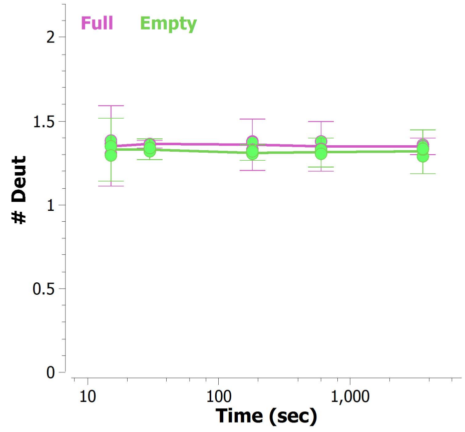

AAV8_VP1 704-714: TSNYYKSTSVD (#471)

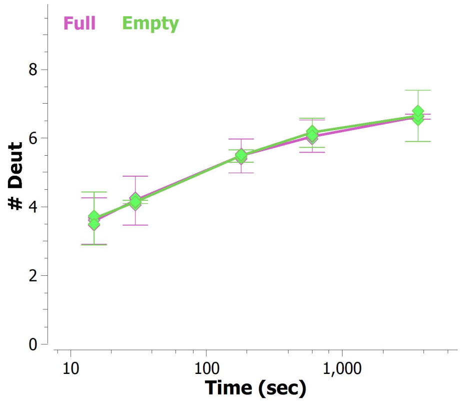

AAV8_VP1 704-715: TSNYYKSTSVDF (#472)

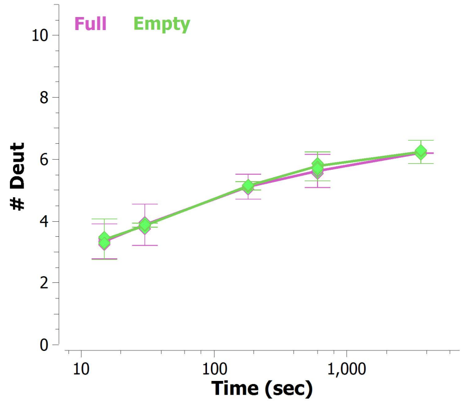

AAV8_VP1 707-714: YYKSTSVD (#473)

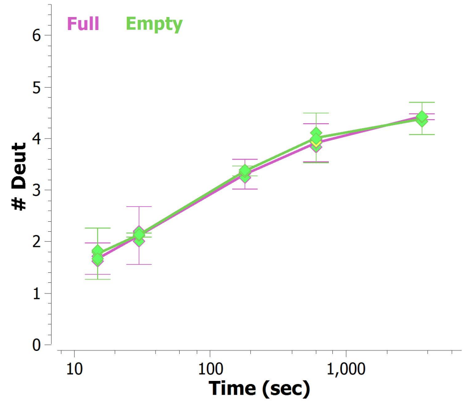

AAV8_VP1 707-715: YYKSTSVDF (#474)

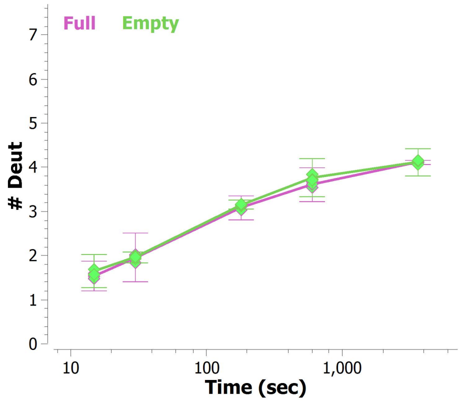

AAV8_VP1 708-715: YKSTSVDF (#475)

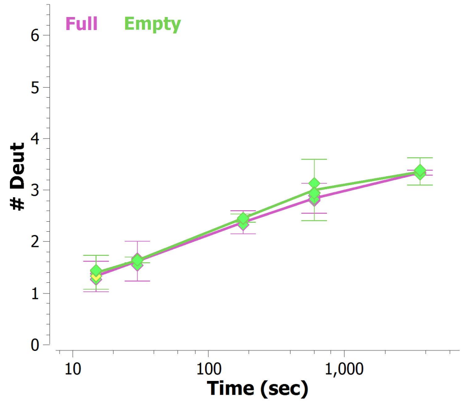

AAV8_VP1 715-722: FAVNTEGV (#476)

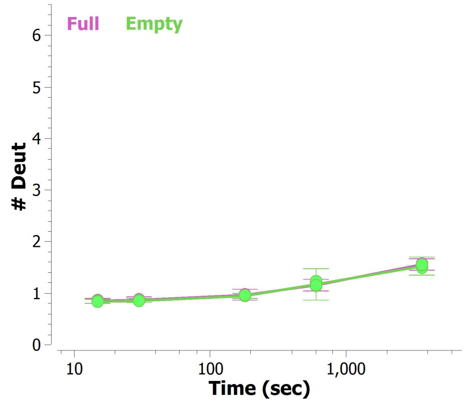

AAV8_VP1 715-733: FAVNTEGVYSEPRPIGTRY (#477)

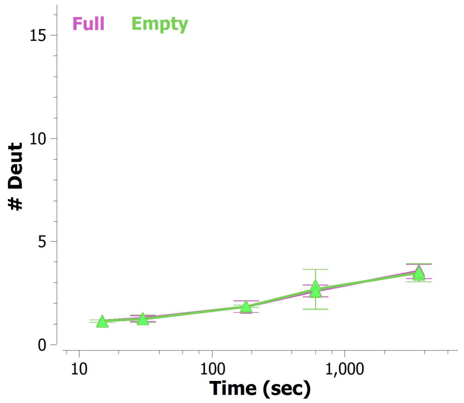

AAV8_VP1 715-738: FAVNTEGVYSEPRPIGTRYLTRNL (#478)

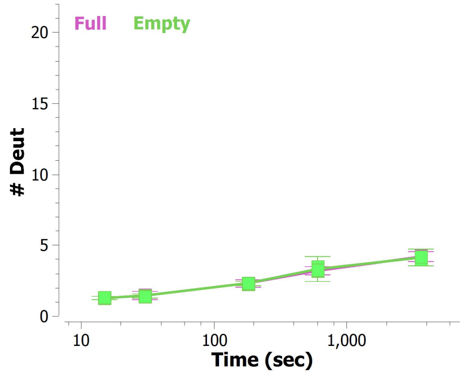

AAV8_VP1 716-722: AVNTEGV (#479)

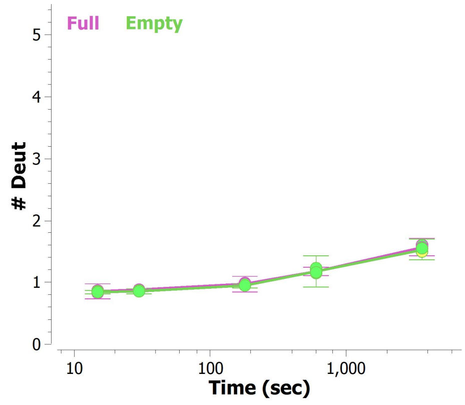

AAV8_VP1 716-731: AVNTEGVYSEPRPIGT (#480)

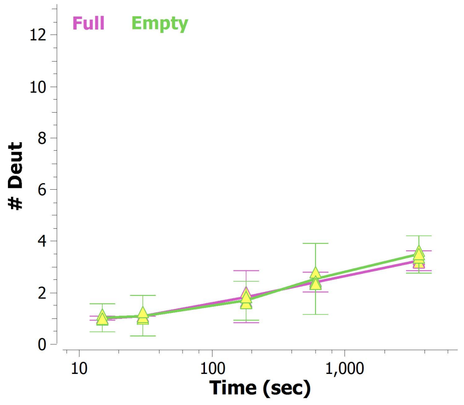

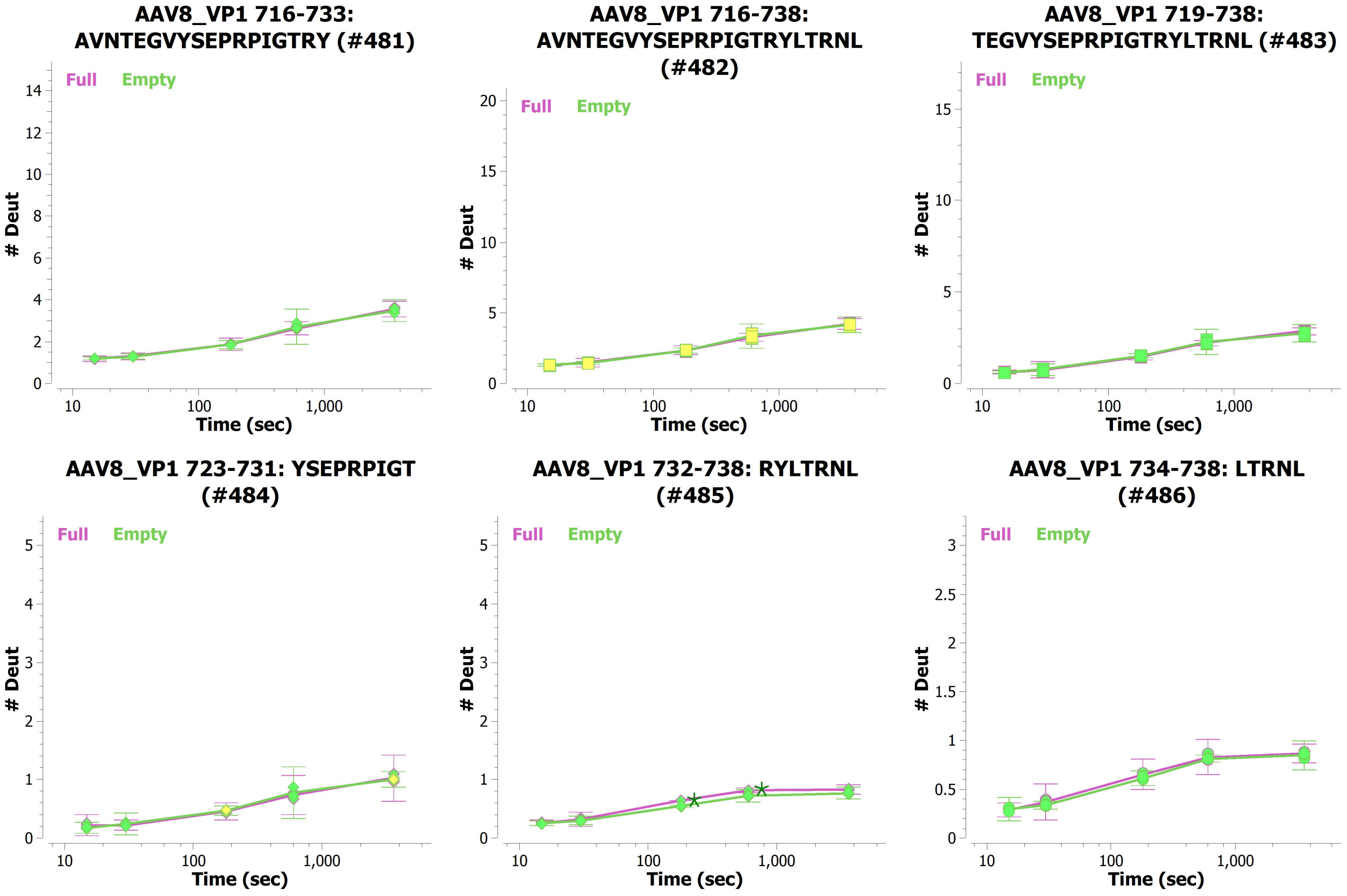

Supplementary Figure S5. Deuterium uptake plots of all peptides without any post translational modification except VP1 N-terminal peptides at a labeling temperature of 4 °C. Purple colored plots represents full capsid, and green colored plots represents empty capsid. Error bars representing standard deviation for each triplicate measurement are displayed.

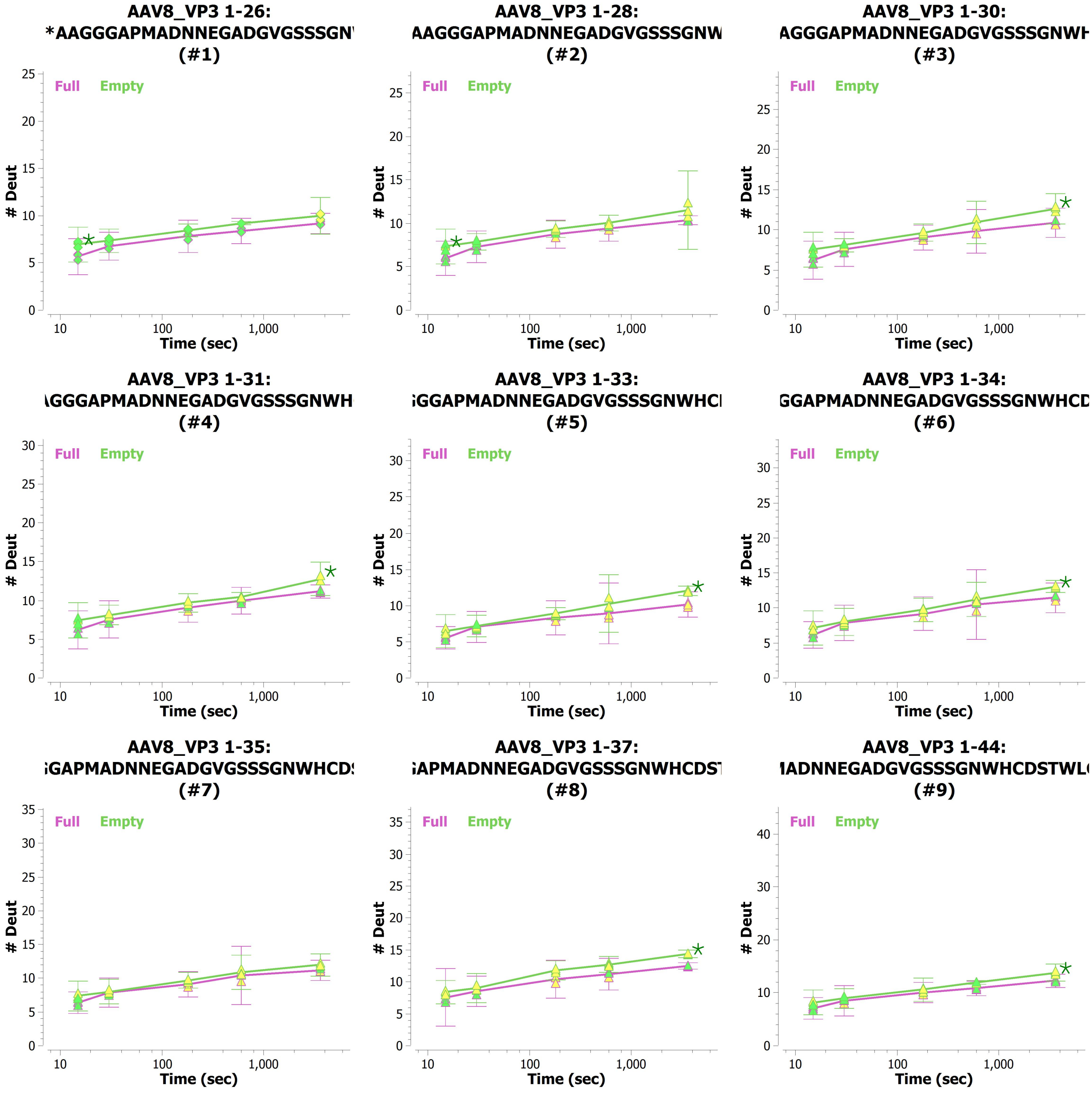

Supplementary Figure S6. Deuterium uptake plots of all peptides with N-terminal Met excision and acetylation of VP3 N-terminal peptides at a labeling temperature of 4 °C. Purple colored plots represents full capsid, and green colored plots represents empty capsid. Error bars representing standard deviation for each triplicate measurement are displayed.

AAV8_VP1 1-13: M*AADGYLPDWLED (#1)

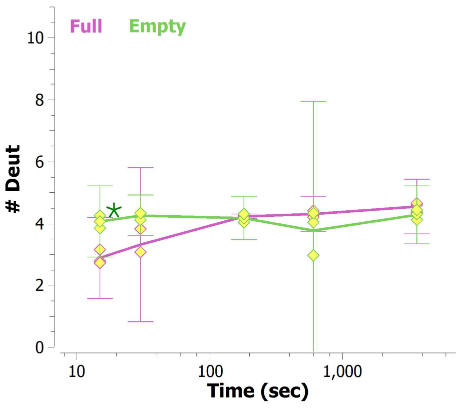

AAV8_VP1 1-14: M*AADGYLPDWLEDN (#2)

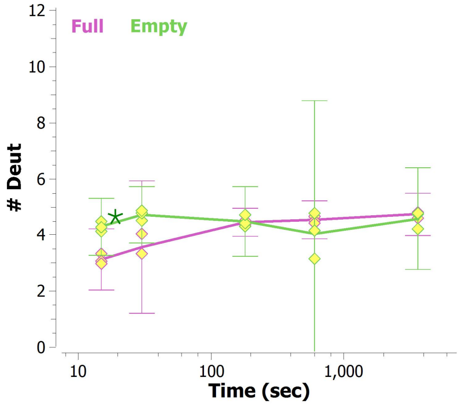

AAV8_VP1 12-18: EDNLSEG (#3)

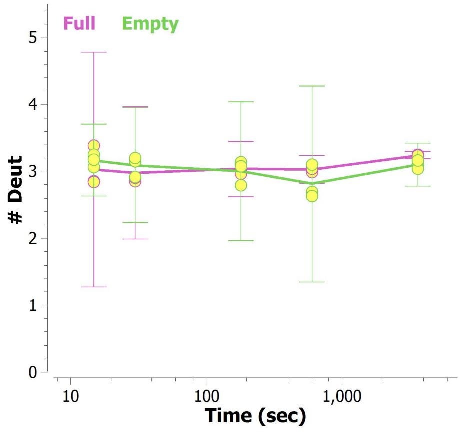

AAV8_VP1 12-21: EDNLSEGIRE (#4)

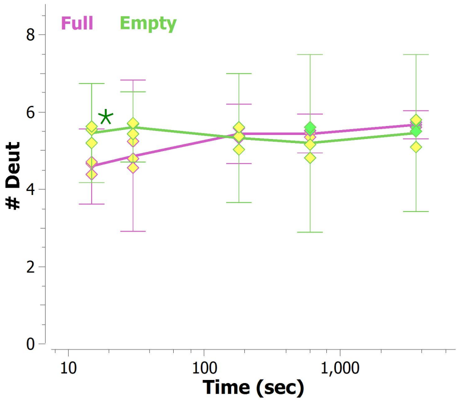

AAV8_VP1 16-22: SEGIREW (#5)

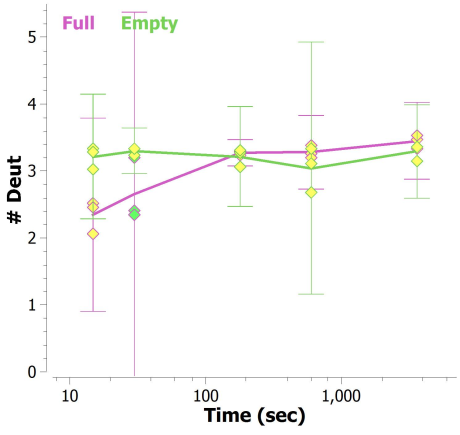

AAV8_VP1 68-83: ADAAALEHDKAYDQQL (#6)

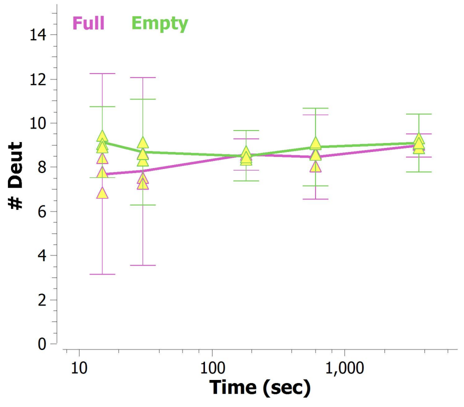

AAV8_VP1 70-100: ALEHDKAYDQQLQAGDNPYLRYNHAD (#7)

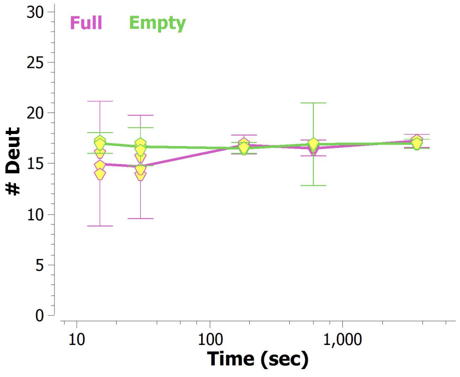

AAV8_VP1 71-78: AALEHDKA (#8)

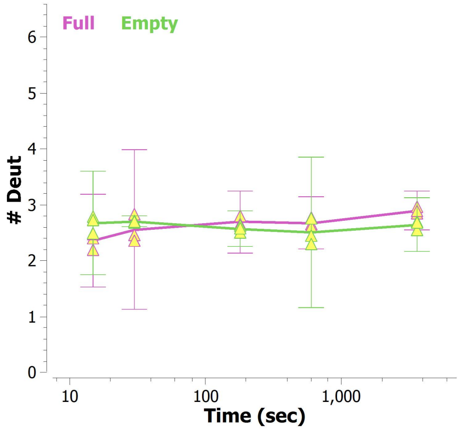

AAV8_VP1 71-83: AALEHDKAYDQQL (#9)

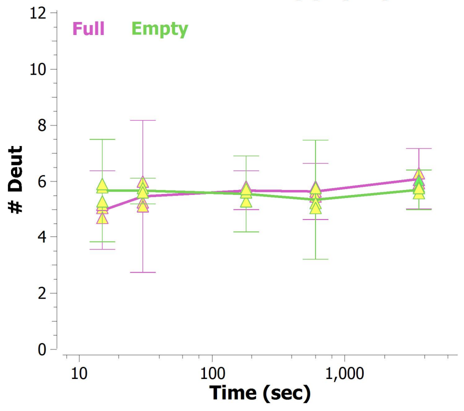

AAV8_VP1 71-96: AALEHDKAYDQQLQAGDNPYLRYNHA (#10)

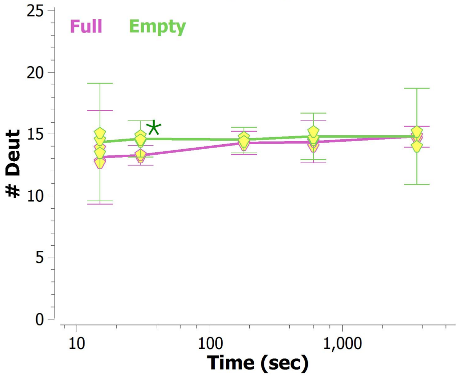

AAV8_VP1 71-99: LEHDKAYDQQLQAGDNPYLRYNHAD (#11)

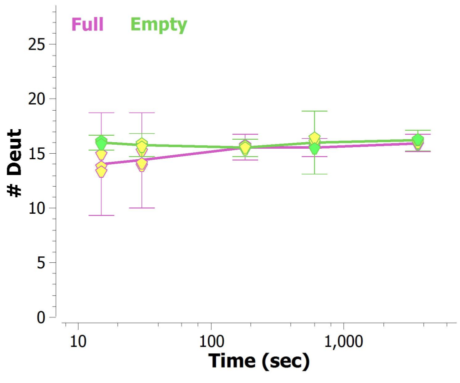

AAV8_VP1 73-83: LEHDKAYDQQL (#12)

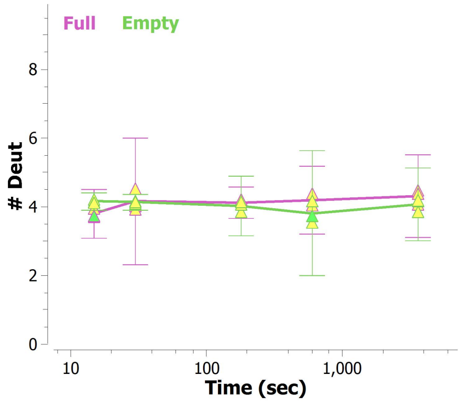

AAV8_VP1 73-99:
EHDKAYDQQLQAGDNPYLRYNHADA
(#13)

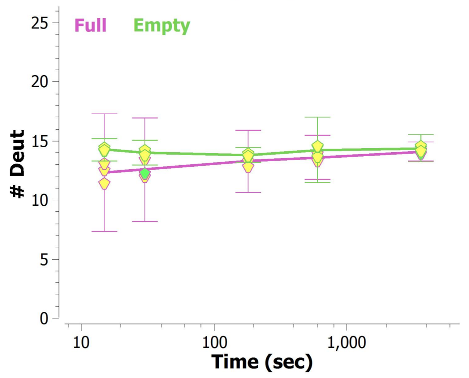

AAV8_VP1 74-96:
EHDKAYDQQLQAGDNPYLRYNHA
(#14)

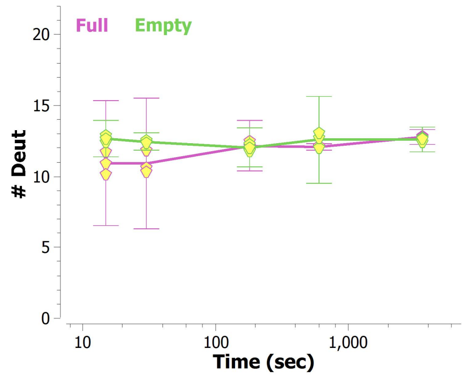

AAV8_VP1 74-99:
EHDKAYDQQLQAGDNPYLRYNHADAE
(#15)

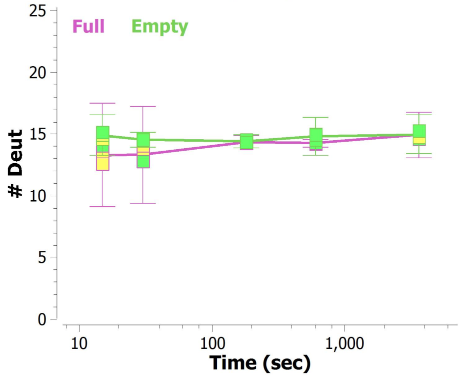

AAV8_VP1 74-100:
EHDKAYDQQLQAGDNPYLRYNHADAE
(#16)

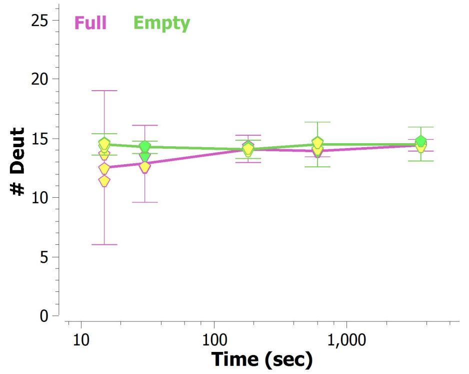

AAV8_VP1 79-83: YDQQL (#17)

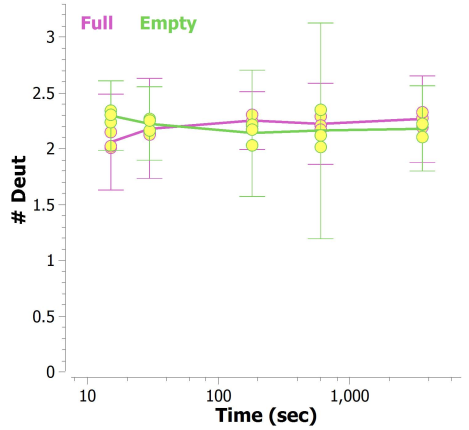

AAV8_VP1 79-96:
YDQQLQAGDNPYLRYNHA (#18)

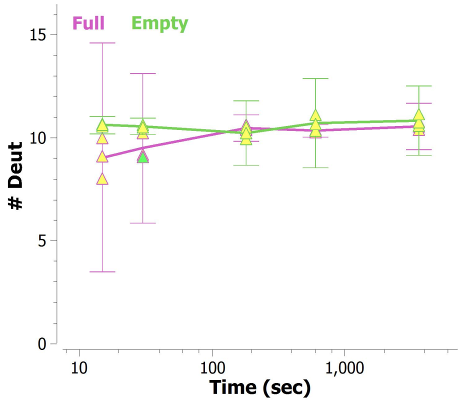

AAV8_VP1 79-99:
YDQQLQAGDNPYLRYNHADAE
(#19)

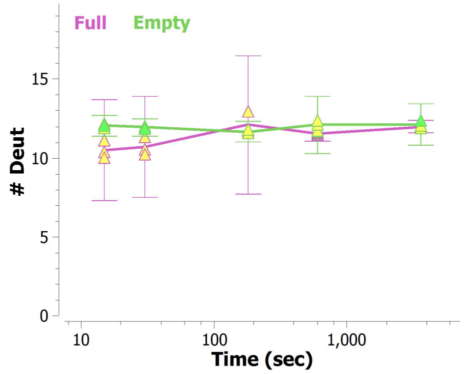

AAV8_VP1 84-91: QAGDNPYL
(#20)

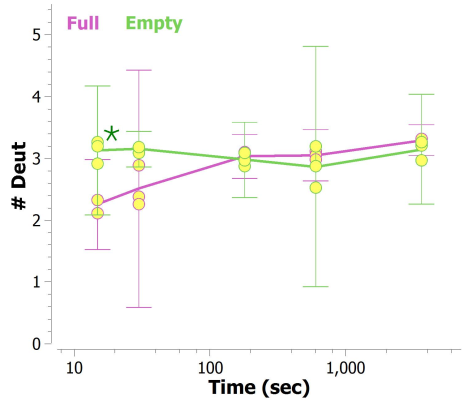

AAV8_VP1 84-96:
QAGDNPYLRYNHA (#21)

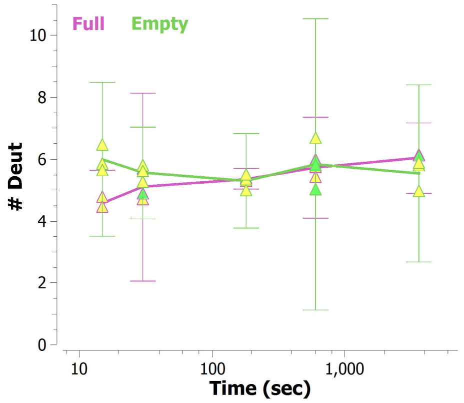

AAV8_VP1 84-99:
QAGDNPYLRYNHADAE (#22)

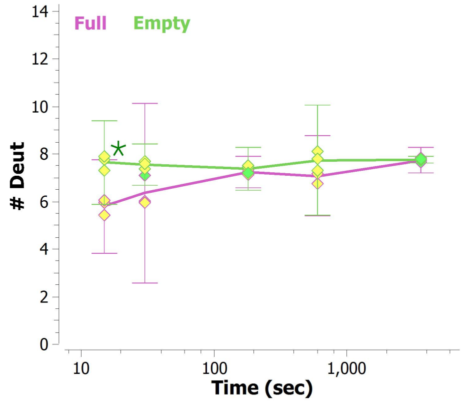

AAV8_VP1 84-100:
QAGDNPYLRYNHADAEF (#23)

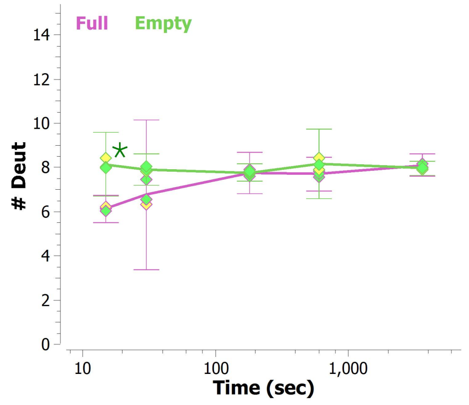

AAV8_VP1 89-100:
PYLRYNHADAEF (#24)

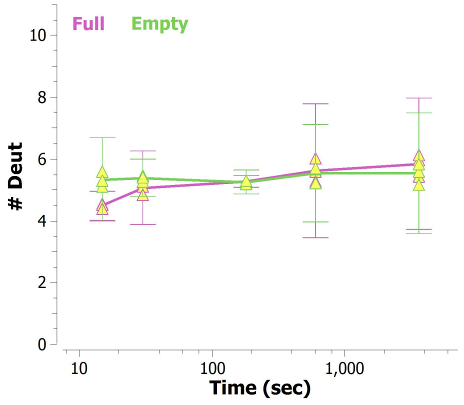

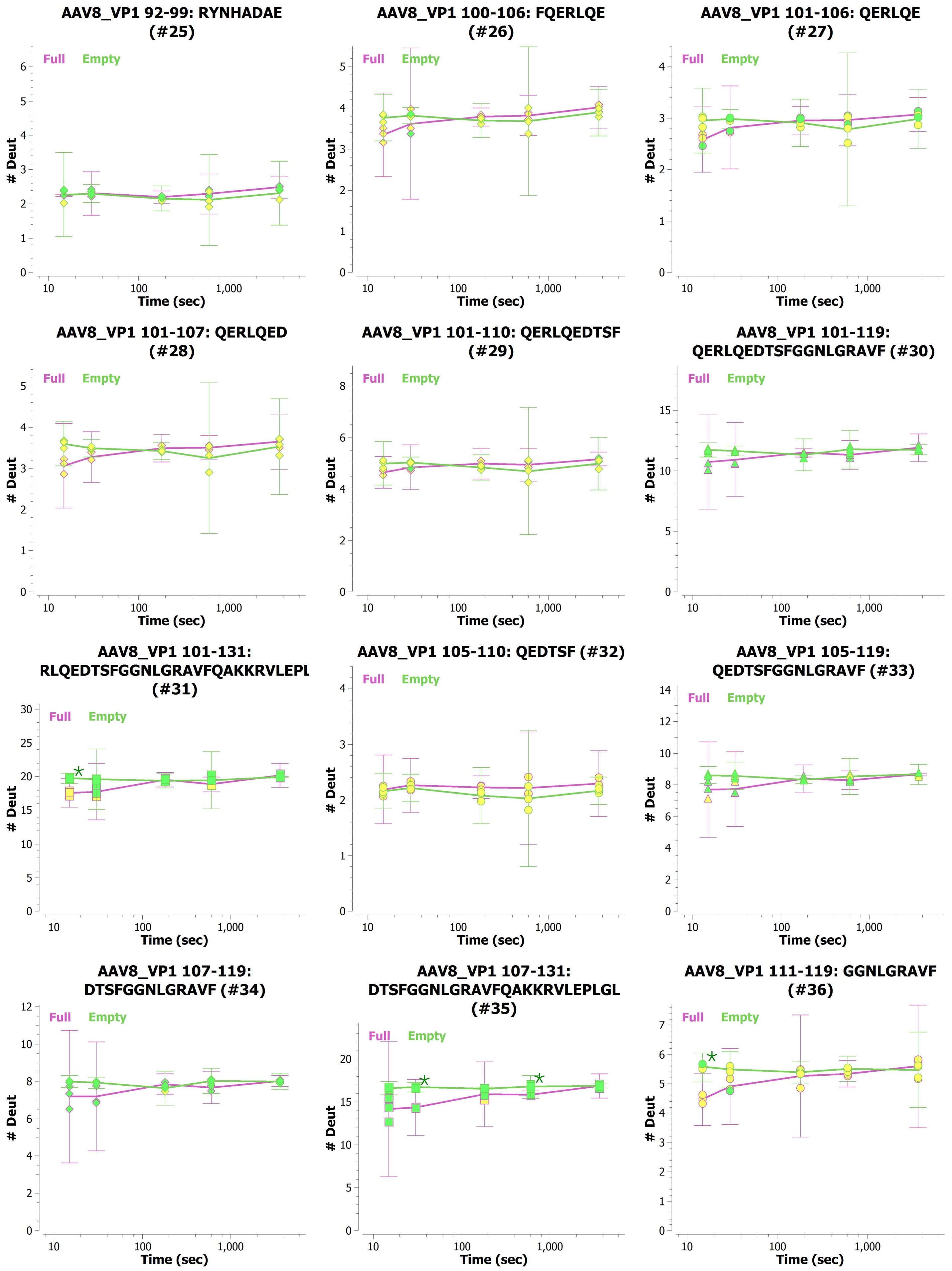
AAV8_VP1 92-99: RYNHADAE (#25)
AAV8_VP1 100-106: FQERLQE (#26)
AAV8_VP1 101-106: QERLQE (#27)
AAV8_VP1 101-107: QERLQED (#28)
AAV8_VP1 101-110: QERLQEDTSF (#29)
AAV8_VP1 101-119: QERLQEDTSFGGNLGRAVF (#30)
AAV8_VP1 101-131: RLQEDTSFGGNLGRAVFQAKKRVLEPL (#31)
AAV8_VP1 105-110: QEDTSF (#32)
AAV8_VP1 105-119: QEDTSFGGNLGRAVF (#33)
AAV8_VP1 107-119: DTSFGGNLGRAVF (#34)
AAV8_VP1 107-131: DTSFGGNLGRAVFQAKKRVLEPLGL (#35)
AAV8_VP1 111-119: GGNLGRAVF (#36)
Full
Empty
Deut
Time (sec)
10
100
1,000

AAV8_VP1 111-131: GGNLGRAVFQAKKRVLEPLGL (#37)

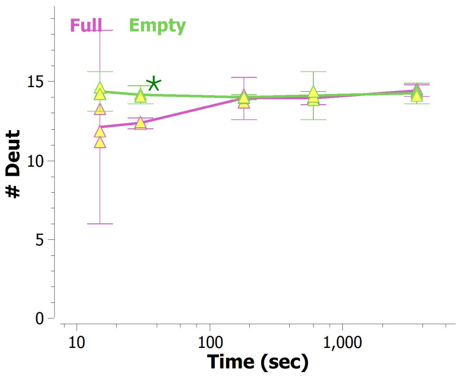

AAV8_VP1 120-131: QAKKRVLEPLGL (#38)

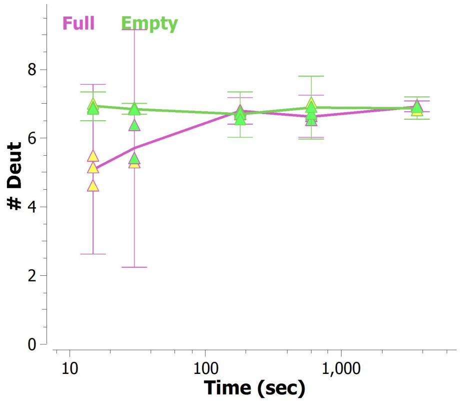

AAV8_VP1 205-229: AAGGGAPMADNNEGADGVGSSSGNW (#39)

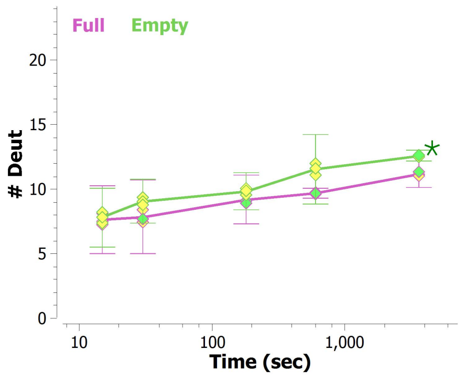

AAV8_VP1 205-238: APMADNNEGADGVGSSSGNWHCDST (#40)

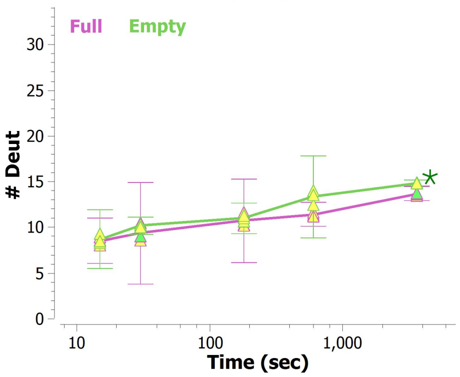

AAV8_VP1 215-229: NNEGADGVGSSSGNW (#41)

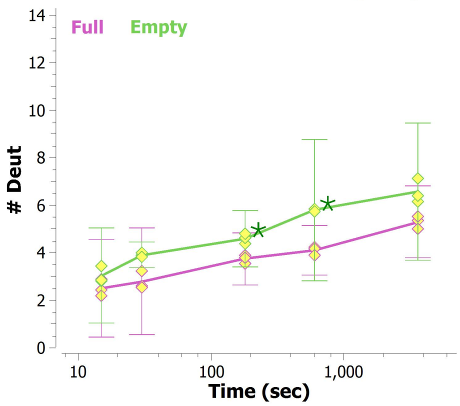

AAV8_VP1 229-238: WHCDSTWLGD (#42)

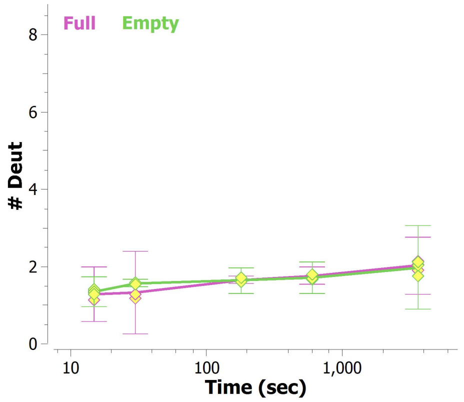

AAV8_VP1 230-236: HCDSTWL (#43)

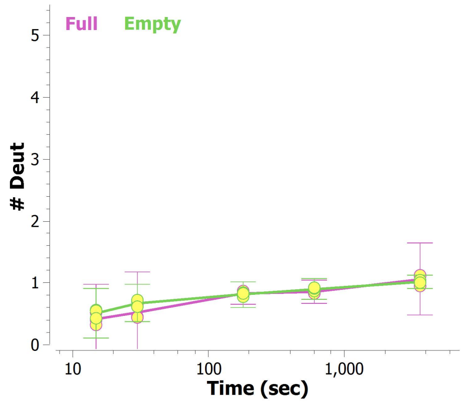

AAV8_VP1 230-238: HCDSTWLGD (#44)

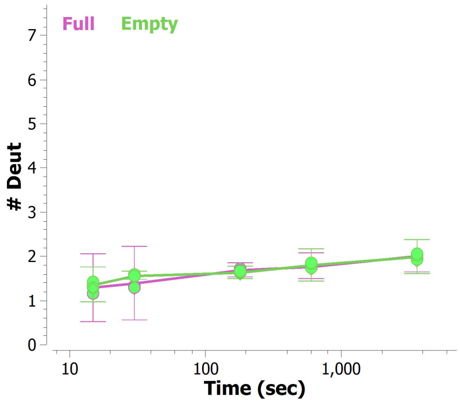

AAV8_VP1 230-240: HCDSTWLGDRV (#45)

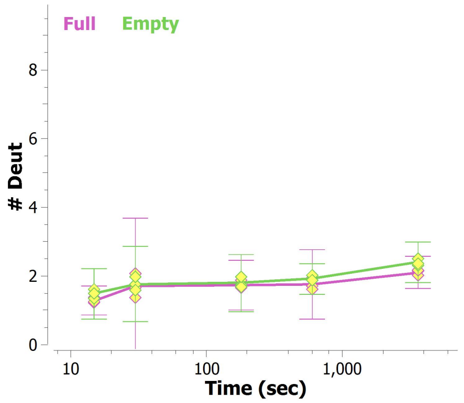

AAV8_VP1 230-242: HCDSTWLGDRVIT (#46)

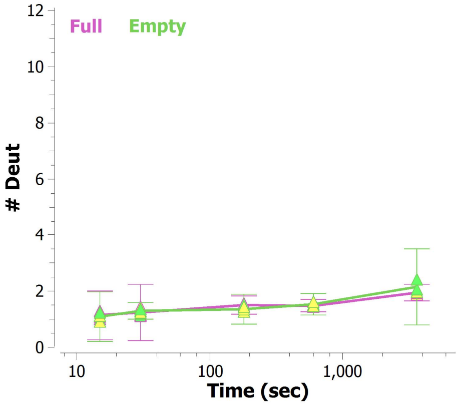

AAV8_VP1 230-243: HCDSTWLGDRVITT (#47)

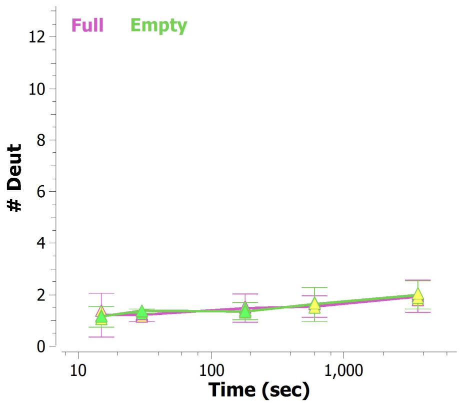

AAV8_VP1 230-247: HCDSTWLGDRVITTSTRT (#48)

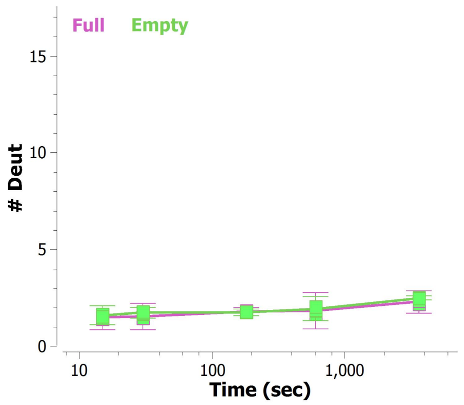

AAV8_VP1 230-257: CDSTWLGDRVITTSTRTWALPTYNNH (#49)

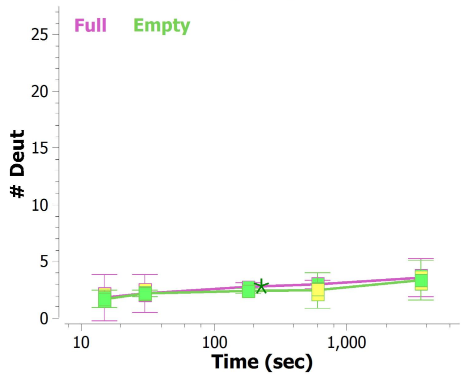

AAV8_VP1 232-243: DSTWLGDRVITT (#50)

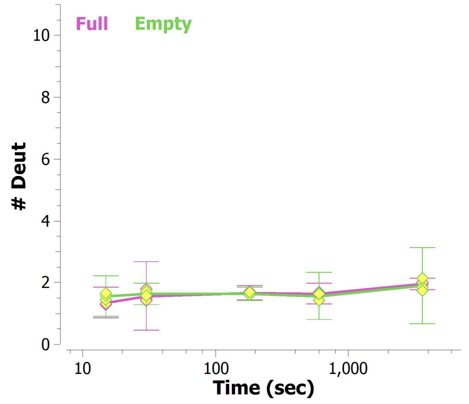

AAV8_VP1 232-247: DSTWLGDRVITTSTRT (#51)

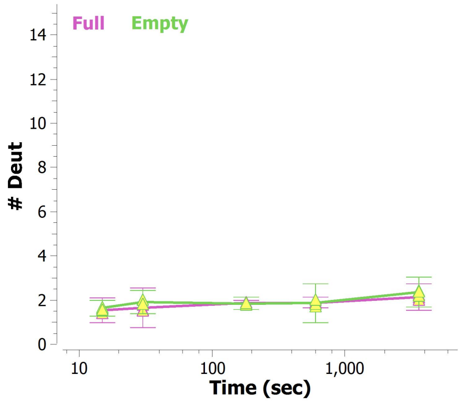

AAV8_VP1 234-238: TWLGD (#52)

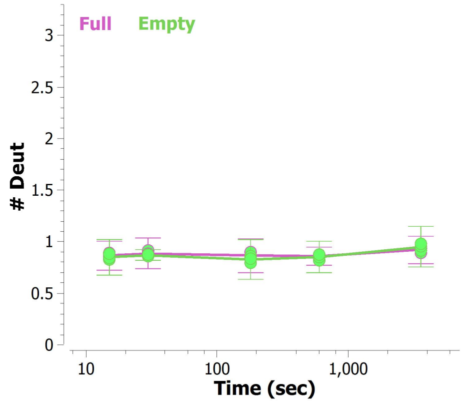

AAV8_VP1 234-247: TWLGDRVITTSTRT (#53)

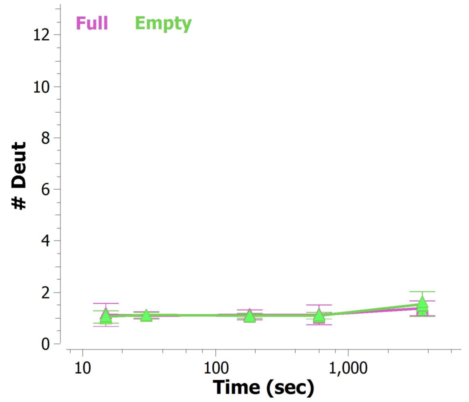

AAV8_VP1 235-247: WLGDRVITTSTRT (#54)

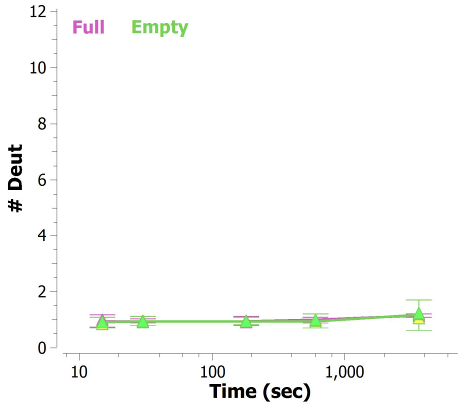

AAV8_VP1 236-247: LGDRVITTSTRT (#55)

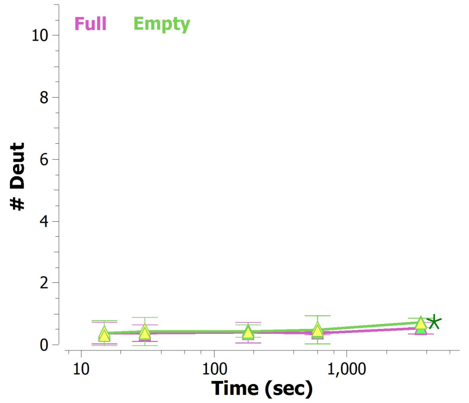

AAV8_VP1 236-257: LGDRVITTSTRTWALPTYNNHL (#56)

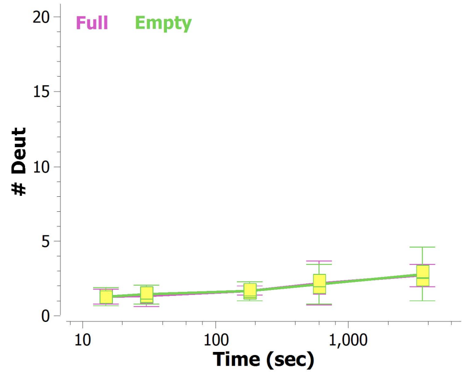

AAV8_VP1 237-247: GDRVITTSTRT (#57)

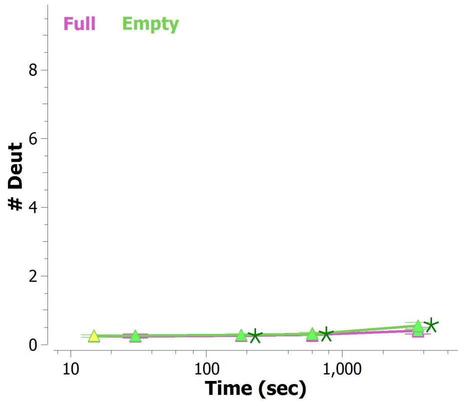

AAV8_VP1 237-257: GDRVITTSTRTWALPTYNNHL (#58)

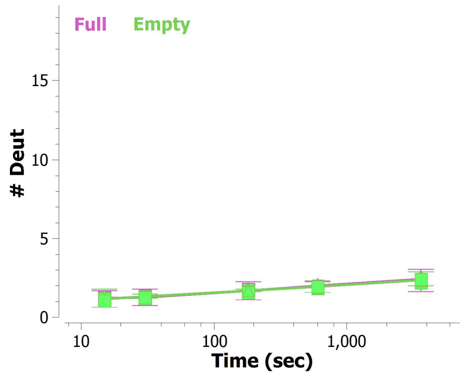

AAV8_VP1 238-247: DRVITTSTRT (#59)

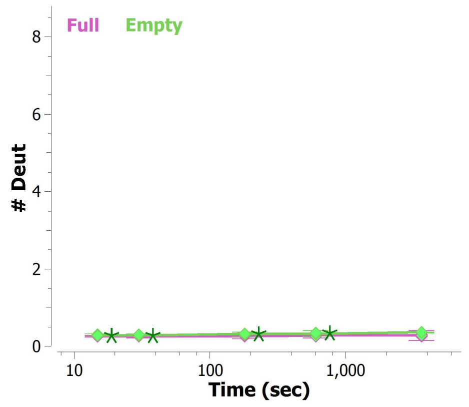

AAV8_VP1 239-243: RVITT (#60)

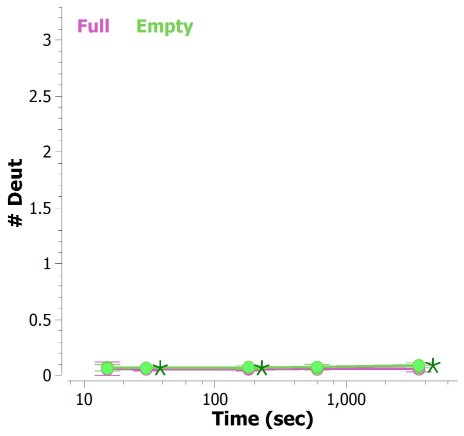

AAV8_VP1 239-245: RVITTST (#61)

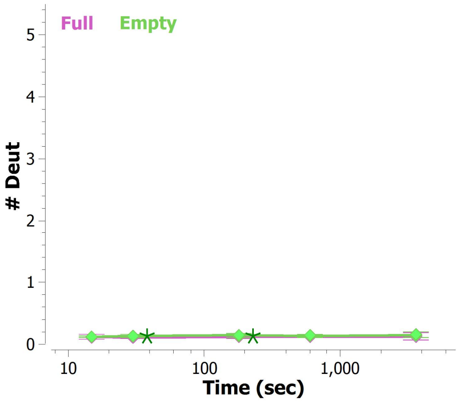

AAV8_VP1 239-247: RVITTSTRT (#62)

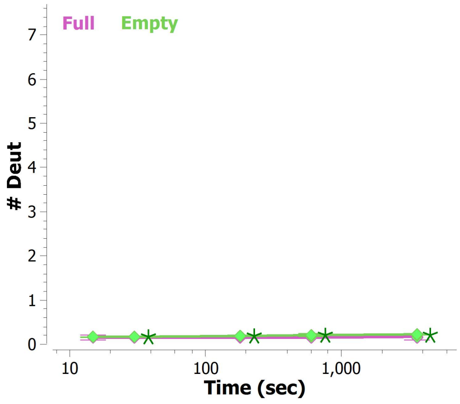

AAV8_VP1 239-250: RVITTSTRTWAL (#63)

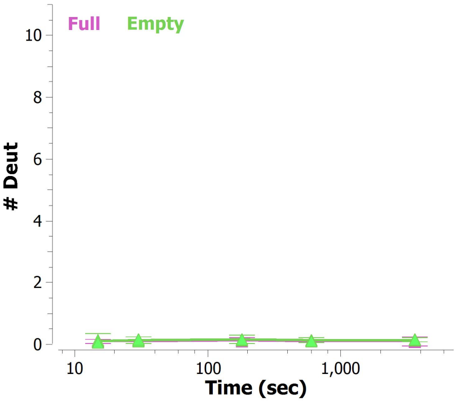

AAV8_VP1 239-257: RVITTSTRTWALPTYNNHL (#64)

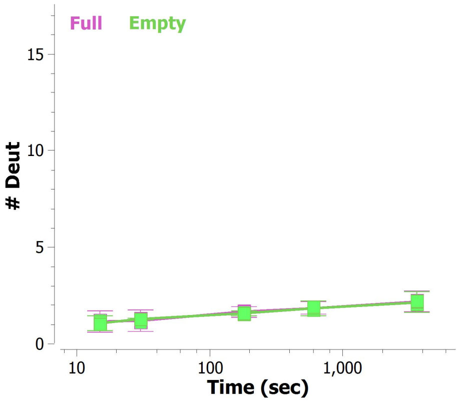

AAV8_VP1 241-257: ITTSTRTWALPTYNNHL (#65)

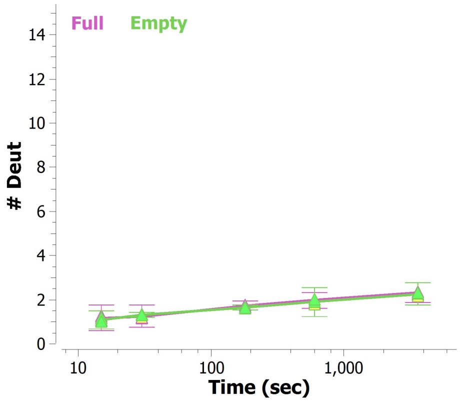

AAV8_VP1 243-247: TSTRT (#66)

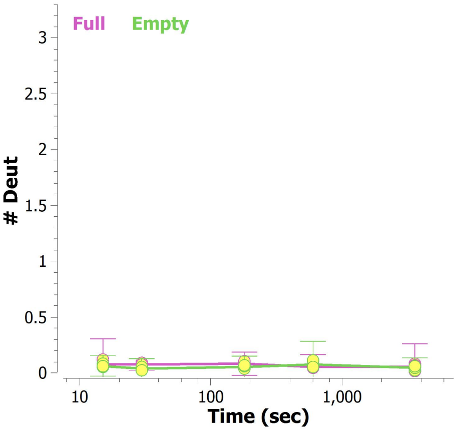

AAV8_VP1 243-257: TSTRTWALPTYNNHL (#67)

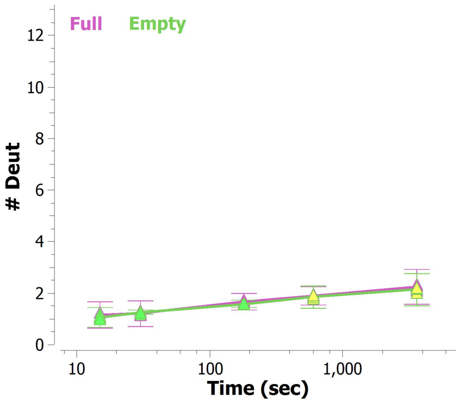

AAV8_VP1 244-257: STRTWALPTYNNHL (#68)

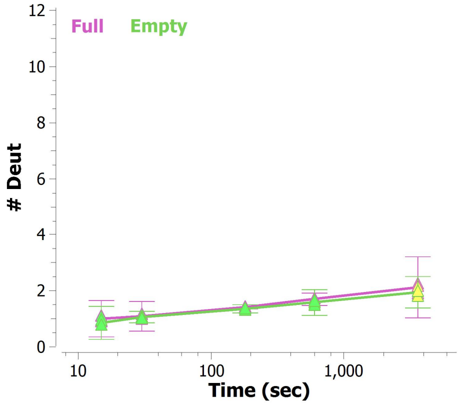

AAV8_VP1 245-257: TRTWALPTYNNHL (#69)

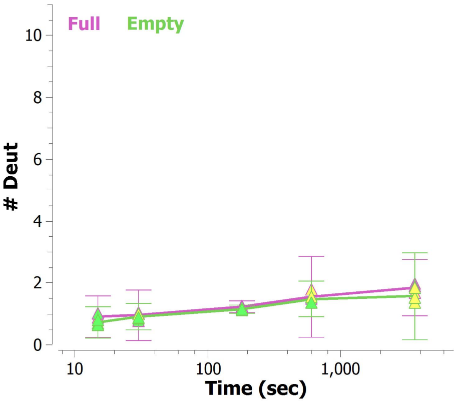

AAV8_VP1 246-257: RTWALPTYNNHL (#70)

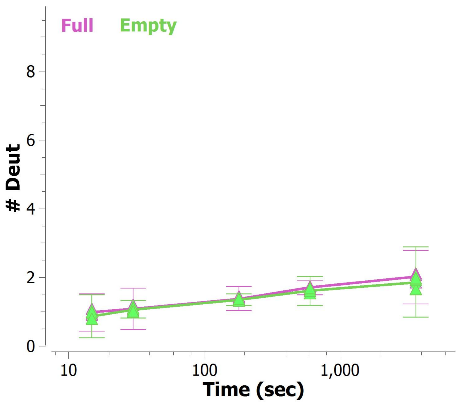

AAV8_VP1 248-257: WALPTYNNHL (#71)

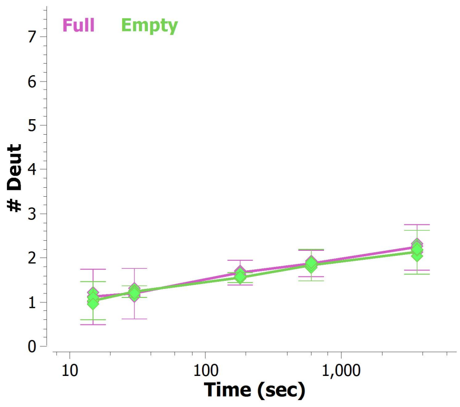

AAV8_VP1 250-257: LPTYNNHL (#72)

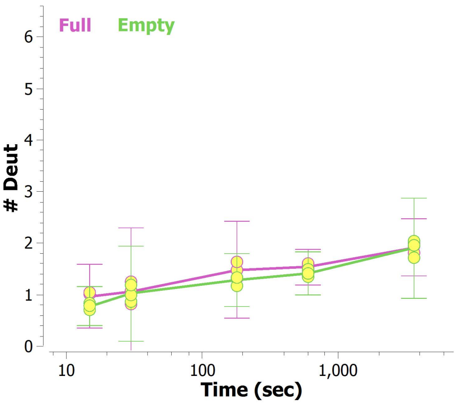

AAV8_VP1 251-257: PTYNNHL (#73)

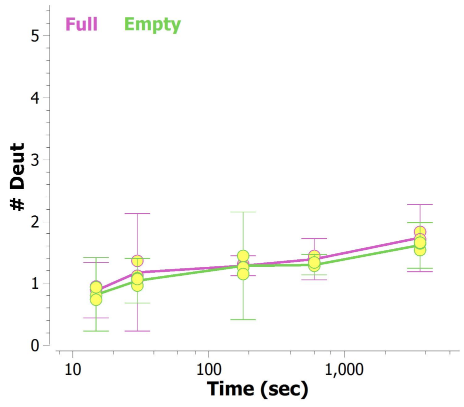

AAV8_VP1 258-264: YKQISNG (#74)

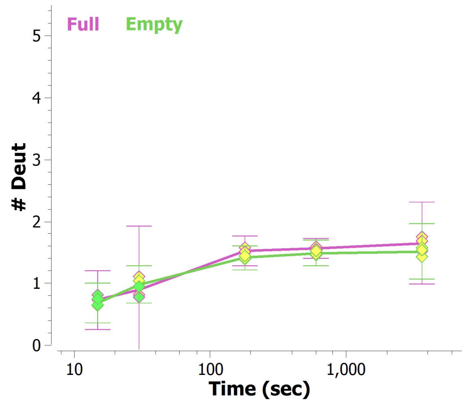

AAV8_VP1 258-266: YKQISNGTS (#75)

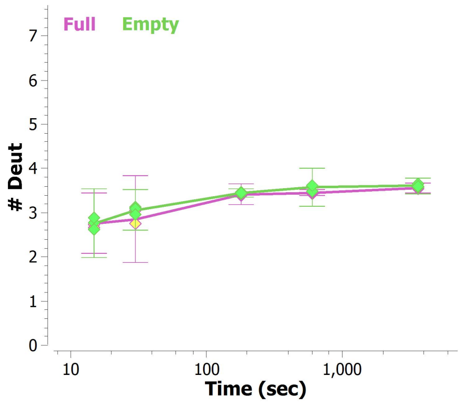

AAV8_VP1 258-275: YKQISNGTSGGATNDNTY (#76)

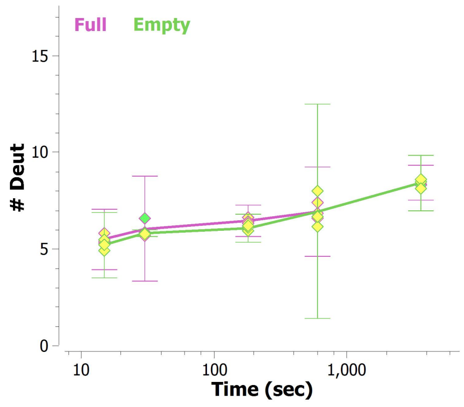

AAV8_VP1 277-285: GYSTPWGYF (#77)

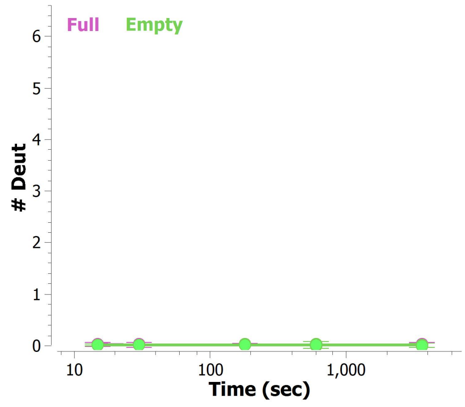

AAV8_VP1 278-284: YSTPWGY (#78)

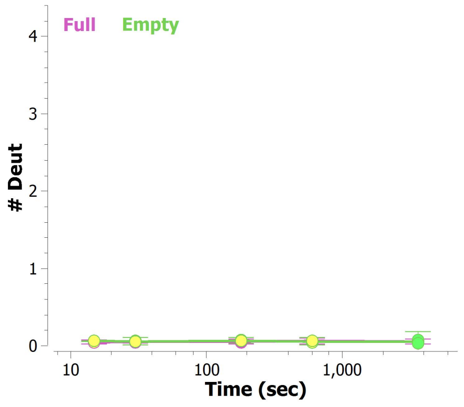

AAV8_VP1 278-285: YSTPWGYF (#79)

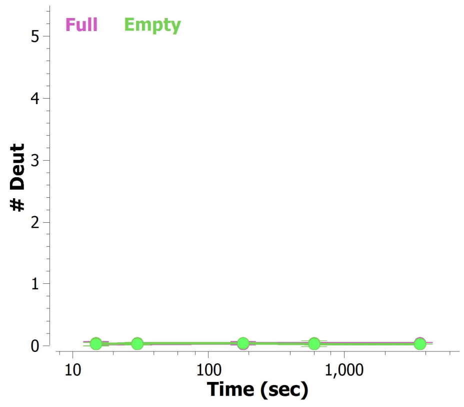

AAV8_VP1 278-286: YSTPWGYFD (#80)

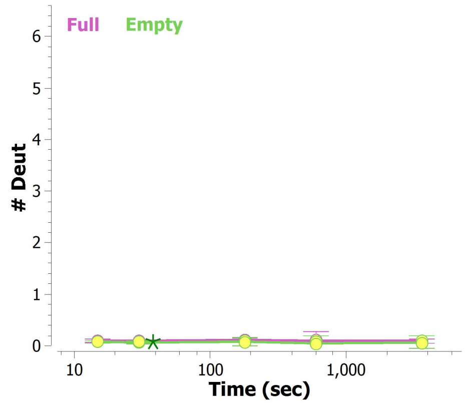

AAV8_VP1 286-290: DFNRF (#81)

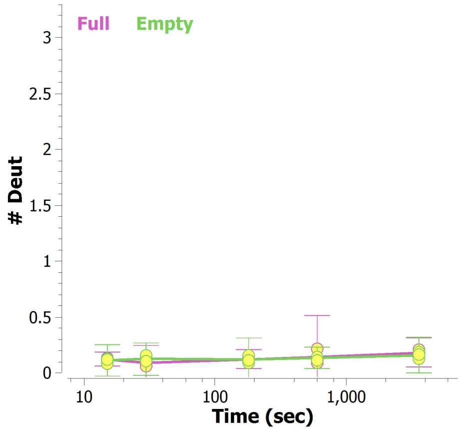

AAV8_VP1 286-294: DFNRFHCHF (#82)

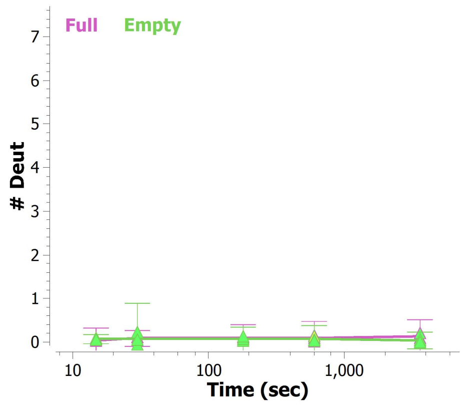

AAV8_VP1 286-298: DFNRFHCHFSPRD (#83)

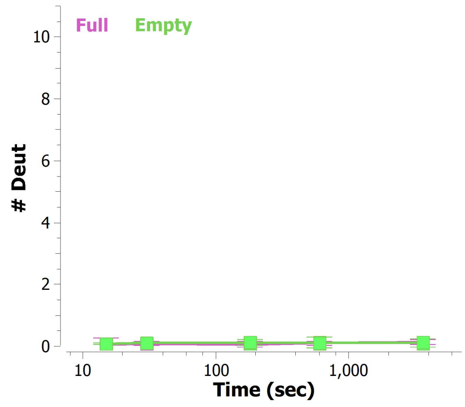

AAV8_VP1 286-299: DFNRFHCHFSPRDW (#84)

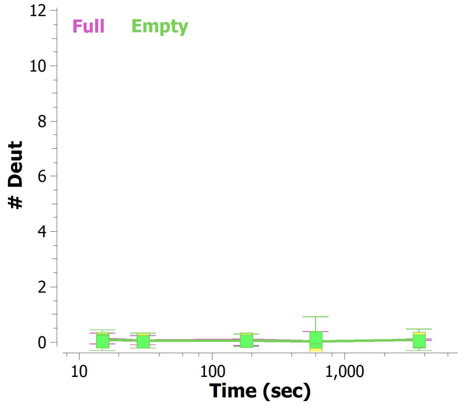

AAV8_VP1 286-306: DFNRFHCHFSPRDWQRLINNN (#85)

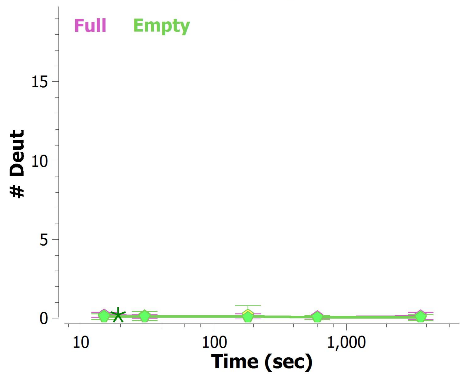

AAV8_VP1 291-298: HCHFSPRD (#86)

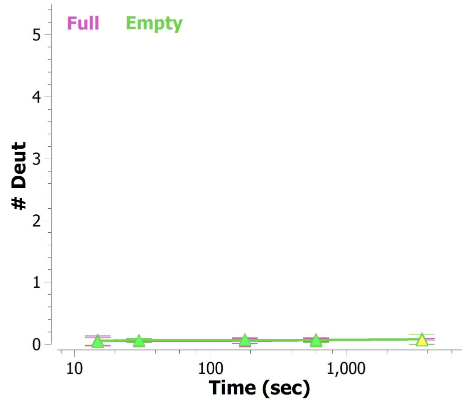

AAV8_VP1 291-299: HCHFSPRDW (#87)

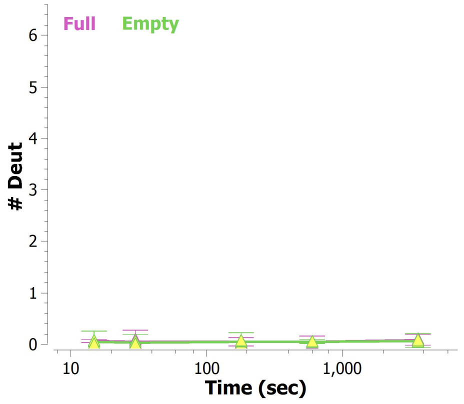

AAV8_VP1 291-302: HCHFSPRDWQRL (#88)

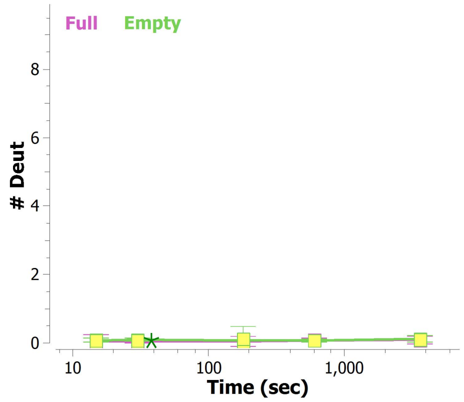

AAV8_VP1 299-306: WQRLINNN (#89)

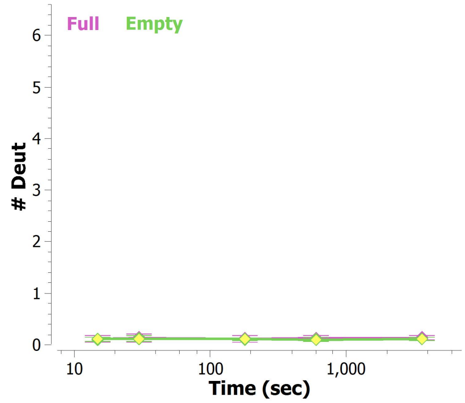

AAV8_VP1 299-307: WQRLINNNW (#90)

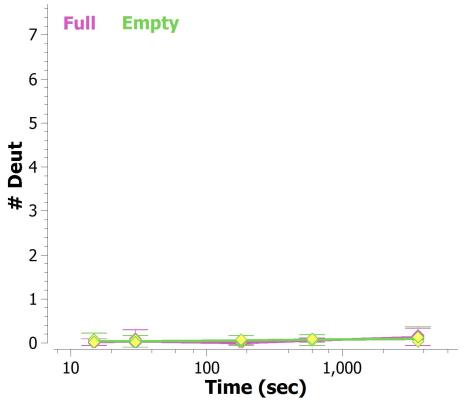

AAV8_VP1 303-316: INNNWGFRPKRLSF (#91)

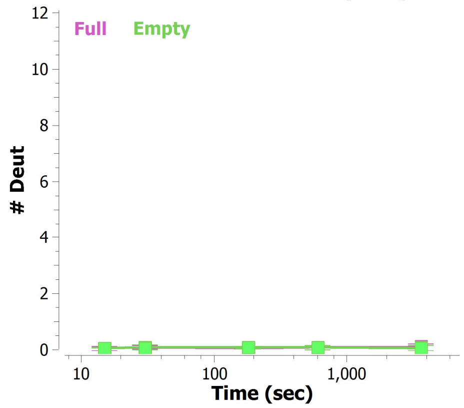

AAV8_VP1 303-318: INNNWGFRPKRLSFKL (#92)

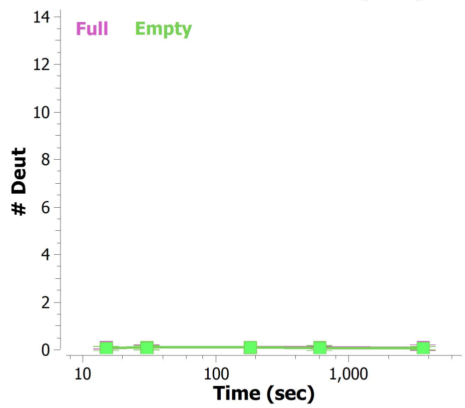

AAV8_VP1 317-320: KLFN (#93)

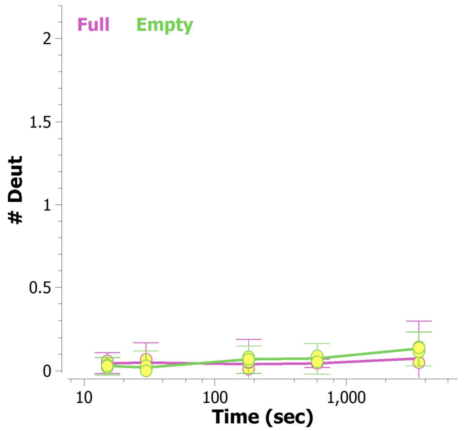

AAV8_VP1 317-322: KLFNIQ (#94)

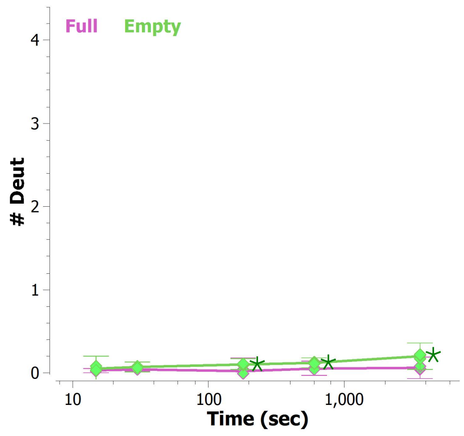

AAV8_VP1 317-323: KLFNIQV (#95)

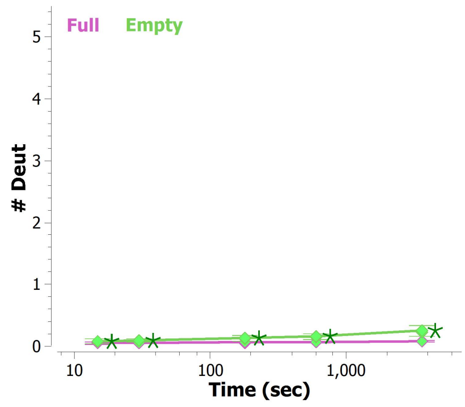

AAV8_VP1 317-325: KLFNIQVKE (#96)

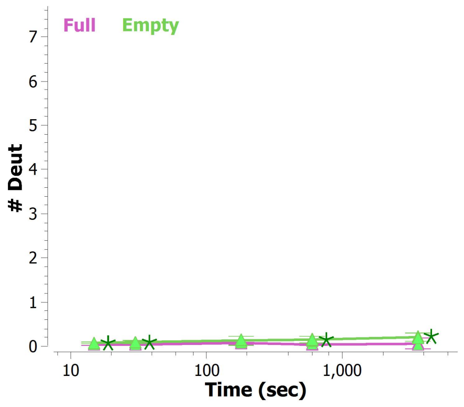

AAV8_VP1 317-338: KLFNIQVKEVTQNEGTKTIANN (#97)

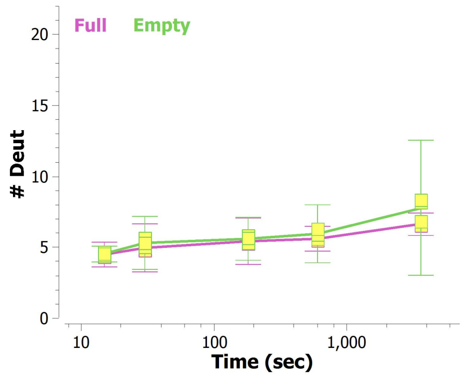

AAV8_VP1 317-339: KLFNIQVKEVTQNEGTKTIANNL (#98)

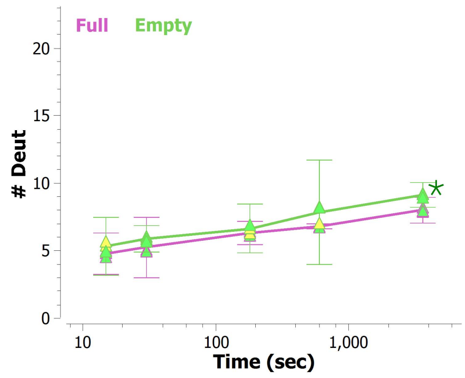

AAV8_VP1 317-341: KLFNIQVKEVTQNEGTKTIANNLTS (#99)

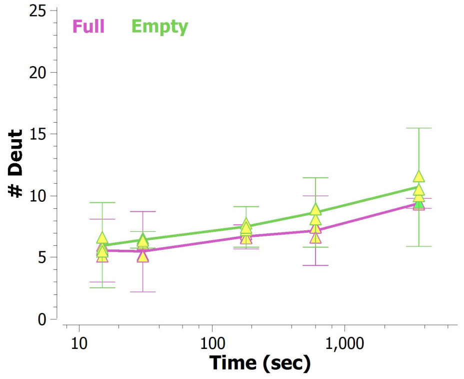

AAV8_VP1 317-342: KLFNIQVKEVTQNEGTKTIANNLTST (#100)

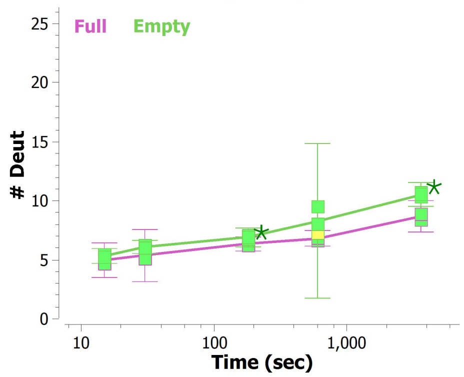

AAV8_VP1 317-345: LFNIQVKEVTQNEGTKTIANNLTSTIQ (#101)

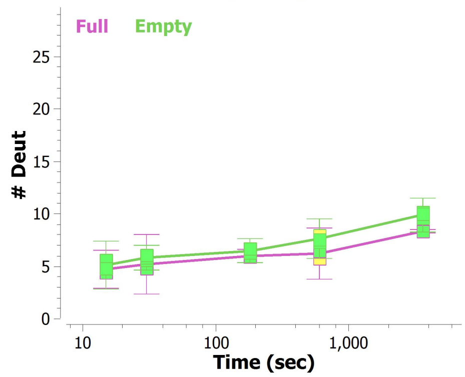

AAV8_VP1 319-325: FNIQVKE (#102)

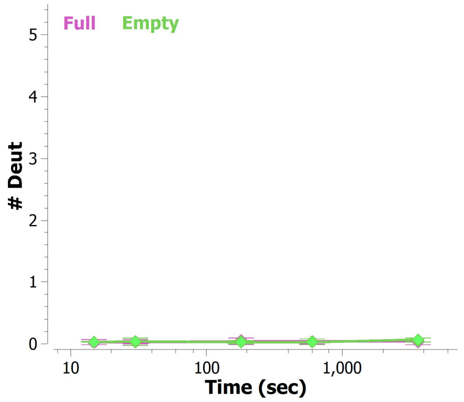

AAV8_VP1 319-339: FNIQVKEVTQNEGTKTIANNL (#103)

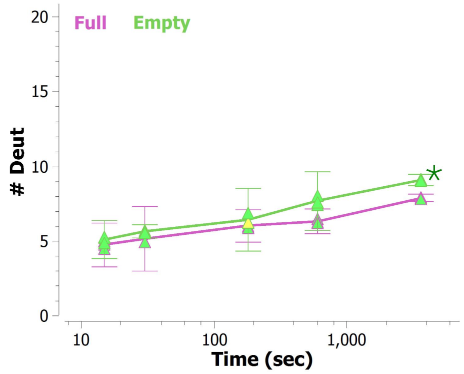

AAV8_VP1 319-341: FNIQVKEVTQNEGTKTIANNLTS (#104)

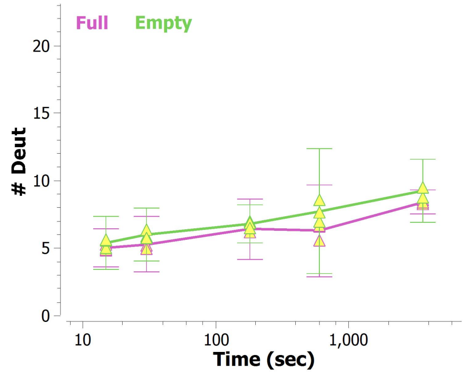

AAV8_VP1 319-342: FNIQVKEVTQNEGTKTIANNLTST (#105)

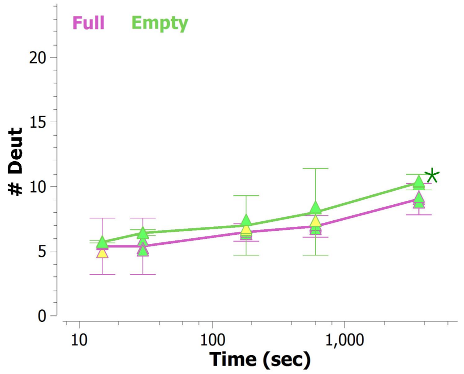

AAV8_VP1 319-344: FNIQVKEVTQNEGTKTIANNLTSTIQ (#106)

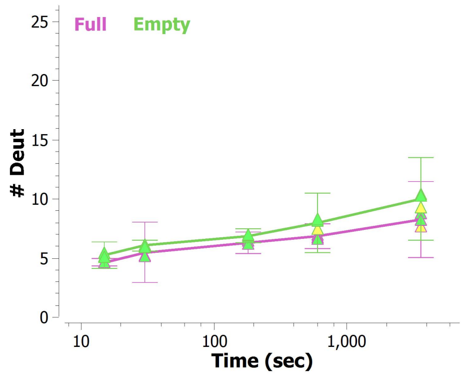

AAV8_VP1 319-345: FNIQVKEVTQNEGTKTIANNLTSTIQV (#107)

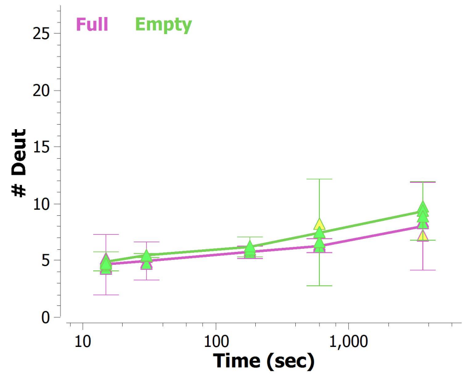

AAV8_VP1 320-339: NIQVKEVTQNEGTKTIANNL (#108)

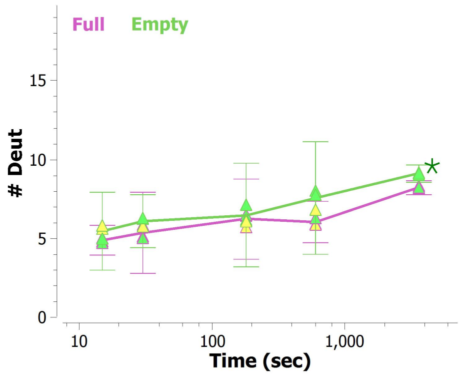

AAV8_VP1 320-342: NIQVKEVTQNEGTKTIANNLTST (#109)

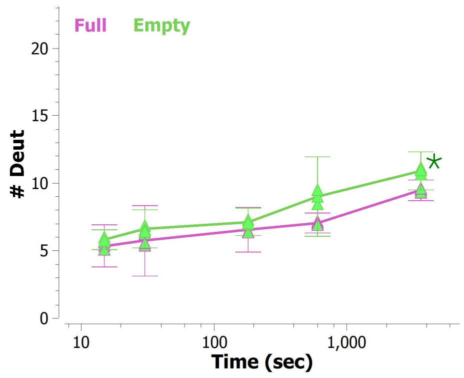

AAV8_VP1 320-345: NIQVKEVTQNEGTKTIANNLTSTIQV (#110)

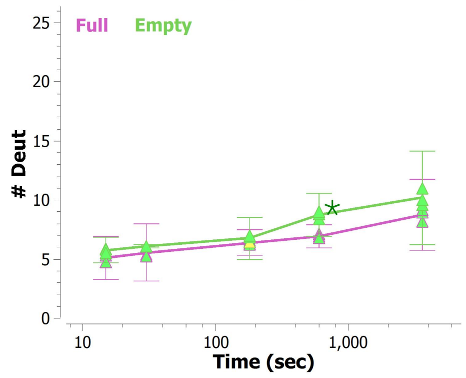

AAV8_VP1 320-346: NIQVKEVTQNEGTKTIANNLTSTIQVF (#111)

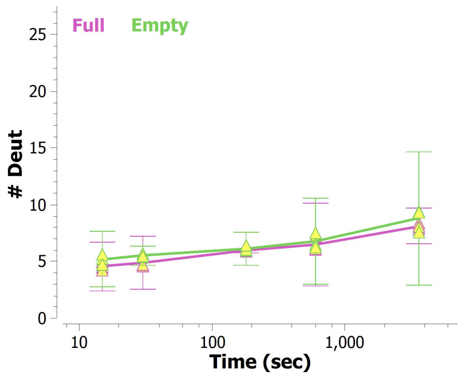

AAV8_VP1 320-350: QVKEVTQNEGTKTIANNLTSTIQVFTD (#112)

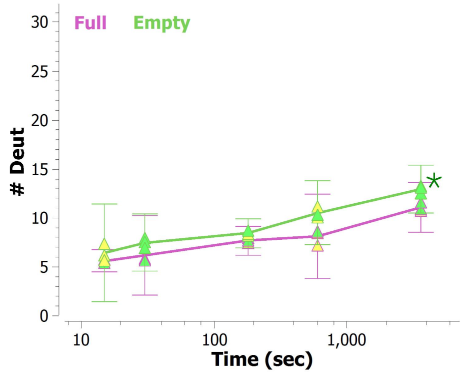

AAV8_VP1 321-342: IQVKEVTQNEGTKTIANNLTST (#113)

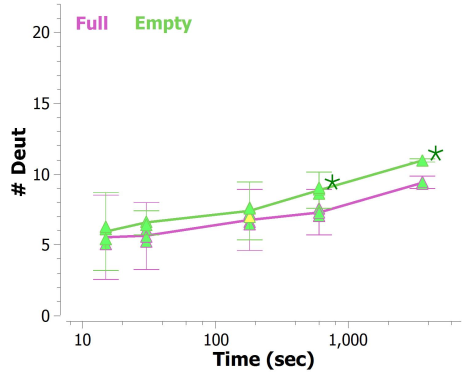

AAV8_VP1 321-345: IQVKEVTQNEGTKTIANNLTSTIQV (#114)

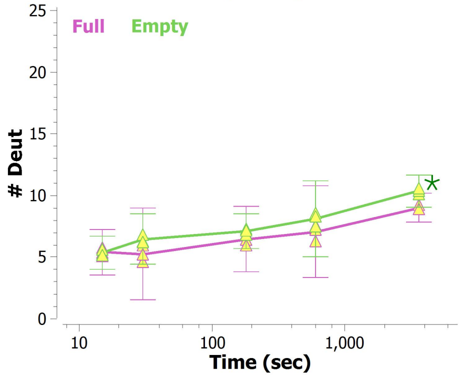

AAV8_VP1 323-339: VKEVTQNEGTKTIANNL (#115)

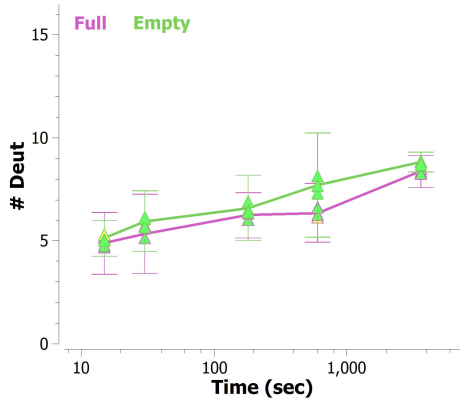

AAV8_VP1 323-342: VKEVTQNEGTKTIANNLTST (#116)

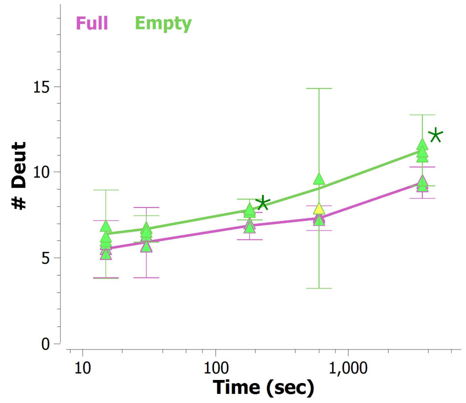

AAV8_VP1 323-344: VKEVTQNEGTKTIANNLTSTIQ (#117)

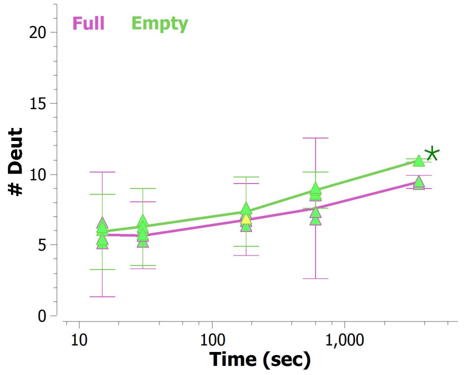

AAV8_VP1 323-345: VKEVTQNEGTKTIANNLTSTIQV (#118)

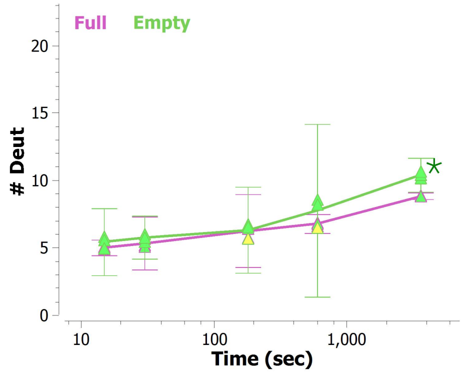

AAV8_VP1 323-346: VKEVTQNEGTKTIANNLTSTIQVF (#119)

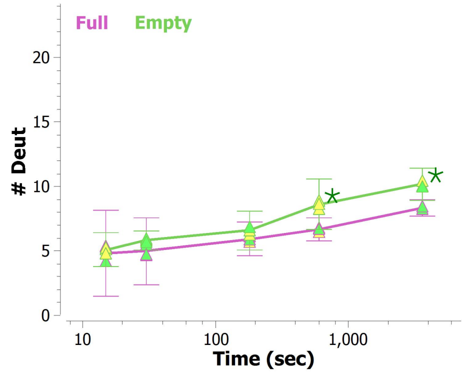

AAV8_VP1 323-350: VKEVTQNEGTKTIANNLTSTIQVFTDSE (#120)

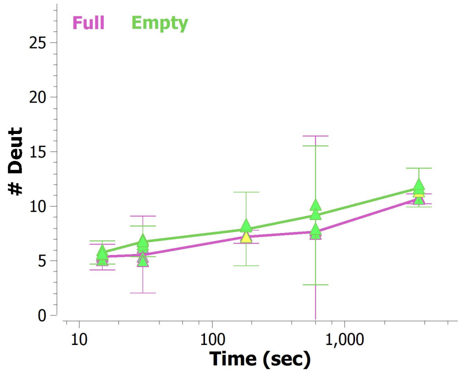

AAV8_VP1 324-339: KEVTQNEGTKTIANNL (#121)

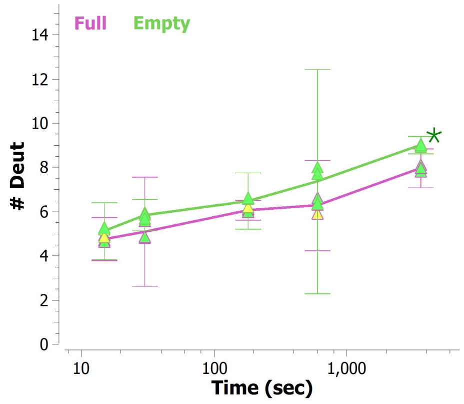

AAV8_VP1 324-342: KEVTQNEGTKTIANNLTST (#122)

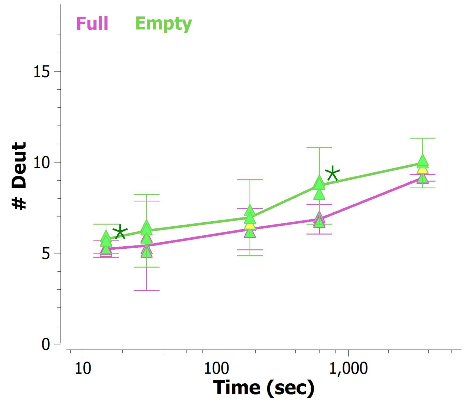

AAV8_VP1 324-345: KEVTQNEGTKTIANNLTSTIQV (#123)

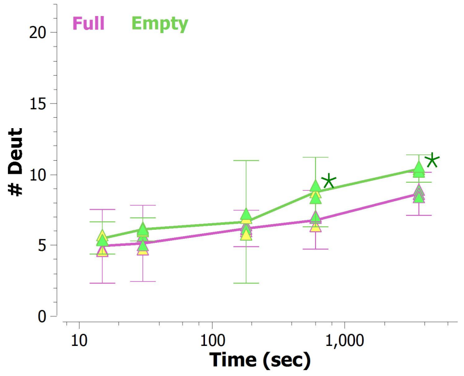

AAV8_VP1 324-346: KEVTQNEGTKTIANNLTSTIQVF (#124)

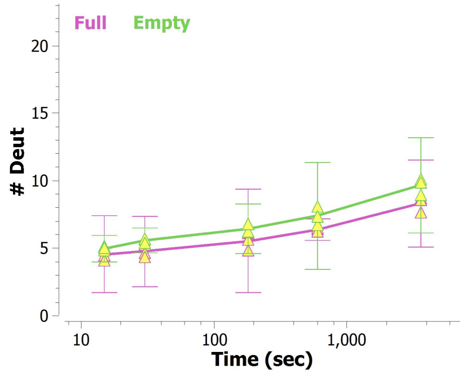

AAV8_VP1 326-345: VTQNEGTKTIANNLTSTIQV (#125)

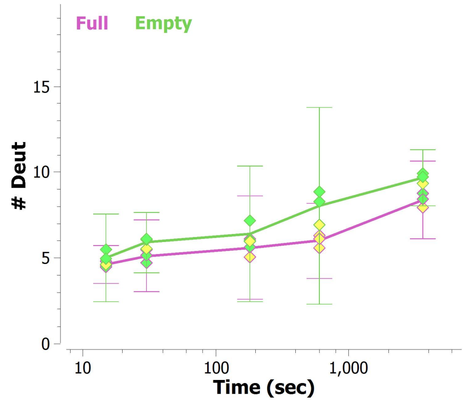

AAV8_VP1 326-350: VTQNEGTKTIANNLTSTIQVFTDSE (#126)

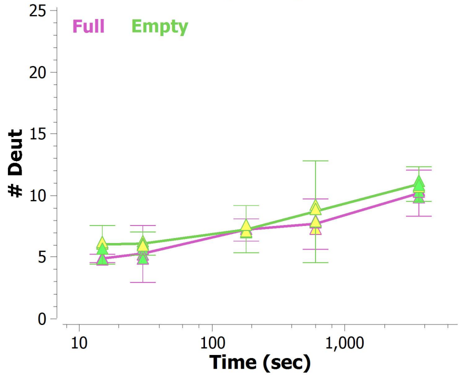

AAV8_VP1 330-342: EGTKTIANNLTST (#127)

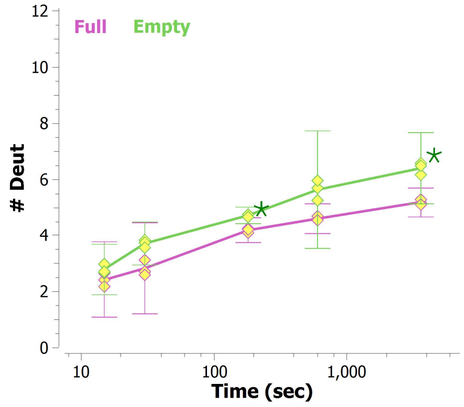

AAV8_VP1 335-339: IANNL (#128)

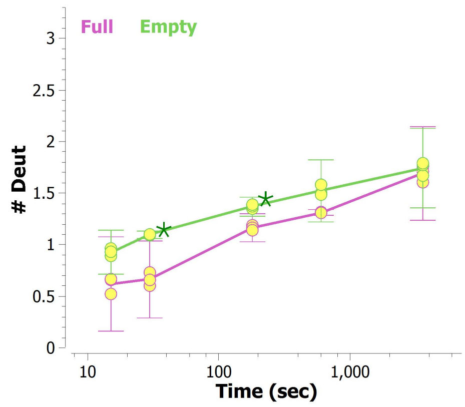

AAV8_VP1 340-345: TSTIQV (#129)

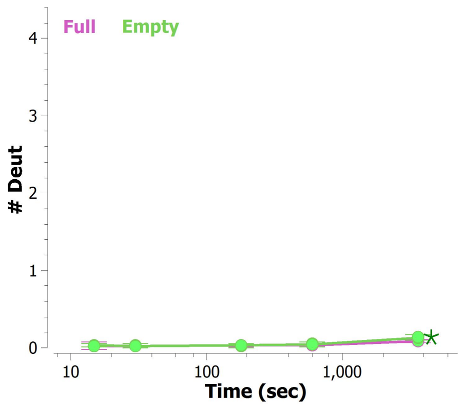

AAV8_VP1 340-346: TSTIQVF (#130)

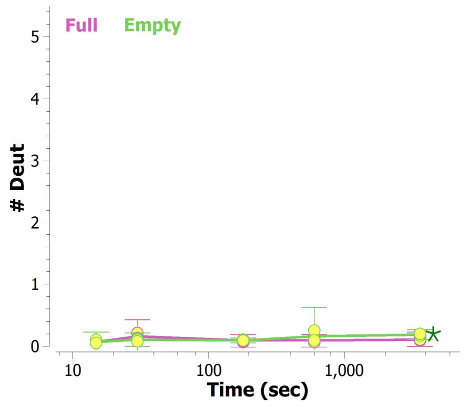

AAV8_VP1 340-350: TSTIQVFTDSE (#131)

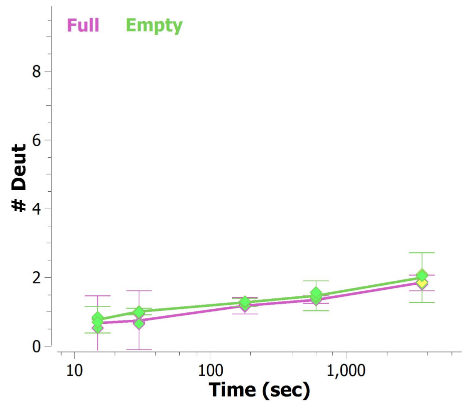

AAV8_VP1 343-346: IQVF (#132)

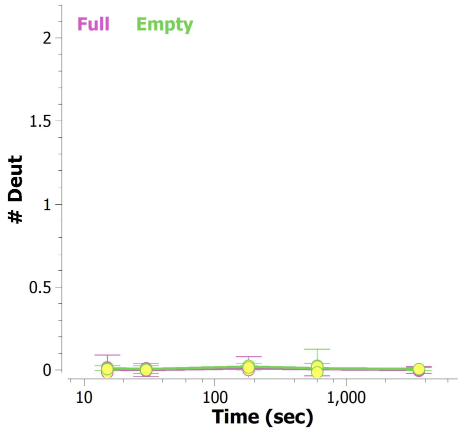

AAV8_VP1 343-347: IQVFT (#133)

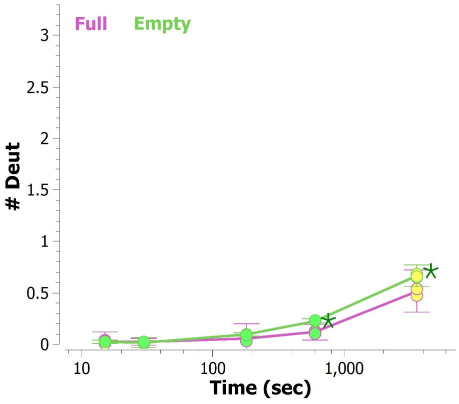

AAV8_VP1 343-349: IQVFTDS (#134)

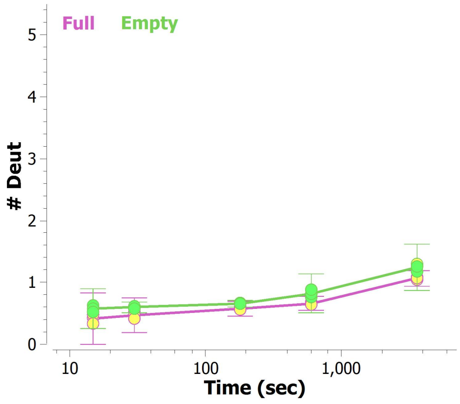

AAV8_VP1 343-350: IQVFTDSE (#135)

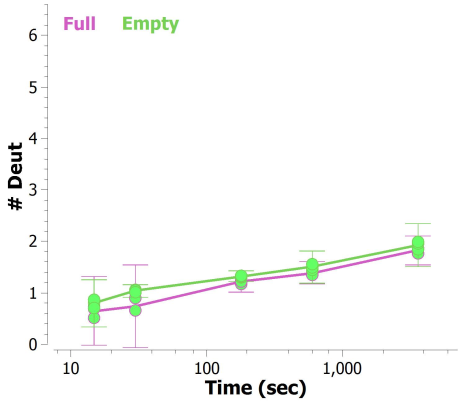

AAV8_VP1 351-357: YQLPYVL (#136)

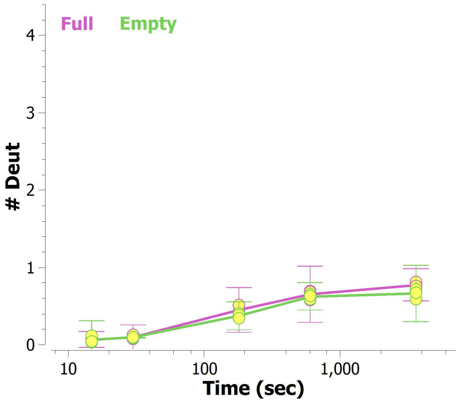

AAV8_VP1 351-358: YQLPYVLG (#137)

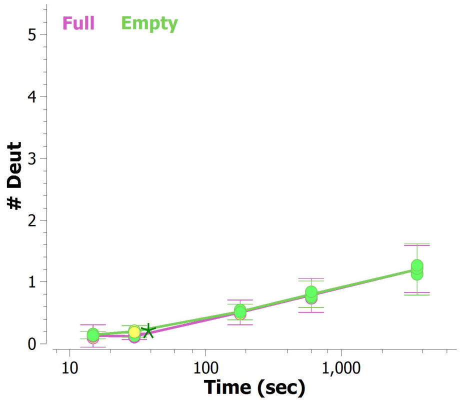

AAV8_VP1 351-360: YQLPYVLGSA (#138)

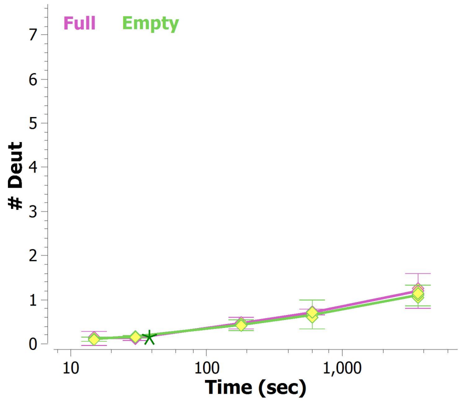

AAV8_VP1 351-371: YQLPYVLGSAHQGCLPPFPAD (#139)

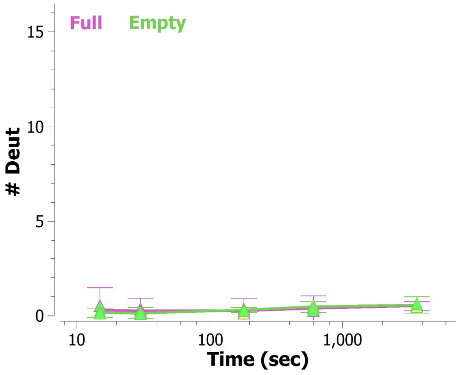

AAV8_VP1 351-373: YQLPYVLGSAHQGCLPPFPADVF (#140)

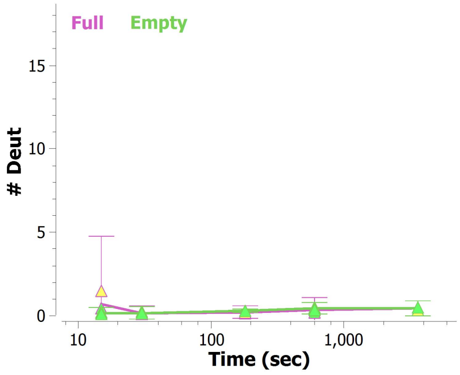

AAV8_VP1 372-379: VFMIPQYG (#141)

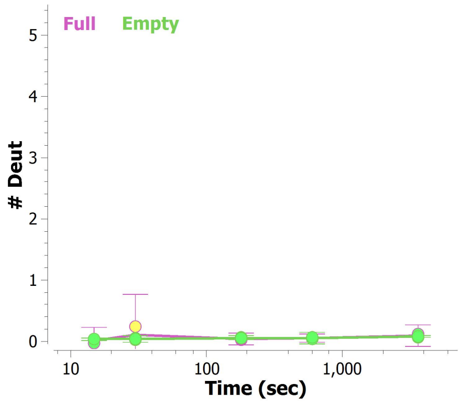

AAV8_VP1 372-380: VFMIPQYGY (#142)

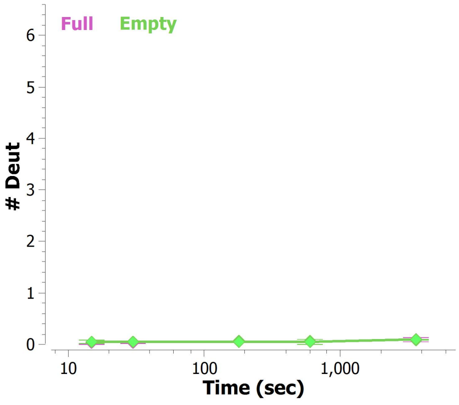

AAV8_VP1 372-381: VFMIPQYGYL (#143)

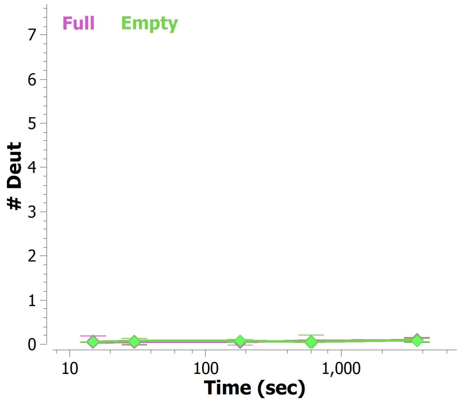

AAV8_VP1 373-379: FMIPQYG (#144)

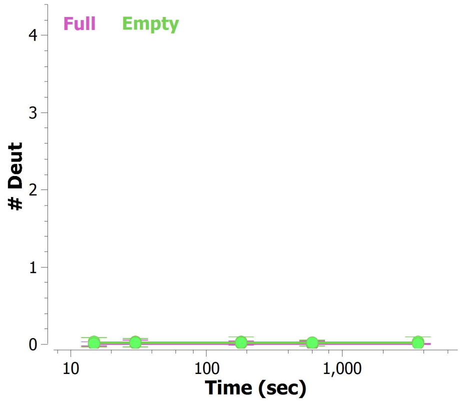

AAV8_VP1 373-380: FMIPQYGY (#145)

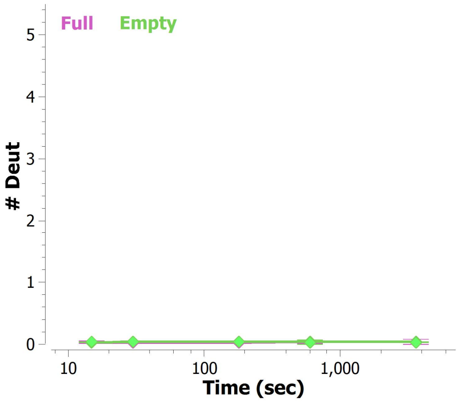

AAV8_VP1 373-381: FMIPQYGYL (#146)

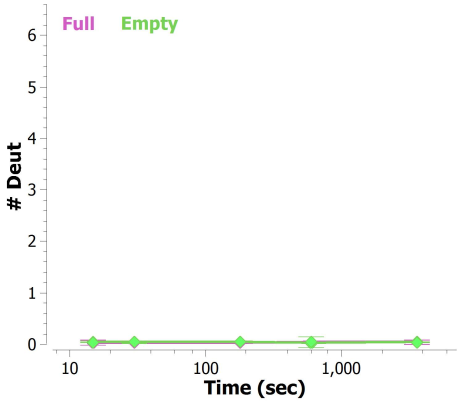

AAV8_VP1 373-388: FMIPQYGYLTLNNGSQ (#147)

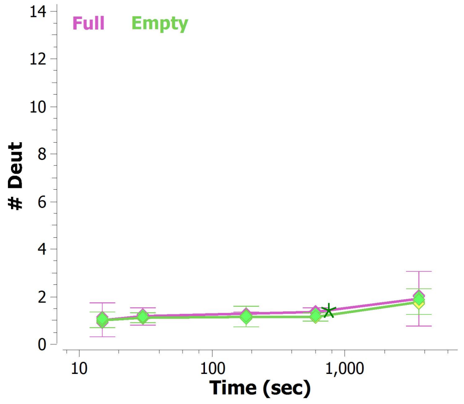

AAV8_VP1 374-380: MIPQYGY (#148)

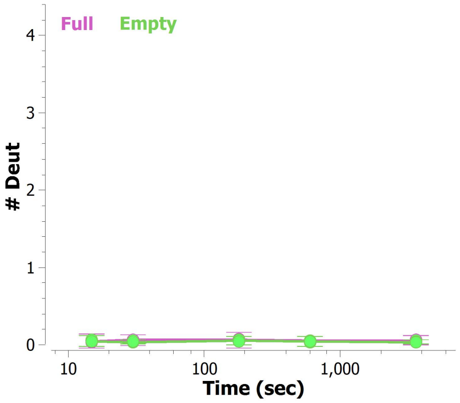

AAV8_VP1 374-381: MIPQYGYL (#149)

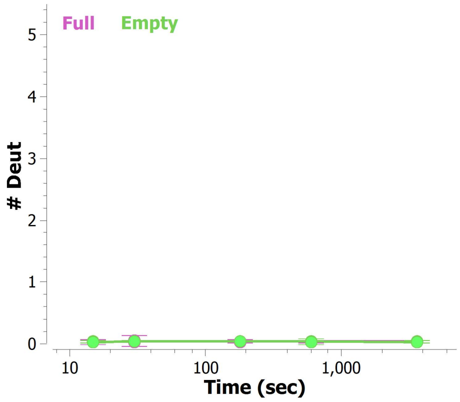

AAV8_VP1 374-383: MIPQYGYLTL (#150)

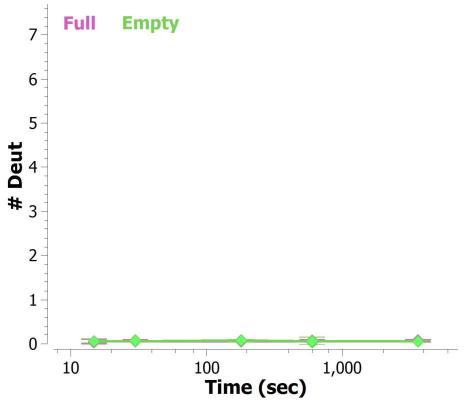

AAV8_VP1 374-388: MIPQYGYLTLNNGSQ (#151)

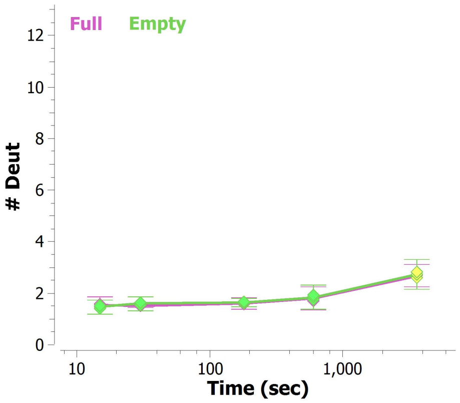

AAV8_VP1 374-395: MIPQYGYLTLNNGSQAVGRSSF (#152)

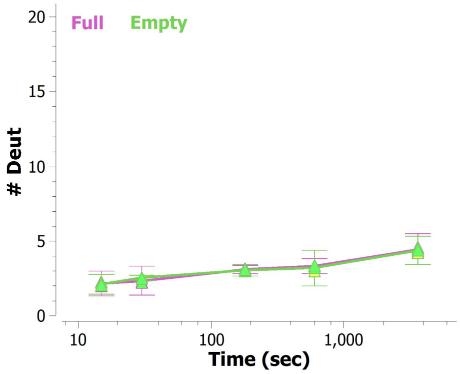

AAV8_VP1 375-383: IPQYGYLTL (#153)

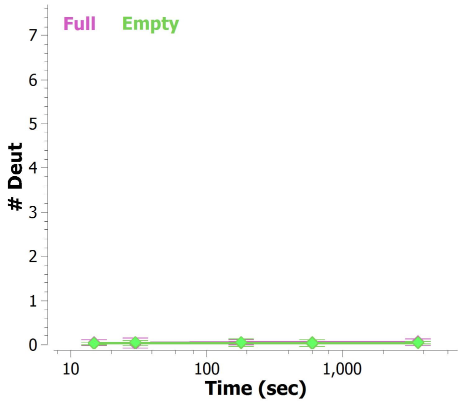

AAV8_VP1 375-395: IPQYGYLTLNNGSQAVGRSSF (#154)

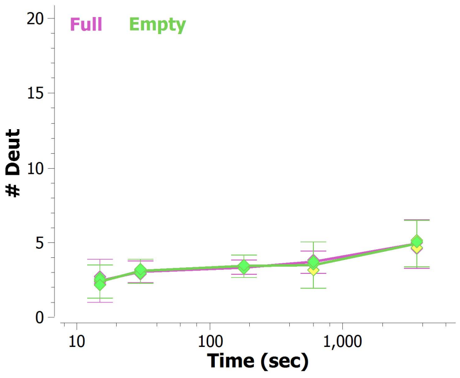

AAV8_VP1 376-380: PQYGY (#155)

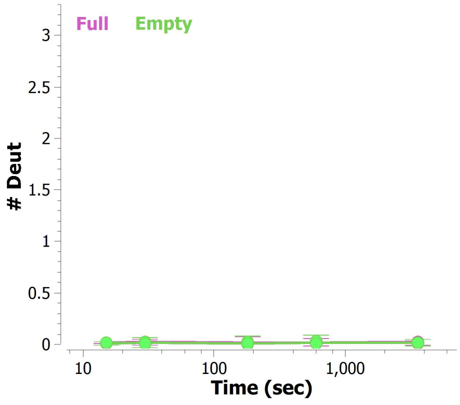

AAV8_VP1 380-395: YLTLNNGSQAVGRSSF (#156)

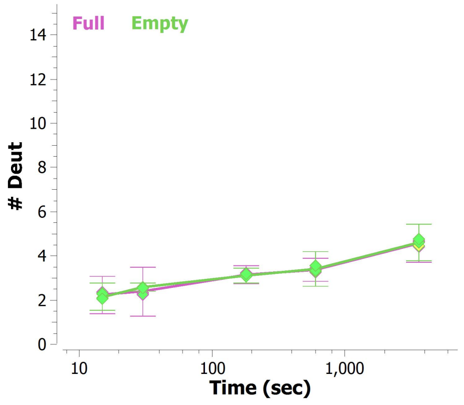

AAV8_VP1 381-388: LTLNNGSQ (#157)

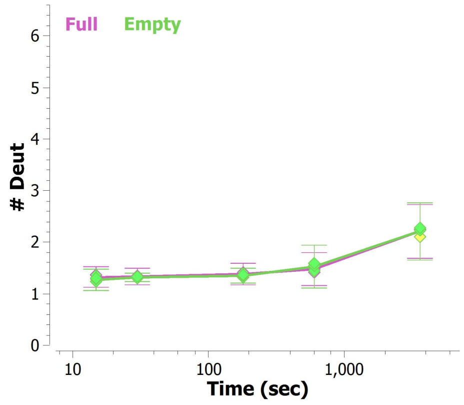

AAV8_VP1 381-395: LTLNNGSQAVGRSSF (#158)

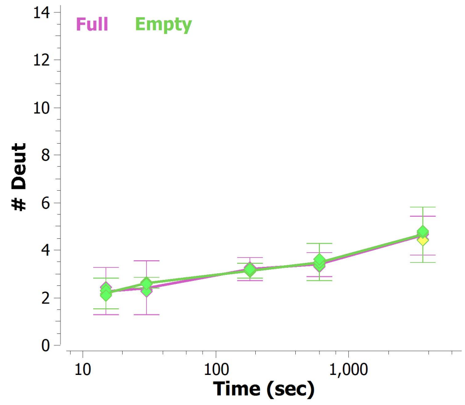

AAV8_VP1 382-388: TLNNGSQ (#159)

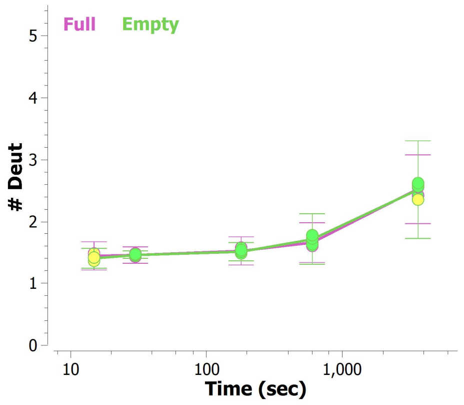

AAV8_VP1 382-395: TLNNGSQAVGRSSF (#160)

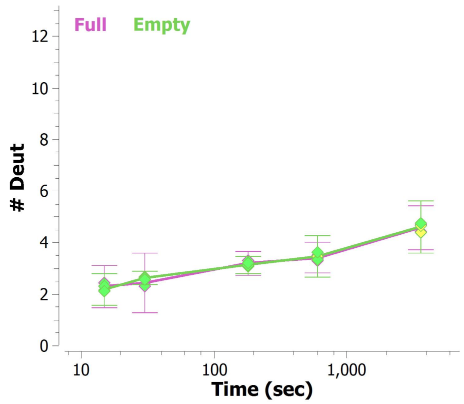

AAV8_VP1 384-395: NNGSQAVGRSSF (#161)

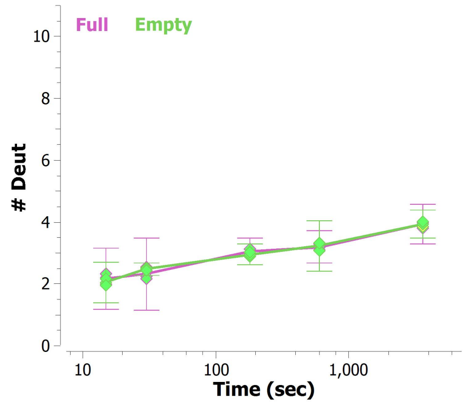

AAV8_VP1 389-395: AVGRSSF (#162)

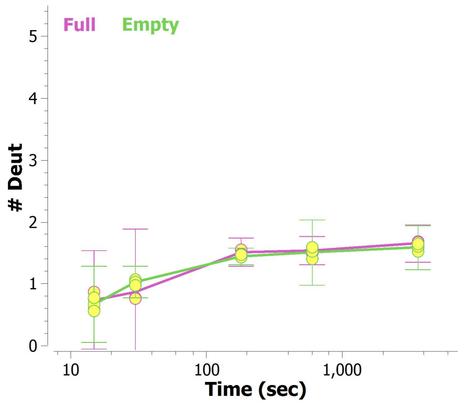

AAV8_VP1 390-395: VGRSSF (#163)

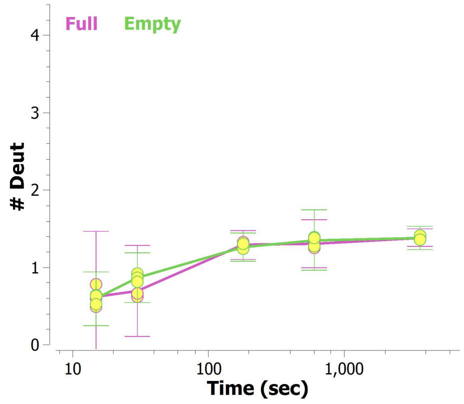

AAV8_VP1 396-399: YCLE (#164)

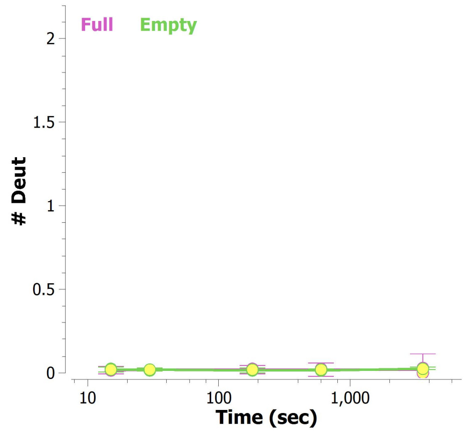

AAV8_VP1 396-405: YCLEYFPSQM (#165)

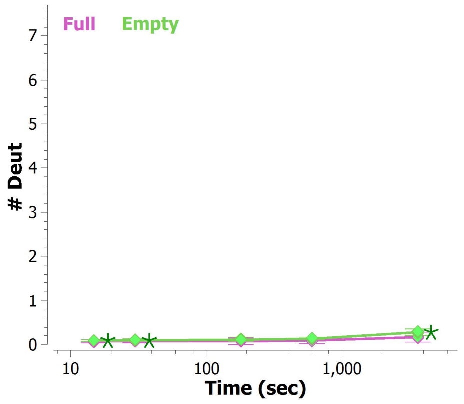

AAV8_VP1 396-411: YCLEYFPSQMLRTGNN (#166)

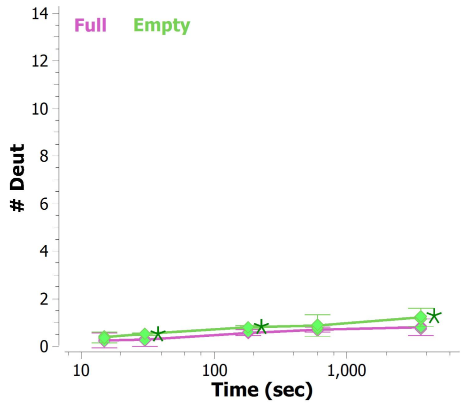

AAV8_VP1 397-405: CLEYFPSQM (#167)

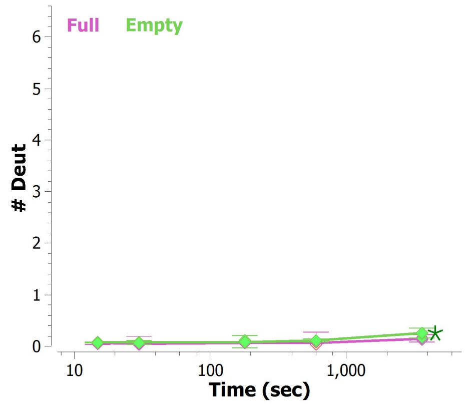

AAV8_VP1 399-404: EYFPSQ (#168)

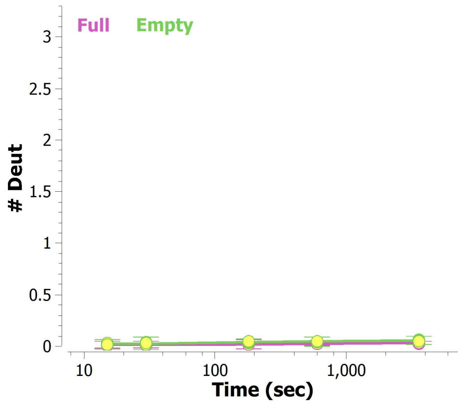

AAV8_VP1 399-405: EYFPSQM (#169)

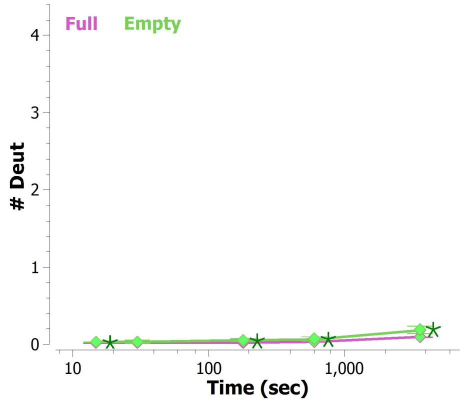

AAV8_VP1 399-411: EYFPSQMLRTGNN (#170)

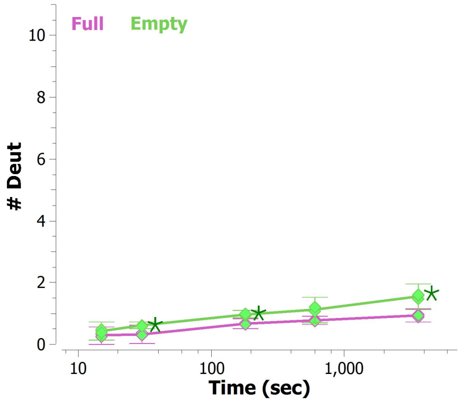

AAV8_VP1 400-405: YFPSQM (#171)

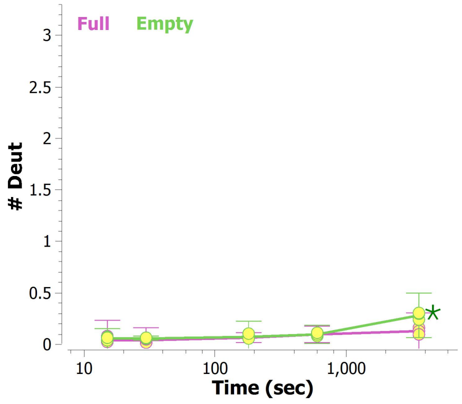

AAV8_VP1 405-411: MLRTGNN (#172)

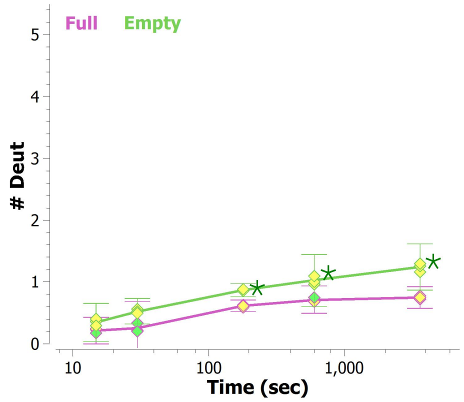

AAV8_VP1 405-413: MLRTGNNFQ (#173)

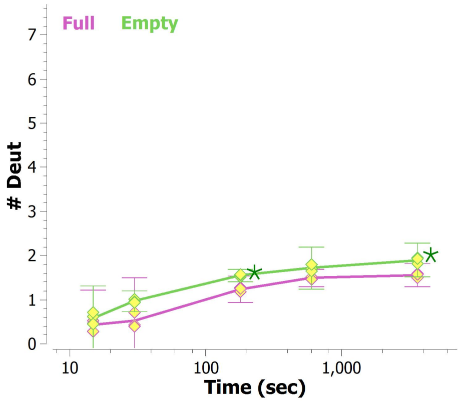

AAV8_VP1 406-411: LRTGNN (#174)

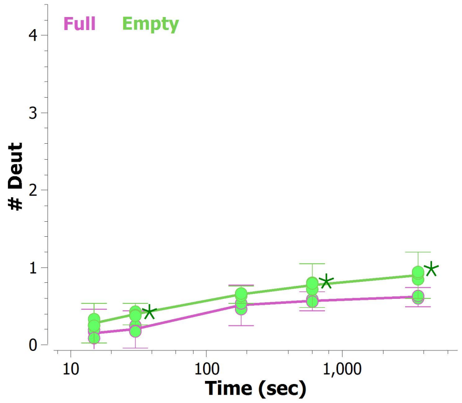

AAV8_VP1 406-412: LRTGNNF (#175)

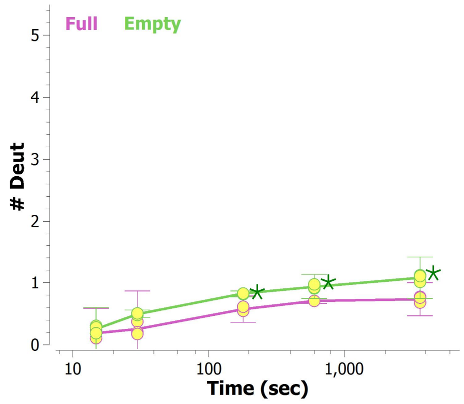

AAV8_VP1 406-413: LRTGNNFQ (#176)

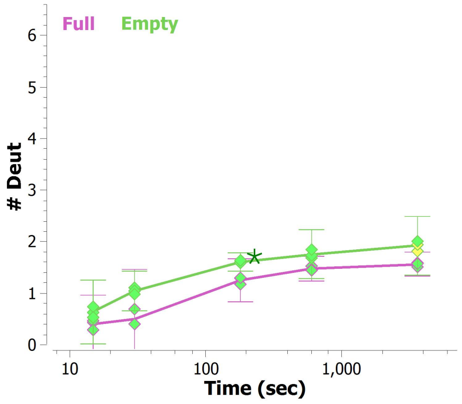

AAV8_VP1 406-415: LRTGNNFQFT (#177)

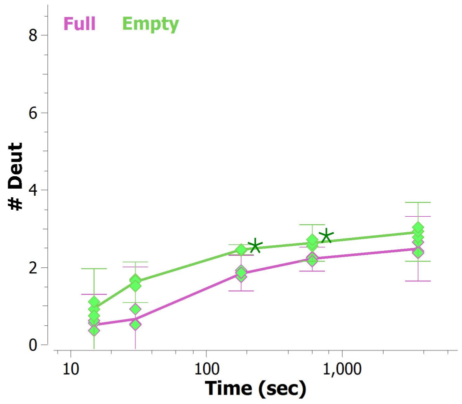

AAV8_VP1 406-417: LRTGNNFQFTYT (#178)

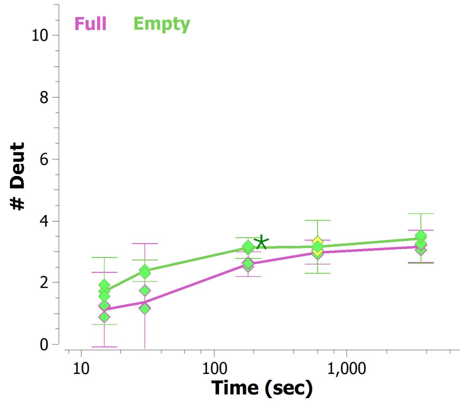

AAV8_VP1 412-417: FQFTYT (#179)

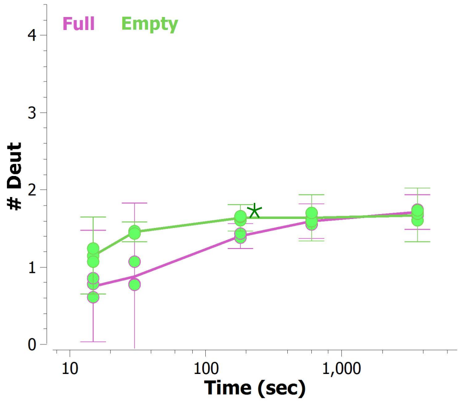

AAV8_VP1 412-418: FQFTYTF (#180)

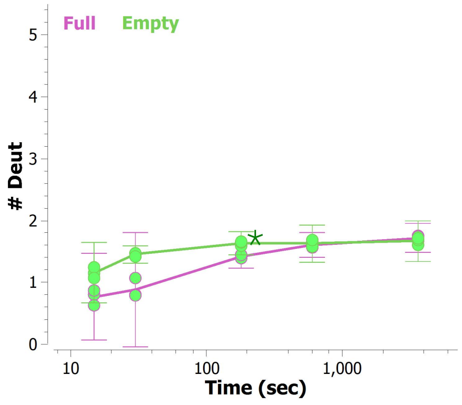

AAV8_VP1 412-419: FQFTYTFE (#181)

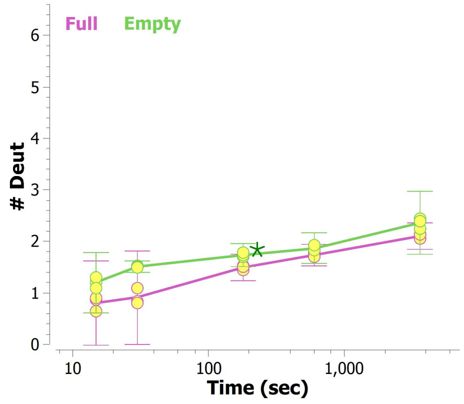

AAV8_VP1 414-418: FTYTF (#182)

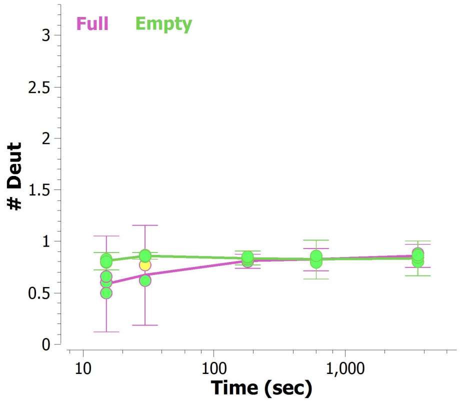

AAV8_VP1 414-433: FTYTFEDVPFHSSYAHSQSL (#183)

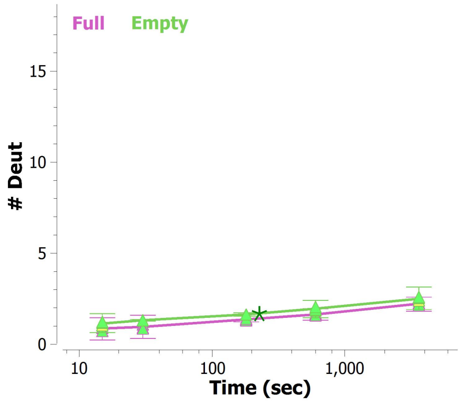

AAV8_VP1 415-433: TYTFEDVPFHSSYAHSQSL (#184)

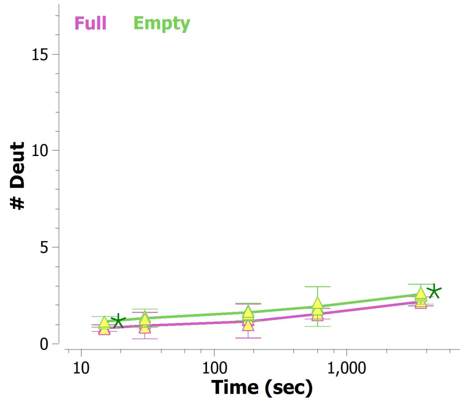

AAV8_VP1 416-433: YTFEDVPFHSSYAHSQSL (#185)

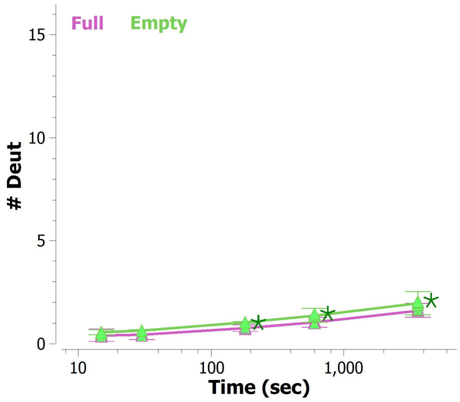

AAV8_VP1 418-426: FEDVPFHSS (#186)

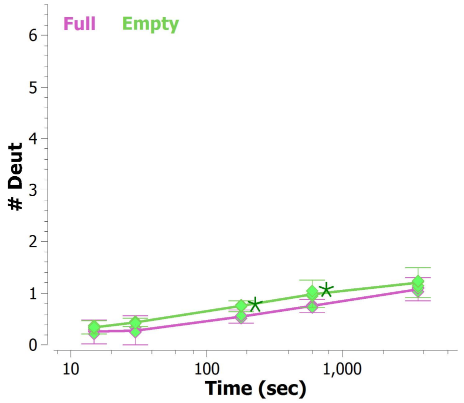

AAV8_VP1 418-428: FEDVPFHSSYA (#187)

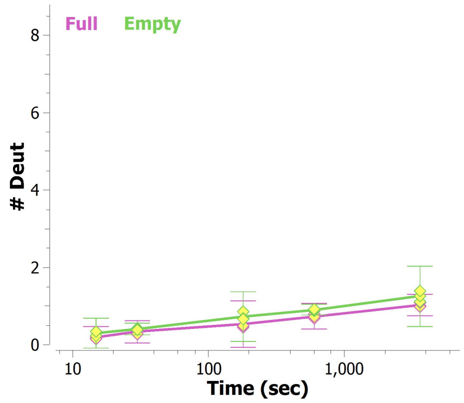

AAV8_VP1 418-430: FEDVPFHSSYAHS (#188)

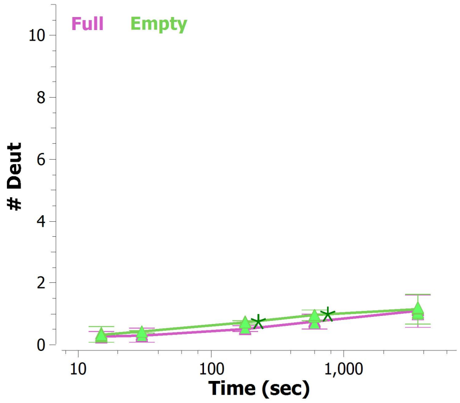

AAV8_VP1 418-433: FEDVPFHSSYAHSQSL (#189)

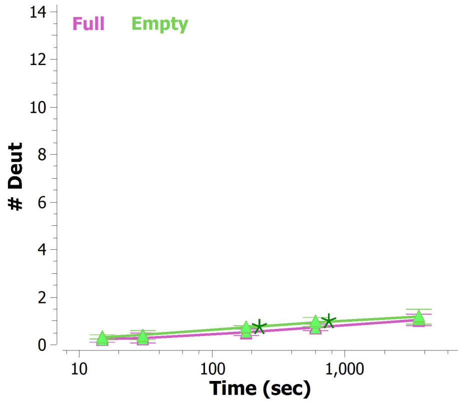

AAV8_VP1 418-434: FEDVPFHSSYAHSQSLD (#190)

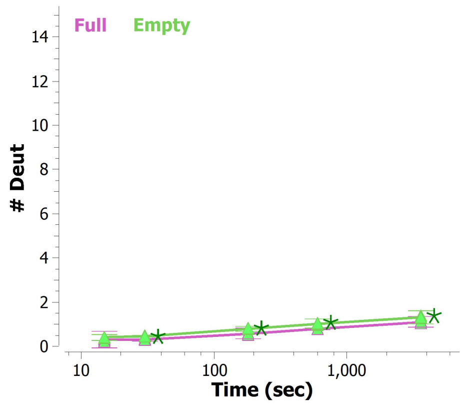

AAV8_VP1 419-430: EDVPFHSSYAHS (#191)

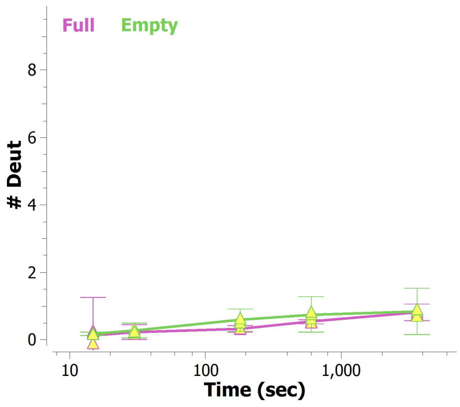

AAV8_VP1 419-443: EDVPFHSSYAHSQSLDRLMNPLIDQ (#192)

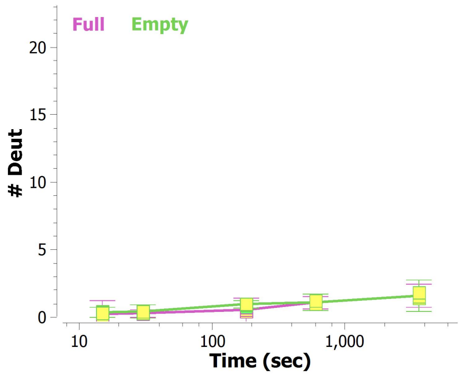

AAV8_VP1 420-433: DVPFHSSYAHSQSL (#193)

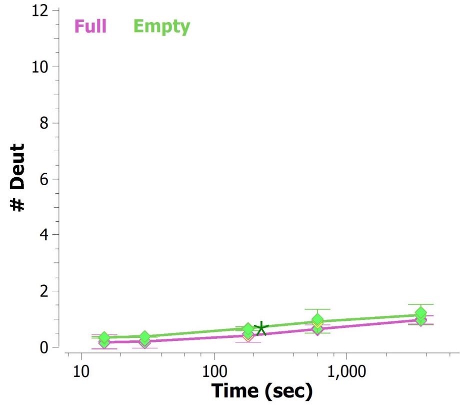

AAV8_VP1 427-433: YAHSQSL (#194)

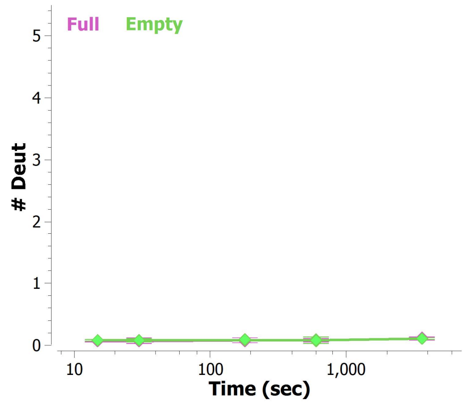

AAV8_VP1 427-443: YAHSQSLDRLMNPLIDQ (#195)

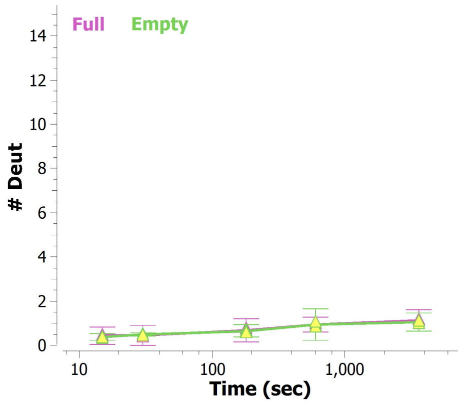

AAV8_VP1 429-433: HSQSL (#196)

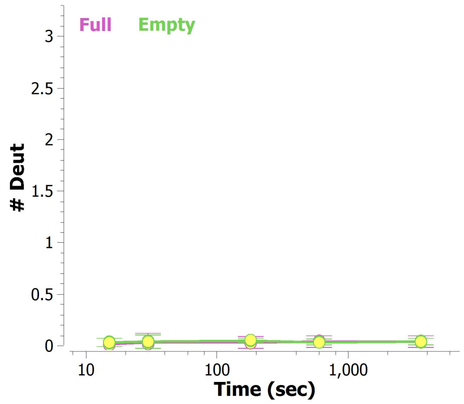

AAV8_VP1 434-442: DRLMNPLID (#197)

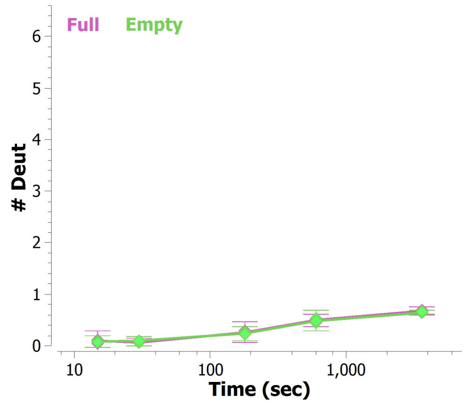

AAV8_VP1 434-443: DRLMNPLIDQ (#198)

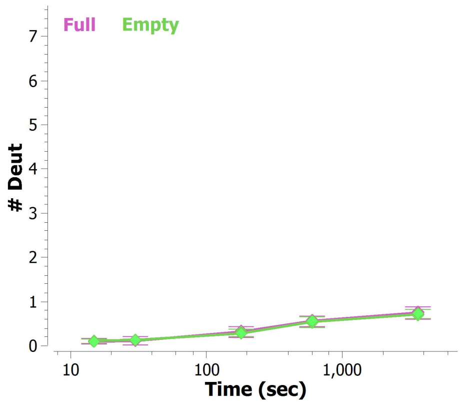

AAV8_VP1 434-444: DRLMNPLIDQY (#199)

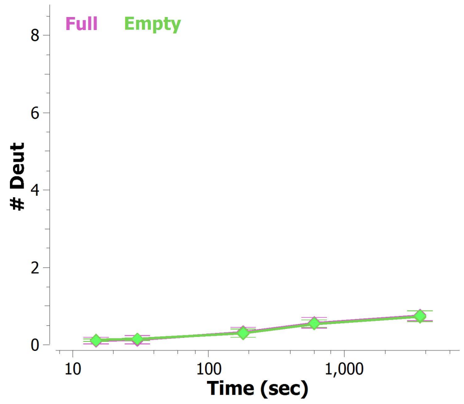

AAV8_VP1 434-445: DRLMNPLIDQYL (#200)

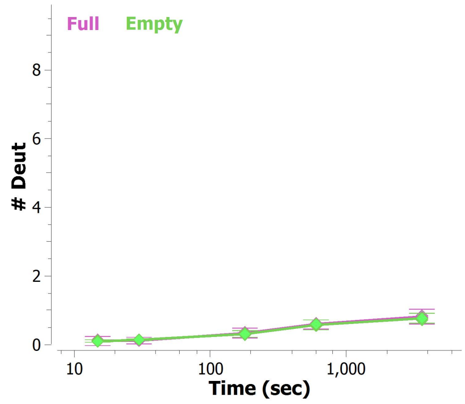

AAV8_VP1 434-446: DRLMNPLIDQYLY (#201)

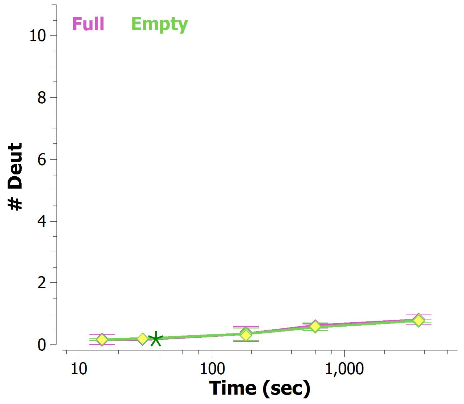

AAV8_VP1 434-447: DRLMNPLIDQYLYY (#202)

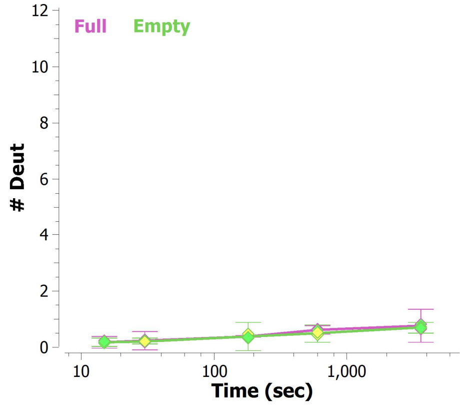

AAV8_VP1 435-445: RLMNPLIDQYL (#203)

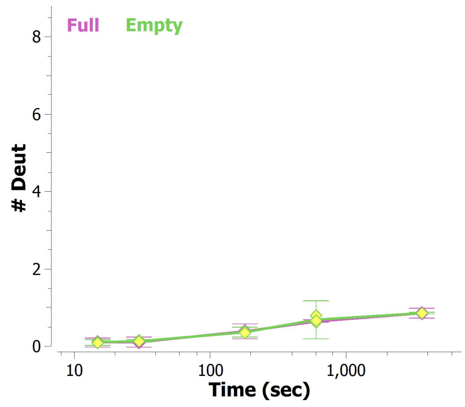

AAV8_VP1 437-445: MNPLIDQYL (#204)

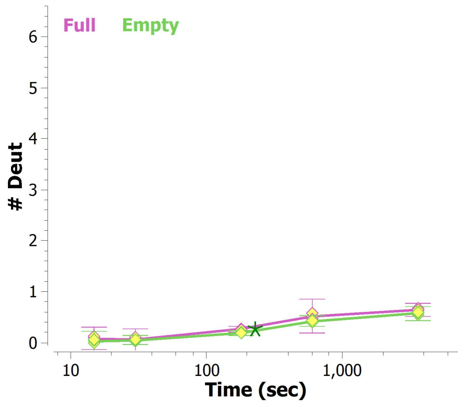

AAV8_VP1 443-446: QYLY (#205)

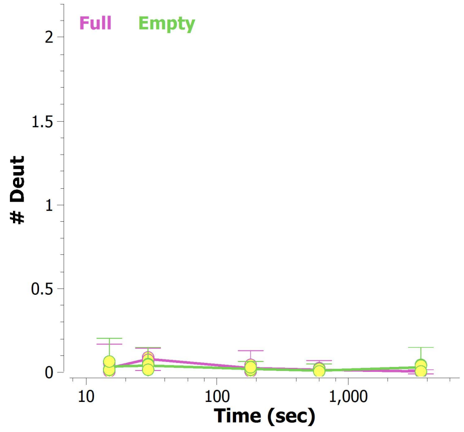

AAV8_VP1 444-447: YLYY (#206)

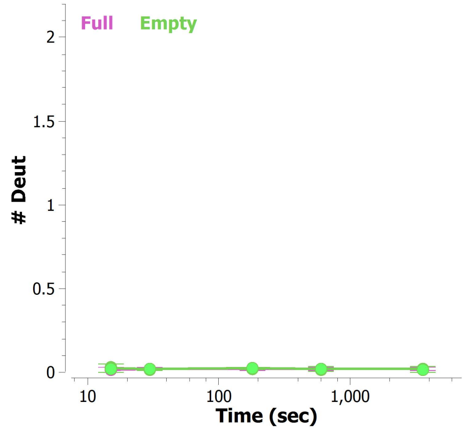

AAV8_VP1 444-460: YLYYLSRTQTTGGTANT (#207)

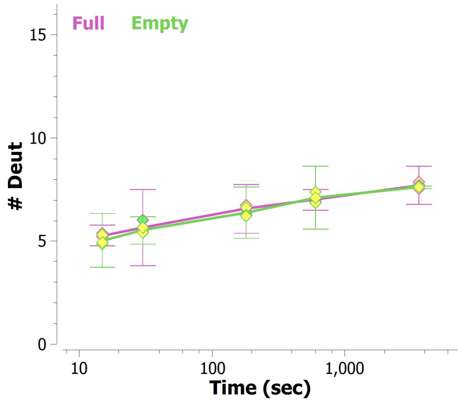

AAV8_VP1 446-453: YYLSRTQT (#208)

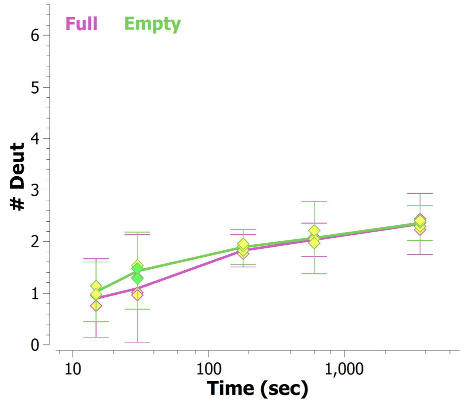

AAV8_VP1 446-460: YYLSRTQTTGGTANT (#209)

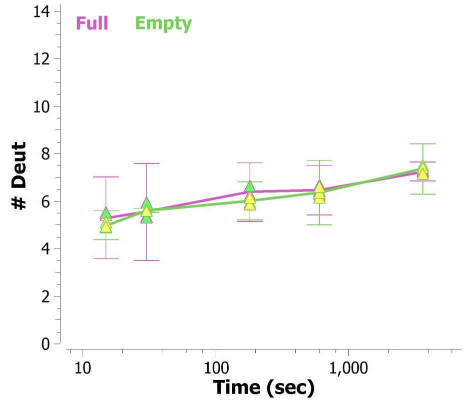

AAV8_VP1 446-462: YYLSRTQTTGGTANTQT (#210)

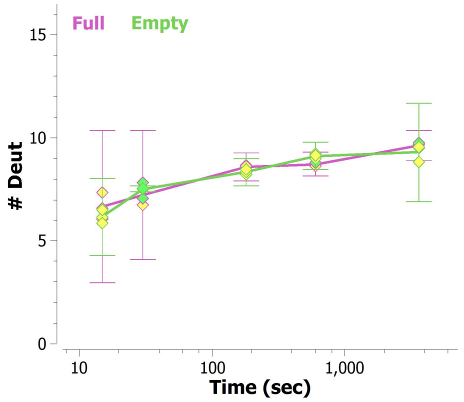

AAV8_VP1 446-464: YYLSRTQTTGGTANTQTLG (#211)

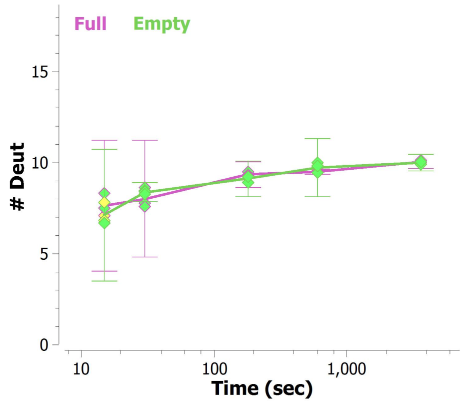

AAV8_VP1 446-465: YYLSRTQTTGGTANTQTLGF (#212)

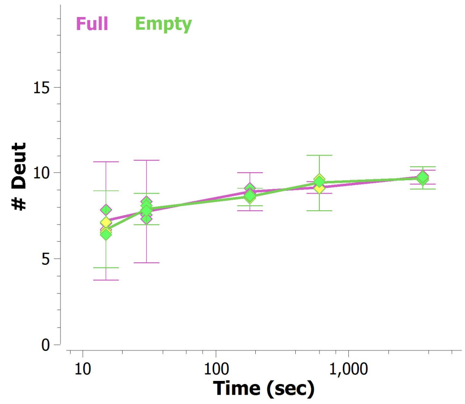

AAV8_VP1 447-460: YLSRTQTTGGTANT (#213)

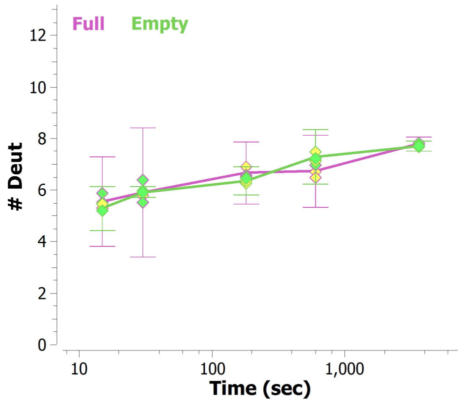

AAV8_VP1 447-464: YLSRTQTTGGTANTQTLG (#214)

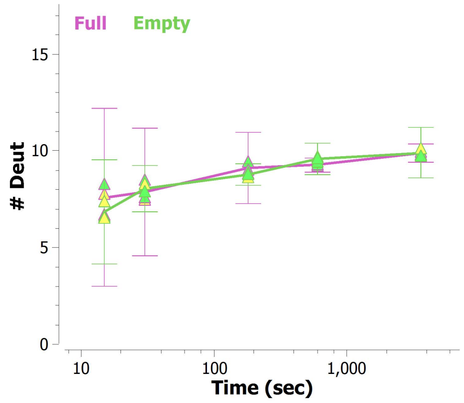

AAV8_VP1 447-465: YLSRTQTTGGTANTQTLGF (#215)

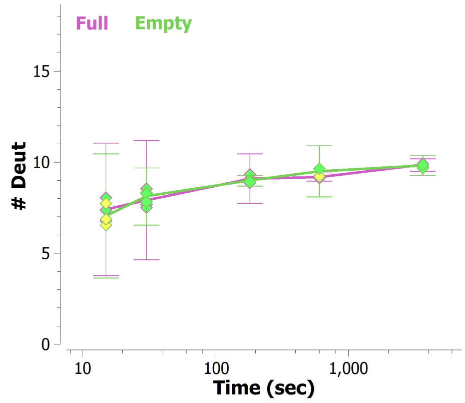

AAV8_VP1 448-460: LSRTQTTGGTANT (#216)

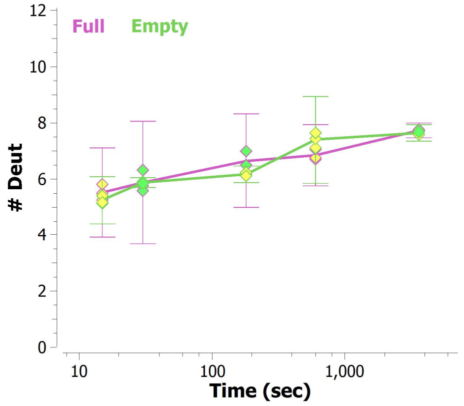

AAV8_VP1 448-464: LSRTQTTGGTANTQTLG (#217)

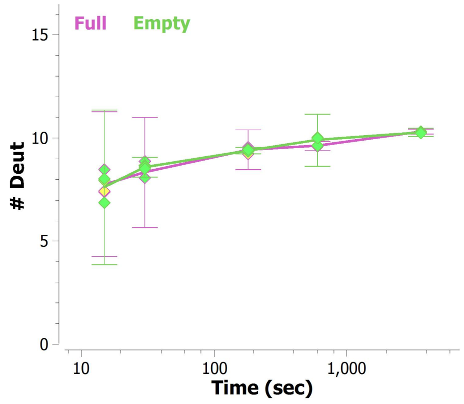

AAV8_VP1 448-465: LSRTQTTGGTANTQTLGF (#218)

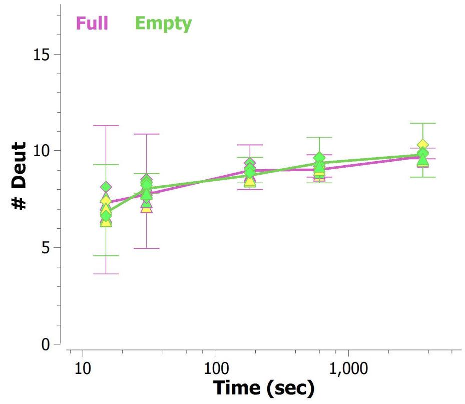

AAV8_VP1 448-472: LSRTQTTGGTANTQTLGFSQGGPNT (#219)

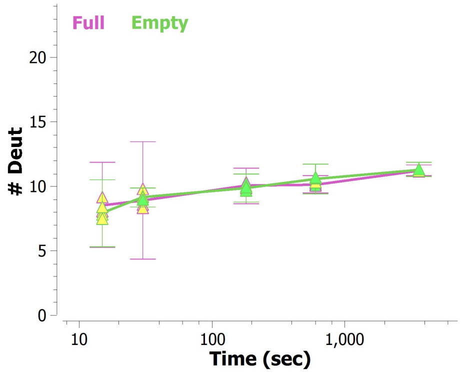

AAV8_VP1 449-464: SRTQTTGGTANTQTLG (#220)

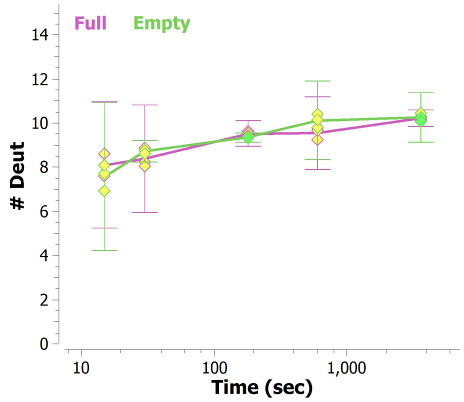

AAV8_VP1 449-465: SRTQTTGGTANTQTLGF (#221)

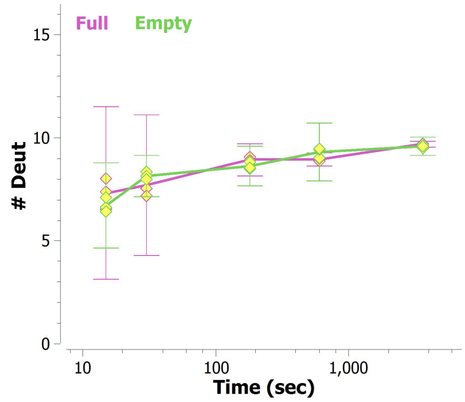

AAV8_VP1 461-465: QTLGF (#222)

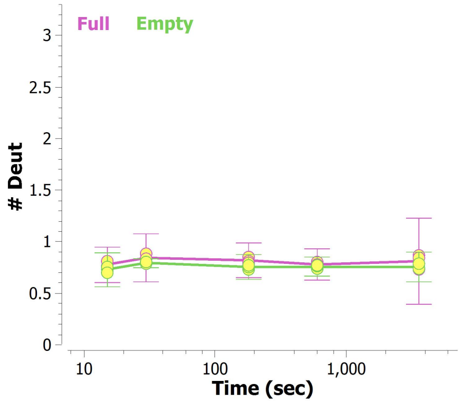

AAV8_VP1 461-486: QTLGFSQGGPNTMANQAKNWLPGPCY (#223)

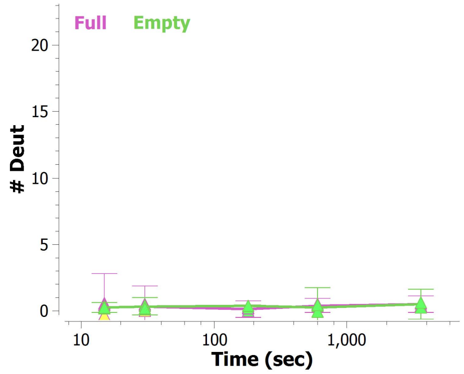

AAV8_VP1 466-472: SQGGPNT (#224)

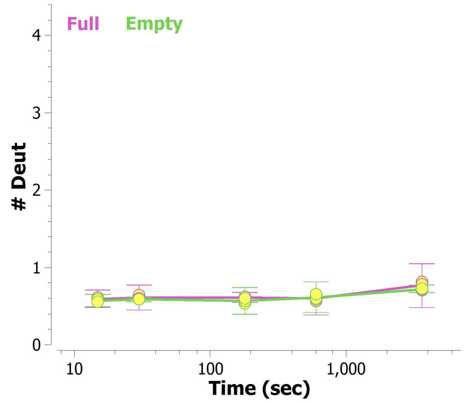

AAV8_VP1 466-486: SQGGPNTMANQAKNWLPGPCY (#225)

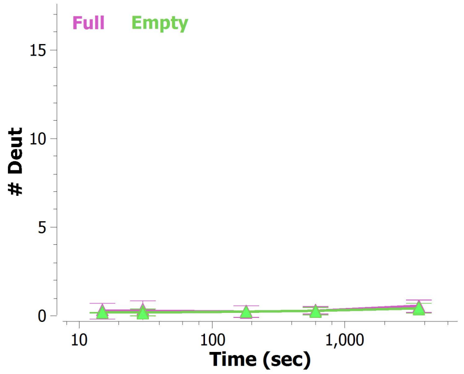

AAV8_VP1 473-486: MANQAKNWLPGPCY (#226)

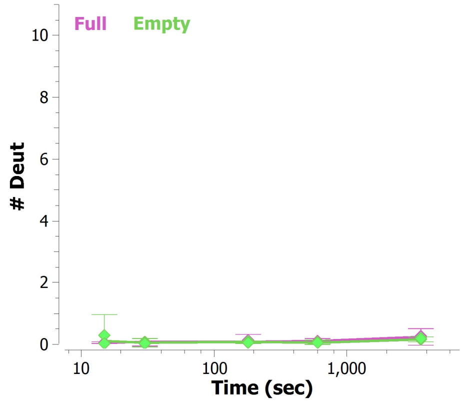

AAV8_VP1 477-486: AKNWLPGPCY (#227)

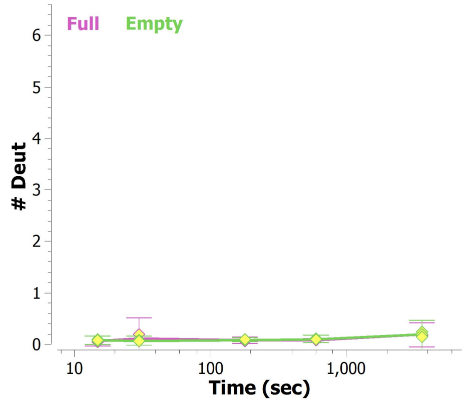

AAV8_VP1 486-503: YRQQRVSTTTGQNNNSNF (#228)

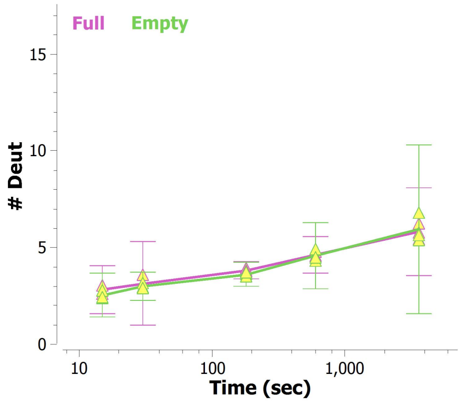

AAV8_VP1 487-504: RQQRVSTTTGQNNNSNFA (#229)

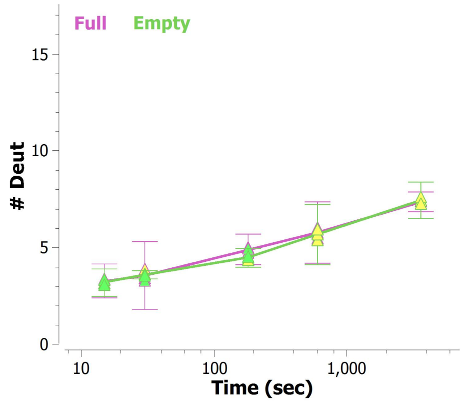

AAV8_VP1 494-503: TTGQNNNSNF (#230)

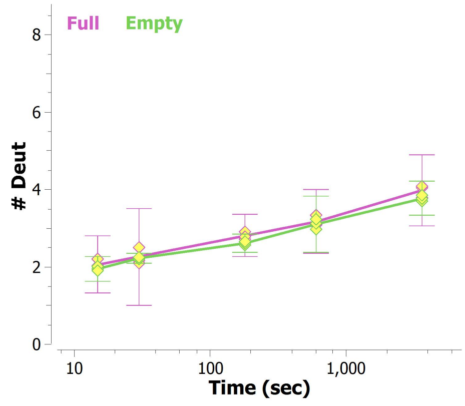

AAV8_VP1 503-525: FAWTAGTKYHLNGRNSLANPGIA (#231)

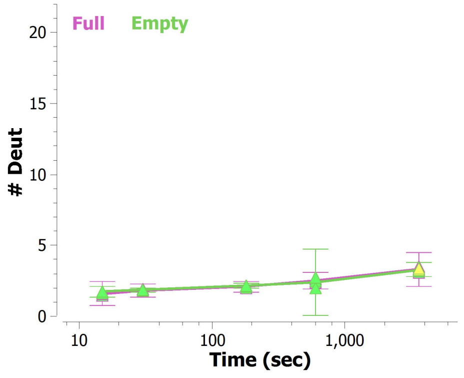

AAV8_VP1 504-511: AWTAGTKY (#232)

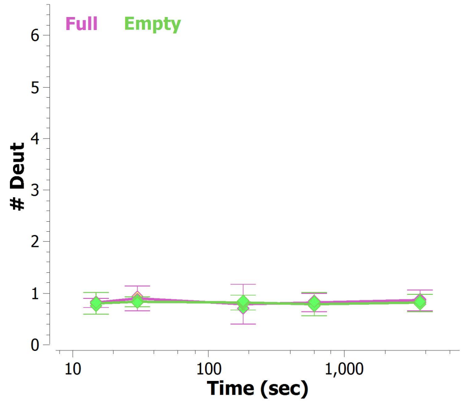

AAV8_VP1 504-513: AWTAGTKYHL (#233)

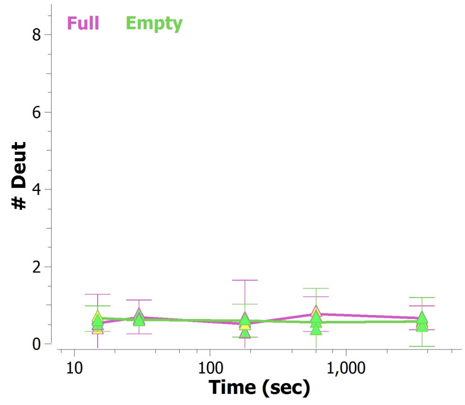

AAV8_VP1 504-517: AWTAGTKYHLNGRN (#234)

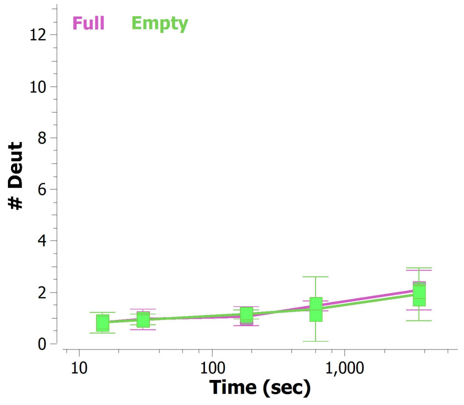

AAV8_VP1 504-525: AWTAGTKYHLNGRNSLANPGIA (#235)

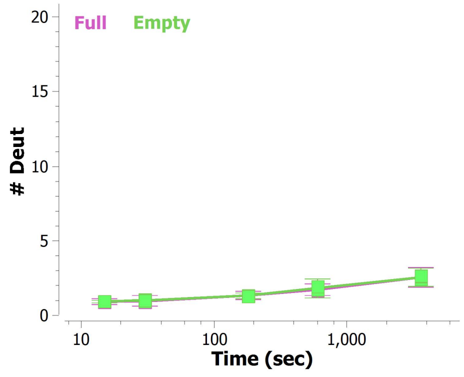

AAV8_VP1 504-526: AWTAGTKYHLNGRNSLANPGIAM (#236)

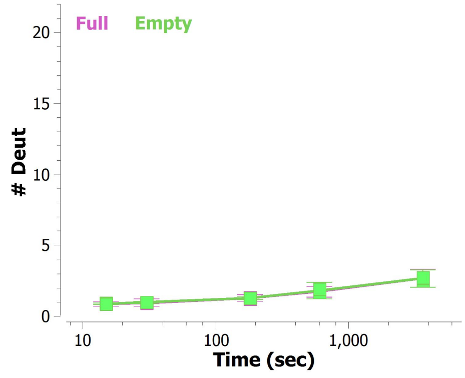

AAV8_VP1 504-533: TAGTKYHLNGRNSLANPGIAMATHKD (#237)

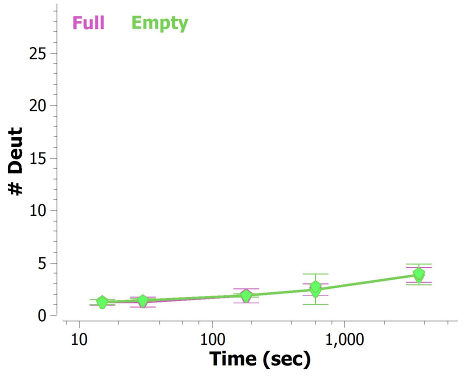

AAV8_VP1 504-534: TAGTKYHLNGRNSLANPGIAMATHKD (#238)

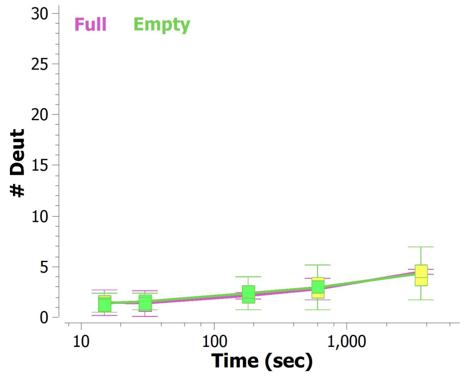

AAV8_VP1 505-517: WTAGTKYHLNGRN (#239)

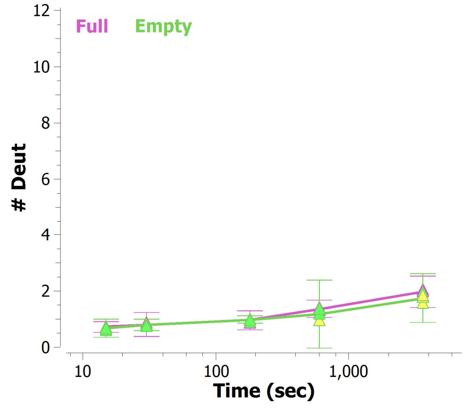

AAV8_VP1 505-525: WTAGTKYHLNGRNSLANPGIA (#240)

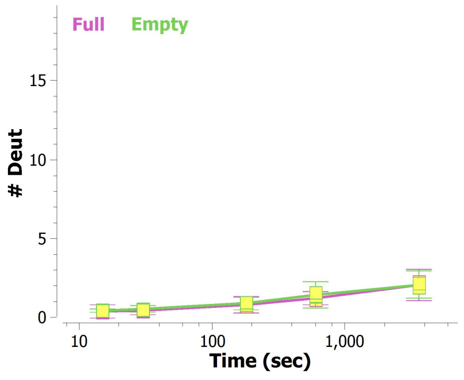

AAV8_VP1 506-517: TAGTKYHLNGRN (#241)

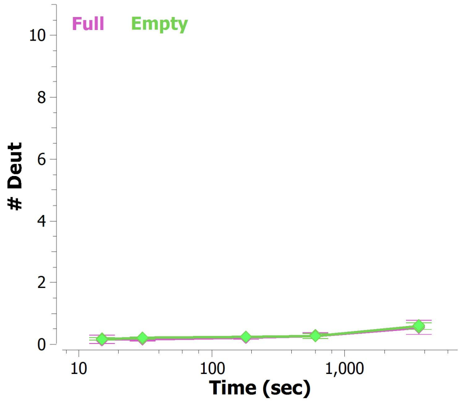

AAV8_VP1 507-517: AGTKYHLNGRN (#242)

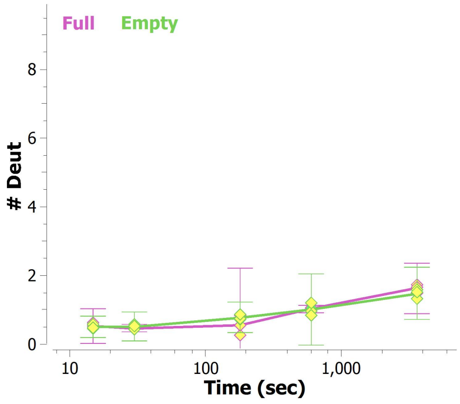

AAV8_VP1 512-525: HLNGRNSLANPGIA (#243)

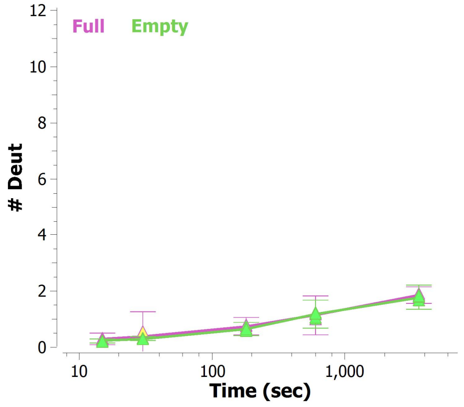

AAV8_VP1 512-526: HLNGRNSLANPGIAM (#244)

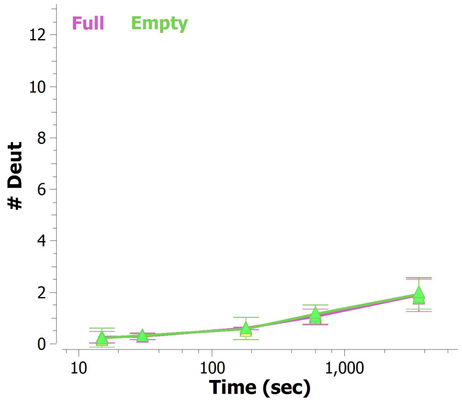

AAV8_VP1 518-525: SLANPGIA (#245)

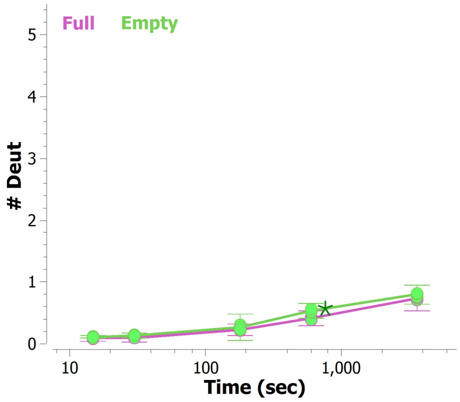

AAV8_VP1 518-526: SLANPGIAM (#246)

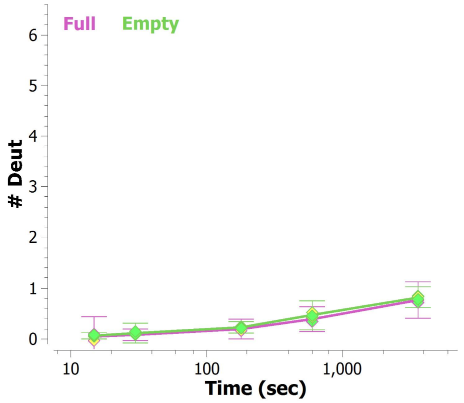

AAV8_VP1 518-533: SLANPGIAMATHKDDE (#247)

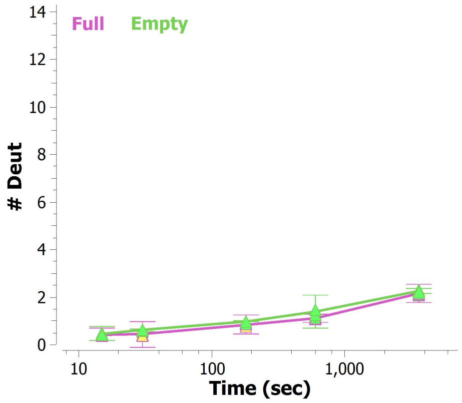

AAV8_VP1 526-533: MATHKDDE (#248)

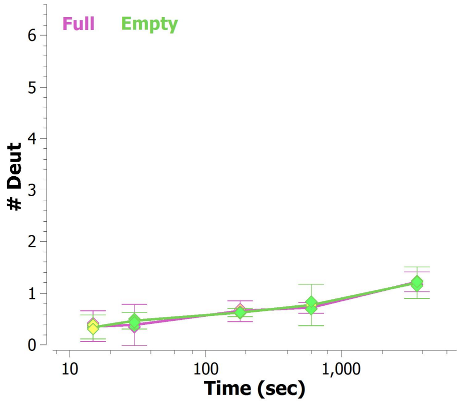

AAV8_VP1 526-534: MATHKDDEE (#249)

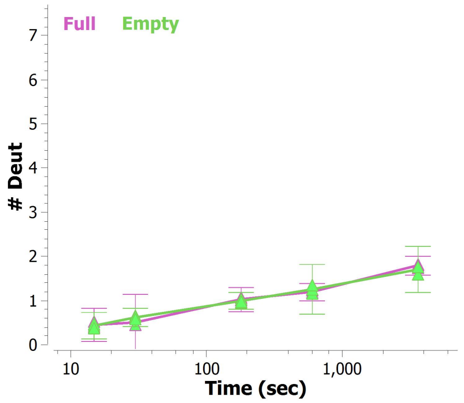

AAV8_VP1 526-536: MATHKDDEERF (#250)

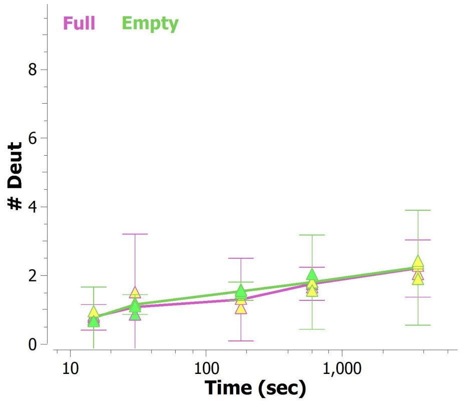

AAV8_VP1 526-540: MATHKDDEERFFPSN (#251)

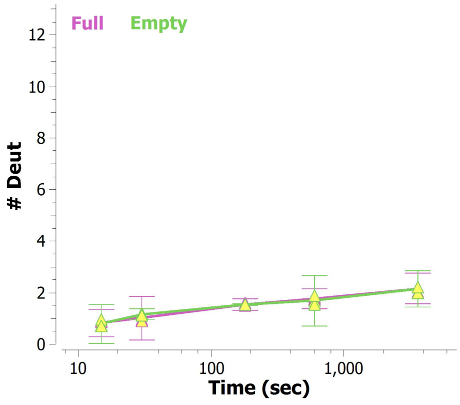

AAV8_VP1 526-541: MATHKDDEERFFPSNG (#252)

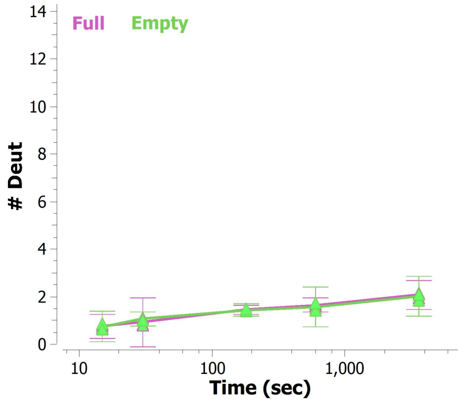

AAV8_VP1 526-543: MATHKDDEERFFPSNGIL (#253)

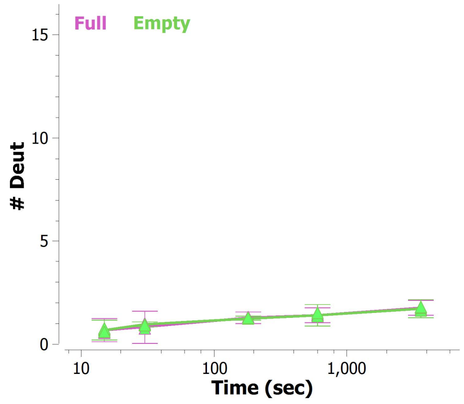

AAV8_VP1 526-545: MATHKDDEERFFPSNGILIF (#254)

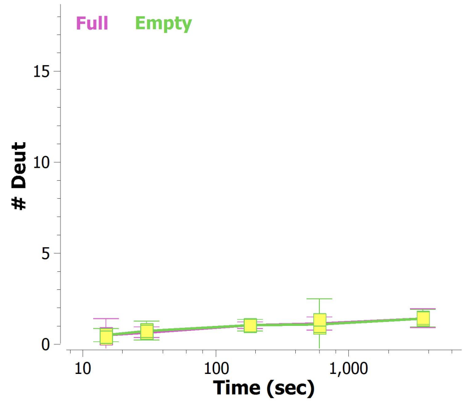

AAV8_VP1 527-540: ATHKDDEERFFPSN (#255)

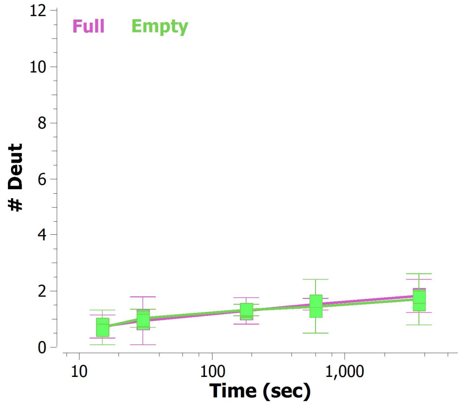

AAV8_VP1 527-541: ATHKDDEERFFPSNG (#256)

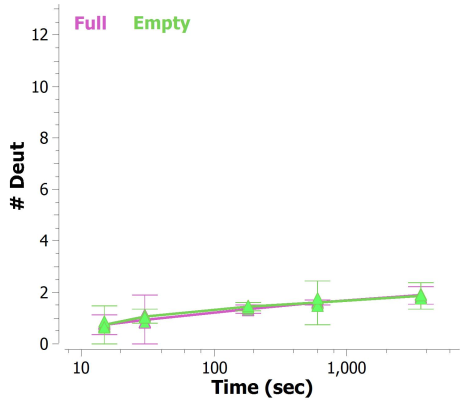

AAV8_VP1 527-543: ATHKDDEERFFPSNGIL (#257)

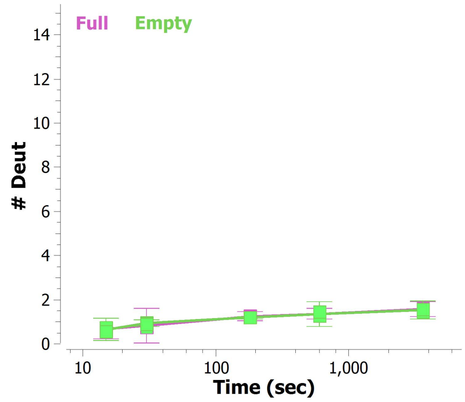

AAV8_VP1 529-543: HKDDEERFFPSNGIL (#258)

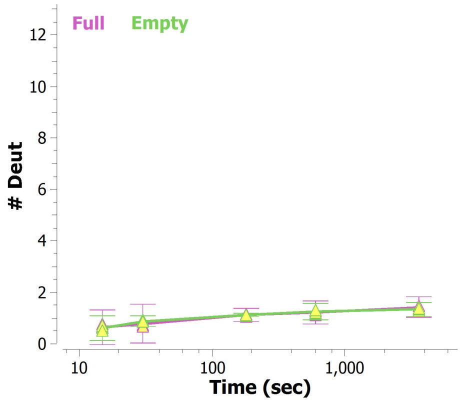

AAV8_VP1 534-540: ERFFPSN (#259)

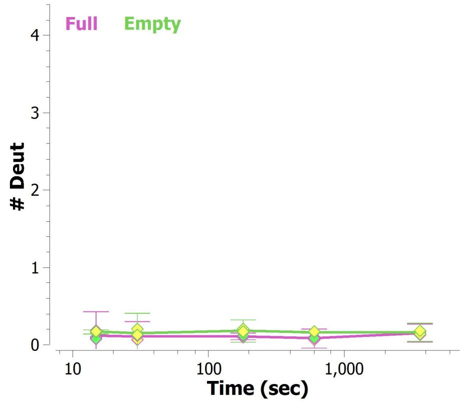

AAV8_VP1 534-541: ERFFPSNG (#260)

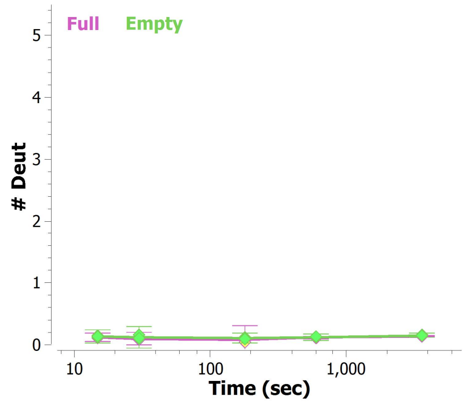

AAV8_VP1 534-543: ERFFPSNGIL (#261)

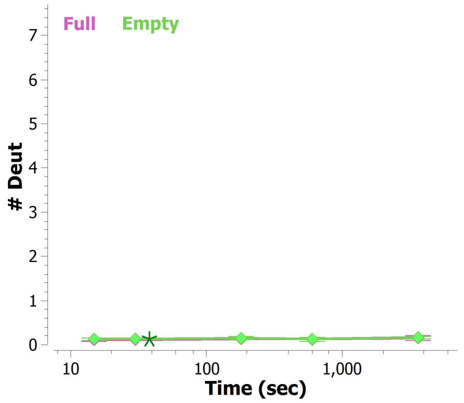

AAV8_VP1 534-544: ERFFPSNGILI (#262)

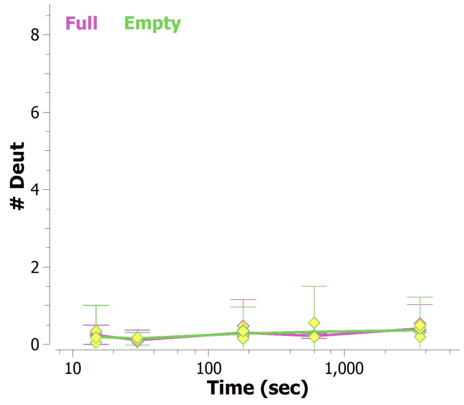

AAV8_VP1 534-556: ERFFPSNGILIFGKQNAARDNAD (#263)

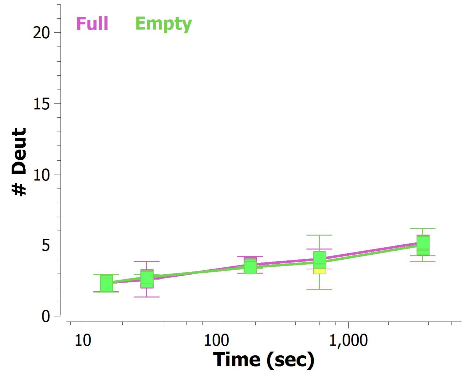

AAV8_VP1 535-541: RFFPSNG (#264)

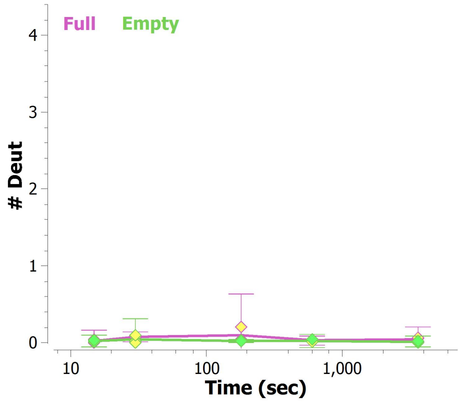

AAV8_VP1 535-543: RFFPSNGIL (#265)

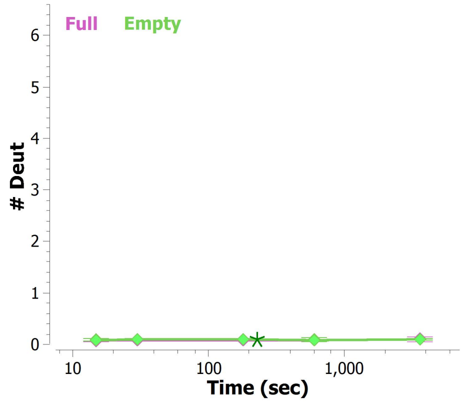

AAV8_VP1 535-556: RFFPSNGILIFGKQNAARDNAD (#266)

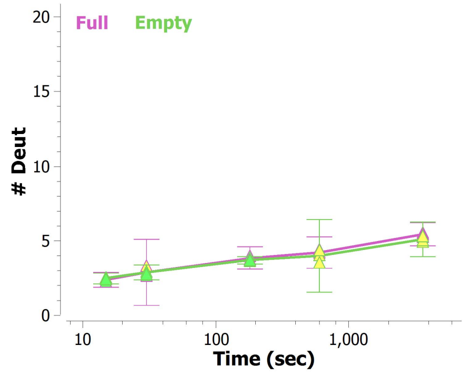

AAV8_VP1 537-543: FPSNGIL (#267)

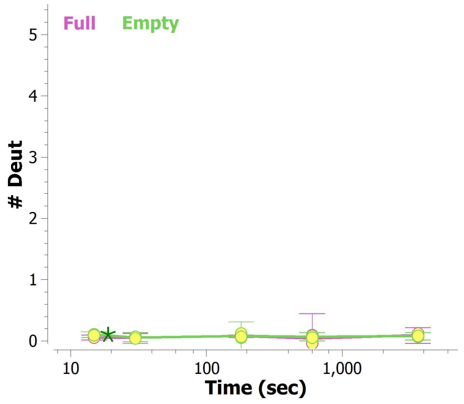

AAV8_VP1 541-556: GILIFGKQNAARDNAD (#268)

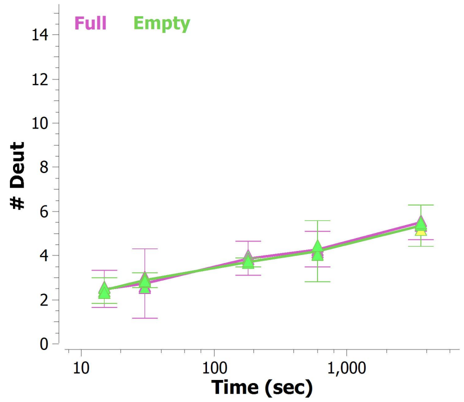

AAV8_VP1 542-556: ILIFGKQNAARDNAD (#269)

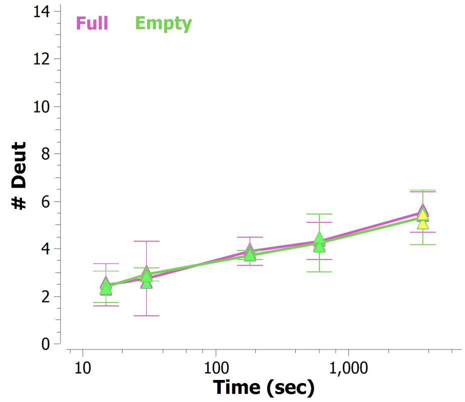

AAV8_VP1 542-559: ILIFGKQNAARDNADYSD (#270)

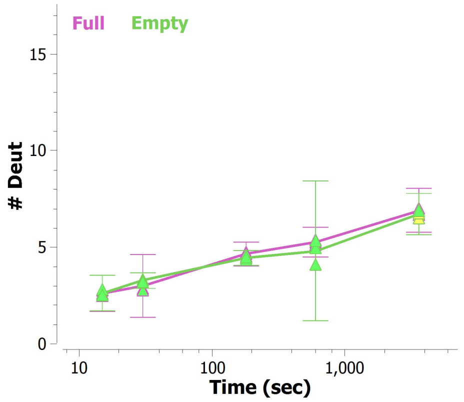

AAV8_VP1 544-550: IFGKQNA (#271)

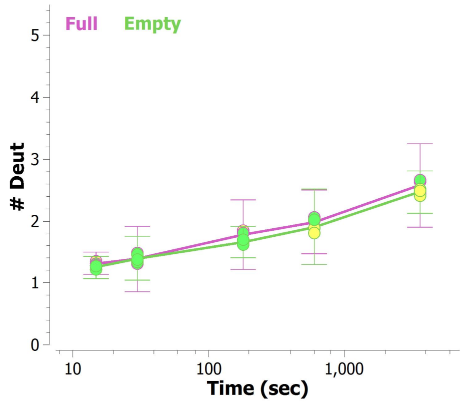

AAV8_VP1 544-553: IFGKQNAARD (#272)

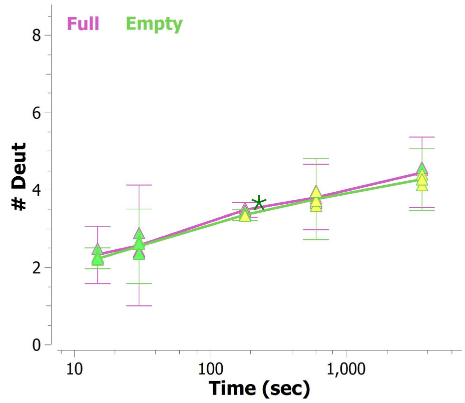

AAV8_VP1 544-556: IFGKQNAARDNAD (#273)

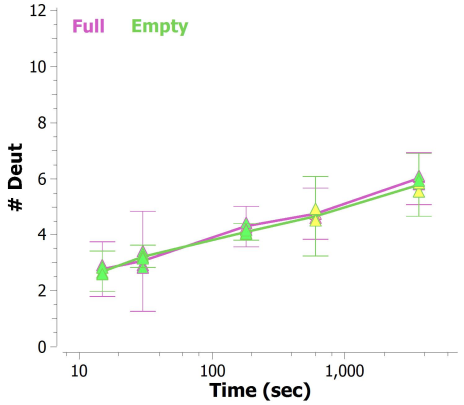

AAV8_VP1 544-557: IFGKQNAARDNADY (#274)

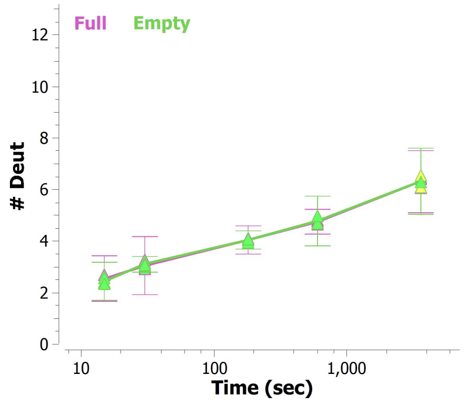

AAV8_VP1 544-558: IFGKQNAARDNADYS (#275)

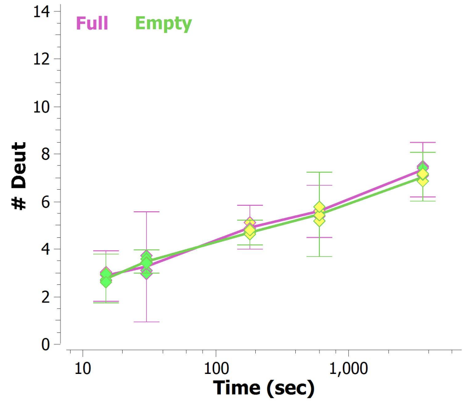

AAV8_VP1 544-559: IFGKQNAARDNADYSD (#276)

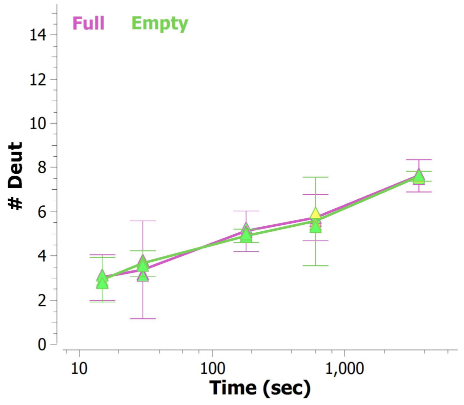

AAV8_VP1 544-561: IFGKQNAARDNADYSDVM (#277)

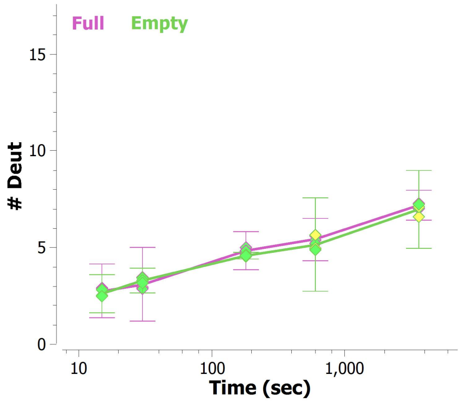

AAV8_VP1 544-562: IFGKQNAARDNADYSDVML (#278)

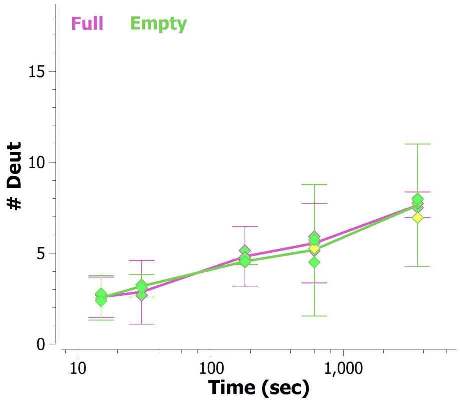

AAV8_VP1 546-556: GKQNAARDNAD (#279)

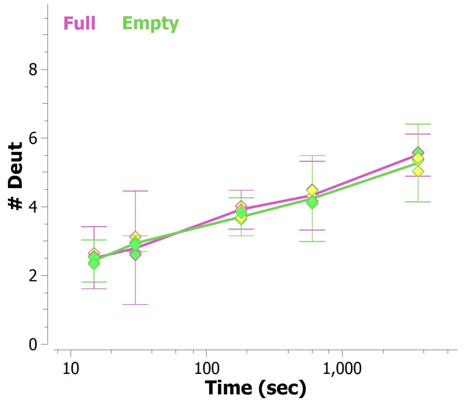

AAV8_VP1 546-559: GKQNAARDNADYSD (#280)

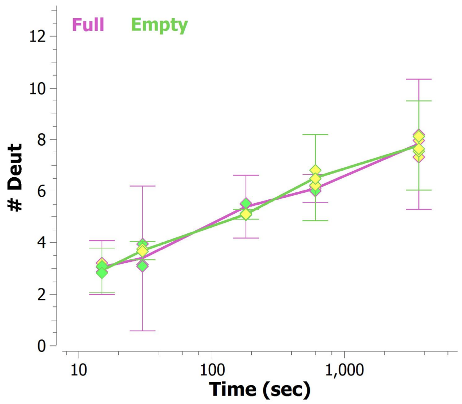

AAV8_VP1 554-559: NADYSD (#281)

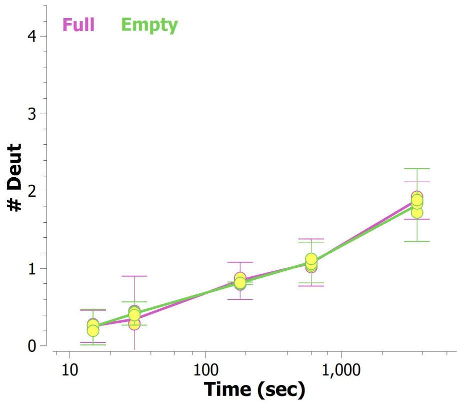

AAV8_VP1 557-560: YSDV (#282)

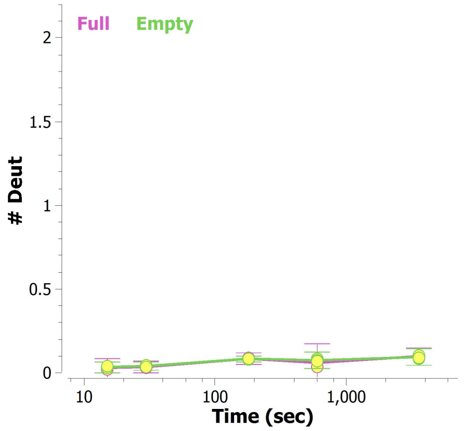

AAV8_VP1 557-561: YSDVM (#283)

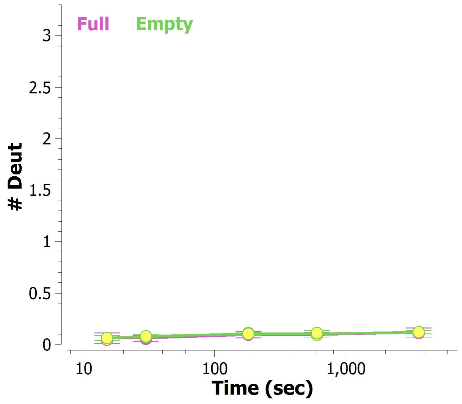

AAV8_VP1 557-565: YSDVMLTSE (#284)

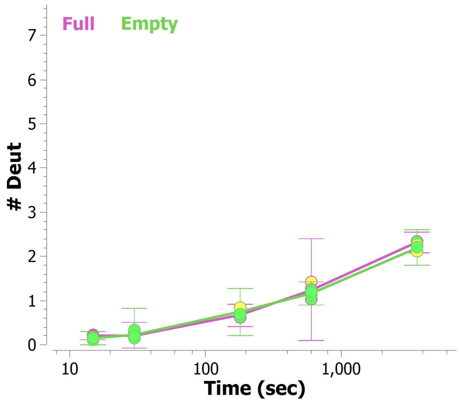

AAV8_VP1 558-562: SDVML (#285)

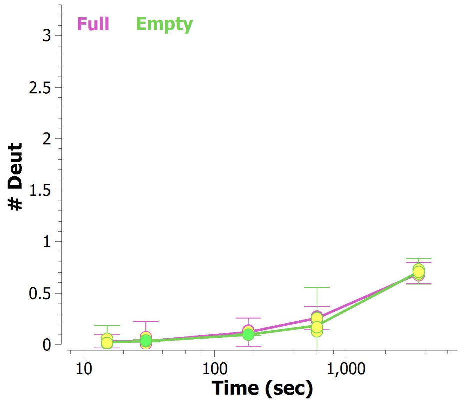

AAV8_VP1 559-565: DVMLTSE (#286)

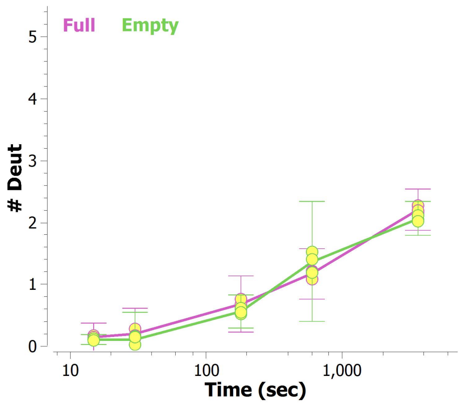

AAV8_VP1 560-565: VMLTSE (#287)

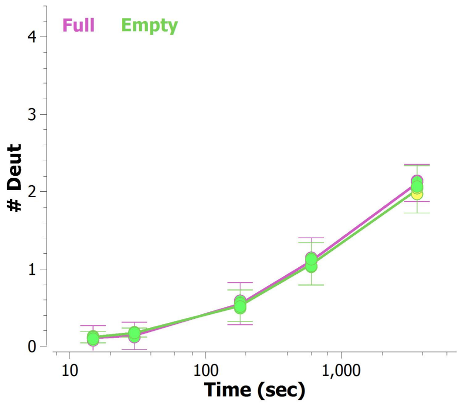

AAV8_VP1 560-566: VMLTSEE (#288)

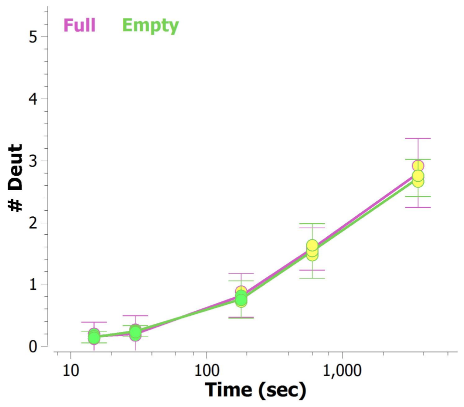

AAV8_VP1 560-567: VMLTSEEE (#289)

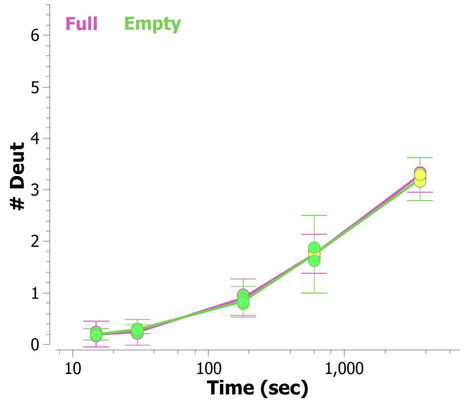

AAV8_VP1 560-576: VMLTSEEEIKTTNPVAT (#290)

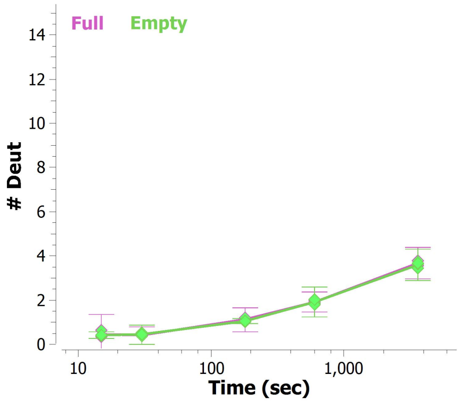

AAV8_VP1 560-578: VMLTSEEEIKTTNPVATEE (#291)

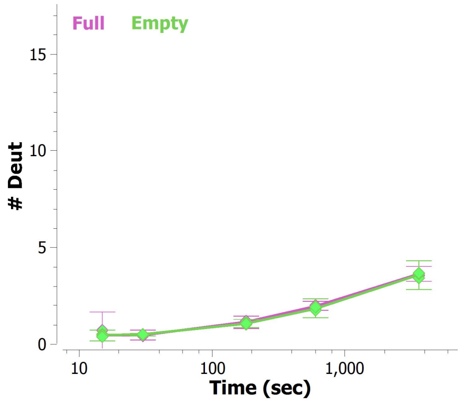

AAV8_VP1 561-565: MLTSE (#292)

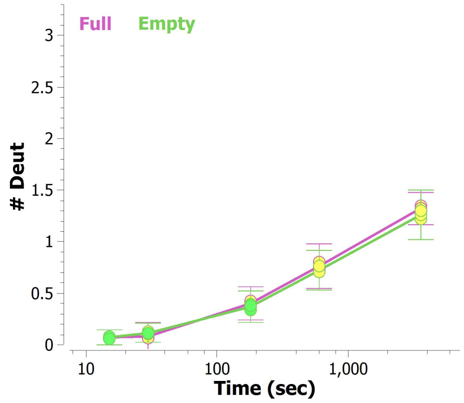

AAV8_VP1 561-567: MLTSEEE (#293)

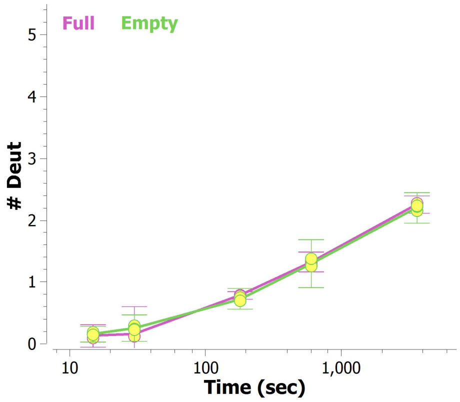

AAV8_VP1 561-578: MLTSEEEIKTTNPVATEE (#294)

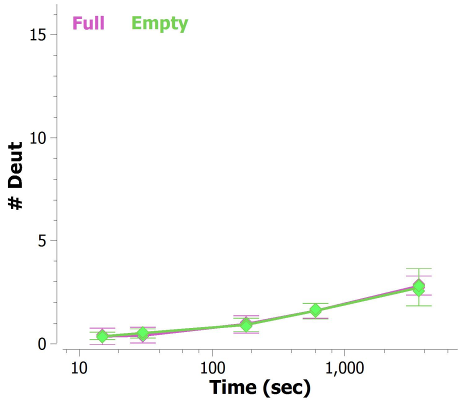

AAV8_VP1 562-566: LTSEE (#295)

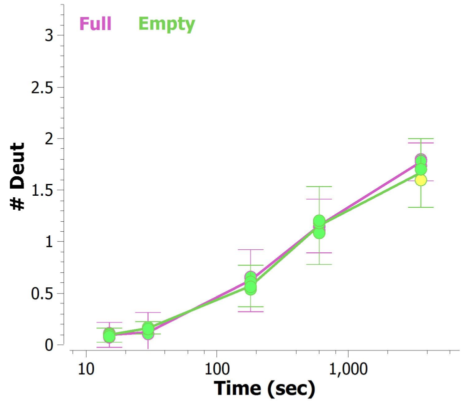

AAV8_VP1 562-567: LTSEEE (#296)

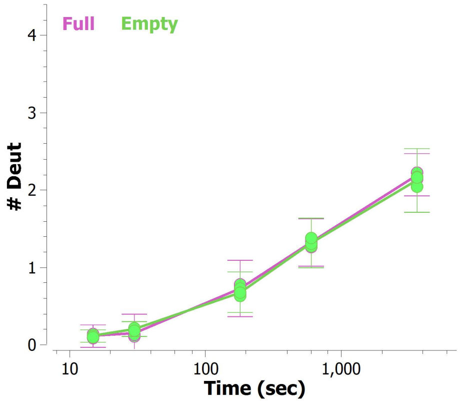

AAV8_VP1 562-575: LTSEEEIKTTNPVA (#297)

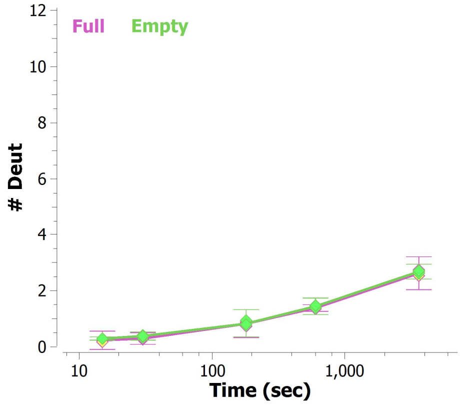

AAV8_VP1 562-576: LTSEEEIKTTNPVAT (#298)

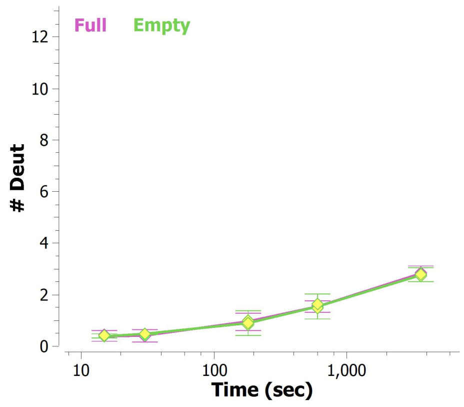

AAV8_VP1 562-578: LTSEEEIKTTNPVATEE (#299)

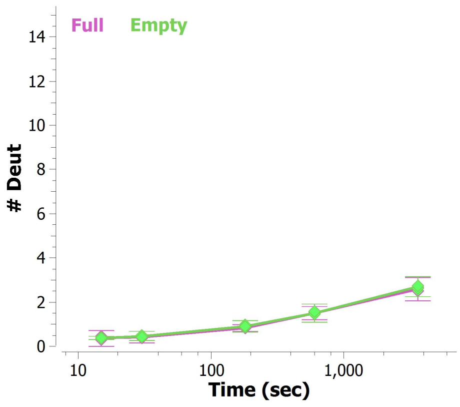

AAV8_VP1 563-575: TSEEEIKTTNPVA (#300)

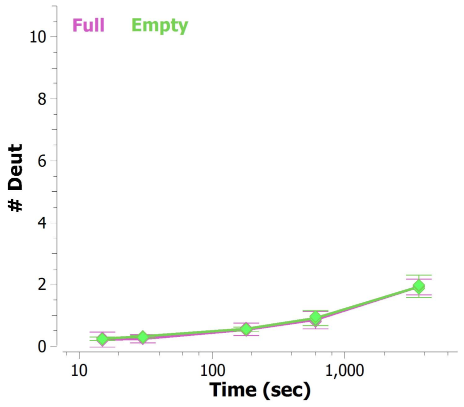

AAV8_VP1 563-576: TSEEEIKTTNPVAT (#301)

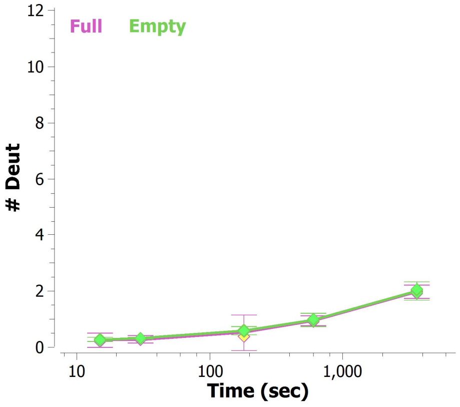

AAV8_VP1 563-578: TSEEEIKTTNPVATEE (#302)

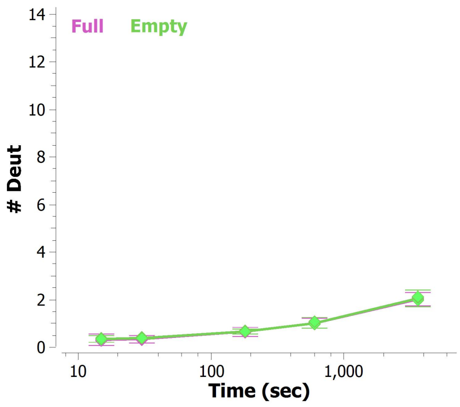

AAV8_VP1 563-586: TSEEEIKTTNPVATEEYGIVADNL (#303)

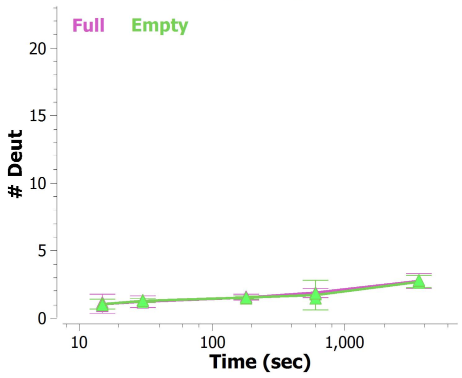

AAV8_VP1 566-576: EEIKTTNPVAT (#304)

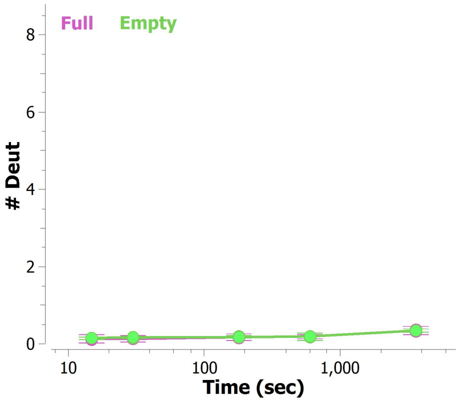

AAV8_VP1 566-578: EEIKTTNPVATEE (#305)

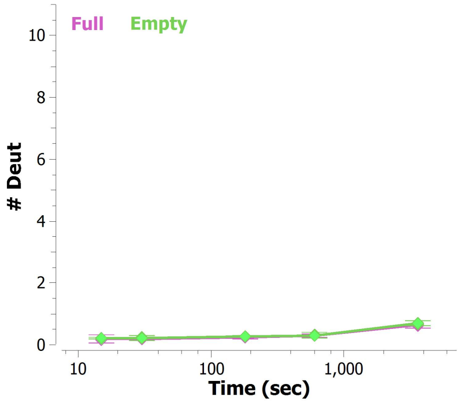

AAV8_VP1 566-579: EEIKTTNPVATEEY (#306)

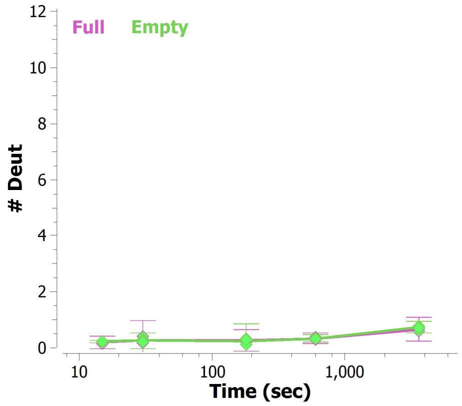

AAV8_VP1 566-580: EEIKTTNPVATEEYG (#307)

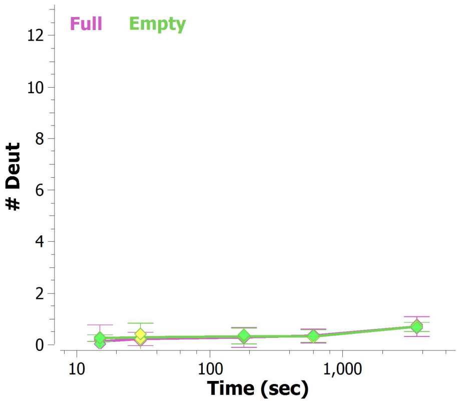

AAV8_VP1 566-586: EEIKTTNPVATEEYGIVADNL (#308)

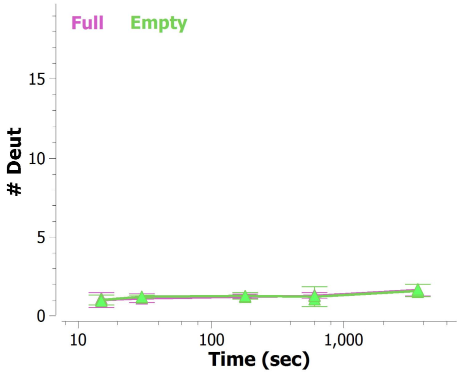

AAV8_VP1 567-576: EIKTTNPVAT (#309)

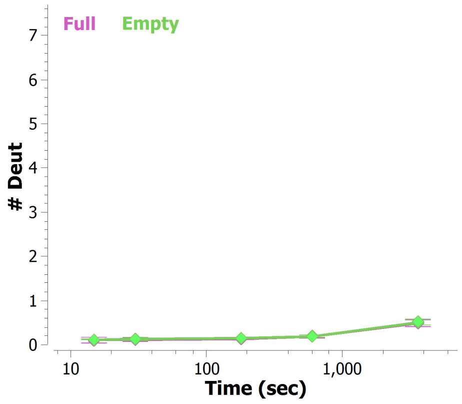

AAV8_VP1 567-578: EIKTTNPVATEE (#310)

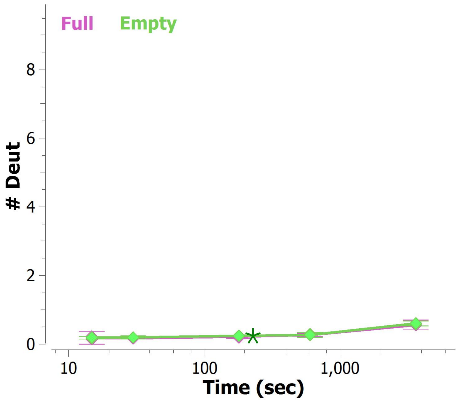

AAV8_VP1 567-586: EIKTTNPVATEEYGIVADNL (#311)

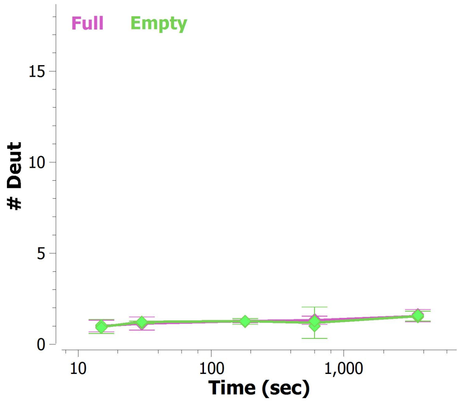

AAV8_VP1 568-576: IKTTNPVAT (#312)

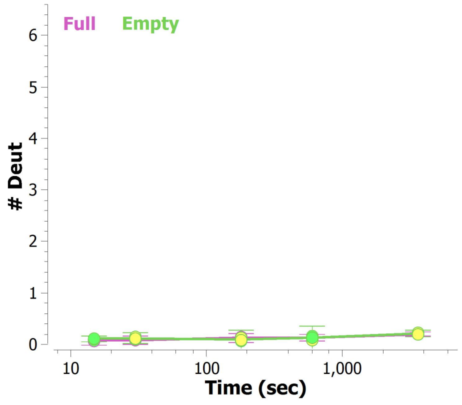

AAV8_VP1 568-578: IKTTNPVATEE (#313)

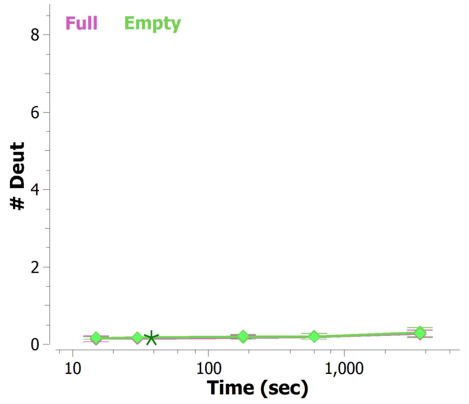

AAV8_VP1 568-586: IKTTNPVATEEYGIVADNL (#314)

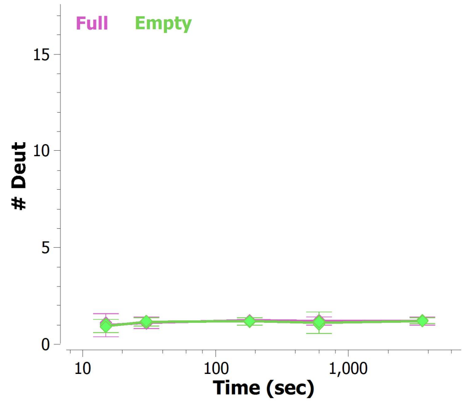

AAV8_VP1 577-584: EEYGIVAD (#315)

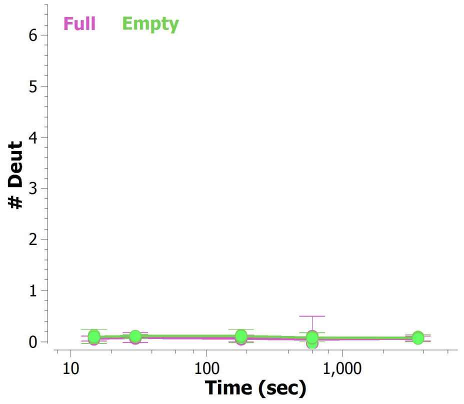

AAV8_VP1 577-586: EEYGIVADNL (#316)

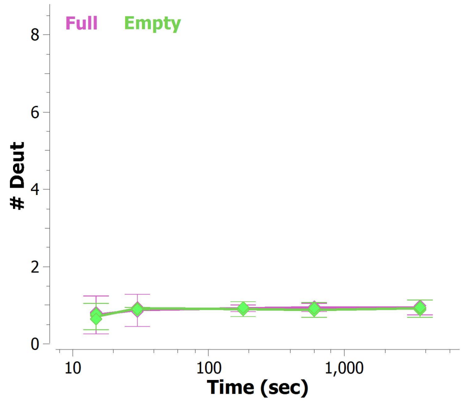

AAV8_VP1 579-584: YGIVAD (#317)

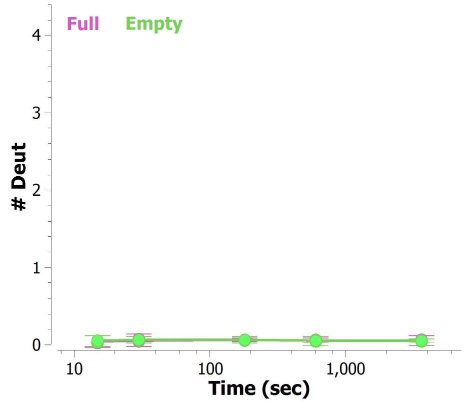

AAV8_VP1 579-586: YGIVADNL (#318)

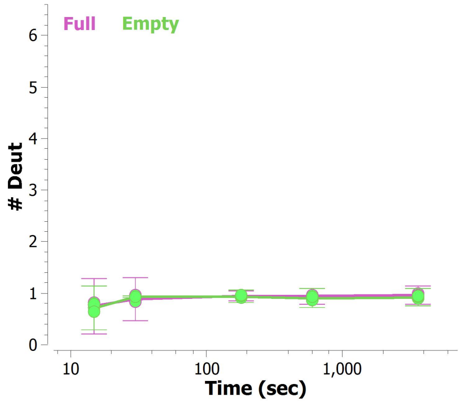

AAV8_VP1 579-587: YGIVADNLQ (#319)

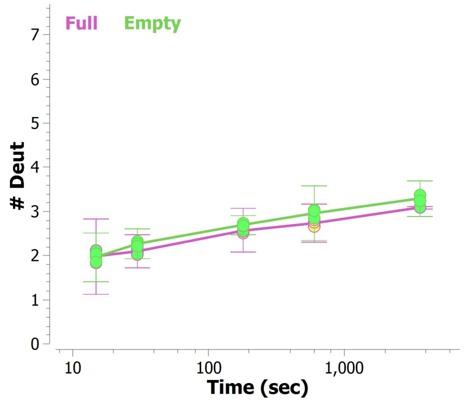

AAV8_VP1 580-586: GIVADNL (#320)

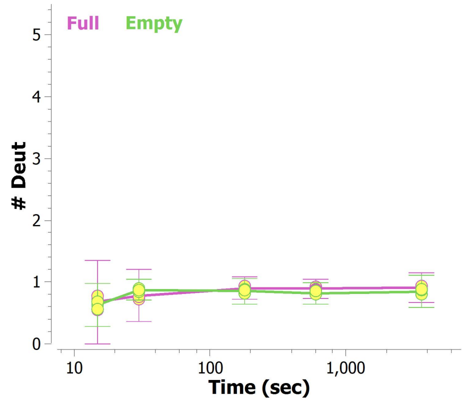

AAV8_VP1 608-613: VWQNRD (#321)

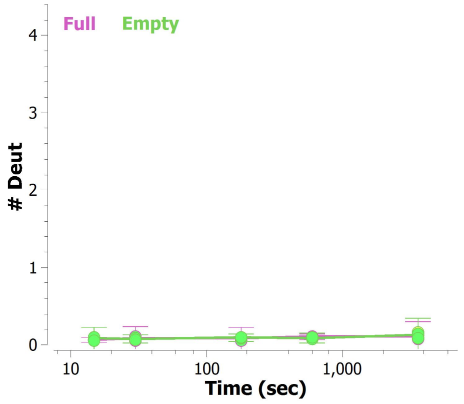

AAV8_VP1 608-616: VWQNRDVYL (#322)

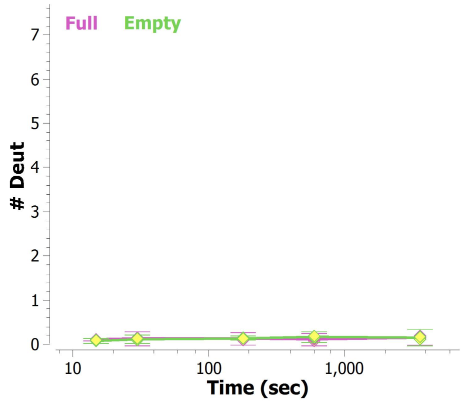

AAV8_VP1 610-621: QNRDVYLQGPIW (#323)

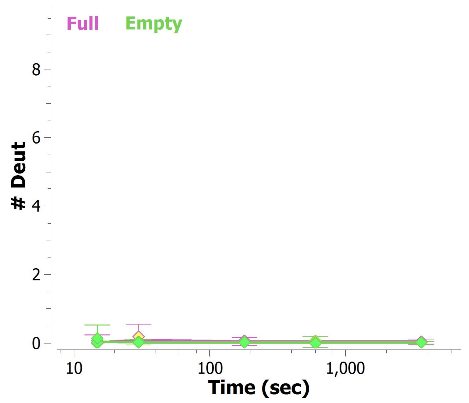

AAV8_VP1 611-621: NRDVYLQGPIW (#324)

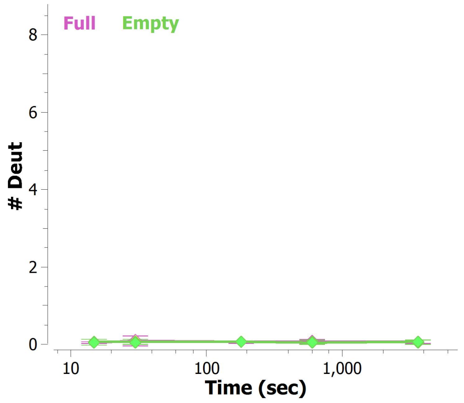

AAV8_VP1 614-621: VYLQGPIW (#325)

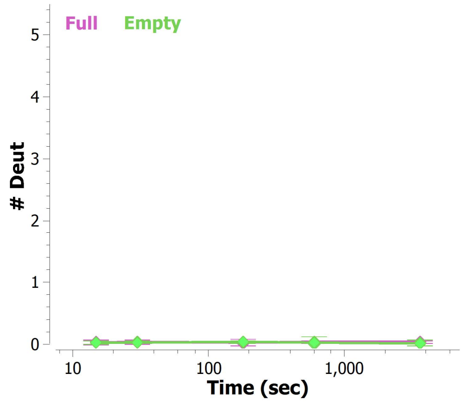

AAV8_VP1 616-621: LQGPIW (#326)

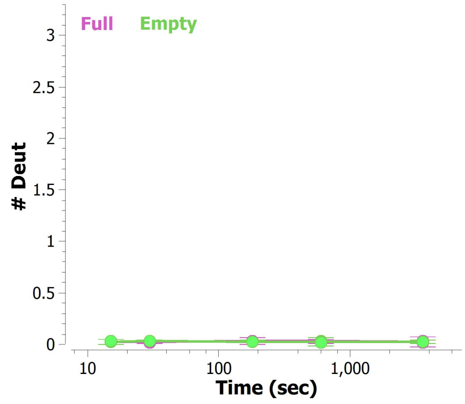

AAV8_VP1 622-631: AKIPHTDGNF (#327)

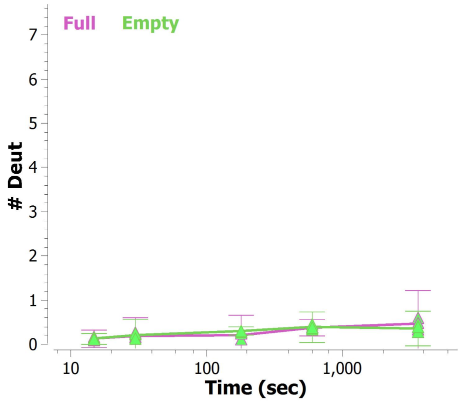

AAV8_VP1 622-637: AKIPHTDGNFHPSPLM (#328)

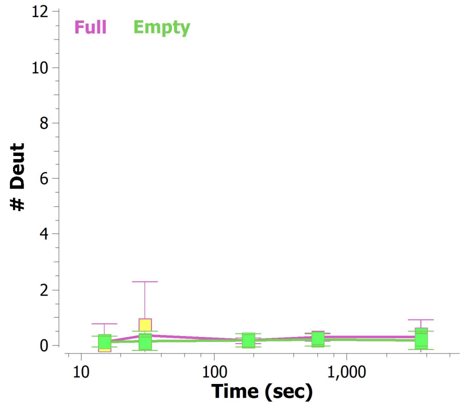

AAV8_VP1 622-640: AKIPHTDGNFHPSPLMGGF (#329)

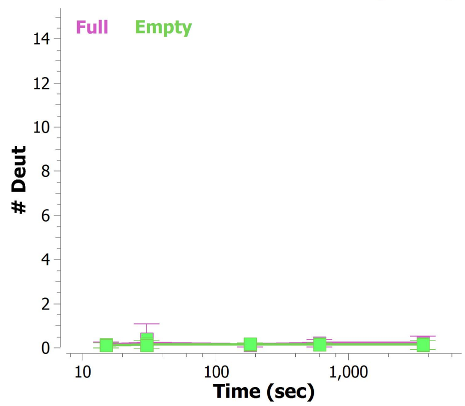

AAV8_VP1 622-650: KIPHTDGNFHPSPLMGGFGLKHPPPQI (#330)

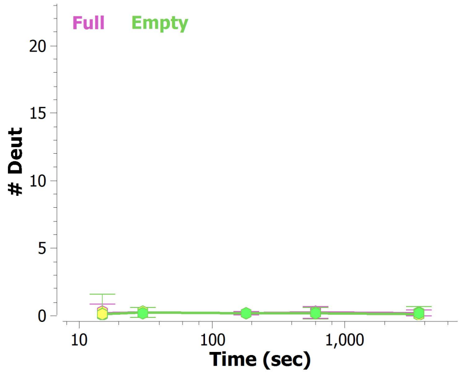

AAV8_VP1 638-650: GGFGLKHPPPQIL (#331)

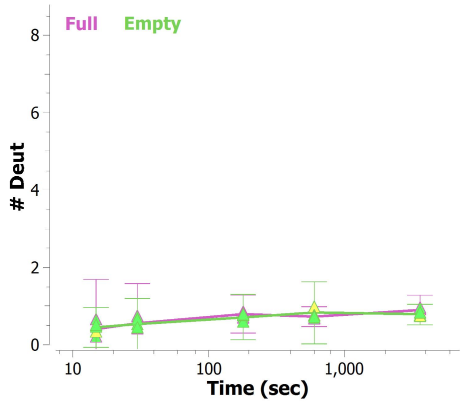

AAV8_VP1 640-650: FGLKHPPPQIL (#332)

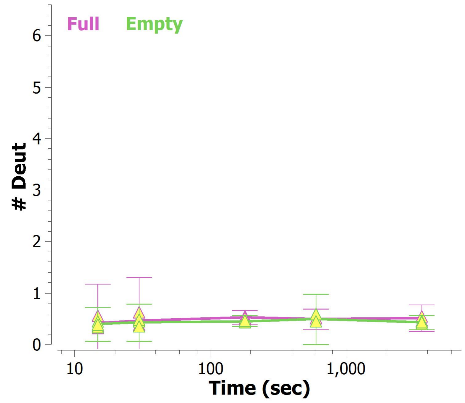

AAV8_VP1 641-650: GLKHPPPQIL (#333)

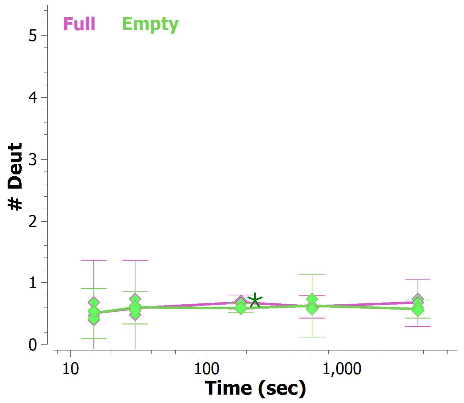

AAV8_VP1 650-671: LIKNTPVPADPPTTFNQSKLNS (#334)

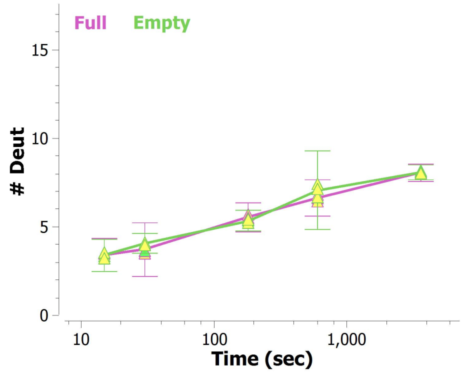

AAV8_VP1 651-664: IKNTPVPADPPTTF (#335)

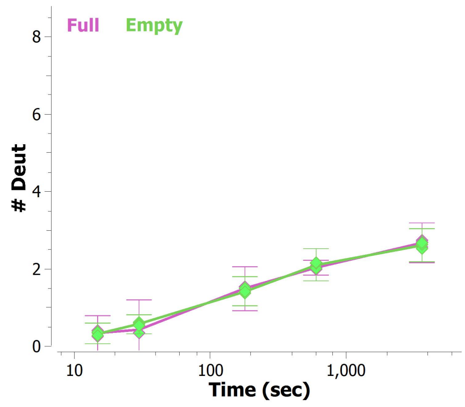

AAV8_VP1 651-665: IKNTPVPADPPTTFN (#336)

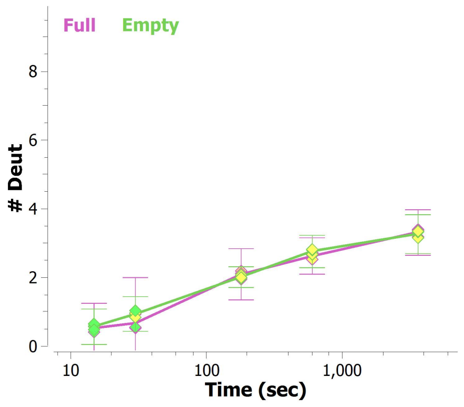

AAV8_VP1 651-666: IKNTPVPADPPTTFNQ (#337)

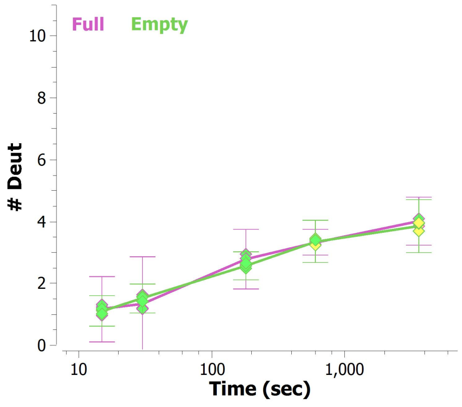

AAV8_VP1 651-669: IKNTPVPADPPTTFNQSKL (#338)

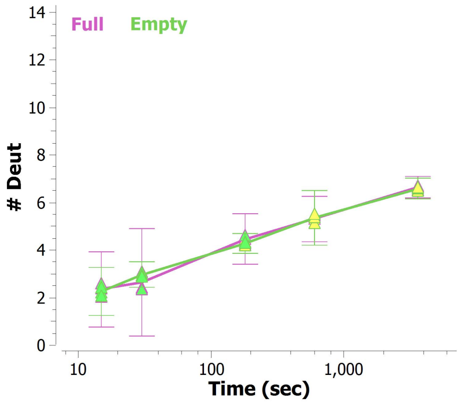

AAV8_VP1 651-671: IKNTPVPADPPTTFNQSKLNS (#339)

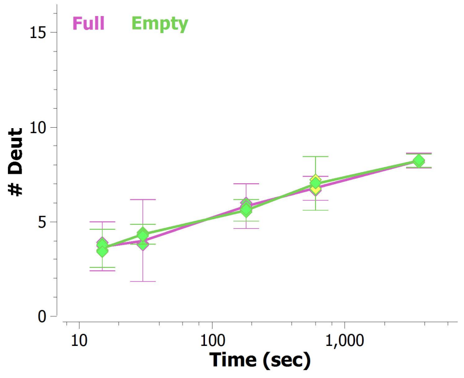

AAV8_VP1 651-672: IKNTPVPADPPTTFNQSKLNSF (#340)

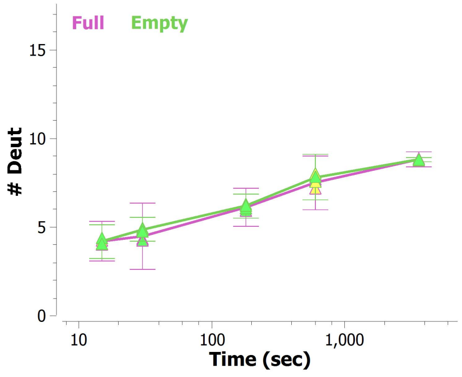

AAV8_VP1 665-684: NQSKLNSFITQYSTGQVSVE (#341)

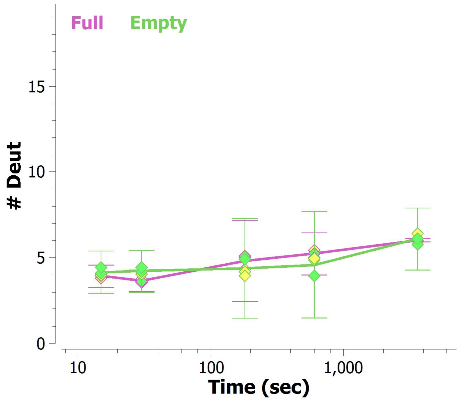

AAV8_VP1 670-675: NSFITQ (#342)

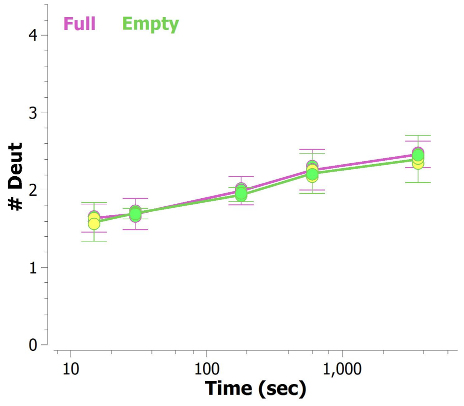

AAV8_VP1 670-683: NSFITQYSTGQVSV (#343)

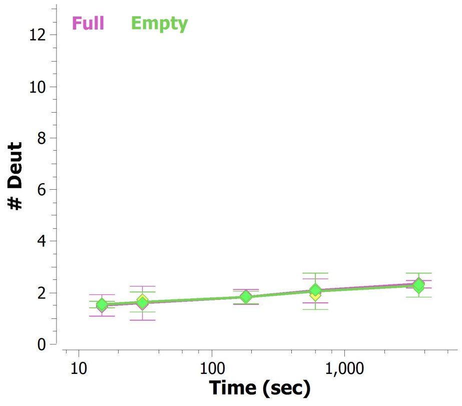

AAV8_VP1 672-675: FITQ (#344)

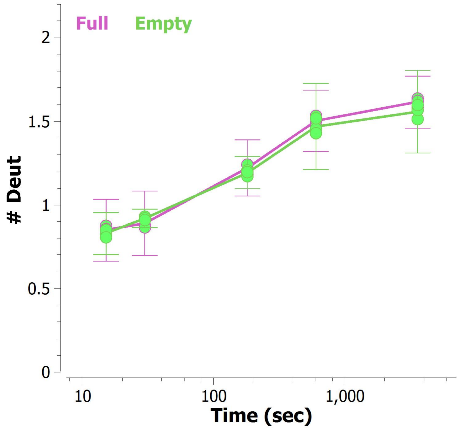

AAV8_VP1 672-682: FITQYSTGQVS (#345)

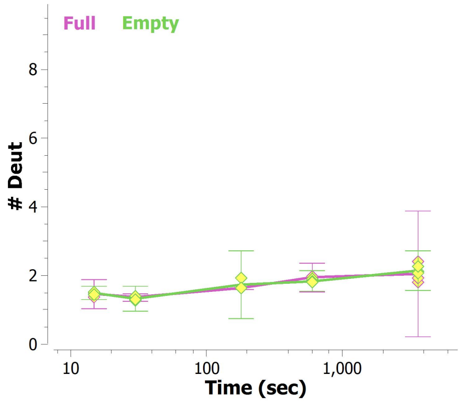

AAV8_VP1 672-683: FITQYSTGQVSV (#346)

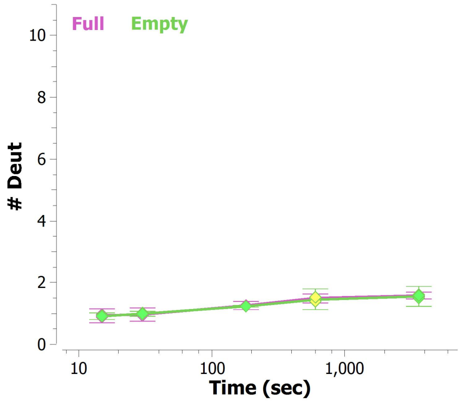

AAV8_VP1 672-684: FITQYSTGQVSVE (#347)

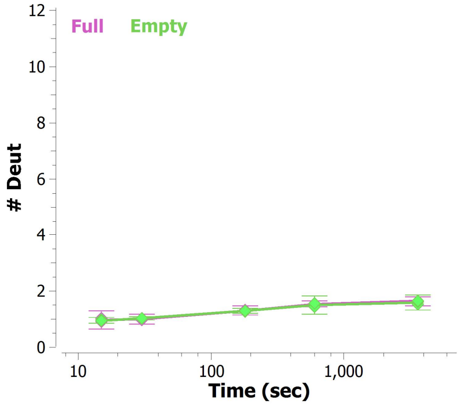

AAV8_VP1 673-676: ITQY (#348)

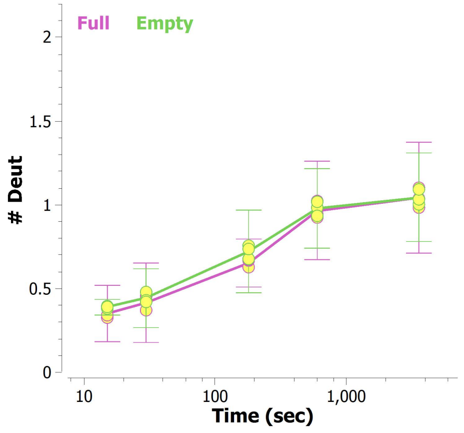

AAV8_VP1 673-683: ITQYSTGQVSV (#349)

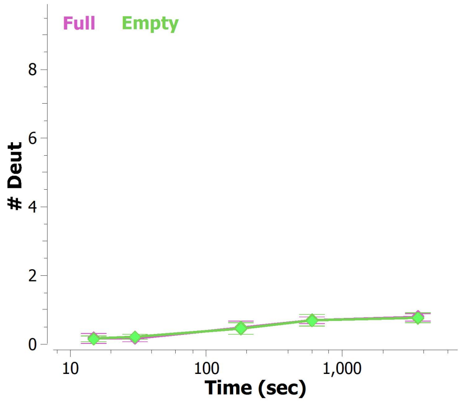

AAV8_VP1 673-684: ITQYSTGQVSVE (#350)

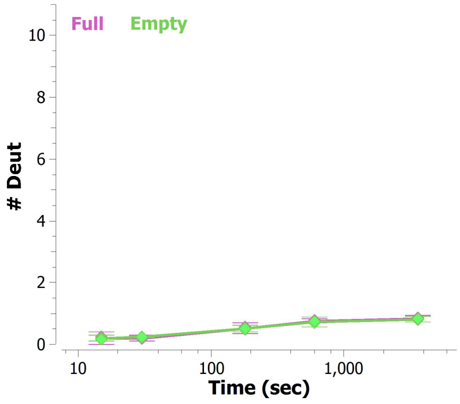

AAV8_VP1 676-683: YSTGQVSV (#351)

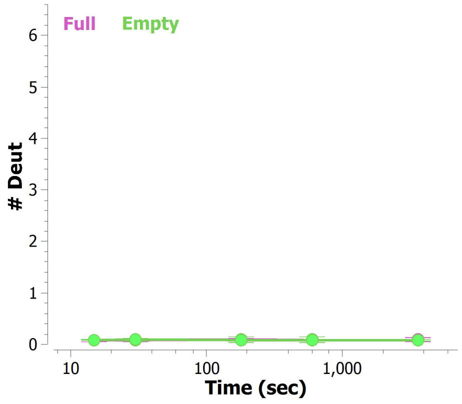

AAV8_VP1 676-684: YSTGQVSVE (#352)

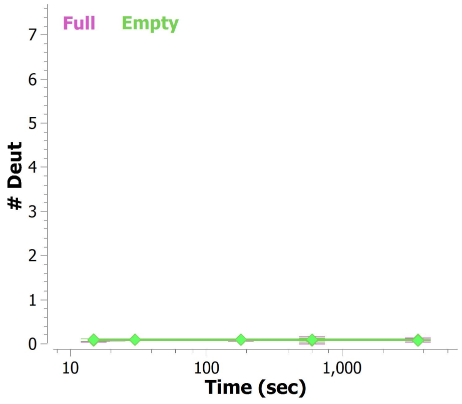

AAV8_VP1 677-684: STGQVSVE (#353)

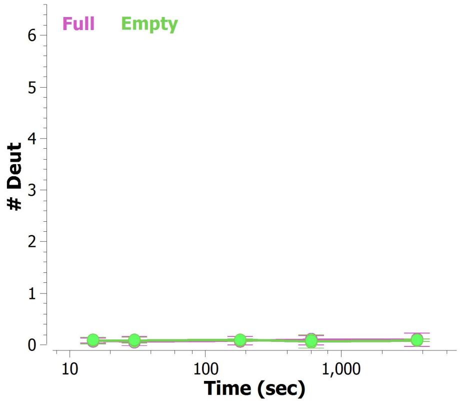

AAV8_VP1 683-689: VEIEWEL (#354)

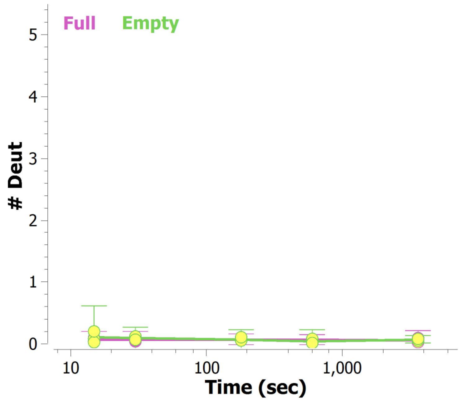

AAV8_VP1 683-703: VEIEWELQKENSKRWNPEIQY (#355)

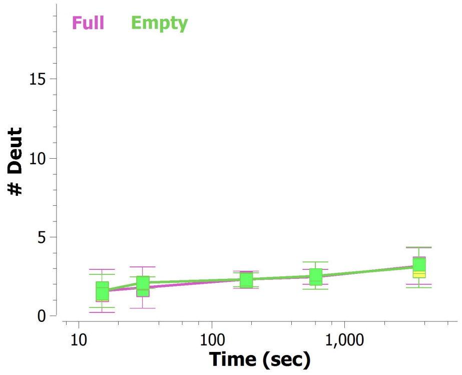

AAV8_VP1 684-689: EIEWEL (#356)

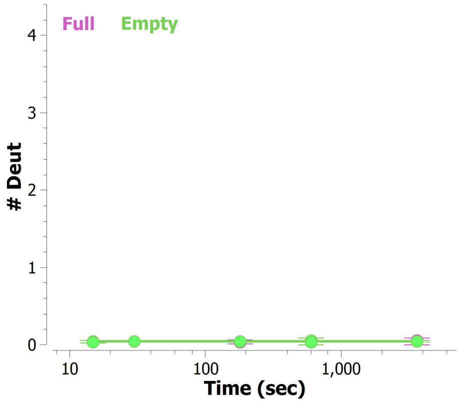

AAV8_VP1 684-702: EIEWELQKENSKRWNPEIQ (#357)

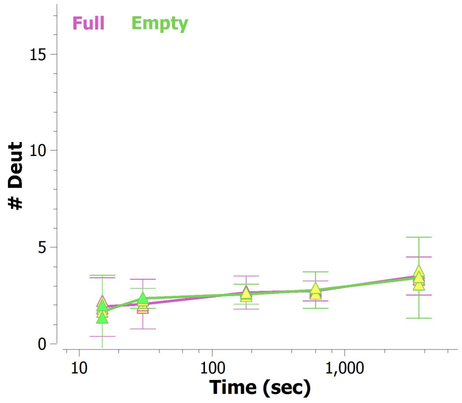

AAV8_VP1 684-703: EIEWELQKENSKRWNPEIQY (#358)

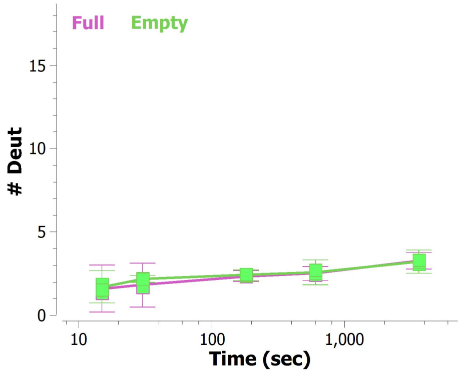

AAV8_VP1 684-705: EIEWELQKENSKRWNPEIQYTS (#359)

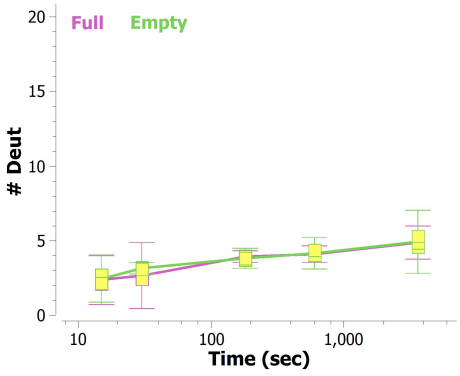

AAV8_VP1 684-706: EIEWELQKENSKRWNPEIQYTSN (#360)

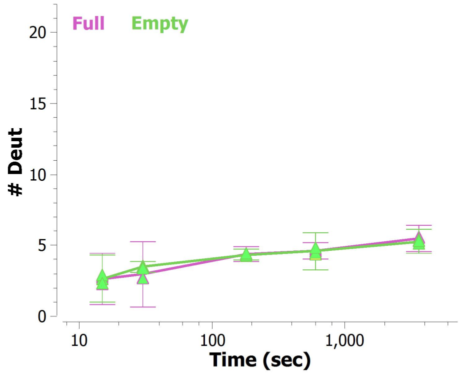

AAV8_VP1 684-707: EIEWELQKENSKRWNPEIQYTSNY (#361)

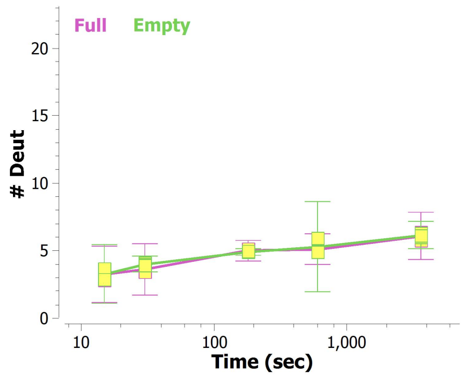

AAV8_VP1 685-689: IEWEL (#362)

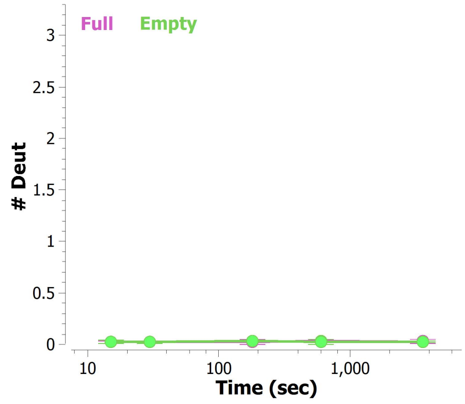

AAV8_VP1 685-702: IEWELQKENSKRWNPEIQ (#363)

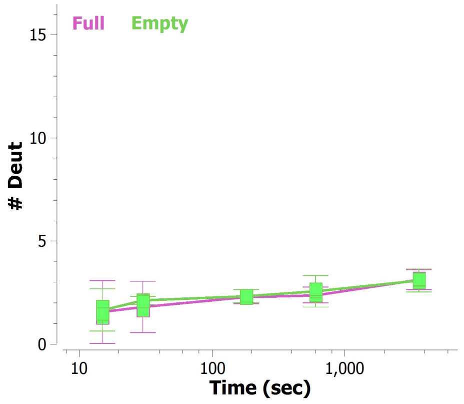

AAV8_VP1 685-703: IEWELQKENSKRWNPEIQY (#364)

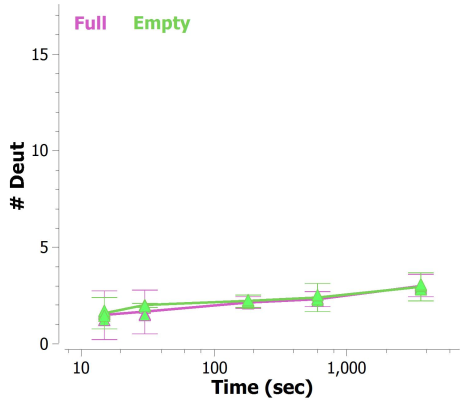

AAV8_VP1 685-706: IEWELQKENSKRWNPEIQYTSN (#365)

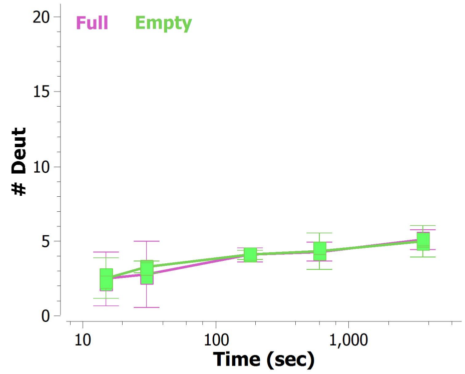

AAV8_VP1 685-707: IEWELQKENSKRWNPEIQYTSNY (#366)

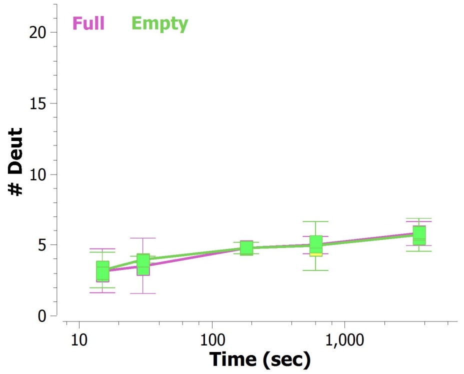

AAV8_VP1 687-703: WELQKENSKRWNPEIQY (#367)

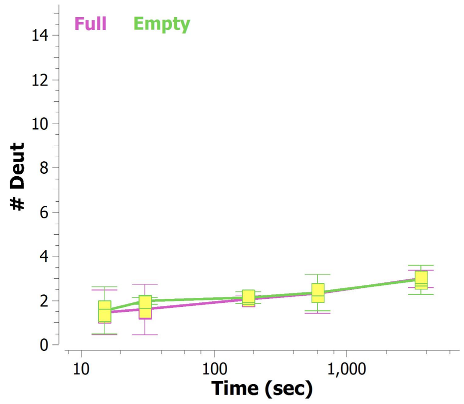

AAV8_VP1 687-706: WELQKENSKRWNPEIQYTSN (#368)

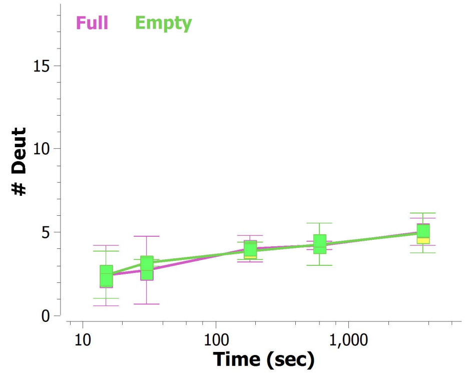

AAV8_VP1 688-703: ELQKENSKRWNPEIQY (#369)

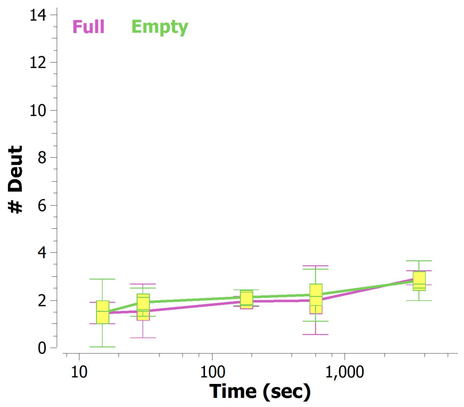

AAV8_VP1 688-706: ELQKENSKRWNPEIQYTSN (#370)

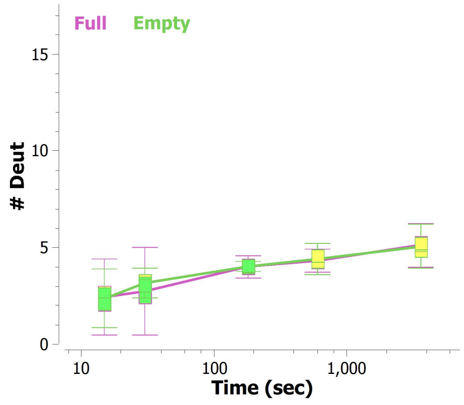

AAV8_VP1 689-706: LQKENSKRWNPEIQYTSN (#371)

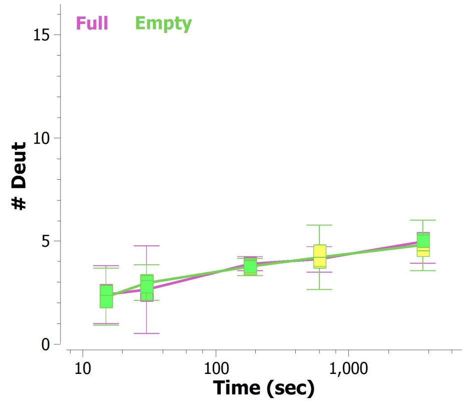

AAV8_VP1 690-702: QKENSKRWNPEIQ (#372)

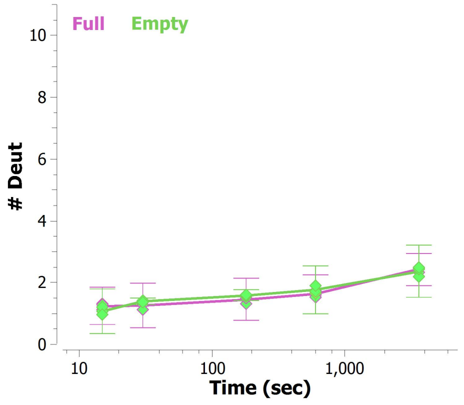

AAV8_VP1 690-703: QKENSKRWNPEIQY (#373)

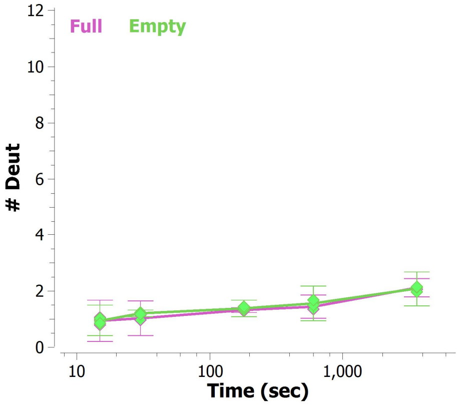

AAV8_VP1 690-706: QKENSKRWNPEIQYTSN (#374)

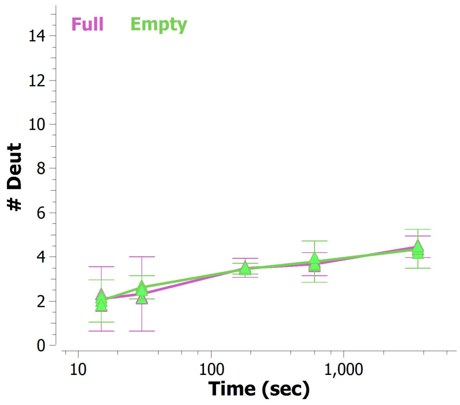

AAV8_VP1 690-707: QKENSKRWNPEIQYTSNY (#375)

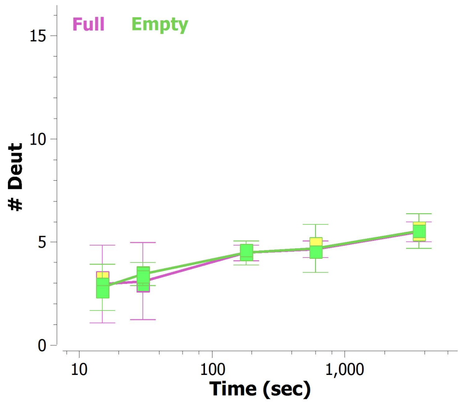

AAV8_VP1 691-703: KENSKRWNPEIQY (#376)

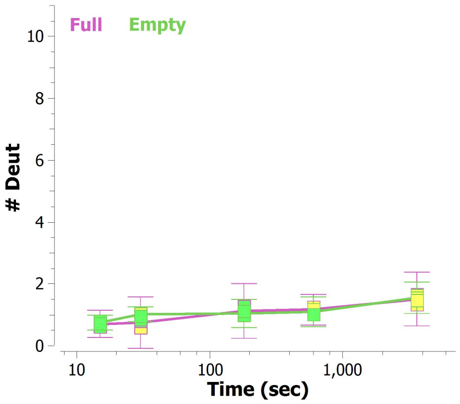

AAV8_VP1 695-703: KRWNPEIQY (#377)

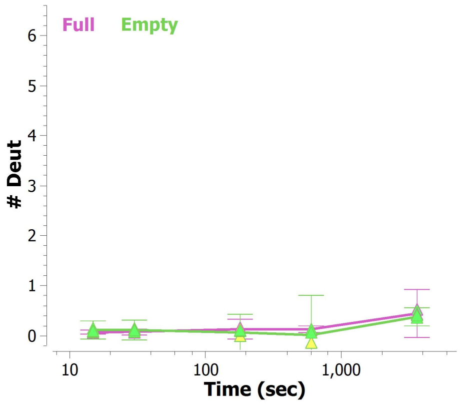

AAV8_VP1 695-706: KRWNPEIQYTSN (#378)

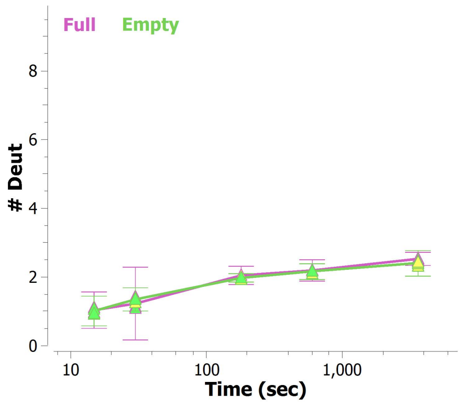

AAV8_VP1 703-715: YTSNYYKSTSVDF (#379)

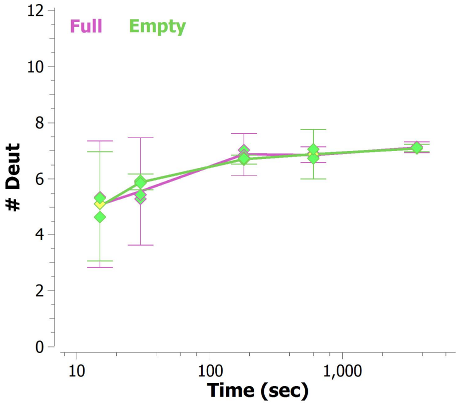

AAV8_VP1 704-715: TSNYYKSTSVDF (#380)

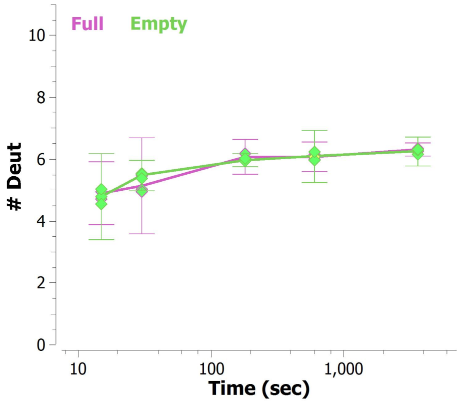

AAV8_VP1 707-714: YYKSTSVD (#381)

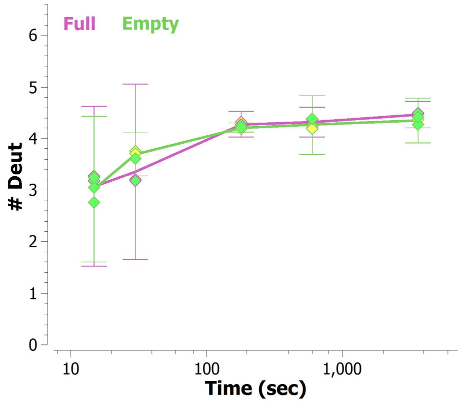

AAV8_VP1 707-715: YYKSTSVDF (#382)

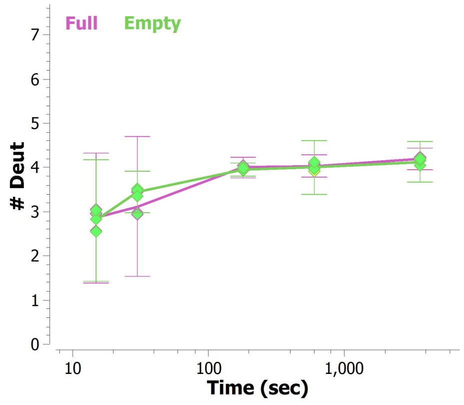

AAV8_VP1 708-715: YKSTSVDF (#383)

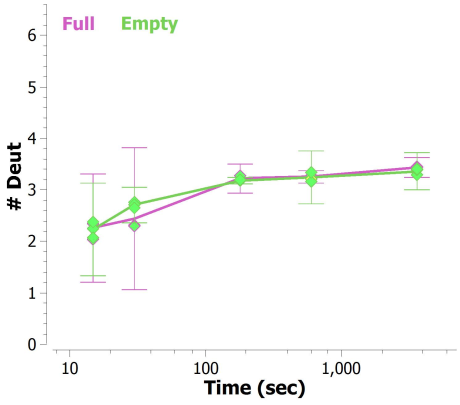

AAV8_VP1 715-722: FAVNTEGV (#384)

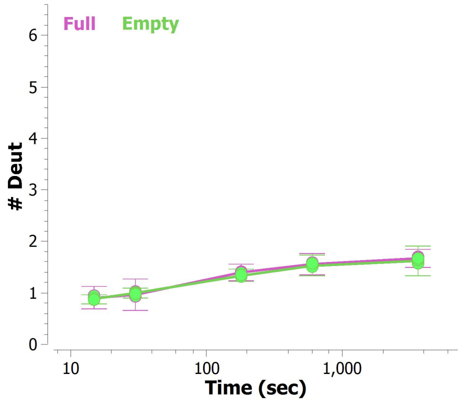

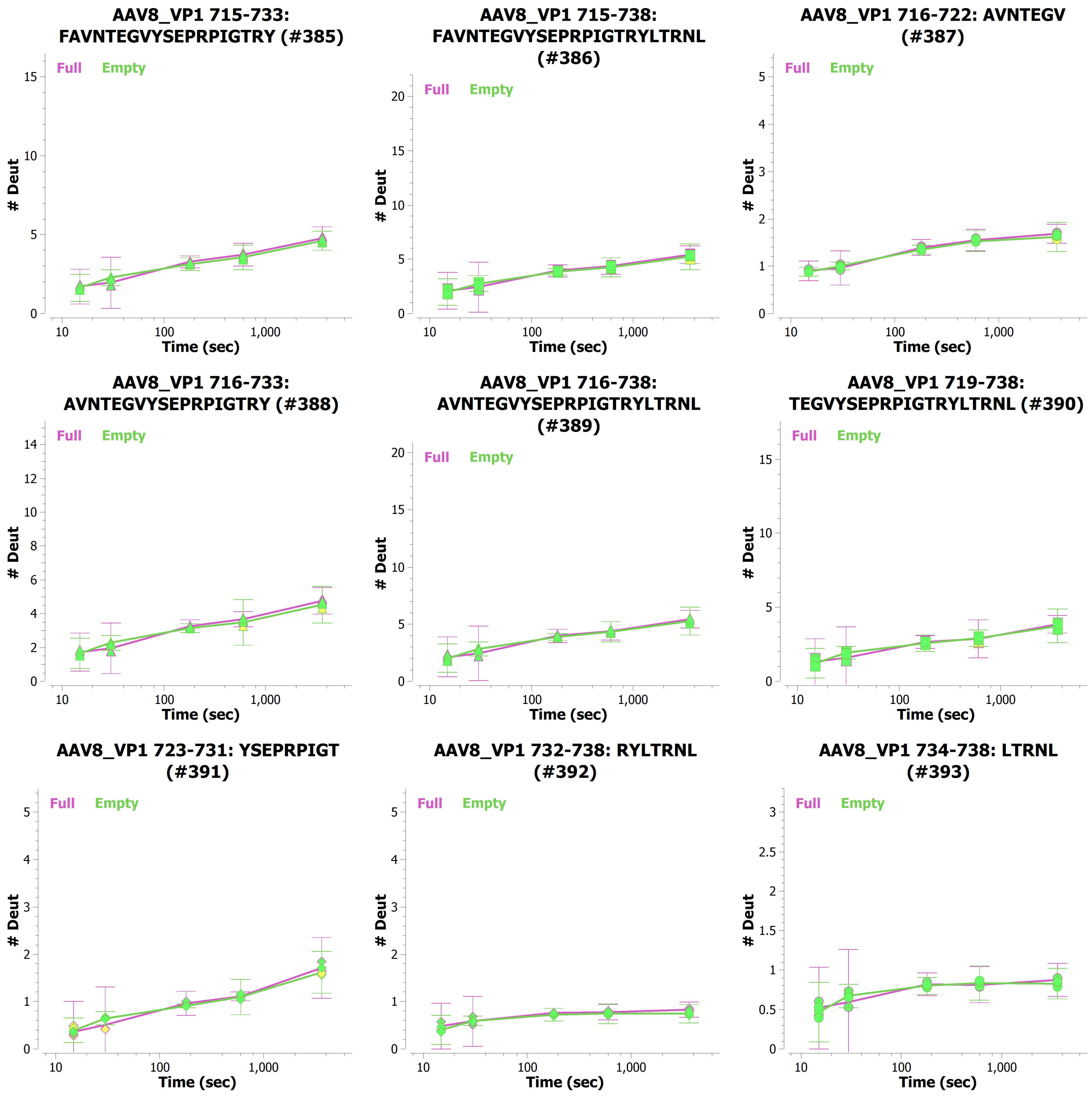

Supplementary Figure S7. Deuterium uptake plots of all peptides without any post translational modification except VP1 N-terminal peptides at a labeling temperature of 20 °C. Purple colored plots represents full capsid, and green colored plots represents empty capsid. Error bars representing standard deviation for each triplicate measurement are displayed.
