# Supplementary material for: Higher-Order Structure of Adeno-Associated Virus Serotype 8 by Hydrogen/Deuterium Exchange Mass Spectrometry
Source: Viruses. 2024 Apr 10;16(4):585. doi: 10.3390/v16040585 (PMC11053801; doi:10.3390/v16040585)
Supplement: Supplementary file 1 [file viruses-16-00585-s001.zip › Supplemental/Viruses_manuscript_Ikeda240327_supplementalfile.pdf]

Supplementary files

Higher-Order Structure of an Adeno-Associated Virus Serotype 8 by Hydrogen/Deuterium Exchange Mass Spectrometry

Tomohiko Ikeda [1], Yuki Yamaguchi [1], Hiroaki Oyama [1], Aoba Matsushita [1], Yasuo Tsunaka [1], Mitsuko Fukuhara [1], Tetsuo Torisu [1], and Susumu Uchiyama [1, 2, *]

[1] Department of Biotechnology, Graduate School of Engineering, Osaka University, 2-1 Yamadaoka, Suita, Osaka, Japan.
[2] Exploratory Research Center on Life and Living Systems (ExCELLS), National Institutes of Natural Sciences, 5-1 Higashiyama, Myodaiji, Okazaki, Aichi, 444-8787, Japan.
* Correspondence: suchi@bio.eng.osaka-u.ac.jp; Tel. +81-6-6879-4215

Supplementary figures

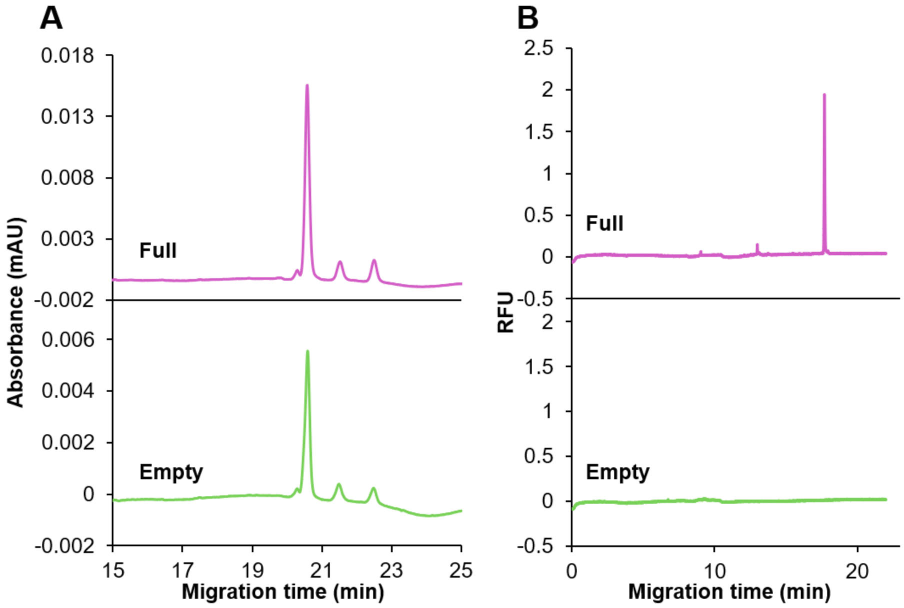

Supplementary Figure S1. rAAV8 sample characterization by capillary gel electrophoresis (CGE) with UV and LIF detector. (A) Electropherogram of CGE for the characterization of capsid proteins. (B) Electropherogram of CGE for the characterization of the encapsidated genome of capsid proteins. The results of upper panel are full sample (colored purple) and of lower panel are empty sample (colored green).

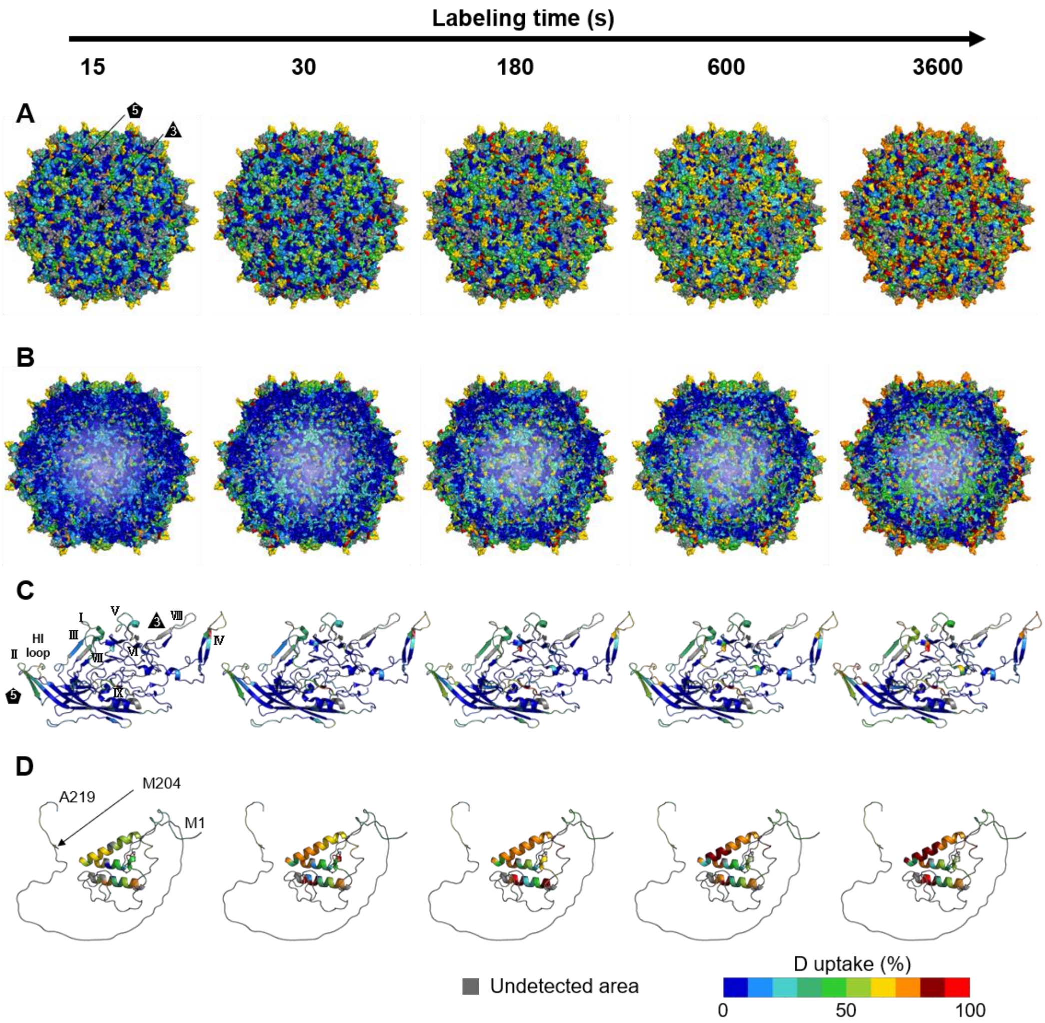

Supplementary Figure S2. Structural dynamics of rAAV8 full capsids at a labeling temperature of 20 °C over the labeling time course. The structural dynamics of the rAAV8 full capsid mapped onto the assembled full capsid (PBD ID: 6PWA) external surface (A) and internal surface which is a cross-sectional view of a capsid (B), VP3 monomer (C), and model of the structurally unsolved region (D). The VP3 monomers are enlarged views of a VP3 taken from a capsid composed of 60 VPs. The predicted model of the structurally unsolved region is magnified. The characteristic structures of rAAV capsids, such as 3-fold and 5-fold symmetry axes and VRs, are shown. The regions where the peptides were not detected are colored gray.

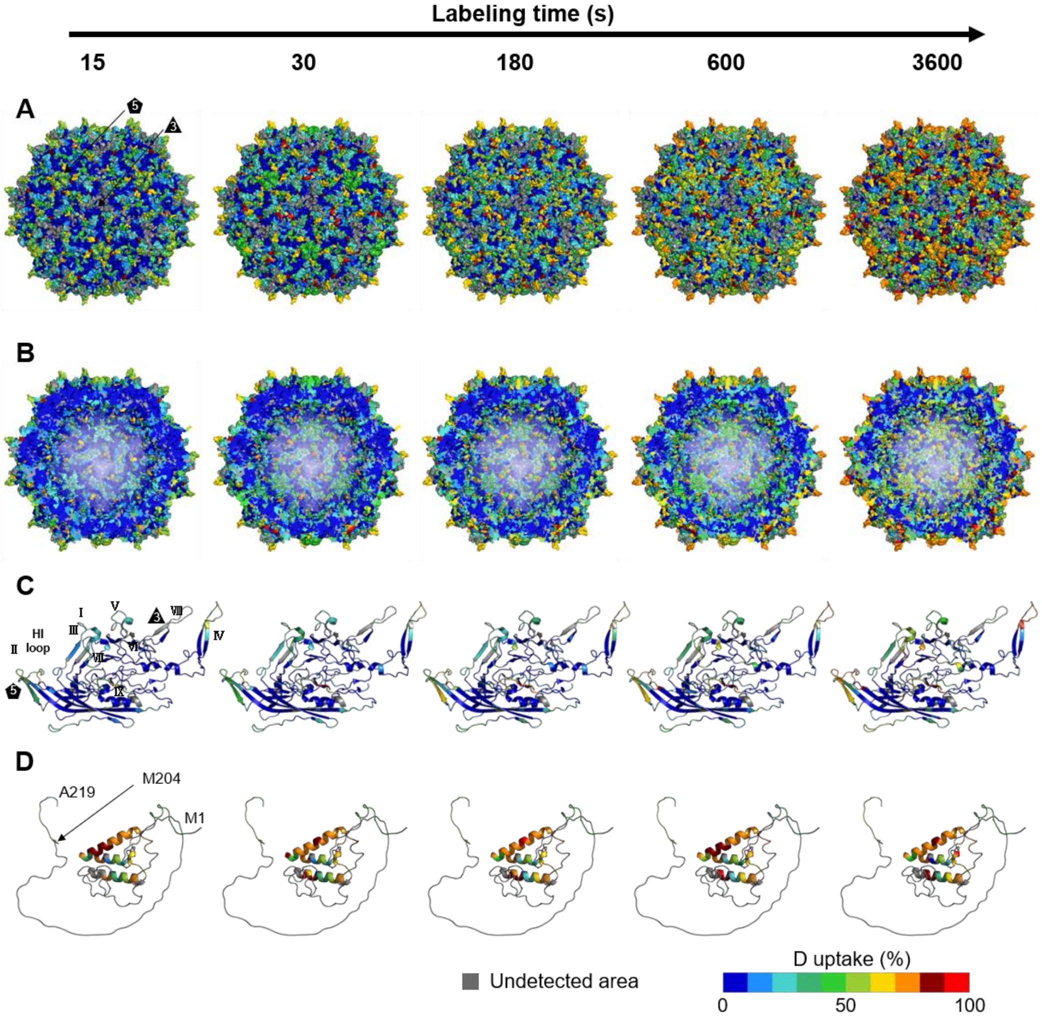

Supplementary Figure S3. Structural dynamics of rAAV8 empty capsids at a labeling temperature of 20 °C over the labeling time course. The structural dynamics of the rAAV8 empty capsid mapped onto the assembled empty capsid (PDB ID: 6U20) external surface (A) and internal surface which is a cross-sectional view of a capsid (B), VP3 monomer (C), and model of the structurally unsolved region (D). The VP3 monomers are enlarged views of a VP3 taken from a capsid composed of 60 VPs. The predicted model of the structurally unsolved region is magnified. The characteristic structures of rAAV capsids, such as 3-fold and 5-fold symmetry axes and VRs, are shown. The regions where the peptides were not detected are colored gray.

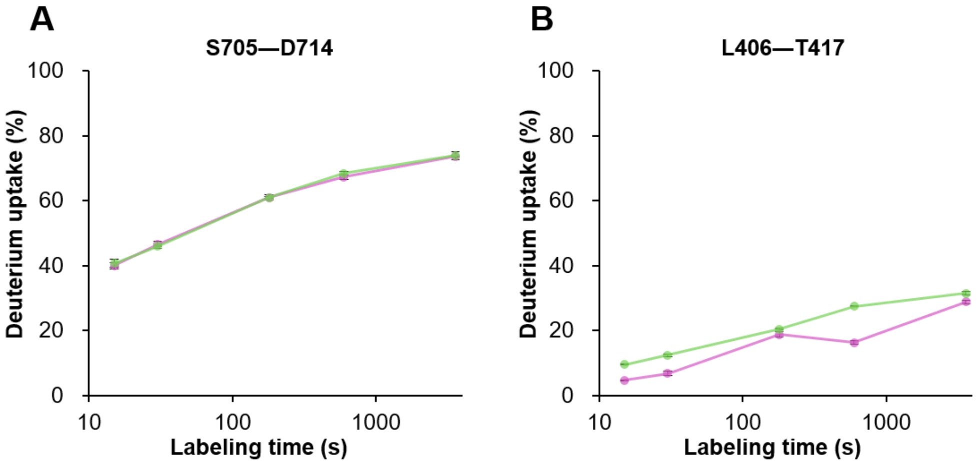

Supplementary Figure S4. Uptake plots of peptides derived from outer and inner surface of capsids. (A) Uptake plot of the S705–D714 peptides derived from outer surface of capsid of rAAV8 full in purple and empty in green capsids. (B) Uptake plot of the L406–T417 peptides derived from inner surface of capsid of rAAV8 full in purple and empty in green capsids.
